# The Comparative Analyses of Six Complete Chloroplast Genomes of Morphologically Diverse *Chenopodium album* L. (Amaranthaceae) Collected in Korea

**DOI:** 10.1155/2021/6643444

**Published:** 2021-04-27

**Authors:** Jongsun Park, Juhyeon Min, Yongsung Kim, Youngjae Chung

**Affiliations:** ^1^InfoBoss Inc., 301 Room, 670, Seolleung-ro, Gangnam-gu, Seoul, Republic of Korea; ^2^InfoBoss Research Center, 301 Room, 670, Seolleung-ro, Gangnam-gu, Seoul, Republic of Korea; ^3^Department of Biological Sciences, Sungkyunkwan University, Suwon, Republic of Korea; ^4^Department of Biology, Shingyeong University, Hwaseong 18274, Republic of Korea

## Abstract

*Chenopodium album* sensu stricto belonging to *C. album* aggregate is an annual cosmopolitan weed displaying the diversity of morphologies. We completed the six chloroplast genomes of *C. album* s. str. collected in Korea to understand the relationship between the diversity of chloroplast genomes and their morphological variations. All six *C. album* chloroplast genomes have a typical quadripartite structure with length ranging from 151,906 bp to 152,199 bp, similar to the previously sequenced *C. album* chloroplast genome (NC_034950). In total, 56 single nucleotide polymorphisms (SNPs) and 26 insertion and deletion (INDEL) regions (308 bp in total) were identified from the six chloroplast genomes, presenting a low level of intraspecific variations in comparison to the other angiosperm species. 376 normal simple sequence repeats were identified in all seven *C. album* chloroplast genomes. The phylogenetic analysis based on all available complete Amaranthaceae chloroplast genomes presents phylogenetic positions of six *C. album* samples as well as correlation with one of *C. album* morphological features. Our results provide the way to investigate intraspecific features of *C. album* chloroplast genomes and also the insights of understanding various intraspecific characteristics including morphological features.

## 1. Introduction

*Chenopodium album* aggregate, also known as *Chenopodium album* sensu lato, is one of the challenging groups for delimitating species boundary by phenotypes [1]. The main possibility of its phenotypic diversity is hybridization and polyploidization [2] because this group shows a diploid-polyploid complex ranging from diploids to decaploids with individual species which are very tightly correlated with a specific ploidy level and genome size [3–5]. Genetic diversity of *C. album* aggregate has been analyzed displaying that at least eight groups were identified, and their evolutionary lineages were supported well using flowering locus T-like (FTL) intron and chloroplast DNA maker sequences [2]. In addition, Central Asia presents the most diverse haplotypes, indicating that this area can be an origin of *C. album* aggregate [2].

*C. album* sensu stricto belonging to *C. album* aggregate is an annual cosmopolitan weed native to Eurasia [6]. It is one of the notorious weeds which reduce crop yield by exploiting resources such as light and nutrients from soil [6]. In contrast, *C. album* s. str. has also been cultivated as a crop in some countries: it was especially considered better nutritional crop species than wheat, barley, maize, and rice in the Himalaya area [7–9]. Besides it, *C. album* s. str. was also cultivated as a leafy vegetable [9, 10].

*C. album* s. str. has been recognized as a morphologically diverse species with difficulty in species identification [1, 11, 12]. This hexaploid species exhibited wide phenotypic plasticity covering morphological variations of other *Chenopodium* species, which shows stable morphologies under the greenhouse environment [1]. This high-level phenotypic variation of *C. album* s. str. can be explained by its allopolyploid originated from maternal tetraploids and paternal diploid parents [13]. Even though a few studies to understand the origin of *C. album* s. str. in the evolutionary aspect have been conducted [13, 14], there is no research to unravel the relationship between its morphological plasticity and the genetic diversity of organelle genomes.

Due to low sequencing cost caused by rapid development of next-generation sequencing (NGS) and third-generation sequencing (TGS) technologies [15–17] as well as the conserved structure of chloroplast genome reducing difficulties of *de novo* assembly, a huge number of chloroplast genomes have been massively sequenced. One of the evidences which reflects this situation is that the number of angiosperm complete chloroplast genomes deposited in NCBI is 9,323 covering 6,727 species as of 11 Jan 2021. These chloroplast genome sequences have been analyzed for the identification of phylogenetic positions [18–20] and for developing useful molecular markers [21, 22]. It presents that sequence variations of complete chloroplast genomes are enough information to conduct the studies for those purposes that complete chloroplast genomes can be also used to reveal the relationship between morphological plasticity and genetic diversity of *C. album* s. str.

Here, we completed the six chloroplast genomes of *C. album* s. str. of which both morphological features and geographical locations in the Korean Peninsula are distinct from each other. The *C. album* chloroplast genomes display a relatively low level of intraspecific variations (56 single nucleotide polymorphisms (SNPs) and 26 insertion and deletion (INDEL) regions across the seven *C. album* chloroplast genomes) with low nucleotide diversity compared to those of other angiosperm chloroplast genomes. 376 normal simple sequence repeats (SSRs), including two individual-specific normal SSRs, are detected among the seven chloroplast genomes, and the numbers of SSRs and their distribution on each *C. album* chloroplast genome are similar. Maximum likelihood and Bayesian inference phylogenetic trees based on all available complete Amaranthaceae chloroplast genomes present that seven *C. album* chloroplast genomes are clustered with short branch length and high supportive values. However, the correlation between their phylogenetic position and morphological features is not so strong. Hence, morphological plasticity of *C. album* which may not be explained by the genetic diversity of chloroplast genomes requires additional sequence data, such as nuclear marker sequences or specific genes which regulate leaf morphologies, to understand the origin of morphological plasticity.

## 2. Results

### 2.1. Six Complete Chloroplast Genomes and Their Morphological Features of *Chenopodium album* Collected in Korea

**Figure 1:**
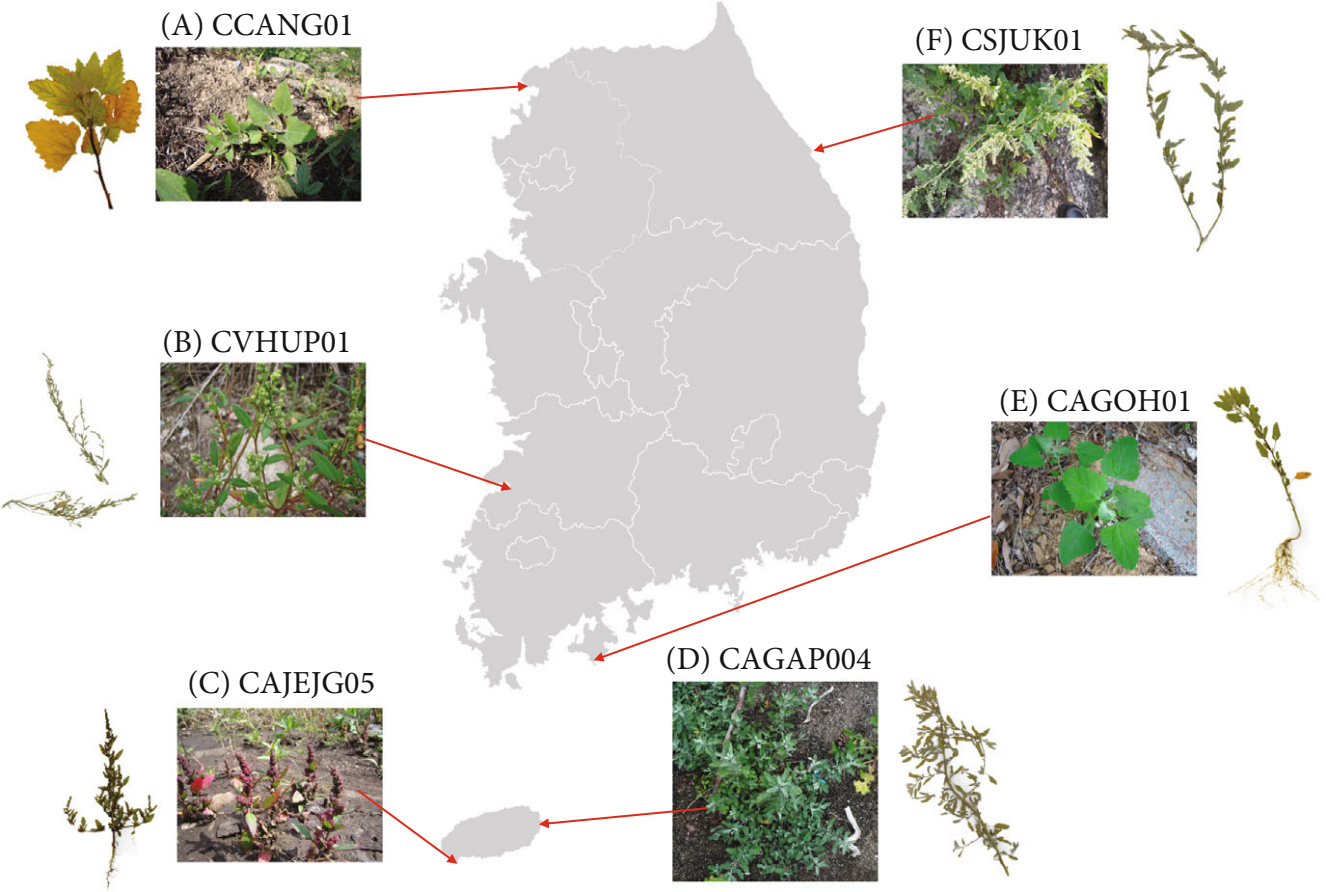
Geographical distribution of six *Chenopodium album*. The red arrows indicate the locations where six *C. album* samples were collected in South Korea. Pictures of voucher specimen were displayed on the left or right side of pictures habit *in situ* of the six samples.

Six samples of *C. album* collected in Korea named CAGAP004, CAGOH01, CAJEJG05, CCANG01, CSJUK01, and CVHUP01 were selected based on their distinct morphological features and collected in different geographical positions in Korean Peninsula (Table 1 and Figure 1). The leaf shape of the six *C. album* samples presents a wide range which is from lanceolate to ovate. Their leaf margin shows the two types: (i) serrate (CAGAP004, CAGOH01, and CCANG01) and (ii) entire (CAJEJG05, CSJUK01, and CVHUP01; Figure 1 and Table 1). In addition, the thickness of leaves of the six samples is also divided into two types: (i) thick type (CAGAP004, CAJEJG05, and CVHUP01) and (ii) thin type (CAGOH01, CCANG01, and CSJUK01).

**Figure 2:**
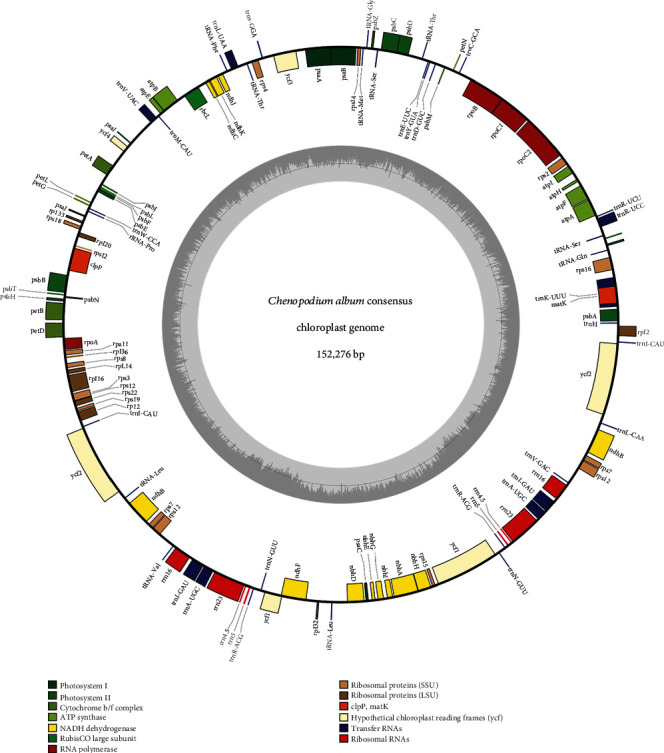
A circular gene map of six *Chenopodium album* chloroplast genomes. Genes shown outside are transcribed clockwise, and inside the circle are transcribed counterclockwise. Genes are color-coded to distinguish different functional groups. The dark grey and the light grey plots in the inner circle correspond to the GC content and AT content, respectively.

To understand the relationship between morphological features with geographical distribution and its genetic background of chloroplast genomes, we completed chloroplast genomes of the six *C. album* samples (Table 1). The six chloroplast genomes have a typical quadripartite structure which has one large single-copy (LSC), one small single-copy, and two inverted repeat (IR) regions (Figure 2). The length of the six chloroplast genomes ranges from 151,906 bp (CCANG01) to 152,199 bp (CAGAP004; Table 2), presenting 293 bp differences. They are similar to that of one of the two previously sequenced *C. album* chloroplast genomes, of which GenBank accession is NC_034950 (152,167 bp) [23]. Interestingly, the other chloroplast genome of *C. album*, MF418659, displays much shorter (150,272 bp) than those of the remaining *C. album* chloroplast genomes [24]. Their overall GC contents are conserved as between 37.2% and 37.3%, which are slightly higher than that of MF418659 (37.0%; Table 2). The GC contents of LSC and SSC regions are identical in the seven *C. album* chloroplast genomes including the firstly sequenced chloroplast of *C. album* (NC_034950), which are 35.3% and 31.0%, respectively. Similarly, GC contents in the IR region of the seven chloroplast genomes are from 42.7% to 42.8% (that of MF418659 is 43.4%).

All seven *C. album* chloroplast genomes including NC_034950 contain 129 genes including 84 protein-coding genes (PGCs), 8 ribosomal RNAs (rRNAs), and 37 transfer RNAs (tRNAs; Table 3). Seventeen genes are duplicated in IR regions including 6 PCGs (*rpl2*, *ycf2*, *ndhB*, *rps12*, *rps7*, and *ycf1)*, 4 rRNAs (*rrn16*, *rrn23*, *rrn4.5*, and *rrn5*), and 7 tRNAs (*trnI-CAU*, *trnL-CAA*, *trnV-GAC*, *trnI-GAU*, *trnA-UGC*, *trnR-ACG*, and *trnN-GUU*). The number of PCGs from the seven *C. album* chloroplast genomes is the same except MF418659 (Table 3), presenting that MF418659 has a quite different gene configuration. It has six additional PCGs, *rpl23*, *ycf15*, and *ycf68* in the IR region, and loses *psaJ* PCG. In addition, it also misses one of the two *rps12*, displaying the same sequences of three exons of *rps12* to the rest seven *C. album* chloroplast genomes; however, this missed *rps12* should be added in the annotation of MF418659, resulting in 89 PCGs. After reannotation of two *Chenopodium quinoa* chloroplast genomes (KY635884 and MF805727), one *ycf1* and four additional PCGs, *ycf1*, *ycf2*, and two *rps12*, are added, respectively, resulting in all chloroplast genomes of *Chenopodium* species having the same number of genes except MF418659 (Table 3).

In the seven *C. album* chloroplast genomes, there are six ATP synthase subunit genes, 11 NADH dehydrogenase genes, four RNA polymerase genes, six genes encoding subunits of cytochrome b/f complex, and 15 photosystem subunit II genes. Five genes encoding photosystem subunit I are found in the seven *C. album* chloroplast genomes (Table 4), while MF418659 has only four genes with losing *psaJ*. There are 20 ribosomal proteins presented in the seven *C. album* chloroplast genomes consisting of 12 ribosomal proteins encoding small subunit and eight genes for large subunits (Table 4). Interestingly, an additional *rpl23* gene is found only in the MF418659 chloroplast genome. Other remaining genes encode acetyl-CoA-carboxylase (*accD*), translation initiation factor (*infA*), protease (*clpP*), chloroplast envelope membrane protein (*cemA*), maturase K gene (*matK*), and cytochrome c biogenesis protein (*ccsA*; Table 4). The number of hypothetical proteins is four among the seven *C. album* chloroplast genomes, except for MF418659 which has six genes (Table 4), presenting the different gene configuration of MF418659.

In the seven *C. album* chloroplast genomes, nine PCGs contain one intron (*rps16*, *atpF*, *rpoC1*, *petB*, *petD*, *rpl16*, *rps12*, *ndhB*, and *ndhA*) and only *clpP* and *ycf3* have two introns, which are conserved across chloroplast genomes of the other *Chenopodium* species. MF418659 chloroplast also has the same intron structure as the remaining *Chenopodium* chloroplast genomes. Taken together with the different properties of the MF418659 chloroplast genome including its length, GC ratio, and a number of genes, we suspected that MF418659 chloroplast genome might not be *C. album*; hence, we will exclude MF418659 for further analyses conducted in this study.

### 2.2. Nucleotide Diversity and Intraspecific Variations Identified from the Seven *Chenopodium album* Chloroplast Genomes

**Figure 3:**
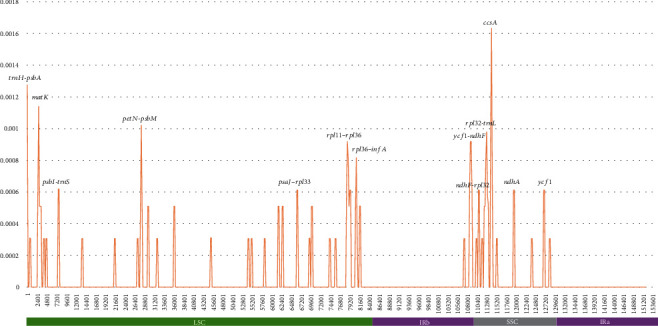
Nucleotide diversity of the seven *Chenopodium album* chloroplast genomes. The *X*-axis presents coordination of chloroplast genome, and the *Y*-axis presents values of nucleotide diversity value of 500 bp window sliding by 200 bp step. Highest peaks of the nucleotide diversity graph display the name of genic regions. Green, pink, and grey bars below the *X*-axis indicate the LSC, IR, and SSC regions, respectively.

To investigate nucleotide diversity (*π*) and intraspecific variations of *C. album* chloroplast genomes, the six *C. album* complete chloroplast genomes sequenced in this study are aligned against the previously sequenced *C. album* chloroplast genome (NC_034950). The average value of nucleotide diversity is 0.0000625 (Figure 3), and a total of 56 single nucleotide polymorphisms (SNPs) and 26 insertion and deletion (INDEL) regions (308 bp in total) are identified. The LSC region, where the average nucleotide diversity is the highest (*π* = 0.00102), contains 35 SNPs (62.5%) and 16 INDEL regions (47 bp in length; 59.3%). Fifteen SNPs (26.8%) and 5 INDEL regions (193 bp in length; 8.93%) are found in the SSC region, displaying that the number of SNPs in the LSC region is larger more than twice that of the SSC region; however, the total length of INDEL regions in the SSC region is about 4 times greater than that of the LSC region. The main reason for this phenomenon is the presence of the 162 bp INDEL located between *rpl32* and *trnL-UAG* genes. An IR region covers three SNPs and three INDEL regions (68 bp in length), which corresponds to the lowest nucleotide diversity in the IR region (*π* = 0.0000146). The low level of sequence variations in the IR region is known as a general phenomenon in the chloroplast genomes [25–27].

Twenty-six out of 56 SNPs (46.4%) are located in PCG regions, while seven SNPs (12.5%) are in intronic and the 24 SNPs (42.9%) are in intergenic regions. As one SNP classified as nonsynonymous SNP is also classified into intronic SNP because *trnK-UUU* contains two exons located before and after *matK*, the total number of SNPs mentioned above is 57. The numbers of SNP per one kilobase pairs (Kbp) of the PCG, intronic, and intergenic regions are 0.329 SNPs/Kbp, 0.305 SNPs/Kbp, and 0.552 SNPs/Kbp, respectively, presenting that the intergenic region shows the highest density of SNPs. Eleven PCGs (*psbA*, *rpl16*, *clpP*, *infA*, *rps11*, *petA*, *ndhD*, *psbA*, *rpoC1*, *rps16*, and *atpB*) contain only one SNP in their coding region, while four genes (*matK*, *ccsA*, *ycf1*, and *ndhF*) cover more than one SNP. In addition, three PCGs (*clpP*, *petB*, and *ndhA*) have one SNP in their intronic region, while *rpl16* has two SNPs in their intronic region. In total, 15 nonsynonymous SNPs and 11 synonymous SNPs are identified in the 14 PCGs. Remarkably, a ratio of nonsynonymous to synonymous SNPs found on *C. album* chloroplast genomes is 15 : 11, which is different from the common phenomenon that the number of nonsynonymous SNPs is smaller than that of synonymous SNPs on the other plant chloroplast genomes [26, 28]. Each synonymous SNP is scattered in the eleven genes (*psbA*, *rps16*, *rpoC1*, *atpB*, *petA*, *clpP*, *rps11*, *rpl16*, *ndhF*, *ccsA*, and *ndhD*), while 15 nonsynonymous SNPs are concentrated only in the five genes (*matK*, *infA*, *ycf1*, *ndhF*, and *ccsA*). *Ycf1*, which is duplicated in the IR region and extends between IR and SSC regions, contains three nonsynonymous SNPs in each pair of the IR region, and one additional nonsynonymous SNP in the SSC region. The PCG containing the largest SNPs is *ndhF*, which contains five SNPs and is located mainly in the SSC region and partially extended over the IR region. Four out of the five SNPs identified in *ndhF* are nonsynonymous, among which one SNP found in the IR region is commonly shared with *ycf1*. Three PCGs (*psbA*, *matK*, and *ycf1*) have one INDEL region (11.5%) of which lengths are 6 bp, 6 bp, and 27 bp, respectively: none of these INDEL regions cause any frameshift mutation. Seven INDEL regions (26.9%) are identified in the intronic regions of *rps16*, *atpF*, *ycf3*, *clpP*, and *rpl16*. Most of them are less than 5 bp long; however, 66 bp INDEL is found in the intron of *trnI-GAU* in each IR region. The remaining 16 INDEL regions (61.5%) are intergenic. The longest INDEL region is 162 bp long, detected between *rpl32* and *trnL-UAG*.

There are eight sites that have relatively higher *π* values (>0.0008) including two PCGs *matK* (*π* = 0.00114) and *ccsA* (*π* = 0.00163) and six intergenic regions (*trnH-psbA*, *petN-psbM*, *rpl11-rpl36*, *rpl36-infA*, *ycf1-ndhF*, and *rpl32-trnL*; Figure 3). *CcsA* contains two nonsynonymous SNPs and one synonymous SNP, and *matK* has two nonsynonymous SNPs and one 6 bp INDEL, presenting that *ccsA* displays the highest SNP density among the PCGs. The highest *π* value of the intergenic region is observed between *trnH* and *psbA* (*π* = 0.00128; Figure 3).

### 2.3. Comparative Analysis of Simple Sequence Repeat (SSR) Polymorphisms on Chloroplast Genomes of *C. album*

**Figure 4:**
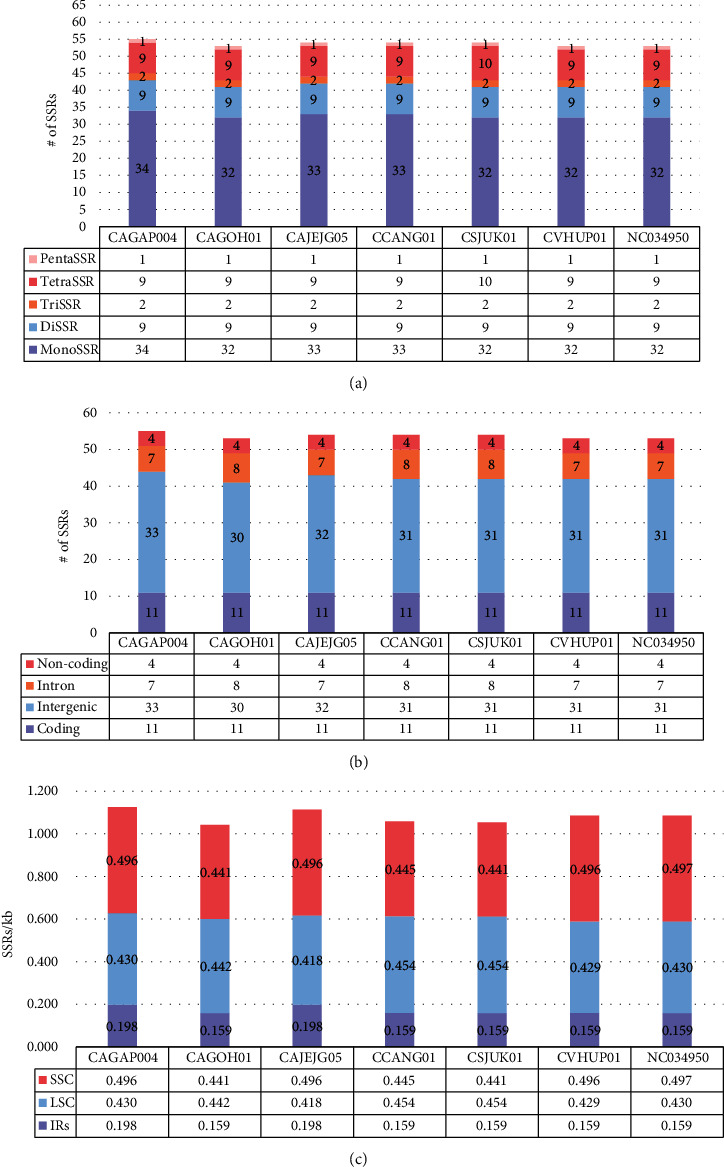
Number of normal SSRs identified on the seven *Chenopodium album* chloroplast genomes. (a) Displays the number of SSR types in each seven *C. album* chloroplast genome. The *X*-axis means the seven *C. album* samples, and the *Y*-axis indicates the number of normal SSRs. Five different colors indicate the five types of SSRs, monoSSRs, diSSRs, triSSRs, tetraSSRs, and pentaSSRs. The table below the *X*-axis presents numbers of SSRs along with samples and the five SSR types. (b) Shows the distribution of SSRs in noncoding, intron, intergenic, and coding regions along with the samples. The *X*-axis means the seven *C. album* samples, and the *Y*-axis indicates the number of normal SSRs. Four different colors correspond to the four different regions. The table below the *X*-axis shows the number of SSRs along with samples and regions. (c) Shows SSR density (# of SSRs/kb) of the LSC, SSC, and IR regions along with the samples. The *X*-axis means the seven *C. album* samples, and the *Y*-axis indicates the number of normal SSRs along with samples and the three regions. Three different colors in the bar graphs mean the three regions. The table below the *X*-axis shows SSR density along with samples and the three regions.

In the seven *C. album* chloroplast genomes, 376 normal SSRs are identified (Table 5; Supplementary Tables 1–7). In addition, we also identified 280 extended SSRs and 3,039 potential SSRs on the seven chloroplast genomes (see Materials and Methods; Supplementary Tables 1–7). We analyzed only normal SSRs hereafter because normal SSRs can be commonly recognized as SSRs in various studies (see Materials and Methods). The unit length of normal SSRs varies from 1 bp (monoSSR) to 5 bp (pentaSSR), and the numbers of normal SSRs in each chloroplast genome are from 53 to 55, displaying an almost similar manner: CAGAP004 contains 55 normal SSRs, the largest, and CAJEJG05, CCANG01, and CSJUK01 contains 54 normal SSRs, while CAGOH01, NC_034950, and CVHUP01 have 53 normal SSRs (Table 5). Interestingly, no hexaSSR is identified on the seven *C. album* chloroplast genomes. The majority of normal SSRs is monoSSR (60.6%), and pentaSSR (3.70%) is the least (Figure 4(a)). In monoSSR, only an A/T motif was detected in all seven chloroplast genomes.

The overall distribution of normal SSRs on the seven *C. album* chloroplast genomes is similar to each other (Figure 4(b)). The intergenic region displays the largest number of normal SSRs, then coding, intron, and noncoding regions are in order (see Materials and Methods; Figure 4(b)). In all seven *C. album* chloroplast genomes, four normal SSRs are found in the noncoding region of *rrn23*, and 11 normal SSRs are identified in the coding regions of *rpoC2*, *rpoB*, *atpB*, *rpoA*, *ycf1*, and *ndhB*. In total, 30 to 33 normal SSRs were found in the intergenic regions on the seven chloroplast genomes. Most of the normal SSRs in the intergenic region are shared among the seven chloroplast genomes; however, CAJEJG05 and CAGAP004 chloroplast genomes have two distinct normal SSRs in their intergenic region of *trnR-trnN* and *rpl32-trnL* caused by one-bp INDEL and one SNP changing “T” to “A,” respectively. Also, CCANG01 and CSJUK01 chloroplast genomes have additional monoSSR between *ndhC* and *rbcL* by insertion of 1 bp nucleotide “T.” In contrast, the deletion of a single nucleotide of “A” between *ndhF* and *rpl32* caused the removal of intergenic SSRs in CAGOH01, CCANG01, and CSJUK01 chloroplast genomes. All seven *C. album* chloroplast genomes have seven common normal SSRs located in the intronic regions of five PCGs, *rps16*, *atpF*, *ycf3*, *rpl16*, and *ndhA.* In the case of CAGOH01 and CCANG01 chloroplast genomes, one additional monoSSR is identified in the intronic region of *trnK* because one SNP changing “A” to “T” occurred in both chloroplast genomes. Besides, CSJUK01 has an extra unique tetraSSR “GTTT” in the intronic regions of *ycf3* by 4 bp insertion. These differences of normal SSRs among the seven chloroplasts of *C. album* can be utilized as molecular markers to distinguish their origins inside the Korean Peninsula once more chloroplast genomes of *C. album* in Korea are available.

Due to the different lengths of the LSC and SSC regions, the density of SSRs per Kbp was calculated. Interestingly, three chloroplast genomes, CAGOH01, CCANG01, and CSJUK01, display similar density in both the LSC and SSC regions, while the remaining four chloroplast genomes present that the density in the SSC region is larger than that of the LSC region. Since in the SSC region of CAGAP004, CAJEJG05, CVHUP01, and NC_034950 there is an additional intergenic normal SSR located in between *ndhF* and *rpl32* (Figure 4(c)). The densities of normal SSRs in IR regions of all seven chloroplast genomes are lowest (0.159 to 0.198 normal SSRs/Kbp; Figure 4(c)).

To understand conserved normal SSRs across the seven chloroplast genomes, we calculated SSR groups which contain normal SSRs of which left and right flanking sequences are similar to each other (see Materials and Methods). In total, 58 SSR groups and two singleton SSRs were identified, and 50 of 58 SSR groups (86.2%) contain seven normal SSRs originating from all seven chloroplast genomes, called the common SSR group. Eleven out of the 50 common SSR groups (22.0%) are located in the coding region, and 36 common SSR groups (72.0%) are in the intergenic region (Figure 4(b)), which is congruent to the analysis result of normal SSRs mentioned in the previous section. Five intergenic loci contain two common SSR groups, *rpl32-trnL*, *atpH-atpI*, *ycf3-trnS*, *trnQ-psbK*, and *trnK-rps16* in each, and 31 intergenic loci contain one common SSR group. Two singleton SSRs were found in CSJUK01 and CCANG01 chloroplast genomes. These intraspecific variations of normal SSRs will provide insights into changes of SSRs inside the species, which can also be utilized to develop molecular markers of *C. album* efficiently.

### 2.4. Phylogenetic Analysis of Korean *C. album* Chloroplast Genome Sequence

**Figure 5:**
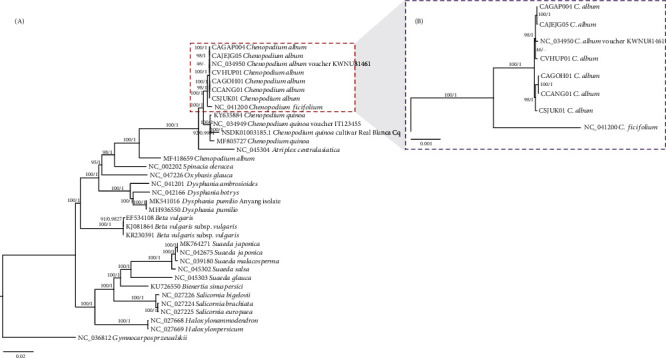
Phylogenetic trees of 34 Amaranthaceae chloroplast genomes. (a) Maximum likelihood (ML) and Bayesian inference (BI) phylogenetic trees were constructed based on complete chloroplast genomes of 34 Amaranthaceae species and *Gymnocarpos przewalskii* as an outgroup species. The phylogenetic tree was drawn based on the ML tree. Numbers on branches in the phylogenetic tree indicate bootstrap values of ML and prior possibility of BI, respectively. The dotted red rectangle indicates the clade covering *C. album* and *C. ficifolium*. Chloroplast genomes sequenced in this study were presented as bold characters. (b) Partially enlarged phylogenetic tree of the clade of *C. album* and *C. ficifolium* was displayed. Numbers on branches in the phylogenetic tree indicate bootstrap values of ML and prior possibility of BI, respectively. Chloroplast genomes sequenced in this study were presented as bold characters.

Bootstrapped maximum likelihood (ML) and Bayesian inference (BI) phylogenetic trees of 34 Amaranthaceae chloroplast genomes including the six *C. album* chloroplasts sequenced in this study and one outgroup species, *Gymnocarpos przewalskii*, were constructed (see Materials and Methods). Phylogenetic trees present that six *C. album* chloroplast genomes are clustered with the previously sequenced *C. album* chloroplast genome (NC_034950) with high supportive values of ML and BI except the node containing NC_034950 in the ML tree (Figure 5(a)). The *C. album* s. str. clade is divided reciprocally into two clades in both trees. CAGAP004 and CAJEJG05 sharing the morphological feature of narrow leaves and collected in Jejudo island (Figure 1) only exhibit a correlation of geographical locations with high supportive values of ML and BI (Figure 5(b)). Leaf shape and margin of the six samples are not correlated to the two clades of *C. album* (Table 1 and Figure 5(b)); however, leaf thickness of the six *C. album* presents correlation to the clades: the clade containing CAGAP004, CAJEG05, and CVHUP01 shows thick leaves, called as a thick-leaf clade, and the clade consisting of CAGOH01, CCANG01, and CSJUK01 displays thin leaves, called as a thin-leaf clade (Table 1 and Figure 5(b)). NC_034950 clustered in the thick-leaf clade was not possible to be confirmed whether its leaves are thick or not. Taken together, the phylogenetic relationship of the six *C. album* chloroplast genomes seems not to be highly correlated with their morphological features and geographical locations, supporting that their high plasticity of morphology links to other factors such as nuclear markers, polyploidy, or any regulatory factor of leaf morphologies. With the additional chloroplast genomes as well as nuclear marker sequences of *C. album* collected in Korea, these relationships of morphological features and geographical locations will be more explicit.

## 3. Discussions

### 3.1. Species Incongruency of *C. album* Chloroplast Genomes

In this study, we sequenced six chloroplast genomes of *C. album* s. str. collected in Korea displaying various morphological features. One of the previously sequenced chloroplast genomes of *C. album*, MF418659, is quite different from the remaining *C. album* chloroplast genomes in the aspects of gene configuration of chloroplast genome (Table 3) as well as phylogenetic relationship (MF418659 chloroplast genome was located outside of the clade of *Chenopodium* and *Atriplex* in Figure 5). These differences indicate that MF418659 may neither be *C. album* nor genus *Chenopodium*.

It is partially supported by the fact that the collection site of MF418659 is the Himalayan area in India [24] where *C. album* has been mainly cultivated as crops [7–9]. Usually, species diversity of the Himalayan area is higher due to its wide variety of climates as well as various climatic perturbations that have been applied to different locations in the Himalayan area [29]. Considering the phylogenetic position of MF418659 (Figure 5), it is possible that MF418659 is misidentified or an unreported species which is very different from *C. album*.

This kind of incongruency problem of species has sometimes been found during comparative analyses in plant species. For instance, two *Magnolia* chloroplast genomes, *Magnolia insignis* (NC_035657) and *Magnolia alba* (NC_037005), were reported as examples of misidentification species based on the phylogenetic analysis based on complete chloroplast genomes [30]. This problem can occur due to not enough taxonomic coverage of whole chloroplast genomes or misidentification of the samples used in the studies because of difficulties in species identification based on morphologies. Therefore, the identification of MF418659 should be revised in some ways, such as species identification of the voucher used in the previous study or sequencing and analyzing more samples of *C. album* collected in the Himalayan area.

### 3.2. Possible Causes of *C. album* s. str. Morphological Variations at the Molecular Level

Based on cytogenic and nuclear molecular marker analysis of *Chenopodium* species, *C. album* is distinct to *C. ficifolium* (B genome diploid) and *C. quinoa* (A genome tetraploid) [14], and two major groups of *C. album* were identified based on the phylogenetic tree based on *rrn5* and ITS sequences [14]. In comparison to the phylogenetic tree which displays that *C. album* and *C. ficifolium* were clustered in one clade with high supportive values (Figure 5), a maternal lineage of both species is nearer than that of biparental lineage. Several *C. quinoa* chloroplast genomes were clustered in the distinct clade to that of *C. album* and *C. ficifolium* (Figure 5), reflecting the different types of their genomes [14]. This phylogenetic tree based on complete chloroplast genomes (Figure 5) also indirectly supports that various intraspecific evolutionary events in several *Chenopodium* species, including *C. album* and *C. quinoa*, may have occurred, such as hybridization and polyploidization [2, 14].

Polyploidization and hybridization events can usually cause morphological plasticity and diversity: e.g., *Nicotiana* species display various flower colors based on events of polyploidization [31], and *Centaurea stoebe*, polyploidy species, shows that it causes various phenotypes to climate, resulting in boosting its invasion [32]. Similarly, morphological variations of *C. album* are not related to maternal lineage (Figures 1 and 5). It can be inferred that *C. album* presents various morphological differences because it is hexaploidy species. It can also be interpreted that these morphological variations are not fully genetically fixed but may be caused by nuclear genes related to leaf development, such as Class I *KNOX* genes, homeobox transcription factors which can regulate leaf shapes in *Arabidopsis thaliana* [33]. In this study, we found that the leaf morphology of six *C. album* has a weak correlation with their phylogenetic relation (Figures 1 and 5). If diverse leaf shapes of *C. album* are caused by these key regulators, we can deduce that the general trend of evolutionary process inferred from organelle genomes including chloroplast cannot explain this diversity because these regulators can display different evolutionary speeds and patterns from those of organelle genomes.

Several studies tried to delimitate species presenting different morphological features using whole chloroplast genome sequences. For instance, a phylogenetic tree constructed based on chloroplast genomes of *Anemopaegma acutifolium* supported that two leaf morphological trait types of *A. acutifolium* were caused by different maternal origins [34]. In the case of *Triplostegia glandulifera* and *T. grandiflora*, their chloroplast genomes were used for solving the boundary of the two species; however, they could not explain the high morphological plasticity of them [35]. Therefore, further analyses with more chloroplast genomes of *C. album* expressing various phenotypic characteristics will be necessary to understand the origin of its morphological plasticity.

### 3.3. Evaluation of Level of Intraspecific Variations on *C. album* Chloroplast Genomes

The intraspecific variations identified among the seven *Chenopodium album* chloroplast genomes (56 SNPs and 26 INDEL regions) are compared with the previous studies which investigated intraspecific variations on chloroplast genomes. Twenty cultivars and wild types of *Ricinus communis* (Castor bean) displayed 162 SNPs and 92 INDEL regions [28], which is three times more than those of *C. album*. Sixty-three chloroplast genomes of *Macadamia integrifolia* (Macadamia nut) are collected in eastern Australia, which is a smaller geographical range of *C. album*. Four hundred and seven SNPs [36] are detected from them, which is seven times more than the number of SNPs identified in this study. Comparing with that of our study, the numbers of intraspecific variations identified from *C. album* chloroplast genome are relatively lower. In the case of *Dioscorea polystachya* (Chinese yam), six chloroplast genomes collected in Northern and Southern China displayed 141 SNPs and 44 INDEL regions [37]. Its geographical coverage is larger than that of *C. album*, and climates of the six regions are quite different than those of *C. album*; the larger number of intraspecific variations in *D. polystachya* is reasonable.

To evaluate intraspecific variations on *C. album* chloroplast genomes considering its geographical distribution, various studies which identified intraspecific variations of organelle genomes from the plant species collected in Korea were surveyed. To compare intraspecific variations between two samples in the same species, we conducted a pairwise comparison of *C. album* chloroplast genomes, resulting in 0 to 33 SNPs and 7 to 36 INDEL regions being identified from the seven *C. album* chloroplast genomes. In the case of *Suaeda japonica* collected in Korea with different morphological features, only three SNPs and three INDEL regions were identified [38], which is mostly smaller than those identified in *C. album*. The number of intraspecific variations identified from *Dysphania pumilio*, another Amaranthaceae species, is 24 SNPs and one INDEL region [39], which is also in the range of those of *C. album*. Based on these previous results, intraspecific variations identified from the seven *C. album* chloroplast genomes are similar to those of Amaranthaceae species.

## 4. Materials and Methods

### 4.1. DNA Extraction of Natural Collection of Korean *C. album*

Six samples of *C. album* were collected in various places in the Korean Peninsula (Table 1 and Figure 1). All vouchers of the six samples were deposited to the Sung Kyun Kwan University Herbarium (SKKU; Table 1). Their total DNA was extracted from fresh leaves of the six samples using a DNeasy Plant Mini Kit (QIAGEN, Hilden, Germany).

### 4.2. Genome Sequencing and *De Novo* Assembly of the Natural Collection of Six *C. album* Chloroplast Genomes

Genome sequencing was performed using HiSeqX at Macrogen Inc., Korea, from the extracted DNA of the six *C. album*. *De novo* assembly with confirmation was accomplished with Velvet v1.2.10 [40] after filtering raw reads using Trimmomatic v0.33 [41]. After obtaining the first draft of the chloroplast genome sequences, gaps were filled with GapCloser v1.12 [42], and all bases from the assembled sequences were confirmed by checking each base in the alignment (tview mode in SAMtools v1.9 [43]) against the assembled chloroplast genome generated with BWA v0.7.17 [44]. All these processes were conducted under the environment of the Genome Information System (GeIS; http://geis.infoboss.co.kr/; Park et al., in preparation) like other Amaranthaceae chloroplast genomes assembled [38, 39, 45–49].

### 4.3. Chloroplast Genome Annotation

Geneious Prime® 2020.2.4 (Biomatters Ltd, Auckland, New Zealand) was used for chloroplast genome annotation based on the *C. album* chloroplast genome (NC_034950) [23] by transferring annotations while correcting exceptional cases, including missing start or stop codons. tRNA was predicted and confirmed based on the prediction of tRNAScan-SE v2.0.6 [50]. A circular map of *C. album* chloroplast was drawn by using the OGDRAW v1.3.1 [51].

### 4.4. Identification of Sequence Variations on the Complete Chloroplast Genomes of *C. album*

Single nucleotide polymorphisms (SNPs) and insertions and deletions (INDELs) were identified from the pair-wise alignments of two selected chloroplast genomes conducted by MAFFT v7.450 [52]. When the number of INDELs was calculated, continuous INDEL bases were considered one INDEL. In addition, we denote the four regions: (i) coding region is exon that encodes a protein, (ii) intron regions indicate the region which does not translate inside protein-coding genes, (iii) intergenic regions are the sequence between two genes, and (iv) noncoding region means the sequence located in tRNAs or rRNAs.

### 4.5. Identification of Simple Sequence Repeats (SSRs)

Simple sequence repeats (SSRs) were identified on the chloroplast genome sequence using the pipeline of the SSR database (SSRDB; http://ssrdb.infoboss.co.kr/; Park et al., in preparation). Based on the conventional definition of an SSR on the chloroplast genome, monoSSR (1 bp) to hexaSSR (6 bp), the total length of SSRs on the chloroplast genome exceeds 10 bp. Owing to the different criteria of SSRs on chloroplast genomes, we adopted the criteria used in chloroplast genomes of *Dysphania* [47] and *Arabidopsis thaliana* [53] and mitochondrial genome of *Rosa rugosa* [54] as follows: the monoSSR (unit sequence length of 1 bp) to hexaSSR (6 bp) are used as normal SSRs, and heptaSSR (7 bp) to decaSSR (10 bp) are defined as extended SSRs. Among the normal SSRs, pentaSSRs and hexaSSRs for which the repeat number of unit sequences is 2 are classified as potential SSRs. Classification of regions on chloroplast genome was conducted in the same way described in the above section.

### 4.6. Comparison of SSRs Identified from Seven *C. album* Chloroplast Genomes

SSRs identified from seven *C. album* chloroplast genomes were compared based on their flanking sequences under the environment of the SSRDB (http://ssrdb.infoboss.co.kr/; Park et al., in preparation). The pipeline of the SSR comparison implemented in the SSRDB used in various organelle genome studies [53, 55] was used with the following conditions: a cut-off *e* value of 1*e* − 10 and a maximum flanking sequence for the comparison of 60 bp.

### 4.7. Nucleotide Diversity Analysis

Nucleotide diversity was calculated using the method proposed by Nei and Li [56] based on the multiple sequence alignment of *Chenopodium* chloroplast genomes using a Perl script used in previous studies [47, 53, 57]. The window size was set to 500 bp, and the step size was 200 bp when using the sliding-window method. Genomic coordination of each window was compared to the gene annotation of the chloroplast genome under the GenomeArchive® (http://www.genomearchive.net/) [58] environment for further analyses.

### 4.8. Construction of Phylogenetic Trees

The whole 34 Amaranthaceae chloroplast genomes and one outgroup of *Gymnocarpos przewalskii* chloroplast genome were aligned by MAFFT v7.450 [52], and alignment quality was checked manually. The maximum likelihood (ML) tree was reconstructed in IQ-TREE v1.6.6 [59]. In the ML analysis, a heuristic search was used with nearest-neighbor interchange (NNI) branch swapping, TVM+F+R4 model, and uniform rates among sites. All other options used the default settings. Bootstrap analyses with 1,000 pseudoreplicates were conducted with the same options. The posterior probability of each node was estimated by Bayesian inference (BI) using the MrBayes v3.2.7a [60] plug-in implemented in Geneious Prime® 2020.2.4 (Biomatters Ltd, Auckland, New Zealand). The HKY85 model with gamma rates was used as a molecular model. A Markov chain Monte Carlo (MCMC) algorithm was employed for 1,100,000 generations, sampling trees every 200 generations, with four chains running simultaneously. Trees from the first 100,000 generations were discarded as burn-in.

## 5. Conclusions

We completed the six chloroplast genomes of *Chenopodium album* showing various morphological features. The structure and gene order of chloroplast are conserved among seven *C. album* including the previously sequenced chloroplast genome (NC_034950). The average nucleotide diversity calculated from the seven *C. album* chloroplast genomes is 0.0000625, and a total of 56 SNPs and 26 INDEL regions are found. In comparison to the other cases of chloroplast intraspecific variations, *C. album* chloroplasts present a low level of sequence variation. The number of normal SSR identified from the seven *C. album* chloroplast genomes ranges from 33 to 35 displaying similar distribution and density of SSRs. Interestingly, specific SNPs and INDEL regions in intronic and intergenic regions make SSR variation among the seven chloroplasts. All seven *C. album* chloroplast genomes are clustered in high supportive values of ML and BI trees with a short length of branches. In addition, one of the morphological characters of *C. album* s. str., the thickness of leaves, presented correlation with the phylogenetic position. Taking together the results in this study, our six chloroplast genomes of *C. album* s. str. will provide the way to investigate intraspecific features of chloroplast genomes, also the insights of intraspecific variations to understand various characteristics of one species including morphological features.

## Figures and Tables

**Table 1:** List of six *Chenopodium album* samples used in this study.

Sample name	Voucher number^∗^	Morphological features			GPS coordinates
		Leaf shape	Leaf margin	Leaf thickness	
CAGAP004	KYS130730	Lanceolate	Serrate	Thick	33°43′25.76^″^N 126°92′45.26^″^E
CAGOH01	sgu180626	Ovate	Serrate	Thin	34°29′30.09^″^N 127°21′19.84^″^E
CAJEJG05	sgu180521	Lance-ovate	Entire	Thick	33°10′16.00^″^N 126°15′57.73^″^E
CCANG01	sgu180601	Widely ovate	Serrate	Thin	37°35′35.75^″^N 126°30′53.71^″^E
CSJUK01	sgu180926	Lanceolate	Entire	Thin	37°58′19.00^″^N 128°45′51.00^″^E
CVHUP01	sgu180918	Lanceolate	Entire	Thick	35°32′54.26^″^N 126°40′27.30^″^E

^∗^All vouchers were deposited in Sung Kyun Kwan University Herbarium (SKKU) in Korea.

**Table 2:** List of six chloroplast genomes of *Chenopodium album* sequenced in this study.

Strain name	GenBank accession	Length (bp)				GC contents			
		Whole	LSC	SSC	IR	Whole	LSC	SSC	IR
CCANG01	MW446241	151,906	83,681	17,969	25,128	37.3%	35.3%	31.0%	42.7%
CSJUK01	MW446242	152,197	83,679	18,132	25,193	37.2%	35.3%	31.0%	42.7%
CAJEJG05	MW446243	152,183	83,680	18,130	25,194	37.2%	35.3%	31.0%	42.7%
CVHUP01	MW446244	152,190	83,832	18,132	25,113	37.2%	35.3%	31.0%	42.8%
CAGOH01	MW446245	152,196	83,679	18,131	25,193	37.3%	35.3%	31.0%	42.7%
CAGAP004	MW446246	152,199	83,681	18,130	25,194	37.2%	35.3%	31.0%	42.7%

**Table 3:** List of chloroplast genomes used for comparative analyses in this study.

Family	Species name	NCBI accession	Total length (bp)	# of PGCs	# of tRNAs	# of rRNAs	GC ratio (%)	Reference
Chenopodioideae	*Chenopodium album*	MW446241	151,906	84	37	8	37.3%	This study
	*Chenopodium album*	MW446242	152,197	84	37	8	37.2%	This study
	*Chenopodium album*	MW446243	152,183	84	37	8	37.2%	This study
	*Chenopodium album*	MW446244	152,190	84	37	8	37.2%	This study
	*Chenopodium album*	MW446245	152,196	84	37	8	37.3%	This study
	*Chenopodium album*	MW446246	152,199	84	37	8	37.2%	This study
	*Chenopodium album*	NC_034950	152,167	84	37	8	37.2%	[23]
	*Chenopodium album* ^∗^	MF418659	150,272	89 (88)^∗∗^	33	8	37.0%	[24]
	*Chenopodium ficifolium*	NC_041200	151,923	84	37	8	37.3%	[45]
	*Chenopodium quinoa*	KY635884	152,075	84 (83)^∗∗^	36	8	37.2%	[61]
	*Chenopodium quinoa*	MF805727	151,069	84 (80)^∗∗^	29	8	37.2%	[62]
	*Chenopodium quinoa*	NC_034949	152,099	84	36	8	37.2%	[23]
	*Chenopodium quinoa* cultivar Real Blanca	NSDK01003185.1	152,282	N/A	N/A	N/A	37.2%	[63]
	*Atriplex centralasiatica*	NC_045304	152,237	85	37	8	37.3%	[64]
	*Dysphania ambrosioides*	NC_041201	151,689	84	36	8	36.9%	[65]
	*Dysphania botrys*	NC_042166	152,055	83	37	8	36.8%	[66]
	*Dysphania pumilio*	MK541016	151,960	84	36	8	36.9%	[39]
	*Dysphania pumilio*	NC_041159	151,962	84	36	8	36.9%	[67]
	*Oxybasis glauca*	NC_047226	151,655	84	37	8	36.9%	[49]
	*Spinacia oleracea*	NC_002202	150,725	96	37	8	36.8%	[68]

Salicornioideae	*Salicornia europaea*	NC_027225	153,232	84	37	8	36.2%	Unpublished
	*Salicornia bigelovii*	NC_027226	153,076	83	37	8	36.3%	Unpublished
	*Salicornia brachiata*	NC_027224	153,324	84	37	8	36.2%	Unpublished

Suaedoideae	*Suaeda japonica*	NC_042675	152,109	83	37	8	36.4%	[46]
	*Suaeda japonica*	MK764271	152,112	80	37	8	36.4%	[38]
	*Suaeda salsa*	NC_045302	151,642	85	37	8	36.4%	[69]
	*Suaeda glauca*	NC_045303	149,807	85	37	8	36.5%	[70]
	*Suaeda malacosperma*	NC_039180	151,989	83	37	8	36.4%	[71]
	*Bienertia sinuspersici*	KU726550	153,472	86	36	8	37.8%	[72]

Salsoloideae	*Haloxylon ammodendron*	NC_027668	151,570	85	37	8	36.6%	[73]
	*Haloxylon persicum*	NC_027669	151,586	85	37	8	36.6%	[73]

Betoideae	*Beta vulgaris*	EF534108	149,696	N/A	N/A	N/A	35.4%	Unpublished
	*Beta vulgaris* subsp. *vulgaris*	KJ081864	149,635	85	37	8	37.0%	[74]
	*Beta vulgaris* subsp*. vulgaris*	KR230391	149,722	81	29	8	37.0%	[75]

Paronychieae	*Gymnocarpos przewalskii*	NC_036812	150,636	81	37	8	36.5%	[66]

∗ indicates that the species name should be reconsidered. ^∗∗^Numbers in parenthesis are the original number of PCGs based on the annotation, and numbers outside of parenthesis indicate the number of PCGs based on our reannotation results.

**Table 4:** List of genes encoded by *Chenopodium album* chloroplast genomes.

Category	Group of gene	Name of gene
Self-replication	Ribosomal RNAs	*rrn4.5*, *rrn5*, *rrn16*, *rrn23*
	Transfer RNA genes	*trnA-UGC*, *trnC-GCA*, *trnD-GUC*, *trnE-UUC*, *trnF-GAA*, *trnG-GCC*, *trnH-GUG*, *trnI-CAU*, *trnI-GAU*, *trnK-UUU*, *trnL-CAA*, *trnL-UAA*, *trnL-UAG*, *trnM-CAU*, *trnN-GUU*, *trnP-UGG*, *trnQ-UUG*, *trnR-ACG*, *trnR-UCU*, *trnS-GCU*, *trnS-GGA*, *trnS-UGA*, *trnT-GGU*, *trnT-UGU*, *trnV-GAC*, *trnV-UAC*, *trnW-CCA*, *trnY-GUA*
	Small subunit of ribosome	*rps2*, *rps3*, *rps4*, *rps7*, *rps8*, *rps11*, *rps12*, *rps14*, *rps15*, *rps16*, *rps18*, *rps19*
	Large subunit of ribosome	*rpl2*, *rpl14*, *rpl16*, *rpl20*, *rpl22*, *rpl32*, *rpl33*, *rpl36*
	RNA polymerase	*rpoA*, *rpoB*, *rpoC1*, *rpoC2*
	Translation initiation factor	*infA*

Photosynthesis	ATP synthase	*atpA*, *atpB*, *atpE*, *atpF*, *atpH*, *atpI*
	NADH dehydrogenase subunit	*ndhA*, *ndhB*, *ndhC*, *ndhD*, *ndhE*, *ndhF*, *ndhG*, *ndhH*, *ndhI*, *ndhJ*, *ndhk*
	Cytochrome b/f complex subunit	*petA*, *petB*, *petD*, *petG*, *petL*, *petN*
	Photosystem subunit I subunit	*psaA*, *psaB*, *psaC*, *psaI*, *psaJ*
	Photosystem subunit II subunit	*psbA*, *psbB*, *psbC*, *psbD*, *psbE*, *psbF*, *psbH*, *psbI*, *psbJ*, *psbK*, *psbL*, *psbM*, *psbN*, *psbT*, *psbZ*
	Rubisco large subunit	*rbcL*

Other genes	Maturase	*matK*
	Protease	*clpP*
	Envelope membrane protein	*cemA*
	Subunit of acetyl-CoA-carboxylase	*accD*
	Cytochrome c-type biogenesis protein	*ccsA*

Genes of unknown function	Hypothetical reading frame	*ycf1*, *ycf2*, *ycf3*, *ycf4*

**Table 5:** List of normal SSRs of six *Chenopodium album*.

SSR type	CAGAP004	CAGOH01	CAJEJG05	CCANG01	CSJUK01	CVHUP01	NC034950
MonoSSR	34	32	33	33	32	32	32
DiSSR	9	9	9	9	9	9	9
TriSSR	2	2	2	2	2	2	2
TetraSSR	9	9	9	9	10	9	9
PentaSSR	1	1	1	1	1	1	1

## Data Availability

Chloroplast genome sequences of *C. album* sequenced in this study can be accessed via accession numbers MW446241 to MW446246 in NCBI GenBank.

## Supplementary Materials

Supplementary Materials Supplementary Table 1: list of SSRs identified in CAGAP004 of C. album. Supplementary Table 2: list of SSRs identified in CAGOH01 of C. album. Supplementary Table 3: list of SSRs identified in CAJEJG05 of C. album. Supplementary Table 4: list of SSRs identified in CCANG01 of C. album. Supplementary Table 5: list of SSRs identified in CSJUK01 of C. album. Supplementary Table 6: list of SSRs identified in CVHUP01 of C. album. Supplementary Table 7: list of SSRs identified in NC_034950 of C. album.

## References

[1] Habibi F., Vít P., Rahiminejad M., Mandák B. (2018). Towards a better understanding of the *Chenopodium album* aggregate (Amaranthaceae) in the Middle East: a karyological, cytometric and morphometric investigation. *Journal of Systematics and Evolution*.

[2] Mandák B., Krak K., Vít P. (2018). Hybridization and polyploidization within the _*Chenopodium album*_ aggregate analysed by means of cytological and molecular markers. *Molecular Phylogenetics and Evolution*.

[3] Mandák B., Trávníček P., Paštová L., Kořínková D. (2012). Is hybridization involved in the evolution of the _*Chenopodium album*_ aggregate? An analysis based on chromosome counts and genome size estimation. *Flora-Morphology, Distribution, Functional Ecology of Plants*.

[4] Mandák B., Krak K., Vít P. (2016). How genome size variation is linked with evolution within _*Chenopodium*_ sensu lato. *Perspectives in Plant Ecology, Evolution and Systematics*.

[5] Vít P., Krak K., Trávníček P., Douda J., Lomonosova M. N., Mandák B. (2016). Genome size stability across Eurasian *Chenopodiums* pecies (Amaranthaceae). *Botanical Journal of the Linnean Society*.

[6] Jellen E. N., Kolano B. A., Sederberg M. C., Bonifacio A., Maughan P. J. (2011). Chenopodium. *Wild Crop Relatives: Genomic and Breeding Resources*.

[7] Partap T., Kapoor P. (1985). The Himalayan grain chenopods. I. Distribution and ethnobotany. *Agriculture, Ecosystems & Environment*.

[8] Partap T., Kapoor P. (1987). The Himalayan grain chenopods. III. An under-exploited food plant with promising potential. *Agriculture, Ecosystems & Environment*.

[9] Łuczaj Ł., Szymański W. M. (2007). Wild vascular plants gathered for consumption in the Polish countryside: a review. *Journal of Ethnobiology and Ethnomedicine*.

[10] Partap T., Joshi B. D., Calwey N. (1998). *Chenopods: Chenopodium spp*.

[11] Wahl H. A. (1952). A preliminary study of the genus Chenopodium in North America. *Bartonia*.

[12] Chung Y. (1992). *A taxonomic study of the Korean Chenopodiaceae, [Ph.D. thesis]*.

[13] Krak K., Vít P., Belyayev A., Douda J., Hreusová L., Mandák B. (2016). Allopolyploid origin of *Chenopodium album* s. str.(Chenopodiaceae): a molecular and cytogenetic insight. *PLoS One*.

[14] Kolano B., McCann J., Oskędra M. (2019). Parental origin and genome evolution of several Eurasian hexaploid species of Chenopodium (Chenopodiaceae). *Phytotaxa*.

[15] Goodwin S., McPherson J. D., McCombie W. R. (2016). Coming of age: ten years of next-generation sequencing technologies. *Nature Reviews Genetics*.

[16] Roberts R. J., Carneiro M. O., Schatz M. C. (2013). The advantages of SMRT sequencing. *Genome Biology*.

[17] Deamer D., Akeson M., Branton D. (2016). Three decades of nanopore sequencing. *Nature Biotechnology*.

[18] Sun J., Wang Y., Liu Y. (2020). Evolutionary and phylogenetic aspects of the chloroplast genome of _Chaenomeles_ species. *Scientific Reports*.

[19] Alzahrani D. A., Yaradua S. S., Albokhari E. J., Abba A. (2020). Complete chloroplast genome sequence of *Barleria prionitis*, comparative chloroplast genomics and phylogenetic relationships among Acanthoideae. *BMC Genomics*.

[20] Liang H., Zhang Y., Deng J. (2020). The complete chloroplast genome sequences of 14 *Curcuma* species: insights into genome evolution and phylogenetic relationships within Zingiberales. *Frontiers in Genetics*.

[21] Wang H., Park S.-Y., Song S.-H. (2020). Analysis of complete chloroplast genome sequence of Korean landrace *Cymbidium goeringii*. *3 Biotech*.

[22] Li C., Zheng Y., Huang P. (2020). Molecular markers from the chloroplast genome of rose provide a complementary tool for variety discrimination and profiling. *Scientific Reports*.

[23] Hong S.-Y., Cheon K.-S., Yoo K.-O. (2017). Complete chloroplast genome sequences and comparative analysis of *Chenopodium quinoa* and *C. album*. *Frontiers in Plant Science*.

[24] Devi R. J., Thongam B. (2017). Complete chloroplast genome sequence of *Chenopodium album* from Northeastern India. *Genome Announcements*.

[25] Li J., Tang J., Zeng S., Han F., Yuan J., Yu J. (2020). Comparative plastid genomics of four Pilea (Urticaceae) species: insight into interspecific plastid genome diversity in Pilea. *BMC Plant Biology*.

[26] Su Q., Liu L., Zhao M. (2020). The complete chloroplast genomes of seventeenAegilops tauschii: genome comparative analysis and phylogenetic inference. *PeerJ*.

[27] Silva S. R., Pinheiro D. G., Penha H. A. (2019). Intraspecific variation within the *Utricularia amethystina* species morphotypes based on chloroplast genomes. *International Journal of Molecular Sciences*.

[28] Muraguri S., Xu W., Chapman M. (2020). Intraspecific variation within castor bean (*Ricinus communis* L.) based on chloroplast genomes. *Industrial Crops and Products*.

[29] Rana S. K., Price T. D., Qian H. (2019). Plant species richness across the Himalaya driven by evolutionary history and current climate. *Ecosphere*.

[30] Park S. H. (2020). *A phylogenomic study of Magnoliaceae and its evolutionary implications, [Ph.D. thesis].*.

[31] McCarthy E. W., Arnold S. E., Chittka L. (2015). The effect of polyploidy and hybridization on the evolution of floral colour in *Nicotiana* (Solanaceae). *Annals of Botany*.

[32] Hahn M. A., Van Kleunen M., Müller-Schärer H. (2012). Increased phenotypic plasticity to climate may have boosted the invasion success of polyploid *Centaurea stoebe*. *PLoS One*.

[33] Hay A., Tsiantis M. (2010). KNOX genes: versatile regulators of plant development and diversity. *Development*.

[34] Firetti F., Zuntini A. R., Gaiarsa J. W., Oliveira R. S., Lohmann L. G., Van Sluys M. A. (2017). Complete chloroplast genome sequences contribute to plant species delimitation: a case study of the *Anemopaegma* species complex. *American Journal of Botany*.

[35] Niu Y.-T., Jabbour F., Barrett R. L. (2018). Combining complete chloroplast genome sequences with target loci data and morphology to resolve species limits in _*Triplostegia*_ (Caprifoliaceae). *Molecular Phylogenetics and Evolution*.

[36] Nock C. J., Hardner C. M., Montenegro J. D. (2019). Wild origins of macadamia domestication identified through intraspecific chloroplast genome sequencing. *Frontiers in Plant Science*.

[37] Cao J., Jiang D., Zhao Z. (2018). Development of chloroplast genomic resources in Chinese Yam (*Dioscorea polystachya*). *BioMed Research International*.

[38] Kim Y., Park J., Chung Y. (2020). The comparison of the complete chloroplast genome of *Suaeda japonica* Makino presenting different external morphology (Amaranthaceae). *Mitochondrial DNA Part B*.

[39] Park J., Kim Y. (2019). The second complete chloroplast genome of *Dysphania pumilio* (R.Br.) mosyakin & clemants (Amranthaceae): intraspecies variation of invasive weeds. *Mitochondrial DNA Part B*.

[40] Zerbino D. R., Birney E. (2008). Velvet: algorithms for de novo short read assembly using de Bruijn graphs. *Genome Research*.

[41] Bolger A. M., Lohse M., Usadel B. (2014). Trimmomatic: a flexible trimmer for Illumina sequence data. *Bioinformatics*.

[42] Zhao Q.-Y., Wang Y., Kong Y.-M., Luo D., Li X., Hao P. (2011). Optimizing de novo transcriptome assembly from short-read RNA-Seq data: a comparative study. *BMC Bioinformatics*.

[43] Li H., Handsaker B., Wysoker A. (2009). The sequence alignment/map format and SAMtools. *Bioinformatics*.

[44] Li H. (2013). Aligning sequence reads, clone sequences and assembly contigs with BWA-MEM.

[45] Kim Y., Chung Y., Park J. (2019). The complete chloroplast genome of *Chenopodium ficifolium* Sm. (Amaranthaceae). *Mitochondrial DNA Part B*.

[46] Kim Y., Park J., Chung Y. (2019). The complete chloroplast genome of *Suaeda japonica* Makino (Amaranthaceae). *Mitochondrial DNA Part B*.

[47] Kim Y., Park J., Chung Y. (2019). Comparative analysis of chloroplast genome of *Dysphania ambrosioides* (L.) Mosyakin & Clemants understanding phylogenetic relationship in genus *Dysphania* R. Br.. *Korean Journal of Plant Resources.*.

[48] Kim Y., Chung Y., Park J. (2019). The complete chloroplast genome sequence of *Dysphania pumilio* (R.Br.) Mosyakin & Clemants (Amaranthaceae). *Mitochondrial DNA Part B*.

[49] Kim Y., Chung Y., Park J. (2020). The complete chloroplast genome of *Oxybasis glauca*(L.) S. Fuentes, Uotila & Borsch (Amaranthaceae) as the first chloroplast genome in genus *Oxybasis*. *Mitochondrial DNA Part B*.

[50] Lowe T. M., Chan P. P. (2016). tRNAscan-SE on-line: integrating search and context for analysis of transfer RNA genes. *Nucleic Acids Research*.

[51] Greiner S., Lehwark P., Bock R. (2019). OrganellarGenomeDRAW (OGDRAW) version 1.3. 1: expanded toolkit for the graphical visualization of organellar genomes. *Nucleic Acids Research*.

[52] Katoh K., Standley D. M. (2013). MAFFT multiple sequence alignment software version 7: improvements in performance and usability. *Molecular Biology and Evolution*.

[53] Park J., Xi H., Kim Y. (2020). The complete chloroplast genome of *Arabidopsis thaliana* isolated in Korea (Brassicaceae): an investigation of intraspecific variations of the chloroplast genome of Korean *A. thaliana*. *International Journal of Genomics*.

[54] Park J., Xi H., Kim Y., Nam S., Heo K.-I. (2020). The complete mitochondrial genome of new species candidate of *Rosa rugosa* (Rosaceae). *Mitochondrial DNA Part B*.

[55] Lee J., Park J., Xi H., Park J. (2020). Comprehensive analyses of the complete mitochondrial genome of *Figulus binodulus* (Coleoptera: Lucanidae). *Journal of Insect Science*.

[56] Nei M., Li W.-H. (1979). Mathematical model for studying genetic variation in terms of restriction endonucleases. *Proceedings of the National Academy of Sciences*.

[57] Park J., Xi H., Oh S.-H. (2020). Comparative chloroplast genomics and phylogenetic analysis of the *Viburnum dilatatum* complex (Adoxaceae) in Korea. *Korean Journal of Plant Taxonomy*.

[58] Park J., Xi H. Genome Archive (R): standardized genome repository for supporting large-scale genome analyses.

[59] Nguyen L.-T., Schmidt H. A., Von Haeseler A., Minh B. Q. (2015). IQ-TREE: a fast and effective stochastic algorithm for estimating maximum-likelihood phylogenies. *Molecular Biology and Evolution*.

[60] Huelsenbeck J. P., Ronquist F. (2001). MRBAYES: Bayesian inference of phylogenetic trees. *Bioinformatics*.

[61] Rabah S. O., Lee C., Hajrah N. H. (2017). Plastome sequencing of ten nonmodel crop species uncovers a large insertion of mitochondrial DNA in cashew. *Plant Genome*.

[62] Wang K., Li L., Li S. (2017). Characterization of the complete chloroplast genome ofChenopodium quinoaWilld. *Mitochondrial DNA Part B*.

[63] Zou C., Chen A., Xiao L. (2017). A high-quality genome assembly of quinoa provides insights into the molecular basis of salt bladder-based salinity tolerance and the exceptional nutritional value. *Cell Research*.

[64] Zhang X.-J., Wang N., Zhang L.-Y., Fan S.-J., Qu X.-J. (2019). Characterization of the complete plastome of *Atriplex centralasiatica* (Chenopodiaceae), an annual halophytic herb. *Mitochondrial DNA Part B*.

[65] Kim Y., Chung Y., Park J. (2019). The complete chloroplast genome sequence of Dysphania ambrosioides (L.) Mosyakin & Clemants (Chenopodiaceae/Amaranthaceae sensu APG), a medicinal plant and invasive species in Korea. *The Korean Journal Of Weed Science*.

[66] Yang Z., Zhang Y., Pan L., Fu C. (2018). Characterization of the complete chloroplast genome of Gymnocarpos przewalskii, an endangered species in China and Mongolia. *Conservation Genetics Resources*.

[67] Kim Y., Heo K.-I., Lee S., Park J. (2018). Complete chloroplast genome sequence of the *Pseudostellaria longipedicellata* S. Lee, K. Heo & SC Kim (Caryophyllaceae). *Mitochondrial DNA Part B*.

[68] Schmitz-Linneweber C., Maier R. M., Alcaraz J.-P., Cottet A., Herrmann R. G., Mache R. (2001). The plastid chromosome of spinach (Spinacia oleracea): complete nucleotide sequence and gene organization. *Plant Molecular Biology*.

[69] Qu X.-J., Li X.-T., Zhang L.-Y., Zhang X.-J., Fan S.-J. (2019). Characterization of the complete chloroplast genome of *Suaeda salsa* (Amaranthaceae/Chenopodiaceae), an annual succulent halophyte. *Mitochondrial DNA Part B*.

[70] Qu X.-J., Liu L.-K., Zhang L.-Y., Zhang X.-J., Fan S.-J. (2019). The complete chloroplast genome of an annual halophyte herb, *Suaeda glauca* (Amaranthaceae). *Mitochondrial DNA Part B*.

[71] Park J.-S., Choi I.-S., Lee D.-H., Choi B.-H. (2018). The complete plastid genome of *Suaeda malacosperma* (Amaranthaceae/Chenopodiaceae), a vulnerable halophyte in coastal regions of Korea and Japan. *Mitochondrial DNA Part B*.

[72] Kim B., Kim J., Park H., Park J. (2016). The complete chloroplast genome sequence of *Bienertia sinuspersici*. *Mitochondrial DNA Part B*.

[73] Dong W., Xu C., Li D. (2016). Comparative analysis of the complete chloroplast genome sequences in psammophytic *Haloxylon* species (Amaranthaceae). *PeerJ*.

[74] Li H., Cao H., Cai Y.-F., Wang J.-H., Qu S.-P., Huang X.-Q. (2014). *The complete chloroplast genome sequence of sugar beet (Beta vulgaris ssp. vulgaris)*.

[75] Stadermann K. B., Weisshaar B., Holtgräwe D. (2015). SMRT sequencing only *de novo* assembly of the sugar beet (*Beta vulgaris*) chloroplast genome. *BMC Bioinformatics*.

