# Supplementary material for: The Comparative Analyses of Six Complete Chloroplast Genomes of Morphologically Diverse Chenopodium album L. (Amaranthaceae) Collected in Korea
Source: Int J Genomics. 2021 Apr 27;2021:6643444. doi: 10.1155/2021/6643444 (PMC8096589; doi:10.1155/2021/6643444)
Supplement: Supplementary Materials — Supplementary Table 1: list of SSRs identified in CAGAP004 of C. album. Supplementary Table 2: list of SSRs identified in CAGOH01 of C. album. Supplementary Table 3: list of SSRs identified in CAJEJG05 of C. album. Supplementary Table 4: list of SSRs identified in CCANG01 of C. album. Supplementary Table 5: list of SSRs identified in CSJUK01 of C. album. Supplementary Table 6: list of SSRs identified in CVHUP01 of C. album. Supplementary Table 7: list of SSRs identified in NC_034950 of C. album. [file 6643444.f1.docx]

**Supplementary Table 1. List of SSRs identified in CAGAP004 of *C. album***

| **Name** | **SSR type** | **Type** | **Cooridnation** | | **Unit sequence** | **Repeat number** | **Genes** | **Annotation** |
| --- | --- | --- | --- | --- | --- | --- | --- | --- |
| cH0000001 | PotentialSSR | HexaSSR-Cp | 113 | 124 | GTAAAG | 2 |  |  |
| cH0000002 | PotentialSSR | HexaSSR-Cp | 1240 | 1251 | GCTTTC | 2 | psbA | YP_009380194.1 [psbA]\| |
| cP0000001 | PotentialSSR | PentaSSR-Cp | 1292 | 1301 | ATTTA | 2 |  |  |
| c70000001 | ExtendedSSR | 7SSR-Cp | 1454 | 1467 | AGAAAAT | 2 |  |  |
| c70000002 | ExtendedSSR | 7SSR-Cp | 1696 | 1709 | AGTAGAA | 2 | trnK-UUU | (Intron)trnK-UUU [trnK-UUU]\| |
| cH0000003 | PotentialSSR | HexaSSR-Cp | 1748 | 1759 | AATTTC | 2 | trnK-UUU | (Intron)trnK-UUU [trnK-UUU]\| |
| cH0000004 | PotentialSSR | HexaSSR-Cp | 2949 | 2960 | TTTTTC | 2 | matK,trnK-UUU | YP_009380195.1 [matK]\|(Intron)trnK-UUU [trnK-UUU]\| |
| cP0000002 | PotentialSSR | PentaSSR-Cp | 4093 | 4102 | TATGT | 2 |  |  |
| cP0000003 | PotentialSSR | PentaSSR-Cp | 4124 | 4133 | AATGG | 2 |  |  |
| cP0000004 | PotentialSSR | PentaSSR-Cp | 4202 | 4211 | CAGAT | 2 |  |  |
| cP0000005 | PotentialSSR | PentaSSR-Cp | 4355 | 4364 | CATTT | 2 |  |  |
| cH0000005 | PotentialSSR | HexaSSR-Cp | 4442 | 4453 | AATATT | 2 |  |  |
| cTe0000001 | SSR | TetraSSR-Cp | 4464 | 4475 | TTTA | 3 |  |  |
| cP0000006 | PotentialSSR | PentaSSR-Cp | 4487 | 4496 | ATTTA | 2 |  |  |
| cP0000007 | PotentialSSR | PentaSSR-Cp | 4572 | 4581 | ATATA | 2 |  |  |
| cT0000001 | SSR | TriSSR-Cp | 4613 | 4624 | ATA | 4 |  |  |
| cP0000008 | PotentialSSR | PentaSSR-Cp | 4970 | 4979 | TAGAT | 2 | rps16 | (Intron)YP_009380196.1 [rps16]\| |
| cP0000009 | PotentialSSR | PentaSSR-Cp | 5204 | 5213 | CATTT | 2 | rps16 | (Intron)YP_009380196.1 [rps16]\| |
| cH0000007 | PotentialSSR | HexaSSR-Cp | 5274 | 5285 | ATCCAA | 2 | rps16 | (Intron)YP_009380196.1 [rps16]\| |
| c70000004 | ExtendedSSR | 7SSR-Cp | 5294 | 5307 | ACAATTA | 2 | rps16 | (Intron)YP_009380196.1 [rps16]\| |
| cM0000001 | SSR | MonoSSR-Cp | 5341 | 5351 | T | 11 | rps16 | (Intron)YP_009380196.1 [rps16]\| |
| cP0000010 | PotentialSSR | PentaSSR-Cp | 5441 | 5450 | AGAAT | 2 | rps16 | (Intron)YP_009380196.1 [rps16]\| |
| cP0000011 | PotentialSSR | PentaSSR-Cp | 5997 | 6006 | AATGC | 2 |  |  |
| cP0000012 | SSR | PentaSSR-Cp | 6080 | 6094 | TTAAT | 3 |  |  |
| cP0000013 | PotentialSSR | PentaSSR-Cp | 6190 | 6199 | TATAT | 2 |  |  |
| cH0000009 | PotentialSSR | HexaSSR-Cp | 6467 | 6478 | GCTCTG | 2 |  |  |
| cP0000014 | PotentialSSR | PentaSSR-Cp | 6555 | 6564 | TTCTA | 2 |  |  |
| cP0000015 | PotentialSSR | PentaSSR-Cp | 6629 | 6638 | GATTC | 2 |  |  |
| cM0000002 | SSR | MonoSSR-Cp | 6787 | 6797 | A | 11 |  |  |
| cM0000003 | SSR | MonoSSR-Cp | 6853 | 6862 | A | 10 |  |  |
| c80000001 | ExtendedSSR | 8SSR-Cp | 7100 | 7115 | ATTCTAAT | 2 |  |  |
| cH0000010 | PotentialSSR | HexaSSR-Cp | 7345 | 7356 | TTACAA | 2 |  |  |
| cP0000016 | PotentialSSR | PentaSSR-Cp | 7398 | 7407 | TCAAA | 2 |  |  |
| cP0000017 | PotentialSSR | PentaSSR-Cp | 7608 | 7617 | TTCTT | 2 |  |  |
| cP0000018 | PotentialSSR | PentaSSR-Cp | 7640 | 7649 | ATTCT | 2 |  |  |
| cM0000004 | SSR | MonoSSR-Cp | 7732 | 7742 | A | 11 |  |  |
| cP0000019 | PotentialSSR | PentaSSR-Cp | 7898 | 7907 | GAAAA | 2 |  |  |
| cP0000020 | PotentialSSR | PentaSSR-Cp | 7988 | 7997 | ATATA | 2 |  |  |
| cH0000012 | PotentialSSR | HexaSSR-Cp | 8068 | 8079 | AGATAA | 2 |  |  |
| cP0000021 | PotentialSSR | PentaSSR-Cp | 8136 | 8145 | TAAAA | 2 |  |  |
| cP0000022 | PotentialSSR | PentaSSR-Cp | 8212 | 8221 | AAAGA | 2 |  |  |
| cP0000023 | PotentialSSR | PentaSSR-Cp | 8267 | 8276 | GGCCT | 2 |  |  |
| cH0000013 | PotentialSSR | HexaSSR-Cp | 8345 | 8356 | TTTGAT | 2 |  |  |
| cP0000024 | PotentialSSR | PentaSSR-Cp | 8861 | 8870 | AATTG | 2 | trnG-UCC | (Intron)trnG-UCC [trnG-UCC]\| |
| cP0000025 | PotentialSSR | PentaSSR-Cp | 9226 | 9235 | TTTTG | 2 | trnG-UCC | (Intron)trnG-UCC [trnG-UCC]\| |
| cP0000026 | PotentialSSR | PentaSSR-Cp | 9251 | 9260 | CTTAA | 2 | trnG-UCC | (Intron)trnG-UCC [trnG-UCC]\| |
| cP0000027 | PotentialSSR | PentaSSR-Cp | 9284 | 9293 | TTTTC | 2 | trnG-UCC | (Intron)trnG-UCC [trnG-UCC]\| |
| cP0000028 | PotentialSSR | PentaSSR-Cp | 9482 | 9491 | TCTCA | 2 |  |  |
| cP0000029 | PotentialSSR | PentaSSR-Cp | 9504 | 9513 | CAAAA | 2 |  |  |
| cM0000005 | SSR | MonoSSR-Cp | 9530 | 9540 | A | 11 |  |  |
| cH0000016 | PotentialSSR | HexaSSR-Cp | 10446 | 10457 | GCTTGT | 2 | atpA | YP_009380199.1 [atpA]\| |
| cH0000017 | PotentialSSR | HexaSSR-Cp | 10849 | 10860 | CGGGAG | 2 | atpA | YP_009380199.1 [atpA]\| |
| c80000002 | ExtendedSSR | 8SSR-Cp | 11970 | 11985 | AAAAATAG | 2 | atpF | (Intron)YP_009380200.1 [atpF]\| |
| c70000008 | ExtendedSSR | 7SSR-Cp | 12213 | 12226 | TCGGTAT | 2 | atpF | (Intron)YP_009380200.1 [atpF]\| |
| cT0000002 | SSR | TriSSR-Cp | 12319 | 12330 | ATT | 4 | atpF | (Intron)YP_009380200.1 [atpF]\| |
| cTe0000002 | SSR | TetraSSR-Cp | 12884 | 12895 | GGAA | 3 |  |  |
| cP0000030 | PotentialSSR | PentaSSR-Cp | 13032 | 13041 | AGAAA | 2 |  |  |
| cP0000031 | PotentialSSR | PentaSSR-Cp | 13057 | 13066 | TTTCT | 2 |  |  |
| cD0000001 | SSR | DiSSR-Cp | 13328 | 13337 | CA | 5 |  |  |
| cP0000032 | PotentialSSR | PentaSSR-Cp | 13819 | 13828 | AAATA | 2 |  |  |
| cD0000002 | SSR | DiSSR-Cp | 13886 | 13895 | AT | 5 |  |  |
| cH0000018 | PotentialSSR | HexaSSR-Cp | 14259 | 14270 | TAAAGC | 2 | atpI | YP_009380202.1 [atpI]\| |
| cM0000006 | SSR | MonoSSR-Cp | 14711 | 14720 | A | 10 |  |  |
| cH0000019 | PotentialSSR | HexaSSR-Cp | 14759 | 14770 | TTTAAT | 2 |  |  |
| cP0000033 | PotentialSSR | PentaSSR-Cp | 15629 | 15638 | ATTAA | 2 |  |  |
| c80000003 | ExtendedSSR | 8SSR-Cp | 15688 | 15703 | TCTACCGC | 2 |  |  |
| cP0000034 | PotentialSSR | PentaSSR-Cp | 15767 | 15776 | TTTAT | 2 |  |  |
| cP0000035 | PotentialSSR | PentaSSR-Cp | 15780 | 15789 | TAAAT | 2 |  |  |
| cP0000036 | PotentialSSR | PentaSSR-Cp | 16844 | 16853 | CAATT | 2 | rpoC2 | YP_009380204.1 [rpoC2]\| |
| cP0000037 | PotentialSSR | PentaSSR-Cp | 17659 | 17668 | CAAAA | 2 | rpoC2 | YP_009380204.1 [rpoC2]\| |
| cP0000038 | PotentialSSR | PentaSSR-Cp | 17779 | 17788 | TATCT | 2 | rpoC2 | YP_009380204.1 [rpoC2]\| |
| cM0000007 | SSR | MonoSSR-Cp | 17835 | 17847 | T | 13 | rpoC2 | YP_009380204.1 [rpoC2]\| |
| cP0000039 | PotentialSSR | PentaSSR-Cp | 18318 | 18327 | CGATT | 2 | rpoC2 | YP_009380204.1 [rpoC2]\| |
| cP0000040 | PotentialSSR | PentaSSR-Cp | 18347 | 18356 | ATCCT | 2 | rpoC2 | YP_009380204.1 [rpoC2]\| |
| cH0000020 | PotentialSSR | HexaSSR-Cp | 18460 | 18471 | TTGATC | 2 | rpoC2 | YP_009380204.1 [rpoC2]\| |
| cH0000021 | PotentialSSR | HexaSSR-Cp | 18832 | 18843 | ACGTGT | 2 | rpoC2 | YP_009380204.1 [rpoC2]\| |
| cP0000041 | PotentialSSR | PentaSSR-Cp | 19044 | 19053 | CATAA | 2 | rpoC2 | YP_009380204.1 [rpoC2]\| |
| cD0000003 | SSR | DiSSR-Cp | 19187 | 19196 | AT | 5 | rpoC2 | YP_009380204.1 [rpoC2]\| |
| cH0000022 | PotentialSSR | HexaSSR-Cp | 21526 | 21537 | CAAATC | 2 | rpoC1 | YP_009380205.1 [rpoC1]\| |
| cP0000042 | PotentialSSR | PentaSSR-Cp | 21640 | 21649 | GGATT | 2 | rpoC1 | YP_009380205.1 [rpoC1]\| |
| cP0000043 | PotentialSSR | PentaSSR-Cp | 21729 | 21738 | ATCCT | 2 | rpoC1 | (Intron)YP_009380205.1 [rpoC1]\| |
| cP0000044 | PotentialSSR | PentaSSR-Cp | 21923 | 21932 | TTCTT | 2 | rpoC1 | (Intron)YP_009380205.1 [rpoC1]\| |
| cP0000045 | PotentialSSR | PentaSSR-Cp | 21955 | 21964 | AATTA | 2 | rpoC1 | (Intron)YP_009380205.1 [rpoC1]\| |
| cP0000046 | PotentialSSR | PentaSSR-Cp | 22139 | 22148 | TTAAA | 2 | rpoC1 | (Intron)YP_009380205.1 [rpoC1]\| |
| cH0000023 | PotentialSSR | HexaSSR-Cp | 22244 | 22255 | ACAAAA | 2 | rpoC1 | (Intron)YP_009380205.1 [rpoC1]\| |
| cM0000008 | SSR | MonoSSR-Cp | 25512 | 25521 | T | 10 | rpoB | YP_009380206.1 [rpoB]\| |
| cH0000024 | PotentialSSR | HexaSSR-Cp | 25955 | 25966 | TCTTTT | 2 | rpoB | YP_009380206.1 [rpoB]\| |
| cP0000047 | PotentialSSR | PentaSSR-Cp | 26434 | 26443 | ACTTA | 2 |  |  |
| cP0000048 | PotentialSSR | PentaSSR-Cp | 26727 | 26736 | TTGTA | 2 |  |  |
| cH0000026 | PotentialSSR | HexaSSR-Cp | 26888 | 26899 | AGAAAA | 2 |  |  |
| cP0000049 | PotentialSSR | PentaSSR-Cp | 27104 | 27113 | GAATC | 2 |  |  |
| cP0000050 | PotentialSSR | PentaSSR-Cp | 27564 | 27573 | TTCCC | 2 |  |  |
| cP0000051 | PotentialSSR | PentaSSR-Cp | 27693 | 27702 | CTGAG | 2 |  |  |
| cH0000027 | PotentialSSR | HexaSSR-Cp | 27805 | 27816 | TTCATA | 2 |  |  |
| cP0000052 | PotentialSSR | PentaSSR-Cp | 27937 | 27946 | TTTAG | 2 |  |  |
| cH0000028 | PotentialSSR | HexaSSR-Cp | 27963 | 27974 | TATCAT | 2 |  |  |
| cP0000053 | PotentialSSR | PentaSSR-Cp | 28196 | 28205 | ATTTC | 2 |  |  |
| cP0000054 | PotentialSSR | PentaSSR-Cp | 28264 | 28273 | TCAAT | 2 |  |  |
| cP0000055 | PotentialSSR | PentaSSR-Cp | 28290 | 28299 | TTGGC | 2 |  |  |
| cP0000056 | PotentialSSR | PentaSSR-Cp | 28348 | 28357 | ACTTT | 2 |  |  |
| c70000009 | ExtendedSSR | 7SSR-Cp | 28415 | 28428 | TTATAGT | 2 |  |  |
| cP0000057 | PotentialSSR | PentaSSR-Cp | 28452 | 28461 | CTAAT | 2 |  |  |
| cP0000058 | PotentialSSR | PentaSSR-Cp | 28828 | 28837 | TATCA | 2 |  |  |
| cH0000029 | PotentialSSR | HexaSSR-Cp | 29053 | 29064 | CAAAAA | 2 |  |  |
| cM0000009 | SSR | MonoSSR-Cp | 29379 | 29388 | A | 10 |  |  |
| cP0000059 | PotentialSSR | PentaSSR-Cp | 29757 | 29766 | ATAAA | 2 |  |  |
| c80000004 | ExtendedSSR | 8SSR-Cp | 29776 | 29791 | TTTTCTTT | 2 |  |  |
| cP0000060 | PotentialSSR | PentaSSR-Cp | 29833 | 29842 | ACCAT | 2 |  |  |
| cH0000030 | PotentialSSR | HexaSSR-Cp | 29873 | 29884 | AATTTC | 2 |  |  |
| cTe0000003 | SSR | TetraSSR-Cp | 29892 | 29903 | AAAT | 3 |  |  |
| cP0000061 | PotentialSSR | PentaSSR-Cp | 30111 | 30120 | TCAAT | 2 |  |  |
| cP0000062 | PotentialSSR | PentaSSR-Cp | 30162 | 30171 | TACCC | 2 | trnE-UUC | trnE-UUC [trnE-UUC]\| |
| cH0000031 | PotentialSSR | HexaSSR-Cp | 30366 | 30377 | ATTTCA | 2 |  |  |
| cH0000032 | PotentialSSR | HexaSSR-Cp | 30504 | 30515 | CTAATA | 2 |  |  |
| cP0000063 | PotentialSSR | PentaSSR-Cp | 30648 | 30657 | GTATA | 2 |  |  |
| cP0000064 | PotentialSSR | PentaSSR-Cp | 30960 | 30969 | ATTAT | 2 |  |  |
| cP0000065 | PotentialSSR | PentaSSR-Cp | 31033 | 31042 | TGGAA | 2 |  |  |
| c70000010 | ExtendedSSR | 7SSR-Cp | 31494 | 31507 | GTTTATT | 2 |  |  |
| cH0000033 | PotentialSSR | HexaSSR-Cp | 31571 | 31582 | GTGAAA | 2 |  |  |
| cP0000066 | PotentialSSR | PentaSSR-Cp | 31747 | 31756 | AAAGA | 2 |  |  |
| cP0000067 | PotentialSSR | PentaSSR-Cp | 31954 | 31963 | TAAAT | 2 |  |  |
| cP0000068 | PotentialSSR | PentaSSR-Cp | 32117 | 32126 | CGTTT | 2 | psbD | YP_009380209.1 [psbD]\| |
| cP0000069 | PotentialSSR | PentaSSR-Cp | 32747 | 32756 | AACCC | 2 | psbD | YP_009380209.1 [psbD]\| |
| cP0000070 | PotentialSSR | PentaSSR-Cp | 33840 | 33849 | GTCTG | 2 | psbC | YP_009380210.1 [psbC]\| |
| cH0000034 | PotentialSSR | HexaSSR-Cp | 33976 | 33987 | CTCAAG | 2 | psbC | YP_009380210.1 [psbC]\| |
| cP0000071 | PotentialSSR | PentaSSR-Cp | 34268 | 34277 | GGGTG | 2 | psbC | YP_009380210.1 [psbC]\| |
| cH0000035 | PotentialSSR | HexaSSR-Cp | 34394 | 34405 | TGCAGC | 2 | psbC | YP_009380210.1 [psbC]\| |
| c70000011 | ExtendedSSR | 7SSR-Cp | 34517 | 34530 | TACATAT | 2 |  |  |
| cP0000072 | PotentialSSR | PentaSSR-Cp | 34595 | 34604 | TAATT | 2 |  |  |
| cH0000036 | PotentialSSR | HexaSSR-Cp | 34826 | 34837 | TTATTC | 2 |  |  |
| cP0000073 | PotentialSSR | PentaSSR-Cp | 34928 | 34937 | TATAT | 2 |  |  |
| c70000012 | ExtendedSSR | 7SSR-Cp | 34968 | 34981 | TTAATTA | 2 |  |  |
| cP0000074 | PotentialSSR | PentaSSR-Cp | 35304 | 35313 | TGGAT | 2 | psbZ | YP_009380211.1 [psbZ]\| |
| cP0000075 | PotentialSSR | PentaSSR-Cp | 35472 | 35481 | AAACA | 2 |  |  |
| cP0000076 | PotentialSSR | PentaSSR-Cp | 35635 | 35644 | TATAT | 2 |  |  |
| cH0000037 | PotentialSSR | HexaSSR-Cp | 35801 | 35812 | GTTATA | 2 |  |  |
| cP0000077 | PotentialSSR | PentaSSR-Cp | 35824 | 35833 | TAGTG | 2 |  |  |
| cP0000078 | PotentialSSR | PentaSSR-Cp | 35927 | 35936 | TATTT | 2 |  |  |
| cP0000079 | PotentialSSR | PentaSSR-Cp | 36838 | 36847 | CCACG | 2 | psaB | YP_009380213.1 [psaB]\| |
| cP0000080 | PotentialSSR | PentaSSR-Cp | 36987 | 36996 | CCATC | 2 | psaB | YP_009380213.1 [psaB]\| |
| cP0000081 | PotentialSSR | PentaSSR-Cp | 38266 | 38275 | TGTCC | 2 | psaB | YP_009380213.1 [psaB]\| |
| cP0000082 | PotentialSSR | PentaSSR-Cp | 38772 | 38781 | ACCAA | 2 | psaB | YP_009380213.1 [psaB]\| |
| cH0000038 | PotentialSSR | HexaSSR-Cp | 39198 | 39209 | TAATAG | 2 | psaA | YP_009380214.1 [psaA]\| |
| cP0000083 | PotentialSSR | PentaSSR-Cp | 39947 | 39956 | ATGTG | 2 | psaA | YP_009380214.1 [psaA]\| |
| cP0000084 | PotentialSSR | PentaSSR-Cp | 41584 | 41593 | TATTT | 2 |  |  |
| cH0000039 | PotentialSSR | HexaSSR-Cp | 41762 | 41773 | TCTTTA | 2 |  |  |
| cP0000085 | PotentialSSR | PentaSSR-Cp | 41856 | 41865 | TTTTA | 2 |  |  |
| cM0000010 | SSR | MonoSSR-Cp | 41895 | 41904 | A | 10 |  |  |
| cP0000086 | PotentialSSR | PentaSSR-Cp | 42437 | 42446 | AAAGA | 2 | ycf3 | (Intron)YP_009380215.1 [ycf3]\| |
| cP0000087 | PotentialSSR | PentaSSR-Cp | 42574 | 42583 | TTCTT | 2 | ycf3 | (Intron)YP_009380215.1 [ycf3]\| |
| cM0000011 | SSR | MonoSSR-Cp | 42647 | 42656 | T | 10 | ycf3 | (Intron)YP_009380215.1 [ycf3]\| |
| cP0000088 | PotentialSSR | PentaSSR-Cp | 43148 | 43157 | AATAT | 2 | ycf3 | (Intron)YP_009380215.1 [ycf3]\| |
| cP0000089 | PotentialSSR | PentaSSR-Cp | 43823 | 43832 | AAATC | 2 | ycf3 | (Intron)YP_009380215.1 [ycf3]\| |
| cP0000090 | PotentialSSR | PentaSSR-Cp | 44032 | 44041 | GTACA | 2 |  |  |
| cP0000091 | PotentialSSR | PentaSSR-Cp | 44121 | 44130 | TATTT | 2 |  |  |
| cP0000092 | PotentialSSR | PentaSSR-Cp | 44208 | 44217 | CAAAT | 2 |  |  |
| cP0000093 | PotentialSSR | PentaSSR-Cp | 44222 | 44231 | TAGTA | 2 |  |  |
| cP0000094 | PotentialSSR | PentaSSR-Cp | 44275 | 44284 | GATCA | 2 |  |  |
| cP0000095 | PotentialSSR | PentaSSR-Cp | 44344 | 44353 | AATAG | 2 |  |  |
| cP0000096 | PotentialSSR | PentaSSR-Cp | 44521 | 44530 | GATTC | 2 |  |  |
| cTe0000004 | SSR | TetraSSR-Cp | 44536 | 44547 | AATT | 3 |  |  |
| cP0000097 | PotentialSSR | PentaSSR-Cp | 44556 | 44565 | TATAT | 2 |  |  |
| cD0000004 | SSR | DiSSR-Cp | 44628 | 44637 | AT | 5 |  |  |
| cP0000098 | PotentialSSR | PentaSSR-Cp | 44656 | 44665 | TTTTA | 2 |  |  |
| cP0000099 | PotentialSSR | PentaSSR-Cp | 44707 | 44716 | ATTAC | 2 |  |  |
| cP0000100 | PotentialSSR | PentaSSR-Cp | 44755 | 44764 | ATAGT | 2 |  |  |
| cP0000101 | PotentialSSR | PentaSSR-Cp | 44840 | 44849 | ATAGT | 2 |  |  |
| cP0000102 | PotentialSSR | PentaSSR-Cp | 45028 | 45037 | CAAAT | 2 |  |  |
| cP0000103 | PotentialSSR | PentaSSR-Cp | 45204 | 45213 | GGGAT | 2 |  |  |
| cH0000040 | PotentialSSR | HexaSSR-Cp | 45271 | 45282 | TTTTCC | 2 |  |  |
| cP0000104 | PotentialSSR | PentaSSR-Cp | 45697 | 45706 | ACCTG | 2 | rps4 | YP_009380216.1 [rps4]\| |
| cD0000005 | SSR | DiSSR-Cp | 45980 | 45993 | TA | 7 |  |  |
| cP0000105 | PotentialSSR | PentaSSR-Cp | 46038 | 46047 | AAATG | 2 |  |  |
| cP0000106 | PotentialSSR | PentaSSR-Cp | 46129 | 46138 | TATTT | 2 |  |  |
| cH0000041 | PotentialSSR | HexaSSR-Cp | 46158 | 46169 | AAAAAG | 2 |  |  |
| cP0000107 | PotentialSSR | PentaSSR-Cp | 46213 | 46222 | CTTTT | 2 |  |  |
| cP0000108 | PotentialSSR | PentaSSR-Cp | 46505 | 46514 | TCAAA | 2 |  |  |
| cP0000109 | PotentialSSR | PentaSSR-Cp | 46583 | 46592 | CGATT | 2 |  |  |
| cP0000110 | PotentialSSR | PentaSSR-Cp | 46819 | 46828 | AATAA | 2 |  |  |
| cP0000111 | PotentialSSR | PentaSSR-Cp | 47408 | 47417 | CTGTG | 2 | trnL-UAA | (Intron)trnL-UAA [trnL-UAA]\| |
| cP0000112 | PotentialSSR | PentaSSR-Cp | 47501 | 47510 | TCTAT | 2 | trnL-UAA | (Intron)trnL-UAA [trnL-UAA]\| |
| cP0000113 | PotentialSSR | PentaSSR-Cp | 47892 | 47901 | TTTTC | 2 |  |  |
| cD0000006 | SSR | DiSSR-Cp | 47949 | 47958 | AT | 5 |  |  |
| cP0000114 | PotentialSSR | PentaSSR-Cp | 48263 | 48272 | CACAT | 2 |  |  |
| cH0000043 | PotentialSSR | HexaSSR-Cp | 48392 | 48403 | GAAAAT | 2 |  |  |
| cP0000115 | PotentialSSR | PentaSSR-Cp | 48523 | 48532 | CTTAA | 2 |  |  |
| cP0000116 | PotentialSSR | PentaSSR-Cp | 48542 | 48551 | TCTTT | 2 |  |  |
| cH0000044 | PotentialSSR | HexaSSR-Cp | 49011 | 49022 | CATATA | 2 | ndhJ | YP_009380217.1 [ndhJ]\| |
| c80000006 | ExtendedSSR | 8SSR-Cp | 49168 | 49183 | ATTTGTTT | 2 |  |  |
| cP0000117 | PotentialSSR | PentaSSR-Cp | 49191 | 49200 | TATTT | 2 |  |  |
| cP0000118 | PotentialSSR | PentaSSR-Cp | 49204 | 49213 | TTTAC | 2 |  |  |
| cH0000045 | PotentialSSR | HexaSSR-Cp | 49971 | 49982 | TTTTTC | 2 |  |  |
| cP0000119 | PotentialSSR | PentaSSR-Cp | 50036 | 50045 | TAAAC | 2 | ndhC | YP_009380219.1 [ndhC]\| |
| cP0000120 | PotentialSSR | PentaSSR-Cp | 50598 | 50607 | TTCTT | 2 |  |  |
| cP0000121 | PotentialSSR | PentaSSR-Cp | 50674 | 50683 | AATTC | 2 |  |  |
| cP0000122 | PotentialSSR | PentaSSR-Cp | 50730 | 50739 | TTCTA | 2 |  |  |
| cP0000123 | PotentialSSR | PentaSSR-Cp | 50789 | 50798 | ACTTG | 2 |  |  |
| c70000015 | ExtendedSSR | 7SSR-Cp | 52546 | 52559 | AATTTTT | 2 |  |  |
| cP0000124 | PotentialSSR | PentaSSR-Cp | 52684 | 52693 | ATTTG | 2 |  |  |
| cP0000125 | PotentialSSR | PentaSSR-Cp | 52785 | 52794 | TTGAA | 2 |  |  |
| cH0000047 | PotentialSSR | HexaSSR-Cp | 52838 | 52849 | TGTATA | 2 |  |  |
| cP0000126 | PotentialSSR | PentaSSR-Cp | 53065 | 53074 | ATTGA | 2 |  |  |
| cP0000127 | PotentialSSR | PentaSSR-Cp | 53104 | 53113 | AATTG | 2 |  |  |
| cM0000012 | SSR | MonoSSR-Cp | 53195 | 53206 | T | 12 |  |  |
| cH0000048 | PotentialSSR | HexaSSR-Cp | 53217 | 53228 | ATTTTC | 2 |  |  |
| cM0000013 | SSR | MonoSSR-Cp | 53302 | 53311 | A | 10 | atpB | YP_009380221.1 [atpB]\| |
| cP0000128 | PotentialSSR | PentaSSR-Cp | 54478 | 54487 | TTGGA | 2 | atpB | YP_009380221.1 [atpB]\| |
| cH0000049 | PotentialSSR | HexaSSR-Cp | 55175 | 55186 | ATAATC | 2 |  |  |
| cH0000050 | PotentialSSR | HexaSSR-Cp | 55245 | 55256 | TCTTTT | 2 |  |  |
| cH0000051 | PotentialSSR | HexaSSR-Cp | 55283 | 55294 | AAAAAG | 2 |  |  |
| cP0000129 | PotentialSSR | PentaSSR-Cp | 55757 | 55766 | AAACA | 2 | trnV-UAC | (Intron)trnV-UAC [trnV-UAC]\| |
| c70000016 | ExtendedSSR | 7SSR-Cp | 56089 | 56102 | AATGAAT | 2 | trnV-UAC | (Intron)trnV-UAC [trnV-UAC]\| |
| cH0000052 | PotentialSSR | HexaSSR-Cp | 56335 | 56346 | CAATTG | 2 |  |  |
| cP0000130 | PotentialSSR | PentaSSR-Cp | 56396 | 56405 | ATTTG | 2 |  |  |
| cH0000053 | PotentialSSR | HexaSSR-Cp | 56420 | 56431 | TTTGAA | 2 |  |  |
| cP0000131 | PotentialSSR | PentaSSR-Cp | 56519 | 56528 | TTTTC | 2 |  |  |
| cP0000132 | PotentialSSR | PentaSSR-Cp | 58585 | 58594 | TATAT | 2 |  |  |
| cP0000133 | PotentialSSR | PentaSSR-Cp | 58641 | 58650 | CTATA | 2 |  |  |
| cTe0000005 | SSR | TetraSSR-Cp | 58668 | 58679 | TAAT | 3 |  |  |
| cP0000134 | PotentialSSR | PentaSSR-Cp | 58682 | 58691 | TAATA | 2 |  |  |
| cH0000054 | PotentialSSR | HexaSSR-Cp | 58723 | 58734 | TACAAT | 2 |  |  |
| cP0000135 | PotentialSSR | PentaSSR-Cp | 58853 | 58862 | AAAAT | 2 | psaI | YP_009380224.1 [psaI]\| |
| cP0000136 | PotentialSSR | PentaSSR-Cp | 58871 | 58880 | TCGTC | 2 |  |  |
| cP0000137 | PotentialSSR | PentaSSR-Cp | 59011 | 59020 | ACAAT | 2 |  |  |
| cP0000138 | PotentialSSR | PentaSSR-Cp | 59175 | 59184 | TTCAA | 2 |  |  |
| cP0000139 | PotentialSSR | PentaSSR-Cp | 59485 | 59494 | TCGAT | 2 | ycf4 | YP_009380225.1 [ycf4]\| |
| cP0000140 | PotentialSSR | PentaSSR-Cp | 59590 | 59599 | TAGAA | 2 | ycf4 | YP_009380225.1 [ycf4]\| |
| cH0000055 | PotentialSSR | HexaSSR-Cp | 59923 | 59934 | TACAAA | 2 |  |  |
| cP0000141 | PotentialSSR | PentaSSR-Cp | 59936 | 59945 | CAATT | 2 |  |  |
| cP0000142 | PotentialSSR | PentaSSR-Cp | 60076 | 60085 | AATTA | 2 |  |  |
| cP0000143 | PotentialSSR | PentaSSR-Cp | 61041 | 61050 | AAAAG | 2 |  |  |
| c70000017 | ExtendedSSR | 7SSR-Cp | 61105 | 61118 | ATAATCA | 2 |  |  |
| cP0000144 | PotentialSSR | PentaSSR-Cp | 61190 | 61199 | TTATT | 2 |  |  |
| cP0000145 | PotentialSSR | PentaSSR-Cp | 61688 | 61697 | AAAGA | 2 | petA | YP_009380227.1 [petA]\| |
| cP0000146 | PotentialSSR | PentaSSR-Cp | 61959 | 61968 | GAAAA | 2 | petA | YP_009380227.1 [petA]\| |
| cH0000056 | PotentialSSR | HexaSSR-Cp | 62279 | 62290 | ATCAAG | 2 |  |  |
| cH0000057 | PotentialSSR | HexaSSR-Cp | 62295 | 62306 | TAACAA | 2 |  |  |
| cP0000147 | PotentialSSR | PentaSSR-Cp | 62319 | 62328 | CAATT | 2 |  |  |
| cM0000014 | SSR | MonoSSR-Cp | 62353 | 62367 | A | 15 |  |  |
| c80000007 | ExtendedSSR | 8SSR-Cp | 62461 | 62476 | ACTTTTTT | 2 |  |  |
| cH0000058 | PotentialSSR | HexaSSR-Cp | 62668 | 62679 | ATTTTT | 2 |  |  |
| cH0000059 | PotentialSSR | HexaSSR-Cp | 62863 | 62874 | TCTTTC | 2 |  |  |
| cP0000148 | PotentialSSR | PentaSSR-Cp | 62877 | 62886 | TTAAC | 2 |  |  |
| c70000018 | ExtendedSSR | 7SSR-Cp | 63129 | 63142 | TTACTAC | 2 | psbJ | YP_009380228.1 [psbJ]\| |
| cP0000149 | PotentialSSR | PentaSSR-Cp | 63482 | 63491 | ATTCA | 2 | psbL | YP_009380229.1 [psbL]\| |
| cH0000060 | PotentialSSR | HexaSSR-Cp | 63533 | 63544 | ATTCGG | 2 |  |  |
| c80000008 | ExtendedSSR | 8SSR-Cp | 64013 | 64028 | ACGTAAAA | 2 |  |  |
| cH0000061 | PotentialSSR | HexaSSR-Cp | 64350 | 64361 | TATAGA | 2 |  |  |
| cH0000062 | PotentialSSR | HexaSSR-Cp | 64589 | 64600 | AGTCTT | 2 |  |  |
| cP0000150 | PotentialSSR | PentaSSR-Cp | 65145 | 65154 | CTGTA | 2 |  |  |
| cM0000015 | SSR | MonoSSR-Cp | 65260 | 65273 | T | 14 |  |  |
| cP0000151 | PotentialSSR | PentaSSR-Cp | 65465 | 65474 | GATTA | 2 |  |  |
| cP0000152 | PotentialSSR | PentaSSR-Cp | 65482 | 65491 | TTTAG | 2 |  |  |
| cH0000063 | PotentialSSR | HexaSSR-Cp | 65501 | 65512 | TTTCTA | 2 |  |  |
| cP0000153 | PotentialSSR | PentaSSR-Cp | 65599 | 65608 | GAACT | 2 | trnW-CCA | trnW-CCA [trnW-CCA]\| |
| cH0000064 | PotentialSSR | HexaSSR-Cp | 65675 | 65686 | CTATAT | 2 |  |  |
| cP0000154 | PotentialSSR | PentaSSR-Cp | 65882 | 65891 | TTCAA | 2 |  |  |
| cP0000155 | PotentialSSR | PentaSSR-Cp | 65921 | 65930 | CCTTG | 2 |  |  |
| cP0000156 | PotentialSSR | PentaSSR-Cp | 66057 | 66066 | GTAAA | 2 |  |  |
| cP0000157 | PotentialSSR | PentaSSR-Cp | 66169 | 66178 | CTTAG | 2 |  |  |
| cP0000158 | PotentialSSR | PentaSSR-Cp | 66525 | 66534 | TTAGT | 2 |  |  |
| cP0000159 | PotentialSSR | PentaSSR-Cp | 66578 | 66587 | GTTAA | 2 |  |  |
| c70000019 | ExtendedSSR | 7SSR-Cp | 66719 | 66732 | CGAATTG | 2 |  |  |
| cH0000065 | PotentialSSR | HexaSSR-Cp | 67082 | 67093 | AATTTT | 2 |  |  |
| cD0000007 | SSR | DiSSR-Cp | 67107 | 67116 | AT | 5 |  |  |
| cP0000160 | PotentialSSR | PentaSSR-Cp | 67176 | 67185 | ATATA | 2 |  |  |
| cP0000161 | PotentialSSR | PentaSSR-Cp | 67251 | 67260 | ATAGG | 2 |  |  |
| cP0000162 | PotentialSSR | PentaSSR-Cp | 67278 | 67287 | TAAAC | 2 |  |  |
| cTe0000006 | SSR | TetraSSR-Cp | 67762 | 67773 | TTTA | 3 |  |  |
| cP0000163 | PotentialSSR | PentaSSR-Cp | 68325 | 68334 | ATAAA | 2 |  |  |
| cH0000066 | PotentialSSR | HexaSSR-Cp | 68816 | 68827 | ATTTTC | 2 |  |  |
| cP0000164 | PotentialSSR | PentaSSR-Cp | 68934 | 68943 | CTATT | 2 |  |  |
| cH0000067 | PotentialSSR | HexaSSR-Cp | 68981 | 68992 | TTTTGT | 2 |  |  |
| cH0000068 | PotentialSSR | HexaSSR-Cp | 69301 | 69312 | TGGGCT | 2 | clpP | YP_009380239.1 [clpP]\| |
| cP0000165 | PotentialSSR | PentaSSR-Cp | 69531 | 69540 | ACACA | 2 | clpP | (Intron)YP_009380239.1 [clpP]\| |
| cP0000166 | PotentialSSR | PentaSSR-Cp | 69676 | 69685 | ATCGA | 2 | clpP | (Intron)YP_009380239.1 [clpP]\| |
| cH0000069 | PotentialSSR | HexaSSR-Cp | 69687 | 69698 | CAGATC | 2 | clpP | (Intron)YP_009380239.1 [clpP]\| |
| cP0000167 | PotentialSSR | PentaSSR-Cp | 69972 | 69981 | GAAAA | 2 | clpP | (Intron)YP_009380239.1 [clpP]\| |
| cH0000070 | PotentialSSR | HexaSSR-Cp | 70262 | 70273 | ACAAAT | 2 | clpP | YP_009380239.1 [clpP]\| |
| cP0000168 | PotentialSSR | PentaSSR-Cp | 70675 | 70684 | TATCA | 2 | clpP | (Intron)YP_009380239.1 [clpP]\| |
| cH0000071 | PotentialSSR | HexaSSR-Cp | 70715 | 70726 | TTCTTG | 2 | clpP | (Intron)YP_009380239.1 [clpP]\| |
| cH0000072 | PotentialSSR | HexaSSR-Cp | 70925 | 70936 | TTGAAA | 2 | clpP | (Intron)YP_009380239.1 [clpP]\| |
| cH0000073 | PotentialSSR | HexaSSR-Cp | 70992 | 71003 | ATTGGG | 2 | clpP | (Intron)YP_009380239.1 [clpP]\| |
| cP0000169 | PotentialSSR | PentaSSR-Cp | 71561 | 71570 | ATAGA | 2 |  |  |
| cH0000074 | PotentialSSR | HexaSSR-Cp | 71680 | 71691 | CATAGT | 2 |  |  |
| c70000021 | ExtendedSSR | 7SSR-Cp | 71830 | 71843 | CTGGTTG | 2 | psbB | YP_009380240.1 [psbB]\| |
| cH0000075 | PotentialSSR | HexaSSR-Cp | 72194 | 72205 | GTTTTG | 2 | psbB | YP_009380240.1 [psbB]\| |
| cP0000170 | PotentialSSR | PentaSSR-Cp | 73300 | 73309 | TTTGA | 2 |  |  |
| cP0000171 | PotentialSSR | PentaSSR-Cp | 73457 | 73466 | CTCTA | 2 | psbT | YP_009380241.1 [psbT]\| |
| cP0000172 | PotentialSSR | PentaSSR-Cp | 73521 | 73530 | AAATG | 2 | psbT | YP_009380241.1 [psbT]\| |
| cH0000076 | PotentialSSR | HexaSSR-Cp | 74052 | 74063 | ACAAAA | 2 |  |  |
| c90000002 | ExtendedSSR | 9SSR-Cp | 74101 | 74118 | CAATACAAA | 2 |  |  |
| cP0000173 | PotentialSSR | PentaSSR-Cp | 74404 | 74413 | TTTTC | 2 | petB | (Intron)YP_009380244.1 [petB]\| |
| cP0000174 | PotentialSSR | PentaSSR-Cp | 74522 | 74531 | AATAA | 2 | petB | (Intron)YP_009380244.1 [petB]\| |
| cP0000175 | PotentialSSR | PentaSSR-Cp | 74534 | 74543 | CCTAT | 2 | petB | (Intron)YP_009380244.1 [petB]\| |
| cH0000077 | PotentialSSR | HexaSSR-Cp | 74710 | 74721 | ATTATA | 2 | petB | (Intron)YP_009380244.1 [petB]\| |
| cH0000078 | PotentialSSR | HexaSSR-Cp | 74722 | 74733 | TACAAA | 2 | petB | (Intron)YP_009380244.1 [petB]\| |
| cP0000176 | PotentialSSR | PentaSSR-Cp | 75664 | 75673 | ATAGA | 2 | petB | YP_009380244.1 [petB]\| |
| cP0000177 | PotentialSSR | PentaSSR-Cp | 76033 | 76042 | TCTAT | 2 | petD | (Intron)YP_009380245.1 [petD]\| |
| cP0000178 | PotentialSSR | PentaSSR-Cp | 76109 | 76118 | ATAAT | 2 | petD | (Intron)YP_009380245.1 [petD]\| |
| cP0000179 | PotentialSSR | PentaSSR-Cp | 76123 | 76132 | ATTAT | 2 | petD | (Intron)YP_009380245.1 [petD]\| |
| cP0000180 | PotentialSSR | PentaSSR-Cp | 76146 | 76155 | TTATT | 2 | petD | (Intron)YP_009380245.1 [petD]\| |
| cP0000181 | PotentialSSR | PentaSSR-Cp | 76618 | 76627 | GAATC | 2 | petD | (Intron)YP_009380245.1 [petD]\| |
| cP0000182 | PotentialSSR | PentaSSR-Cp | 76631 | 76640 | AAGAA | 2 | petD | (Intron)YP_009380245.1 [petD]\| |
| cP0000183 | PotentialSSR | PentaSSR-Cp | 77210 | 77219 | ATTCA | 2 |  |  |
| cM0000016 | SSR | MonoSSR-Cp | 77437 | 77446 | T | 10 | rpoA | YP_009380246.1 [rpoA]\| |
| cH0000079 | PotentialSSR | HexaSSR-Cp | 77656 | 77667 | CATTTC | 2 | rpoA | YP_009380246.1 [rpoA]\| |
| cP0000184 | PotentialSSR | PentaSSR-Cp | 78086 | 78095 | TCGCA | 2 | rpoA | YP_009380246.1 [rpoA]\| |
| cP0000185 | PotentialSSR | PentaSSR-Cp | 78854 | 78863 | TAGTA | 2 |  |  |
| cM0000017 | SSR | MonoSSR-Cp | 78997 | 79007 | T | 11 |  |  |
| cH0000080 | PotentialSSR | HexaSSR-Cp | 79080 | 79091 | GTTGAA | 2 |  |  |
| cP0000186 | PotentialSSR | PentaSSR-Cp | 79912 | 79921 | TTAGT | 2 |  |  |
| cH0000081 | PotentialSSR | HexaSSR-Cp | 79983 | 79994 | TATTTT | 2 |  |  |
| cH0000082 | PotentialSSR | HexaSSR-Cp | 80990 | 81001 | AAAAAT | 2 | rpl16 | (Intron)YP_009380252.1 [rpl16]\| |
| cP0000187 | PotentialSSR | PentaSSR-Cp | 81017 | 81026 | TATTT | 2 | rpl16 | (Intron)YP_009380252.1 [rpl16]\| |
| c70000023 | ExtendedSSR | 7SSR-Cp | 81235 | 81248 | TTTTATA | 2 | rpl16 | (Intron)YP_009380252.1 [rpl16]\| |
| cP0000188 | PotentialSSR | PentaSSR-Cp | 81291 | 81300 | AAAAG | 2 | rpl16 | (Intron)YP_009380252.1 [rpl16]\| |
| cM0000018 | SSR | MonoSSR-Cp | 81577 | 81586 | T | 10 | rpl16 | (Intron)YP_009380252.1 [rpl16]\| |
| cM0000019 | SSR | MonoSSR-Cp | 81710 | 81720 | T | 11 | rpl16 | (Intron)YP_009380252.1 [rpl16]\| |
| cM0000020 | SSR | MonoSSR-Cp | 81769 | 81778 | T | 10 | rpl16 | (Intron)YP_009380252.1 [rpl16]\| |
| cH0000084 | PotentialSSR | HexaSSR-Cp | 81958 | 81969 | TTTTAA | 2 | rpl16 | (Intron)YP_009380252.1 [rpl16]\| |
| cP0000189 | PotentialSSR | PentaSSR-Cp | 82265 | 82274 | ACCCT | 2 | rps3 | YP_009380253.1 [rps3]\| |
| cP0000190 | PotentialSSR | PentaSSR-Cp | 82399 | 82408 | TACTC | 2 | rps3 | YP_009380253.1 [rps3]\| |
| cP0000191 | PotentialSSR | PentaSSR-Cp | 82502 | 82511 | CAATT | 2 | rps3 | YP_009380253.1 [rps3]\| |
| cP0000192 | PotentialSSR | PentaSSR-Cp | 82860 | 82869 | TTTAT | 2 | rpl22 | YP_009380254.1 [rpl22]\| |
| cP0000193 | PotentialSSR | PentaSSR-Cp | 83381 | 83390 | TTTCT | 2 |  |  |
| cH0000085 | PotentialSSR | HexaSSR-Cp | 83810 | 83821 | ATTTTC | 2 |  |  |
| cP0000194 | PotentialSSR | PentaSSR-Cp | 84225 | 84234 | TATGT | 2 | rpl2 | YP_009380256.1 [rpl2]\| |
| cP0000195 | PotentialSSR | PentaSSR-Cp | 84573 | 84582 | TGGAT | 2 | rpl2 | YP_009380256.1 [rpl2]\| |
| cP0000196 | PotentialSSR | PentaSSR-Cp | 84674 | 84683 | TTCTT | 2 |  |  |
| cP0000197 | PotentialSSR | PentaSSR-Cp | 84695 | 84704 | GAATA | 2 |  |  |
| cP0000198 | PotentialSSR | PentaSSR-Cp | 85053 | 85062 | TATGA | 2 |  |  |
| cP0000199 | PotentialSSR | PentaSSR-Cp | 85269 | 85278 | TGAAA | 2 |  |  |
| cH0000086 | PotentialSSR | HexaSSR-Cp | 85935 | 85946 | GAAAGA | 2 | ycf2 | YP_009380257.1 [ycf2]\| |
| cP0000200 | PotentialSSR | PentaSSR-Cp | 85987 | 85996 | GATCC | 2 | ycf2 | YP_009380257.1 [ycf2]\| |
| c90000003 | ExtendedSSR | 9SSR-Cp | 87267 | 87284 | GGAACATTT | 2 | ycf2 | YP_009380257.1 [ycf2]\| |
| cP0000201 | PotentialSSR | PentaSSR-Cp | 88114 | 88123 | CGATC | 2 | ycf2 | YP_009380257.1 [ycf2]\| |
| cP0000202 | PotentialSSR | PentaSSR-Cp | 88279 | 88288 | TTCAA | 2 | ycf2 | YP_009380257.1 [ycf2]\| |
| cH0000088 | PotentialSSR | HexaSSR-Cp | 89056 | 89067 | GGTCCC | 2 | ycf2 | YP_009380257.1 [ycf2]\| |
| cH0000089 | PotentialSSR | HexaSSR-Cp | 89232 | 89243 | AAGAAA | 2 | ycf2 | YP_009380257.1 [ycf2]\| |
| cP0000203 | PotentialSSR | PentaSSR-Cp | 89257 | 89266 | GATTG | 2 | ycf2 | YP_009380257.1 [ycf2]\| |
| cH0000090 | PotentialSSR | HexaSSR-Cp | 89898 | 89909 | GGAGCT | 2 | ycf2 | YP_009380257.1 [ycf2]\| |
| cP0000204 | PotentialSSR | PentaSSR-Cp | 90280 | 90289 | GAAAA | 2 | ycf2 | YP_009380257.1 [ycf2]\| |
| cH0000091 | PotentialSSR | HexaSSR-Cp | 90785 | 90796 | TAGAAG | 2 | ycf2 | YP_009380257.1 [ycf2]\| |
| cH0000092 | PotentialSSR | HexaSSR-Cp | 91385 | 91396 | CTATAT | 2 | ycf2 | YP_009380257.1 [ycf2]\| |
| cP0000205 | PotentialSSR | PentaSSR-Cp | 91879 | 91888 | AAGTT | 2 |  |  |
| cP0000206 | PotentialSSR | PentaSSR-Cp | 91907 | 91916 | TTGTT | 2 |  |  |
| cP0000207 | PotentialSSR | PentaSSR-Cp | 92151 | 92160 | GTTAC | 2 |  |  |
| cH0000093 | PotentialSSR | HexaSSR-Cp | 92326 | 92337 | ATTCCA | 2 |  |  |
| cP0000208 | PotentialSSR | PentaSSR-Cp | 92642 | 92651 | CTTAT | 2 |  |  |
| cP0000209 | PotentialSSR | PentaSSR-Cp | 92777 | 92786 | ATGGA | 2 |  |  |
| cH0000094 | PotentialSSR | HexaSSR-Cp | 93738 | 93749 | GCTGAA | 2 | ndhB | YP_009380258.1 [ndhB]\| |
| cH0000095 | PotentialSSR | HexaSSR-Cp | 93797 | 93808 | AGAGTC | 2 | ndhB | YP_009380258.1 [ndhB]\| |
| cP0000210 | PotentialSSR | PentaSSR-Cp | 94093 | 94102 | TGATT | 2 | ndhB | (Intron)YP_009380258.1 [ndhB]\| |
| cP0000211 | PotentialSSR | PentaSSR-Cp | 94293 | 94302 | AAAGA | 2 | ndhB | (Intron)YP_009380258.1 [ndhB]\| |
| cH0000096 | PotentialSSR | HexaSSR-Cp | 95360 | 95371 | TTCTTA | 2 |  |  |
| cP0000212 | PotentialSSR | PentaSSR-Cp | 95426 | 95435 | AGAAA | 2 |  |  |
| cP0000213 | PotentialSSR | PentaSSR-Cp | 95530 | 95539 | CTGTT | 2 |  |  |
| cH0000097 | PotentialSSR | HexaSSR-Cp | 96277 | 96288 | TCCATA | 2 |  |  |
| cP0000214 | PotentialSSR | PentaSSR-Cp | 96465 | 96474 | CGAAT | 2 |  |  |
| cH0000098 | PotentialSSR | HexaSSR-Cp | 97054 | 97065 | TTGATT | 2 |  |  |
| cH0000099 | PotentialSSR | HexaSSR-Cp | 97101 | 97112 | TTCCTC | 2 |  |  |
| cH0000100 | PotentialSSR | HexaSSR-Cp | 97113 | 97124 | TATCCC | 2 |  |  |
| cP0000215 | PotentialSSR | PentaSSR-Cp | 97359 | 97368 | TGTTG | 2 |  |  |
| cP0000216 | PotentialSSR | PentaSSR-Cp | 97457 | 97466 | TATTA | 2 |  |  |
| cP0000217 | PotentialSSR | PentaSSR-Cp | 97476 | 97485 | ATTAG | 2 |  |  |
| cP0000218 | PotentialSSR | PentaSSR-Cp | 97658 | 97667 | GCAAT | 2 |  |  |
| cH0000101 | PotentialSSR | HexaSSR-Cp | 97778 | 97789 | TATTAC | 2 |  |  |
| cH0000102 | PotentialSSR | HexaSSR-Cp | 97925 | 97936 | AATGGA | 2 |  |  |
| cM0000021 | SSR | MonoSSR-Cp | 98035 | 98046 | T | 12 |  |  |
| cP0000219 | PotentialSSR | PentaSSR-Cp | 98286 | 98295 | CAAGA | 2 |  |  |
| cP0000220 | PotentialSSR | PentaSSR-Cp | 98369 | 98378 | AGGGA | 2 |  |  |
| cH0000103 | PotentialSSR | HexaSSR-Cp | 98493 | 98504 | GAATGA | 2 |  |  |
| cH0000104 | PotentialSSR | HexaSSR-Cp | 99366 | 99377 | GACACT | 2 | rrn16 | rrn16 [rrn16]\| |
| cH0000105 | PotentialSSR | HexaSSR-Cp | 100823 | 100834 | AATGGA | 2 | trnI-GAU | (Intron)trnI-GAU [trnI-GAU]\| |
| cH0000106 | PotentialSSR | HexaSSR-Cp | 101579 | 101590 | AAGAAT | 2 |  |  |
| cP0000221 | PotentialSSR | PentaSSR-Cp | 101810 | 101819 | ACAAA | 2 | trnA-UGC | (Intron)trnA-UGC [trnA-UGC]\| |
| cP0000222 | PotentialSSR | PentaSSR-Cp | 102129 | 102138 | TTCAA | 2 | trnA-UGC | (Intron)trnA-UGC [trnA-UGC]\| |
| c80000010 | ExtendedSSR | 8SSR-Cp | 102353 | 102368 | TTTTGAGA | 2 | trnA-UGC | (Intron)trnA-UGC [trnA-UGC]\| |
| cD0000008 | SSR | DiSSR-Cp | 103928 | 103937 | CG | 5 | rrn23 | rrn23 [rrn23]\| |
| cH0000107 | PotentialSSR | HexaSSR-Cp | 103951 | 103962 | GAAGCG | 2 | rrn23 | rrn23 [rrn23]\| |
| cTe0000007 | SSR | TetraSSR-Cp | 104345 | 104356 | AGGT | 3 | rrn23 | rrn23 [rrn23]\| |
| cP0000223 | PotentialSSR | PentaSSR-Cp | 105438 | 105447 | GCGGA | 2 | rrn23 | rrn23 [rrn23]\| |
| cP0000224 | PotentialSSR | PentaSSR-Cp | 105736 | 105745 | ATCCA | 2 |  |  |
| c70000024 | ExtendedSSR | 7SSR-Cp | 105808 | 105821 | AAAAACC | 2 |  |  |
| cH0000108 | PotentialSSR | HexaSSR-Cp | 105838 | 105849 | TCTATC | 2 |  |  |
| cH0000109 | PotentialSSR | HexaSSR-Cp | 106215 | 106226 | TTCTTA | 2 |  |  |
| cP0000225 | PotentialSSR | PentaSSR-Cp | 106512 | 106521 | AGTGG | 2 |  |  |
| cM0000022 | SSR | MonoSSR-Cp | 106556 | 106565 | T | 10 |  |  |
| cH0000110 | PotentialSSR | HexaSSR-Cp | 106653 | 106664 | CAAGTA | 2 |  |  |
| cP0000226 | PotentialSSR | PentaSSR-Cp | 106669 | 106678 | TAGCA | 2 |  |  |
| cP0000227 | PotentialSSR | PentaSSR-Cp | 106693 | 106702 | GTCAT | 2 |  |  |
| c70000025 | ExtendedSSR | 7SSR-Cp | 106706 | 106719 | TATGTTT | 2 |  |  |
| cP0000228 | PotentialSSR | PentaSSR-Cp | 106878 | 106887 | CAGAA | 2 |  |  |
| c70000026 | ExtendedSSR | 7SSR-Cp | 106984 | 106997 | AAGAATG | 2 |  |  |
| c90000004 | ExtendedSSR | 9SSR-Cp | 107545 | 107562 | GAAGAAGGA | 2 | ycf1 | YP_009380260.1 [ycf1]\| |
| cH0000112 | PotentialSSR | HexaSSR-Cp | 108167 | 108178 | TAGAAA | 2 | ycf1 | YP_009380260.1 [ycf1]\| |
| cH0000113 | PotentialSSR | HexaSSR-Cp | 108234 | 108245 | TCCTTC | 2 | ycf1 | YP_009380260.1 [ycf1]\| |
| cP0000229 | PotentialSSR | PentaSSR-Cp | 108257 | 108266 | AAGAA | 2 | ycf1 | YP_009380260.1 [ycf1]\| |
| cH0000114 | PotentialSSR | HexaSSR-Cp | 108276 | 108287 | CAAAAT | 2 | ycf1 | YP_009380260.1 [ycf1]\| |
| cP0000230 | PotentialSSR | PentaSSR-Cp | 108323 | 108332 | ACAAA | 2 | ycf1 | YP_009380260.1 [ycf1]\| |
| cP0000231 | PotentialSSR | PentaSSR-Cp | 108548 | 108557 | GAAAT | 2 | ycf1 | YP_009380260.1 [ycf1]\| |
| cM0000023 | SSR | MonoSSR-Cp | 108734 | 108743 | A | 10 | ycf1 | YP_009380260.1 [ycf1]\| |
| cP0000232 | PotentialSSR | PentaSSR-Cp | 108848 | 108857 | AAAAT | 2 | ycf1,ndhF | YP_009380260.1 [ycf1]\|YP_009380261.1 [ndhF]\| |
| cH0000115 | PotentialSSR | HexaSSR-Cp | 110006 | 110017 | AGATCC | 2 | ndhF | YP_009380261.1 [ndhF]\| |
| c70000027 | ExtendedSSR | 7SSR-Cp | 110473 | 110486 | CTCGAAA | 2 | ndhF | YP_009380261.1 [ndhF]\| |
| cP0000233 | PotentialSSR | PentaSSR-Cp | 111007 | 111016 | AAAAG | 2 | ndhF | YP_009380261.1 [ndhF]\| |
| cP0000234 | PotentialSSR | PentaSSR-Cp | 111099 | 111108 | TAGAA | 2 |  |  |
| c70000028 | ExtendedSSR | 7SSR-Cp | 111272 | 111285 | TTAAAAC | 2 |  |  |
| cP0000235 | PotentialSSR | PentaSSR-Cp | 111528 | 111537 | TTACT | 2 |  |  |
| cM0000024 | SSR | MonoSSR-Cp | 111926 | 111935 | A | 10 |  |  |
| cM0000025 | SSR | MonoSSR-Cp | 112243 | 112252 | A | 10 |  |  |
| cP0000236 | PotentialSSR | PentaSSR-Cp | 112491 | 112500 | TTTTA | 2 |  |  |
| c70000029 | ExtendedSSR | 7SSR-Cp | 112511 | 112524 | TAAAAGA | 2 |  |  |
| cM0000026 | SSR | MonoSSR-Cp | 112562 | 112571 | A | 10 |  |  |
| cP0000237 | PotentialSSR | PentaSSR-Cp | 112858 | 112867 | AGAAA | 2 |  |  |
| cP0000238 | PotentialSSR | PentaSSR-Cp | 112987 | 112996 | GAAAA | 2 |  |  |
| cM0000027 | SSR | MonoSSR-Cp | 113120 | 113129 | A | 10 |  |  |
| cH0000116 | PotentialSSR | HexaSSR-Cp | 113173 | 113184 | TATGAA | 2 |  |  |
| cH0000117 | PotentialSSR | HexaSSR-Cp | 113433 | 113444 | AATGAA | 2 |  |  |
| cP0000239 | PotentialSSR | PentaSSR-Cp | 114617 | 114626 | TTTTG | 2 |  |  |
| cP0000240 | PotentialSSR | PentaSSR-Cp | 114655 | 114664 | TTTCA | 2 |  |  |
| cH0000118 | PotentialSSR | HexaSSR-Cp | 114753 | 114764 | AATACC | 2 | ndhD | YP_009380264.1 [ndhD]\| |
| cM0000028 | SSR | MonoSSR-Cp | 114819 | 114828 | A | 10 | ndhD | YP_009380264.1 [ndhD]\| |
| cH0000119 | PotentialSSR | HexaSSR-Cp | 115749 | 115760 | TAATTC | 2 | ndhD | YP_009380264.1 [ndhD]\| |
| cH0000120 | PotentialSSR | HexaSSR-Cp | 116601 | 116612 | TCTAGT | 2 |  |  |
| cP0000241 | PotentialSSR | PentaSSR-Cp | 117236 | 117245 | AAGTT | 2 |  |  |
| cP0000242 | PotentialSSR | PentaSSR-Cp | 117536 | 117545 | ATACC | 2 | ndhG | YP_009380267.1 [ndhG]\| |
| c70000030 | ExtendedSSR | 7SSR-Cp | 117748 | 117761 | TAGAATA | 2 | ndhG | YP_009380267.1 [ndhG]\| |
| cP0000243 | PotentialSSR | PentaSSR-Cp | 118023 | 118032 | TAAAG | 2 |  |  |
| cP0000244 | PotentialSSR | PentaSSR-Cp | 118188 | 118197 | TTTAA | 2 |  |  |
| cP0000245 | PotentialSSR | PentaSSR-Cp | 118309 | 118318 | TAATT | 2 | ndhI | YP_009380268.1 [ndhI]\| |
| cH0000121 | PotentialSSR | HexaSSR-Cp | 118951 | 118962 | GAACAA | 2 | ndhA | YP_009380269.1 [ndhA]\| |
| cP0000246 | PotentialSSR | PentaSSR-Cp | 118975 | 118984 | TAATG | 2 | ndhA | YP_009380269.1 [ndhA]\| |
| cP0000247 | PotentialSSR | PentaSSR-Cp | 119128 | 119137 | ATAAA | 2 | ndhA | YP_009380269.1 [ndhA]\| |
| cP0000248 | PotentialSSR | PentaSSR-Cp | 119356 | 119365 | AAGAT | 2 | ndhA | (Intron)YP_009380269.1 [ndhA]\| |
| cP0000249 | PotentialSSR | PentaSSR-Cp | 119544 | 119553 | CTATA | 2 | ndhA | (Intron)YP_009380269.1 [ndhA]\| |
| c70000031 | ExtendedSSR | 7SSR-Cp | 119666 | 119679 | TATCAAT | 2 | ndhA | (Intron)YP_009380269.1 [ndhA]\| |
| cM0000029 | SSR | MonoSSR-Cp | 119795 | 119804 | T | 10 | ndhA | (Intron)YP_009380269.1 [ndhA]\| |
| cH0000122 | PotentialSSR | HexaSSR-Cp | 119809 | 119820 | CTATTA | 2 | ndhA | (Intron)YP_009380269.1 [ndhA]\| |
| cP0000250 | PotentialSSR | PentaSSR-Cp | 121260 | 121269 | CATTC | 2 | ndhH | YP_009380270.1 [ndhH]\| |
| cH0000123 | PotentialSSR | HexaSSR-Cp | 122269 | 122280 | ATAATT | 2 |  |  |
| cP0000251 | PotentialSSR | PentaSSR-Cp | 122674 | 122683 | TTTAT | 2 |  |  |
| cH0000124 | PotentialSSR | HexaSSR-Cp | 122959 | 122970 | AATTTT | 2 | ycf1 | YP_009380272.1 [ycf1]\| |
| cP0000252 | PotentialSSR | PentaSSR-Cp | 123399 | 123408 | TTCTT | 2 | ycf1 | YP_009380272.1 [ycf1]\| |
| cM0000030 | SSR | MonoSSR-Cp | 123978 | 123989 | T | 12 | ycf1 | YP_009380272.1 [ycf1]\| |
| cH0000125 | PotentialSSR | HexaSSR-Cp | 124345 | 124356 | CTATAT | 2 | ycf1 | YP_009380272.1 [ycf1]\| |
| cH0000126 | PotentialSSR | HexaSSR-Cp | 124443 | 124454 | CAATAA | 2 | ycf1 | YP_009380272.1 [ycf1]\| |
| cTe0000008 | SSR | TetraSSR-Cp | 124477 | 124488 | TAAT | 3 | ycf1 | YP_009380272.1 [ycf1]\| |
| cM0000031 | SSR | MonoSSR-Cp | 125523 | 125533 | A | 11 | ycf1 | YP_009380272.1 [ycf1]\| |
| cP0000253 | PotentialSSR | PentaSSR-Cp | 125688 | 125697 | AAAAC | 2 | ycf1 | YP_009380272.1 [ycf1]\| |
| cP0000254 | PotentialSSR | PentaSSR-Cp | 127024 | 127033 | ATTTT | 2 | ycf1 | YP_009380272.1 [ycf1]\| |
| cM0000032 | SSR | MonoSSR-Cp | 127138 | 127147 | T | 10 | ycf1 | YP_009380272.1 [ycf1]\| |
| cP0000255 | PotentialSSR | PentaSSR-Cp | 127323 | 127332 | CATTT | 2 | ycf1 | YP_009380272.1 [ycf1]\| |
| cP0000256 | PotentialSSR | PentaSSR-Cp | 127549 | 127558 | TTTGT | 2 | ycf1 | YP_009380272.1 [ycf1]\| |
| cH0000127 | PotentialSSR | HexaSSR-Cp | 127594 | 127605 | ATTTTG | 2 | ycf1 | YP_009380272.1 [ycf1]\| |
| cP0000257 | PotentialSSR | PentaSSR-Cp | 127615 | 127624 | TTCTT | 2 | ycf1 | YP_009380272.1 [ycf1]\| |
| cH0000128 | PotentialSSR | HexaSSR-Cp | 127636 | 127647 | GAAGGA | 2 | ycf1 | YP_009380272.1 [ycf1]\| |
| cH0000129 | PotentialSSR | HexaSSR-Cp | 127703 | 127714 | TTTCTA | 2 | ycf1 | YP_009380272.1 [ycf1]\| |
| c90000005 | ExtendedSSR | 9SSR-Cp | 128319 | 128336 | TCCTTCTTC | 2 | ycf1 | YP_009380272.1 [ycf1]\| |
| c70000032 | ExtendedSSR | 7SSR-Cp | 128882 | 128895 | TTCATTC | 2 |  |  |
| cP0000258 | PotentialSSR | PentaSSR-Cp | 128994 | 129003 | TTCTG | 2 |  |  |
| c70000033 | ExtendedSSR | 7SSR-Cp | 129162 | 129175 | AAACATA | 2 |  |  |
| cP0000259 | PotentialSSR | PentaSSR-Cp | 129179 | 129188 | ATGAC | 2 |  |  |
| cP0000260 | PotentialSSR | PentaSSR-Cp | 129203 | 129212 | TGCTA | 2 |  |  |
| cH0000131 | PotentialSSR | HexaSSR-Cp | 129217 | 129228 | TACTTG | 2 |  |  |
| cM0000033 | SSR | MonoSSR-Cp | 129316 | 129325 | A | 10 |  |  |
| cP0000261 | PotentialSSR | PentaSSR-Cp | 129359 | 129368 | TCCAC | 2 |  |  |
| cH0000132 | PotentialSSR | HexaSSR-Cp | 129654 | 129665 | ATAAGA | 2 |  |  |
| cH0000133 | PotentialSSR | HexaSSR-Cp | 130032 | 130043 | GATAGA | 2 |  |  |
| c70000034 | ExtendedSSR | 7SSR-Cp | 130060 | 130073 | GGTTTTT | 2 |  |  |
| cP0000262 | PotentialSSR | PentaSSR-Cp | 130136 | 130145 | TGGAT | 2 |  |  |
| cP0000263 | PotentialSSR | PentaSSR-Cp | 130434 | 130443 | TCCGC | 2 | rrn23 | rrn23 [rrn23]\| |
| cTe0000009 | SSR | TetraSSR-Cp | 131523 | 131534 | CTAC | 3 | rrn23 | rrn23 [rrn23]\| |
| cH0000134 | PotentialSSR | HexaSSR-Cp | 131919 | 131930 | CGCTTC | 2 | rrn23 | rrn23 [rrn23]\| |
| cD0000009 | SSR | DiSSR-Cp | 131944 | 131953 | CG | 5 | rrn23 | rrn23 [rrn23]\| |
| c80000011 | ExtendedSSR | 8SSR-Cp | 133513 | 133528 | TCTCAAAA | 2 | trnA-UGC | (Intron)trnA-UGC [trnA-UGC]\| |
| cP0000264 | PotentialSSR | PentaSSR-Cp | 133742 | 133751 | ATTGA | 2 | trnA-UGC | (Intron)trnA-UGC [trnA-UGC]\| |
| cP0000265 | PotentialSSR | PentaSSR-Cp | 134062 | 134071 | TTTGT | 2 | trnA-UGC | (Intron)trnA-UGC [trnA-UGC]\| |
| cH0000135 | PotentialSSR | HexaSSR-Cp | 134291 | 134302 | ATTCTT | 2 |  |  |
| cH0000136 | PotentialSSR | HexaSSR-Cp | 135047 | 135058 | TCCATT | 2 | trnI-GAU | (Intron)trnI-GAU [trnI-GAU]\| |
| cH0000137 | PotentialSSR | HexaSSR-Cp | 136502 | 136513 | TCAGTG | 2 | rrn16 | rrn16 [rrn16]\| |
| cH0000138 | PotentialSSR | HexaSSR-Cp | 137377 | 137388 | TCATTC | 2 |  |  |
| cP0000266 | PotentialSSR | PentaSSR-Cp | 137503 | 137512 | TCCCT | 2 | trnV-GAC | trnV-GAC [trnV-GAC]\| |
| cP0000267 | PotentialSSR | PentaSSR-Cp | 137586 | 137595 | TCTTG | 2 |  |  |
| cM0000034 | SSR | MonoSSR-Cp | 137835 | 137846 | A | 12 |  |  |
| cH0000139 | PotentialSSR | HexaSSR-Cp | 137943 | 137954 | TTTCCA | 2 |  |  |
| cH0000140 | PotentialSSR | HexaSSR-Cp | 138089 | 138100 | ATAGTA | 2 |  |  |
| cP0000268 | PotentialSSR | PentaSSR-Cp | 138213 | 138222 | CATTG | 2 |  |  |
| cP0000269 | PotentialSSR | PentaSSR-Cp | 138396 | 138405 | CTAAT | 2 |  |  |
| cP0000270 | PotentialSSR | PentaSSR-Cp | 138415 | 138424 | TAATA | 2 |  |  |
| cP0000271 | PotentialSSR | PentaSSR-Cp | 138513 | 138522 | CAACA | 2 |  |  |
| cH0000141 | PotentialSSR | HexaSSR-Cp | 138757 | 138768 | GGGATA | 2 |  |  |
| cH0000142 | PotentialSSR | HexaSSR-Cp | 138769 | 138780 | GAGGAA | 2 |  |  |
| cH0000143 | PotentialSSR | HexaSSR-Cp | 138814 | 138825 | AAAATC | 2 |  |  |
| cP0000272 | PotentialSSR | PentaSSR-Cp | 139406 | 139415 | GATTC | 2 |  |  |
| cH0000144 | PotentialSSR | HexaSSR-Cp | 139593 | 139604 | TATGGA | 2 |  |  |
| cP0000273 | PotentialSSR | PentaSSR-Cp | 140341 | 140350 | GAACA | 2 |  |  |
| cP0000274 | PotentialSSR | PentaSSR-Cp | 140446 | 140455 | TTTCT | 2 |  |  |
| cH0000145 | PotentialSSR | HexaSSR-Cp | 140510 | 140521 | TAAGAA | 2 |  |  |
| cP0000275 | PotentialSSR | PentaSSR-Cp | 141579 | 141588 | TCTTT | 2 | ndhB | (Intron)YP_009380274.1 [ndhB]\| |
| cP0000276 | PotentialSSR | PentaSSR-Cp | 141779 | 141788 | AATCA | 2 | ndhB | (Intron)YP_009380274.1 [ndhB]\| |
| cH0000146 | PotentialSSR | HexaSSR-Cp | 142073 | 142084 | GACTCT | 2 | ndhB | YP_009380274.1 [ndhB]\| |
| cH0000147 | PotentialSSR | HexaSSR-Cp | 142130 | 142141 | GCTTCA | 2 | ndhB | YP_009380274.1 [ndhB]\| |
| cP0000277 | PotentialSSR | PentaSSR-Cp | 143094 | 143103 | TTCCA | 2 |  |  |
| cP0000278 | PotentialSSR | PentaSSR-Cp | 143230 | 143239 | ATAAG | 2 |  |  |
| cH0000148 | PotentialSSR | HexaSSR-Cp | 143543 | 143554 | TTGGAA | 2 |  |  |
| cP0000279 | PotentialSSR | PentaSSR-Cp | 143720 | 143729 | CGTAA | 2 |  |  |
| cP0000280 | PotentialSSR | PentaSSR-Cp | 143965 | 143974 | AACAA | 2 |  |  |
| cP0000281 | PotentialSSR | PentaSSR-Cp | 143993 | 144002 | AACTT | 2 |  |  |
| cH0000149 | PotentialSSR | HexaSSR-Cp | 144485 | 144496 | ATATAG | 2 | ycf2 | YP_009380275.1 [ycf2]\| |
| cH0000150 | PotentialSSR | HexaSSR-Cp | 145085 | 145096 | CTTCTA | 2 | ycf2 | YP_009380275.1 [ycf2]\| |
| cP0000282 | PotentialSSR | PentaSSR-Cp | 145592 | 145601 | TTTTC | 2 | ycf2 | YP_009380275.1 [ycf2]\| |
| cH0000151 | PotentialSSR | HexaSSR-Cp | 145971 | 145982 | CAGCTC | 2 | ycf2 | YP_009380275.1 [ycf2]\| |
| cP0000283 | PotentialSSR | PentaSSR-Cp | 146615 | 146624 | CAATC | 2 | ycf2 | YP_009380275.1 [ycf2]\| |
| cH0000152 | PotentialSSR | HexaSSR-Cp | 146635 | 146646 | CTTTTT | 2 | ycf2 | YP_009380275.1 [ycf2]\| |
| cH0000153 | PotentialSSR | HexaSSR-Cp | 146814 | 146825 | GGGACC | 2 | ycf2 | YP_009380275.1 [ycf2]\| |
| cP0000284 | PotentialSSR | PentaSSR-Cp | 147593 | 147602 | TTGAA | 2 | ycf2 | YP_009380275.1 [ycf2]\| |
| cP0000285 | PotentialSSR | PentaSSR-Cp | 147758 | 147767 | GATCG | 2 | ycf2 | YP_009380275.1 [ycf2]\| |
| c90000006 | ExtendedSSR | 9SSR-Cp | 148597 | 148614 | AAATGTTCC | 2 | ycf2 | YP_009380275.1 [ycf2]\| |
| cP0000286 | PotentialSSR | PentaSSR-Cp | 149884 | 149893 | CGGAT | 2 | ycf2 | YP_009380275.1 [ycf2]\| |
| cH0000155 | PotentialSSR | HexaSSR-Cp | 149935 | 149946 | TCTTTC | 2 | ycf2 | YP_009380275.1 [ycf2]\| |
| cP0000287 | PotentialSSR | PentaSSR-Cp | 150602 | 150611 | ATTTC | 2 |  |  |
| cP0000288 | PotentialSSR | PentaSSR-Cp | 150819 | 150828 | TCATA | 2 |  |  |
| cP0000289 | PotentialSSR | PentaSSR-Cp | 151177 | 151186 | TATTC | 2 |  |  |
| cP0000290 | PotentialSSR | PentaSSR-Cp | 151197 | 151206 | AAAGA | 2 |  |  |
| cP0000291 | PotentialSSR | PentaSSR-Cp | 151298 | 151307 | AATCC | 2 | rpl2 | YP_009380276.1 [rpl2]\| |
| cP0000292 | PotentialSSR | PentaSSR-Cp | 151647 | 151656 | ACATA | 2 | rpl2 | YP_009380276.1 [rpl2]\| |
| cH0000156 | PotentialSSR | HexaSSR-Cp | 152060 | 152071 | GAAAAT | 2 |  |  |

**Supplementary Table 2. List of SSRs identified in CAGOH01 of *C. album***

| **Name** | **SSR type** | **Type** | **Cooridnation** | | **Unit sequence** | **Repeat number** | **Genes** | **Annotation** |
| --- | --- | --- | --- | --- | --- | --- | --- | --- |
| cH0000001 | PotentialSSR | HexaSSR-Cp | 113 | 124 | GTAAAG | 2 |  |  |
| cH0000002 | PotentialSSR | HexaSSR-Cp | 1240 | 1251 | GCTTTC | 2 | psbA | YP_009380194.1 [psbA]\| |
| cP0000001 | PotentialSSR | PentaSSR-Cp | 1292 | 1301 | ATTTA | 2 |  |  |
| c70000001 | ExtendedSSR | 7SSR-Cp | 1454 | 1467 | AGAAAAT | 2 |  |  |
| c70000002 | ExtendedSSR | 7SSR-Cp | 1696 | 1709 | AGTAGAA | 2 | trnK-UUU | (Intron)trnK-UUU [trnK-UUU]\| |
| cH0000003 | PotentialSSR | HexaSSR-Cp | 1748 | 1759 | AATTTC | 2 | trnK-UUU | (Intron)trnK-UUU [trnK-UUU]\| |
| cH0000004 | PotentialSSR | HexaSSR-Cp | 2949 | 2960 | TTTTTC | 2 | matK,trnK-UUU | YP_009380195.1 [matK]\|(Intron)trnK-UUU [trnK-UUU]\| |
| cM0000001 | SSR | MonoSSR-Cp | 3483 | 3493 | T | 11 | trnK-UUU | (Intron)trnK-UUU [trnK-UUU]\| |
| cP0000002 | PotentialSSR | PentaSSR-Cp | 4093 | 4102 | TATGT | 2 |  |  |
| cP0000003 | PotentialSSR | PentaSSR-Cp | 4124 | 4133 | AATGG | 2 |  |  |
| cP0000004 | PotentialSSR | PentaSSR-Cp | 4202 | 4211 | CAGAT | 2 |  |  |
| cP0000005 | PotentialSSR | PentaSSR-Cp | 4355 | 4364 | CATTT | 2 |  |  |
| cH0000005 | PotentialSSR | HexaSSR-Cp | 4442 | 4453 | AATATT | 2 |  |  |
| cTe0000001 | SSR | TetraSSR-Cp | 4464 | 4475 | TTTA | 3 |  |  |
| cP0000006 | PotentialSSR | PentaSSR-Cp | 4487 | 4496 | ATTTA | 2 |  |  |
| cP0000007 | PotentialSSR | PentaSSR-Cp | 4572 | 4581 | ATATA | 2 |  |  |
| cT0000001 | SSR | TriSSR-Cp | 4613 | 4624 | ATA | 4 |  |  |
| cP0000008 | PotentialSSR | PentaSSR-Cp | 4970 | 4979 | TAGAT | 2 | rps16 | (Intron)YP_009380196.1 [rps16]\| |
| cP0000009 | PotentialSSR | PentaSSR-Cp | 5204 | 5213 | CATTT | 2 | rps16 | (Intron)YP_009380196.1 [rps16]\| |
| cH0000007 | PotentialSSR | HexaSSR-Cp | 5274 | 5285 | ATCCAA | 2 | rps16 | (Intron)YP_009380196.1 [rps16]\| |
| c70000004 | ExtendedSSR | 7SSR-Cp | 5294 | 5307 | ACAATTA | 2 | rps16 | (Intron)YP_009380196.1 [rps16]\| |
| cM0000002 | SSR | MonoSSR-Cp | 5341 | 5350 | T | 10 | rps16 | (Intron)YP_009380196.1 [rps16]\| |
| cP0000010 | PotentialSSR | PentaSSR-Cp | 5440 | 5449 | AGAAT | 2 | rps16 | (Intron)YP_009380196.1 [rps16]\| |
| cP0000011 | PotentialSSR | PentaSSR-Cp | 5996 | 6005 | AATGC | 2 |  |  |
| cP0000012 | SSR | PentaSSR-Cp | 6079 | 6093 | TTAAT | 3 |  |  |
| cP0000013 | PotentialSSR | PentaSSR-Cp | 6189 | 6198 | TATAT | 2 |  |  |
| cH0000009 | PotentialSSR | HexaSSR-Cp | 6466 | 6477 | GCTCTG | 2 |  |  |
| cP0000014 | PotentialSSR | PentaSSR-Cp | 6554 | 6563 | TTCTA | 2 |  |  |
| cP0000015 | PotentialSSR | PentaSSR-Cp | 6628 | 6637 | GATTC | 2 |  |  |
| cM0000003 | SSR | MonoSSR-Cp | 6786 | 6796 | A | 11 |  |  |
| cM0000004 | SSR | MonoSSR-Cp | 6852 | 6861 | A | 10 |  |  |
| c80000001 | ExtendedSSR | 8SSR-Cp | 7099 | 7114 | ATTCTAAT | 2 |  |  |
| cH0000010 | PotentialSSR | HexaSSR-Cp | 7344 | 7355 | TTACAA | 2 |  |  |
| cP0000016 | PotentialSSR | PentaSSR-Cp | 7397 | 7406 | TCAAA | 2 |  |  |
| cP0000017 | PotentialSSR | PentaSSR-Cp | 7607 | 7616 | TTCTT | 2 |  |  |
| cP0000018 | PotentialSSR | PentaSSR-Cp | 7639 | 7648 | ATTCT | 2 |  |  |
| cM0000005 | SSR | MonoSSR-Cp | 7728 | 7738 | A | 11 |  |  |
| cP0000019 | PotentialSSR | PentaSSR-Cp | 7894 | 7903 | GAAAA | 2 |  |  |
| cP0000020 | PotentialSSR | PentaSSR-Cp | 7984 | 7993 | ATATA | 2 |  |  |
| cH0000012 | PotentialSSR | HexaSSR-Cp | 8064 | 8075 | AGATAA | 2 |  |  |
| cP0000021 | PotentialSSR | PentaSSR-Cp | 8132 | 8141 | TAAAA | 2 |  |  |
| cP0000022 | PotentialSSR | PentaSSR-Cp | 8208 | 8217 | AAAGA | 2 |  |  |
| cP0000023 | PotentialSSR | PentaSSR-Cp | 8263 | 8272 | GGCCT | 2 |  |  |
| cH0000013 | PotentialSSR | HexaSSR-Cp | 8341 | 8352 | TTTGAT | 2 |  |  |
| cP0000024 | PotentialSSR | PentaSSR-Cp | 8857 | 8866 | AATTG | 2 | trnG-UCC | (Intron)trnG-UCC [trnG-UCC]\| |
| cP0000025 | PotentialSSR | PentaSSR-Cp | 9222 | 9231 | TTTTG | 2 | trnG-UCC | (Intron)trnG-UCC [trnG-UCC]\| |
| cP0000026 | PotentialSSR | PentaSSR-Cp | 9247 | 9256 | CTTAA | 2 | trnG-UCC | (Intron)trnG-UCC [trnG-UCC]\| |
| cP0000027 | PotentialSSR | PentaSSR-Cp | 9280 | 9289 | TTTTC | 2 | trnG-UCC | (Intron)trnG-UCC [trnG-UCC]\| |
| cP0000028 | PotentialSSR | PentaSSR-Cp | 9478 | 9487 | TCTCA | 2 |  |  |
| cP0000029 | PotentialSSR | PentaSSR-Cp | 9500 | 9509 | CAAAA | 2 |  |  |
| cM0000006 | SSR | MonoSSR-Cp | 9526 | 9536 | A | 11 |  |  |
| cH0000016 | PotentialSSR | HexaSSR-Cp | 10442 | 10453 | GCTTGT | 2 | atpA | YP_009380199.1 [atpA]\| |
| cH0000017 | PotentialSSR | HexaSSR-Cp | 10845 | 10856 | CGGGAG | 2 | atpA | YP_009380199.1 [atpA]\| |
| c80000002 | ExtendedSSR | 8SSR-Cp | 11966 | 11981 | AAAAATAG | 2 | atpF | (Intron)YP_009380200.1 [atpF]\| |
| c70000008 | ExtendedSSR | 7SSR-Cp | 12208 | 12221 | TCGGTAT | 2 | atpF | (Intron)YP_009380200.1 [atpF]\| |
| cT0000002 | SSR | TriSSR-Cp | 12314 | 12325 | ATT | 4 | atpF | (Intron)YP_009380200.1 [atpF]\| |
| cTe0000002 | SSR | TetraSSR-Cp | 12879 | 12890 | GGAA | 3 |  |  |
| cP0000030 | PotentialSSR | PentaSSR-Cp | 13027 | 13036 | AGAAA | 2 |  |  |
| cP0000031 | PotentialSSR | PentaSSR-Cp | 13052 | 13061 | TTTCT | 2 |  |  |
| cD0000001 | SSR | DiSSR-Cp | 13323 | 13332 | CA | 5 |  |  |
| cP0000032 | PotentialSSR | PentaSSR-Cp | 13814 | 13823 | AAATA | 2 |  |  |
| cD0000002 | SSR | DiSSR-Cp | 13881 | 13890 | AT | 5 |  |  |
| cH0000018 | PotentialSSR | HexaSSR-Cp | 14254 | 14265 | TAAAGC | 2 | atpI | YP_009380202.1 [atpI]\| |
| cM0000007 | SSR | MonoSSR-Cp | 14706 | 14715 | A | 10 |  |  |
| cH0000019 | PotentialSSR | HexaSSR-Cp | 14754 | 14765 | TTTAAT | 2 |  |  |
| cP0000033 | PotentialSSR | PentaSSR-Cp | 15624 | 15633 | ATTAA | 2 |  |  |
| c80000003 | ExtendedSSR | 8SSR-Cp | 15683 | 15698 | TCTACCGC | 2 |  |  |
| cP0000034 | PotentialSSR | PentaSSR-Cp | 15762 | 15771 | TTTAT | 2 |  |  |
| cP0000035 | PotentialSSR | PentaSSR-Cp | 15775 | 15784 | TAAAT | 2 |  |  |
| cP0000036 | PotentialSSR | PentaSSR-Cp | 16839 | 16848 | CAATT | 2 | rpoC2 | YP_009380204.1 [rpoC2]\| |
| cP0000037 | PotentialSSR | PentaSSR-Cp | 17654 | 17663 | CAAAA | 2 | rpoC2 | YP_009380204.1 [rpoC2]\| |
| cP0000038 | PotentialSSR | PentaSSR-Cp | 17774 | 17783 | TATCT | 2 | rpoC2 | YP_009380204.1 [rpoC2]\| |
| cM0000008 | SSR | MonoSSR-Cp | 17830 | 17842 | T | 13 | rpoC2 | YP_009380204.1 [rpoC2]\| |
| cP0000039 | PotentialSSR | PentaSSR-Cp | 18313 | 18322 | CGATT | 2 | rpoC2 | YP_009380204.1 [rpoC2]\| |
| cP0000040 | PotentialSSR | PentaSSR-Cp | 18342 | 18351 | ATCCT | 2 | rpoC2 | YP_009380204.1 [rpoC2]\| |
| cH0000020 | PotentialSSR | HexaSSR-Cp | 18455 | 18466 | TTGATC | 2 | rpoC2 | YP_009380204.1 [rpoC2]\| |
| cH0000021 | PotentialSSR | HexaSSR-Cp | 18827 | 18838 | ACGTGT | 2 | rpoC2 | YP_009380204.1 [rpoC2]\| |
| cP0000041 | PotentialSSR | PentaSSR-Cp | 19039 | 19048 | CATAA | 2 | rpoC2 | YP_009380204.1 [rpoC2]\| |
| cD0000003 | SSR | DiSSR-Cp | 19182 | 19191 | AT | 5 | rpoC2 | YP_009380204.1 [rpoC2]\| |
| cH0000022 | PotentialSSR | HexaSSR-Cp | 21521 | 21532 | CAAATC | 2 | rpoC1 | YP_009380205.1 [rpoC1]\| |
| cP0000042 | PotentialSSR | PentaSSR-Cp | 21635 | 21644 | GGATT | 2 | rpoC1 | YP_009380205.1 [rpoC1]\| |
| cP0000043 | PotentialSSR | PentaSSR-Cp | 21724 | 21733 | ATCCT | 2 | rpoC1 | (Intron)YP_009380205.1 [rpoC1]\| |
| cP0000044 | PotentialSSR | PentaSSR-Cp | 21918 | 21927 | TTCTT | 2 | rpoC1 | (Intron)YP_009380205.1 [rpoC1]\| |
| cP0000045 | PotentialSSR | PentaSSR-Cp | 21950 | 21959 | AATTA | 2 | rpoC1 | (Intron)YP_009380205.1 [rpoC1]\| |
| cP0000046 | PotentialSSR | PentaSSR-Cp | 22134 | 22143 | TTAAA | 2 | rpoC1 | (Intron)YP_009380205.1 [rpoC1]\| |
| cH0000023 | PotentialSSR | HexaSSR-Cp | 22239 | 22250 | ACAAAA | 2 | rpoC1 | (Intron)YP_009380205.1 [rpoC1]\| |
| cM0000009 | SSR | MonoSSR-Cp | 25507 | 25516 | T | 10 | rpoB | YP_009380206.1 [rpoB]\| |
| cH0000024 | PotentialSSR | HexaSSR-Cp | 25950 | 25961 | TCTTTT | 2 | rpoB | YP_009380206.1 [rpoB]\| |
| cP0000047 | PotentialSSR | PentaSSR-Cp | 26429 | 26438 | ACTTA | 2 |  |  |
| cP0000048 | PotentialSSR | PentaSSR-Cp | 26722 | 26731 | TTGTA | 2 |  |  |
| cH0000026 | PotentialSSR | HexaSSR-Cp | 26883 | 26894 | AGAAAA | 2 |  |  |
| cP0000049 | PotentialSSR | PentaSSR-Cp | 27099 | 27108 | GAATC | 2 |  |  |
| cP0000050 | PotentialSSR | PentaSSR-Cp | 27559 | 27568 | TTCCC | 2 |  |  |
| cP0000051 | PotentialSSR | PentaSSR-Cp | 27688 | 27697 | CTGAG | 2 |  |  |
| cH0000027 | PotentialSSR | HexaSSR-Cp | 27800 | 27811 | TTCATA | 2 |  |  |
| cP0000052 | PotentialSSR | PentaSSR-Cp | 27932 | 27941 | TTTAG | 2 |  |  |
| cH0000028 | PotentialSSR | HexaSSR-Cp | 27958 | 27969 | TATCAT | 2 |  |  |
| cP0000053 | PotentialSSR | PentaSSR-Cp | 28088 | 28097 | GAATT | 2 |  |  |
| cP0000054 | PotentialSSR | PentaSSR-Cp | 28131 | 28140 | AATTC | 2 |  |  |
| cP0000055 | PotentialSSR | PentaSSR-Cp | 28191 | 28200 | ATTTC | 2 |  |  |
| cP0000056 | PotentialSSR | PentaSSR-Cp | 28259 | 28268 | TCAAT | 2 |  |  |
| cP0000057 | PotentialSSR | PentaSSR-Cp | 28285 | 28294 | TTGGC | 2 |  |  |
| cP0000058 | PotentialSSR | PentaSSR-Cp | 28343 | 28352 | ACTTT | 2 |  |  |
| c70000009 | ExtendedSSR | 7SSR-Cp | 28410 | 28423 | TTATAGT | 2 |  |  |
| cP0000059 | PotentialSSR | PentaSSR-Cp | 28447 | 28456 | CTAAT | 2 |  |  |
| cP0000060 | PotentialSSR | PentaSSR-Cp | 28823 | 28832 | TATCA | 2 |  |  |
| cH0000029 | PotentialSSR | HexaSSR-Cp | 29048 | 29059 | CAAAAA | 2 |  |  |
| cM0000010 | SSR | MonoSSR-Cp | 29374 | 29383 | A | 10 |  |  |
| cP0000061 | PotentialSSR | PentaSSR-Cp | 29752 | 29761 | ATAAA | 2 |  |  |
| c80000004 | ExtendedSSR | 8SSR-Cp | 29771 | 29786 | TTTTCTTT | 2 |  |  |
| cP0000062 | PotentialSSR | PentaSSR-Cp | 29828 | 29837 | ACCAT | 2 |  |  |
| cH0000030 | PotentialSSR | HexaSSR-Cp | 29868 | 29879 | AATTTC | 2 |  |  |
| cTe0000003 | SSR | TetraSSR-Cp | 29887 | 29898 | AAAT | 3 |  |  |
| cP0000063 | PotentialSSR | PentaSSR-Cp | 30106 | 30115 | TCAAT | 2 |  |  |
| cP0000064 | PotentialSSR | PentaSSR-Cp | 30157 | 30166 | TACCC | 2 | trnE-UUC | trnE-UUC [trnE-UUC]\| |
| cH0000031 | PotentialSSR | HexaSSR-Cp | 30361 | 30372 | ATTTCA | 2 |  |  |
| cH0000032 | PotentialSSR | HexaSSR-Cp | 30499 | 30510 | CTAATA | 2 |  |  |
| cP0000065 | PotentialSSR | PentaSSR-Cp | 30643 | 30652 | GTATA | 2 |  |  |
| cP0000066 | PotentialSSR | PentaSSR-Cp | 30955 | 30964 | ATTAT | 2 |  |  |
| cP0000067 | PotentialSSR | PentaSSR-Cp | 31028 | 31037 | TGGAA | 2 |  |  |
| c70000010 | ExtendedSSR | 7SSR-Cp | 31489 | 31502 | GTTTATT | 2 |  |  |
| cH0000033 | PotentialSSR | HexaSSR-Cp | 31566 | 31577 | GTGAAA | 2 |  |  |
| cP0000068 | PotentialSSR | PentaSSR-Cp | 31742 | 31751 | AAAGA | 2 |  |  |
| cP0000069 | PotentialSSR | PentaSSR-Cp | 31949 | 31958 | TAAAT | 2 |  |  |
| cP0000070 | PotentialSSR | PentaSSR-Cp | 32112 | 32121 | CGTTT | 2 | psbD | YP_009380209.1 [psbD]\| |
| cP0000071 | PotentialSSR | PentaSSR-Cp | 32742 | 32751 | AACCC | 2 | psbD | YP_009380209.1 [psbD]\| |
| cP0000072 | PotentialSSR | PentaSSR-Cp | 33835 | 33844 | GTCTG | 2 | psbC | YP_009380210.1 [psbC]\| |
| cH0000034 | PotentialSSR | HexaSSR-Cp | 33971 | 33982 | CTCAAG | 2 | psbC | YP_009380210.1 [psbC]\| |
| cP0000073 | PotentialSSR | PentaSSR-Cp | 34263 | 34272 | GGGTG | 2 | psbC | YP_009380210.1 [psbC]\| |
| cH0000035 | PotentialSSR | HexaSSR-Cp | 34389 | 34400 | TGCAGC | 2 | psbC | YP_009380210.1 [psbC]\| |
| c70000011 | ExtendedSSR | 7SSR-Cp | 34512 | 34525 | TACATAT | 2 |  |  |
| cP0000074 | PotentialSSR | PentaSSR-Cp | 34590 | 34599 | TAATT | 2 |  |  |
| cH0000036 | PotentialSSR | HexaSSR-Cp | 34821 | 34832 | TTATTC | 2 |  |  |
| cP0000075 | PotentialSSR | PentaSSR-Cp | 34923 | 34932 | TATAT | 2 |  |  |
| c70000012 | ExtendedSSR | 7SSR-Cp | 34963 | 34976 | TTAATTA | 2 |  |  |
| cP0000076 | PotentialSSR | PentaSSR-Cp | 35299 | 35308 | TGGAT | 2 | psbZ | YP_009380211.1 [psbZ]\| |
| cP0000077 | PotentialSSR | PentaSSR-Cp | 35467 | 35476 | AAACA | 2 |  |  |
| cP0000078 | PotentialSSR | PentaSSR-Cp | 35630 | 35639 | TATAT | 2 |  |  |
| cH0000037 | PotentialSSR | HexaSSR-Cp | 35796 | 35807 | GTTATA | 2 |  |  |
| cP0000079 | PotentialSSR | PentaSSR-Cp | 35819 | 35828 | TAGTG | 2 |  |  |
| cP0000080 | PotentialSSR | PentaSSR-Cp | 35922 | 35931 | TATTT | 2 |  |  |
| cP0000081 | PotentialSSR | PentaSSR-Cp | 36833 | 36842 | CCACG | 2 | psaB | YP_009380213.1 [psaB]\| |
| cP0000082 | PotentialSSR | PentaSSR-Cp | 36982 | 36991 | CCATC | 2 | psaB | YP_009380213.1 [psaB]\| |
| cP0000083 | PotentialSSR | PentaSSR-Cp | 38261 | 38270 | TGTCC | 2 | psaB | YP_009380213.1 [psaB]\| |
| cP0000084 | PotentialSSR | PentaSSR-Cp | 38767 | 38776 | ACCAA | 2 | psaB | YP_009380213.1 [psaB]\| |
| cH0000038 | PotentialSSR | HexaSSR-Cp | 39193 | 39204 | TAATAG | 2 | psaA | YP_009380214.1 [psaA]\| |
| cP0000085 | PotentialSSR | PentaSSR-Cp | 39942 | 39951 | ATGTG | 2 | psaA | YP_009380214.1 [psaA]\| |
| cP0000086 | PotentialSSR | PentaSSR-Cp | 41579 | 41588 | TATTT | 2 |  |  |
| cH0000039 | PotentialSSR | HexaSSR-Cp | 41757 | 41768 | TCTTTA | 2 |  |  |
| cP0000087 | PotentialSSR | PentaSSR-Cp | 41851 | 41860 | TTTTA | 2 |  |  |
| cM0000011 | SSR | MonoSSR-Cp | 41890 | 41899 | A | 10 |  |  |
| cP0000088 | PotentialSSR | PentaSSR-Cp | 42432 | 42441 | AAAGA | 2 | ycf3 | (Intron)YP_009380215.1 [ycf3]\| |
| cP0000089 | PotentialSSR | PentaSSR-Cp | 42569 | 42578 | TTCTT | 2 | ycf3 | (Intron)YP_009380215.1 [ycf3]\| |
| cM0000012 | SSR | MonoSSR-Cp | 42642 | 42651 | T | 10 | ycf3 | (Intron)YP_009380215.1 [ycf3]\| |
| cP0000090 | PotentialSSR | PentaSSR-Cp | 43143 | 43152 | AATAT | 2 | ycf3 | (Intron)YP_009380215.1 [ycf3]\| |
| cP0000091 | PotentialSSR | PentaSSR-Cp | 43818 | 43827 | AAATC | 2 | ycf3 | (Intron)YP_009380215.1 [ycf3]\| |
| cP0000092 | PotentialSSR | PentaSSR-Cp | 44027 | 44036 | GTACA | 2 |  |  |
| cP0000093 | PotentialSSR | PentaSSR-Cp | 44116 | 44125 | TATTT | 2 |  |  |
| cP0000094 | PotentialSSR | PentaSSR-Cp | 44203 | 44212 | CAAAT | 2 |  |  |
| cP0000095 | PotentialSSR | PentaSSR-Cp | 44217 | 44226 | TAGTA | 2 |  |  |
| cP0000096 | PotentialSSR | PentaSSR-Cp | 44270 | 44279 | GATCA | 2 |  |  |
| cP0000097 | PotentialSSR | PentaSSR-Cp | 44339 | 44348 | AATAG | 2 |  |  |
| cP0000098 | PotentialSSR | PentaSSR-Cp | 44516 | 44525 | GATTC | 2 |  |  |
| cTe0000004 | SSR | TetraSSR-Cp | 44531 | 44542 | AATT | 3 |  |  |
| cP0000099 | PotentialSSR | PentaSSR-Cp | 44551 | 44560 | TATAT | 2 |  |  |
| cD0000004 | SSR | DiSSR-Cp | 44623 | 44632 | AT | 5 |  |  |
| cP0000100 | PotentialSSR | PentaSSR-Cp | 44651 | 44660 | TTTTA | 2 |  |  |
| cP0000101 | PotentialSSR | PentaSSR-Cp | 44702 | 44711 | ATTAC | 2 |  |  |
| cP0000102 | PotentialSSR | PentaSSR-Cp | 44750 | 44759 | ATAGT | 2 |  |  |
| cP0000103 | PotentialSSR | PentaSSR-Cp | 44835 | 44844 | ATAGT | 2 |  |  |
| cP0000104 | PotentialSSR | PentaSSR-Cp | 45023 | 45032 | CAAAT | 2 |  |  |
| cP0000105 | PotentialSSR | PentaSSR-Cp | 45199 | 45208 | GGGAT | 2 |  |  |
| cH0000040 | PotentialSSR | HexaSSR-Cp | 45266 | 45277 | TTTTCC | 2 |  |  |
| cH0000041 | PotentialSSR | HexaSSR-Cp | 45281 | 45292 | TGAAAG | 2 |  |  |
| cP0000106 | PotentialSSR | PentaSSR-Cp | 45698 | 45707 | ACCTG | 2 | rps4 | YP_009380216.1 [rps4]\| |
| cD0000005 | SSR | DiSSR-Cp | 45981 | 45994 | TA | 7 |  |  |
| cP0000107 | PotentialSSR | PentaSSR-Cp | 46039 | 46048 | AAATG | 2 |  |  |
| cP0000108 | PotentialSSR | PentaSSR-Cp | 46130 | 46139 | TATTT | 2 |  |  |
| cH0000042 | PotentialSSR | HexaSSR-Cp | 46159 | 46170 | AAAAAG | 2 |  |  |
| cP0000109 | PotentialSSR | PentaSSR-Cp | 46214 | 46223 | CTTTT | 2 |  |  |
| cP0000110 | PotentialSSR | PentaSSR-Cp | 46506 | 46515 | TCAAA | 2 |  |  |
| cP0000111 | PotentialSSR | PentaSSR-Cp | 46584 | 46593 | CGATT | 2 |  |  |
| cP0000112 | PotentialSSR | PentaSSR-Cp | 46820 | 46829 | AATAA | 2 |  |  |
| cP0000113 | PotentialSSR | PentaSSR-Cp | 47409 | 47418 | CTGTG | 2 | trnL-UAA | (Intron)trnL-UAA [trnL-UAA]\| |
| cP0000114 | PotentialSSR | PentaSSR-Cp | 47502 | 47511 | TCTAT | 2 | trnL-UAA | (Intron)trnL-UAA [trnL-UAA]\| |
| cP0000115 | PotentialSSR | PentaSSR-Cp | 47893 | 47902 | TTTTC | 2 |  |  |
| cD0000006 | SSR | DiSSR-Cp | 47950 | 47959 | AT | 5 |  |  |
| cP0000116 | PotentialSSR | PentaSSR-Cp | 48264 | 48273 | CACAT | 2 |  |  |
| cH0000044 | PotentialSSR | HexaSSR-Cp | 48393 | 48404 | GAAAAT | 2 |  |  |
| cP0000117 | PotentialSSR | PentaSSR-Cp | 48524 | 48533 | CTTAA | 2 |  |  |
| cP0000118 | PotentialSSR | PentaSSR-Cp | 48543 | 48552 | TCTTT | 2 |  |  |
| cH0000045 | PotentialSSR | HexaSSR-Cp | 49012 | 49023 | CATATA | 2 | ndhJ | YP_009380217.1 [ndhJ]\| |
| c80000006 | ExtendedSSR | 8SSR-Cp | 49169 | 49184 | ATTTGTTT | 2 |  |  |
| cP0000119 | PotentialSSR | PentaSSR-Cp | 49192 | 49201 | TATTT | 2 |  |  |
| cP0000120 | PotentialSSR | PentaSSR-Cp | 49205 | 49214 | TTTAC | 2 |  |  |
| cH0000046 | PotentialSSR | HexaSSR-Cp | 49972 | 49983 | TTTTTC | 2 |  |  |
| cP0000121 | PotentialSSR | PentaSSR-Cp | 50037 | 50046 | TAAAC | 2 | ndhC | YP_009380219.1 [ndhC]\| |
| cP0000122 | PotentialSSR | PentaSSR-Cp | 50599 | 50608 | TTCTT | 2 |  |  |
| cP0000123 | PotentialSSR | PentaSSR-Cp | 50675 | 50684 | AATTC | 2 |  |  |
| cP0000124 | PotentialSSR | PentaSSR-Cp | 50731 | 50740 | TTCTA | 2 |  |  |
| cP0000125 | PotentialSSR | PentaSSR-Cp | 50790 | 50799 | ACTTG | 2 |  |  |
| c70000015 | ExtendedSSR | 7SSR-Cp | 52547 | 52560 | AATTTTT | 2 |  |  |
| cP0000126 | PotentialSSR | PentaSSR-Cp | 52685 | 52694 | ATTTG | 2 |  |  |
| cP0000127 | PotentialSSR | PentaSSR-Cp | 52786 | 52795 | TTGAA | 2 |  |  |
| cH0000048 | PotentialSSR | HexaSSR-Cp | 52839 | 52850 | TGTATA | 2 |  |  |
| cP0000128 | PotentialSSR | PentaSSR-Cp | 53066 | 53075 | ATTGA | 2 |  |  |
| cP0000129 | PotentialSSR | PentaSSR-Cp | 53105 | 53114 | AATTG | 2 |  |  |
| cM0000013 | SSR | MonoSSR-Cp | 53196 | 53207 | T | 12 |  |  |
| cH0000049 | PotentialSSR | HexaSSR-Cp | 53218 | 53229 | ATTTTC | 2 |  |  |
| cM0000014 | SSR | MonoSSR-Cp | 53303 | 53312 | A | 10 | atpB | YP_009380221.1 [atpB]\| |
| cP0000130 | PotentialSSR | PentaSSR-Cp | 54479 | 54488 | TTGGA | 2 | atpB | YP_009380221.1 [atpB]\| |
| cH0000050 | PotentialSSR | HexaSSR-Cp | 55176 | 55187 | ATAATC | 2 |  |  |
| cH0000051 | PotentialSSR | HexaSSR-Cp | 55246 | 55257 | TCTTTT | 2 |  |  |
| cH0000052 | PotentialSSR | HexaSSR-Cp | 55284 | 55295 | AAAAAG | 2 |  |  |
| cP0000131 | PotentialSSR | PentaSSR-Cp | 55758 | 55767 | AAACA | 2 | trnV-UAC | (Intron)trnV-UAC [trnV-UAC]\| |
| c70000016 | ExtendedSSR | 7SSR-Cp | 56090 | 56103 | AATGAAT | 2 | trnV-UAC | (Intron)trnV-UAC [trnV-UAC]\| |
| cH0000053 | PotentialSSR | HexaSSR-Cp | 56336 | 56347 | CAATTG | 2 |  |  |
| cP0000132 | PotentialSSR | PentaSSR-Cp | 56397 | 56406 | ATTTG | 2 |  |  |
| cH0000054 | PotentialSSR | HexaSSR-Cp | 56421 | 56432 | TTTGAA | 2 |  |  |
| cP0000133 | PotentialSSR | PentaSSR-Cp | 56520 | 56529 | TTTTC | 2 |  |  |
| cP0000134 | PotentialSSR | PentaSSR-Cp | 58586 | 58595 | TATAT | 2 |  |  |
| cP0000135 | PotentialSSR | PentaSSR-Cp | 58642 | 58651 | CTATA | 2 |  |  |
| cTe0000005 | SSR | TetraSSR-Cp | 58669 | 58680 | TAAT | 3 |  |  |
| cP0000136 | PotentialSSR | PentaSSR-Cp | 58683 | 58692 | TAATA | 2 |  |  |
| cH0000055 | PotentialSSR | HexaSSR-Cp | 58724 | 58735 | TACAAT | 2 |  |  |
| cP0000137 | PotentialSSR | PentaSSR-Cp | 58854 | 58863 | AAAAT | 2 | psaI | YP_009380224.1 [psaI]\| |
| cP0000138 | PotentialSSR | PentaSSR-Cp | 58872 | 58881 | TCGTC | 2 |  |  |
| cP0000139 | PotentialSSR | PentaSSR-Cp | 59012 | 59021 | ACAAT | 2 |  |  |
| cP0000140 | PotentialSSR | PentaSSR-Cp | 59176 | 59185 | TTCAA | 2 |  |  |
| cP0000141 | PotentialSSR | PentaSSR-Cp | 59486 | 59495 | TCGAT | 2 | ycf4 | YP_009380225.1 [ycf4]\| |
| cP0000142 | PotentialSSR | PentaSSR-Cp | 59591 | 59600 | TAGAA | 2 | ycf4 | YP_009380225.1 [ycf4]\| |
| cH0000056 | PotentialSSR | HexaSSR-Cp | 59924 | 59935 | TACAAA | 2 |  |  |
| cP0000143 | PotentialSSR | PentaSSR-Cp | 59937 | 59946 | CAATT | 2 |  |  |
| cP0000144 | PotentialSSR | PentaSSR-Cp | 60077 | 60086 | AATTA | 2 |  |  |
| cP0000145 | PotentialSSR | PentaSSR-Cp | 61042 | 61051 | AAAAG | 2 |  |  |
| c70000017 | ExtendedSSR | 7SSR-Cp | 61106 | 61119 | ATAATCA | 2 |  |  |
| cP0000146 | PotentialSSR | PentaSSR-Cp | 61191 | 61200 | TTATT | 2 |  |  |
| cP0000147 | PotentialSSR | PentaSSR-Cp | 61689 | 61698 | AAAGA | 2 | petA | YP_009380227.1 [petA]\| |
| cP0000148 | PotentialSSR | PentaSSR-Cp | 61960 | 61969 | GAAAA | 2 | petA | YP_009380227.1 [petA]\| |
| cH0000057 | PotentialSSR | HexaSSR-Cp | 62280 | 62291 | ATCAAG | 2 |  |  |
| cH0000058 | PotentialSSR | HexaSSR-Cp | 62296 | 62307 | TAACAA | 2 |  |  |
| cP0000149 | PotentialSSR | PentaSSR-Cp | 62320 | 62329 | CAATT | 2 |  |  |
| cM0000015 | SSR | MonoSSR-Cp | 62354 | 62367 | A | 14 |  |  |
| c80000007 | ExtendedSSR | 8SSR-Cp | 62461 | 62476 | ACTTTTTT | 2 |  |  |
| cH0000059 | PotentialSSR | HexaSSR-Cp | 62668 | 62679 | ATTTTT | 2 |  |  |
| cH0000060 | PotentialSSR | HexaSSR-Cp | 62863 | 62874 | TCTTTC | 2 |  |  |
| cP0000150 | PotentialSSR | PentaSSR-Cp | 62877 | 62886 | TTAAC | 2 |  |  |
| c70000018 | ExtendedSSR | 7SSR-Cp | 63129 | 63142 | TTACTAC | 2 | psbJ | YP_009380228.1 [psbJ]\| |
| cP0000151 | PotentialSSR | PentaSSR-Cp | 63482 | 63491 | ATTCA | 2 | psbL | YP_009380229.1 [psbL]\| |
| cH0000061 | PotentialSSR | HexaSSR-Cp | 63533 | 63544 | ATTCGG | 2 |  |  |
| c80000008 | ExtendedSSR | 8SSR-Cp | 64013 | 64028 | ACGTAAAA | 2 |  |  |
| cH0000062 | PotentialSSR | HexaSSR-Cp | 64349 | 64360 | TATAGA | 2 |  |  |
| cH0000063 | PotentialSSR | HexaSSR-Cp | 64588 | 64599 | AGTCTT | 2 |  |  |
| cP0000152 | PotentialSSR | PentaSSR-Cp | 65144 | 65153 | CTGTA | 2 |  |  |
| cM0000016 | SSR | MonoSSR-Cp | 65259 | 65271 | T | 13 |  |  |
| cP0000153 | PotentialSSR | PentaSSR-Cp | 65463 | 65472 | GATTA | 2 |  |  |
| cP0000154 | PotentialSSR | PentaSSR-Cp | 65480 | 65489 | TTTAG | 2 |  |  |
| cH0000064 | PotentialSSR | HexaSSR-Cp | 65499 | 65510 | TTTCTA | 2 |  |  |
| cP0000155 | PotentialSSR | PentaSSR-Cp | 65597 | 65606 | GAACT | 2 | trnW-CCA | trnW-CCA [trnW-CCA]\| |
| cH0000065 | PotentialSSR | HexaSSR-Cp | 65673 | 65684 | CTATAT | 2 |  |  |
| cP0000156 | PotentialSSR | PentaSSR-Cp | 65880 | 65889 | TTCAA | 2 |  |  |
| cP0000157 | PotentialSSR | PentaSSR-Cp | 65919 | 65928 | CCTTG | 2 |  |  |
| cP0000158 | PotentialSSR | PentaSSR-Cp | 66055 | 66064 | GTAAA | 2 |  |  |
| cP0000159 | PotentialSSR | PentaSSR-Cp | 66167 | 66176 | CTTAG | 2 |  |  |
| cP0000160 | PotentialSSR | PentaSSR-Cp | 66523 | 66532 | TTAGT | 2 |  |  |
| cP0000161 | PotentialSSR | PentaSSR-Cp | 66576 | 66585 | GTTAA | 2 |  |  |
| c70000019 | ExtendedSSR | 7SSR-Cp | 66717 | 66730 | CGAATTG | 2 |  |  |
| cH0000066 | PotentialSSR | HexaSSR-Cp | 67080 | 67091 | AATTTT | 2 |  |  |
| cD0000007 | SSR | DiSSR-Cp | 67105 | 67114 | AT | 5 |  |  |
| cP0000162 | PotentialSSR | PentaSSR-Cp | 67174 | 67183 | ATATA | 2 |  |  |
| cP0000163 | PotentialSSR | PentaSSR-Cp | 67249 | 67258 | ATAGG | 2 |  |  |
| cP0000164 | PotentialSSR | PentaSSR-Cp | 67276 | 67285 | TAAAC | 2 |  |  |
| cTe0000006 | SSR | TetraSSR-Cp | 67760 | 67771 | TTTA | 3 |  |  |
| cP0000165 | PotentialSSR | PentaSSR-Cp | 68323 | 68332 | ATAAA | 2 |  |  |
| cH0000067 | PotentialSSR | HexaSSR-Cp | 68814 | 68825 | ATTTTC | 2 |  |  |
| cP0000166 | PotentialSSR | PentaSSR-Cp | 68932 | 68941 | CTATT | 2 |  |  |
| cH0000068 | PotentialSSR | HexaSSR-Cp | 68979 | 68990 | TTTTGT | 2 |  |  |
| cH0000069 | PotentialSSR | HexaSSR-Cp | 69299 | 69310 | TGGGCT | 2 | clpP | YP_009380239.1 [clpP]\| |
| cP0000167 | PotentialSSR | PentaSSR-Cp | 69529 | 69538 | ACACA | 2 | clpP | (Intron)YP_009380239.1 [clpP]\| |
| cP0000168 | PotentialSSR | PentaSSR-Cp | 69674 | 69683 | ATCGA | 2 | clpP | (Intron)YP_009380239.1 [clpP]\| |
| cH0000070 | PotentialSSR | HexaSSR-Cp | 69685 | 69696 | CAGATC | 2 | clpP | (Intron)YP_009380239.1 [clpP]\| |
| cP0000169 | PotentialSSR | PentaSSR-Cp | 69970 | 69979 | GAAAA | 2 | clpP | (Intron)YP_009380239.1 [clpP]\| |
| cH0000071 | PotentialSSR | HexaSSR-Cp | 70260 | 70271 | ACAAAT | 2 | clpP | YP_009380239.1 [clpP]\| |
| cP0000170 | PotentialSSR | PentaSSR-Cp | 70673 | 70682 | TATCA | 2 | clpP | (Intron)YP_009380239.1 [clpP]\| |
| cH0000072 | PotentialSSR | HexaSSR-Cp | 70713 | 70724 | TTCTTG | 2 | clpP | (Intron)YP_009380239.1 [clpP]\| |
| cH0000073 | PotentialSSR | HexaSSR-Cp | 70923 | 70934 | TTGAAA | 2 | clpP | (Intron)YP_009380239.1 [clpP]\| |
| cH0000074 | PotentialSSR | HexaSSR-Cp | 70990 | 71001 | ATTGGG | 2 | clpP | (Intron)YP_009380239.1 [clpP]\| |
| cP0000171 | PotentialSSR | PentaSSR-Cp | 71559 | 71568 | ATAGA | 2 |  |  |
| cH0000075 | PotentialSSR | HexaSSR-Cp | 71678 | 71689 | CATAGT | 2 |  |  |
| c70000021 | ExtendedSSR | 7SSR-Cp | 71828 | 71841 | CTGGTTG | 2 | psbB | YP_009380240.1 [psbB]\| |
| cH0000076 | PotentialSSR | HexaSSR-Cp | 72192 | 72203 | GTTTTG | 2 | psbB | YP_009380240.1 [psbB]\| |
| cP0000172 | PotentialSSR | PentaSSR-Cp | 73298 | 73307 | TTTGA | 2 |  |  |
| cP0000173 | PotentialSSR | PentaSSR-Cp | 73455 | 73464 | CTCTA | 2 | psbT | YP_009380241.1 [psbT]\| |
| cP0000174 | PotentialSSR | PentaSSR-Cp | 73519 | 73528 | AAATG | 2 | psbT | YP_009380241.1 [psbT]\| |
| cH0000077 | PotentialSSR | HexaSSR-Cp | 74050 | 74061 | ACAAAA | 2 |  |  |
| c90000002 | ExtendedSSR | 9SSR-Cp | 74099 | 74116 | CAATACAAA | 2 |  |  |
| cP0000175 | PotentialSSR | PentaSSR-Cp | 74402 | 74411 | TTTTC | 2 | petB | (Intron)YP_009380244.1 [petB]\| |
| cP0000176 | PotentialSSR | PentaSSR-Cp | 74520 | 74529 | AATAA | 2 | petB | (Intron)YP_009380244.1 [petB]\| |
| cP0000177 | PotentialSSR | PentaSSR-Cp | 74532 | 74541 | CCTAT | 2 | petB | (Intron)YP_009380244.1 [petB]\| |
| cH0000078 | PotentialSSR | HexaSSR-Cp | 74708 | 74719 | ATTATA | 2 | petB | (Intron)YP_009380244.1 [petB]\| |
| cH0000079 | PotentialSSR | HexaSSR-Cp | 74720 | 74731 | TACAAA | 2 | petB | (Intron)YP_009380244.1 [petB]\| |
| cP0000178 | PotentialSSR | PentaSSR-Cp | 75662 | 75671 | ATAGA | 2 | petB | YP_009380244.1 [petB]\| |
| cP0000179 | PotentialSSR | PentaSSR-Cp | 76031 | 76040 | TCTAT | 2 | petD | (Intron)YP_009380245.1 [petD]\| |
| cP0000180 | PotentialSSR | PentaSSR-Cp | 76107 | 76116 | ATAAT | 2 | petD | (Intron)YP_009380245.1 [petD]\| |
| cP0000181 | PotentialSSR | PentaSSR-Cp | 76121 | 76130 | ATTAT | 2 | petD | (Intron)YP_009380245.1 [petD]\| |
| cP0000182 | PotentialSSR | PentaSSR-Cp | 76144 | 76153 | TTATT | 2 | petD | (Intron)YP_009380245.1 [petD]\| |
| cP0000183 | PotentialSSR | PentaSSR-Cp | 76616 | 76625 | GAATC | 2 | petD | (Intron)YP_009380245.1 [petD]\| |
| cP0000184 | PotentialSSR | PentaSSR-Cp | 76629 | 76638 | AAGAA | 2 | petD | (Intron)YP_009380245.1 [petD]\| |
| cP0000185 | PotentialSSR | PentaSSR-Cp | 77208 | 77217 | ATTCA | 2 |  |  |
| cM0000017 | SSR | MonoSSR-Cp | 77435 | 77444 | T | 10 | rpoA | YP_009380246.1 [rpoA]\| |
| cH0000080 | PotentialSSR | HexaSSR-Cp | 77654 | 77665 | CATTTC | 2 | rpoA | YP_009380246.1 [rpoA]\| |
| cP0000186 | PotentialSSR | PentaSSR-Cp | 78084 | 78093 | TCGCA | 2 | rpoA | YP_009380246.1 [rpoA]\| |
| cP0000187 | PotentialSSR | PentaSSR-Cp | 78852 | 78861 | TAGTA | 2 |  |  |
| cM0000018 | SSR | MonoSSR-Cp | 78995 | 79005 | T | 11 |  |  |
| cH0000081 | PotentialSSR | HexaSSR-Cp | 79078 | 79089 | GTTGAA | 2 |  |  |
| cP0000188 | PotentialSSR | PentaSSR-Cp | 79910 | 79919 | TTAGT | 2 |  |  |
| cH0000082 | PotentialSSR | HexaSSR-Cp | 79981 | 79992 | TATTTT | 2 |  |  |
| cH0000083 | PotentialSSR | HexaSSR-Cp | 80988 | 80999 | AAAAAT | 2 | rpl16 | (Intron)YP_009380252.1 [rpl16]\| |
| cP0000189 | PotentialSSR | PentaSSR-Cp | 81015 | 81024 | TATTT | 2 | rpl16 | (Intron)YP_009380252.1 [rpl16]\| |
| c70000023 | ExtendedSSR | 7SSR-Cp | 81233 | 81246 | TTTTATA | 2 | rpl16 | (Intron)YP_009380252.1 [rpl16]\| |
| cP0000190 | PotentialSSR | PentaSSR-Cp | 81289 | 81298 | AAAAG | 2 | rpl16 | (Intron)YP_009380252.1 [rpl16]\| |
| cM0000019 | SSR | MonoSSR-Cp | 81575 | 81584 | T | 10 | rpl16 | (Intron)YP_009380252.1 [rpl16]\| |
| cM0000020 | SSR | MonoSSR-Cp | 81708 | 81718 | T | 11 | rpl16 | (Intron)YP_009380252.1 [rpl16]\| |
| cM0000021 | SSR | MonoSSR-Cp | 81767 | 81776 | T | 10 | rpl16 | (Intron)YP_009380252.1 [rpl16]\| |
| cH0000085 | PotentialSSR | HexaSSR-Cp | 81956 | 81967 | TTTTAA | 2 | rpl16 | (Intron)YP_009380252.1 [rpl16]\| |
| cP0000191 | PotentialSSR | PentaSSR-Cp | 82263 | 82272 | ACCCT | 2 | rps3 | YP_009380253.1 [rps3]\| |
| cP0000192 | PotentialSSR | PentaSSR-Cp | 82397 | 82406 | TACTC | 2 | rps3 | YP_009380253.1 [rps3]\| |
| cP0000193 | PotentialSSR | PentaSSR-Cp | 82500 | 82509 | CAATT | 2 | rps3 | YP_009380253.1 [rps3]\| |
| cP0000194 | PotentialSSR | PentaSSR-Cp | 82858 | 82867 | TTTAT | 2 | rpl22 | YP_009380254.1 [rpl22]\| |
| cP0000195 | PotentialSSR | PentaSSR-Cp | 83379 | 83388 | TTTCT | 2 |  |  |
| cH0000086 | PotentialSSR | HexaSSR-Cp | 83808 | 83819 | ATTTTC | 2 |  |  |
| cP0000196 | PotentialSSR | PentaSSR-Cp | 84223 | 84232 | TATGT | 2 | rpl2 | YP_009380256.1 [rpl2]\| |
| cP0000197 | PotentialSSR | PentaSSR-Cp | 84571 | 84580 | TGGAT | 2 | rpl2 | YP_009380256.1 [rpl2]\| |
| cP0000198 | PotentialSSR | PentaSSR-Cp | 84672 | 84681 | TTCTT | 2 |  |  |
| cP0000199 | PotentialSSR | PentaSSR-Cp | 84693 | 84702 | GAATA | 2 |  |  |
| cP0000200 | PotentialSSR | PentaSSR-Cp | 85051 | 85060 | TATGA | 2 |  |  |
| cP0000201 | PotentialSSR | PentaSSR-Cp | 85267 | 85276 | TGAAA | 2 |  |  |
| cH0000087 | PotentialSSR | HexaSSR-Cp | 85933 | 85944 | GAAAGA | 2 | ycf2 | YP_009380257.1 [ycf2]\| |
| cP0000202 | PotentialSSR | PentaSSR-Cp | 85985 | 85994 | GATCC | 2 | ycf2 | YP_009380257.1 [ycf2]\| |
| c90000003 | ExtendedSSR | 9SSR-Cp | 87265 | 87282 | GGAACATTT | 2 | ycf2 | YP_009380257.1 [ycf2]\| |
| cP0000203 | PotentialSSR | PentaSSR-Cp | 88112 | 88121 | CGATC | 2 | ycf2 | YP_009380257.1 [ycf2]\| |
| cP0000204 | PotentialSSR | PentaSSR-Cp | 88277 | 88286 | TTCAA | 2 | ycf2 | YP_009380257.1 [ycf2]\| |
| cH0000089 | PotentialSSR | HexaSSR-Cp | 89054 | 89065 | GGTCCC | 2 | ycf2 | YP_009380257.1 [ycf2]\| |
| cH0000090 | PotentialSSR | HexaSSR-Cp | 89230 | 89241 | AAGAAA | 2 | ycf2 | YP_009380257.1 [ycf2]\| |
| cP0000205 | PotentialSSR | PentaSSR-Cp | 89255 | 89264 | GATTG | 2 | ycf2 | YP_009380257.1 [ycf2]\| |
| cH0000091 | PotentialSSR | HexaSSR-Cp | 89896 | 89907 | GGAGCT | 2 | ycf2 | YP_009380257.1 [ycf2]\| |
| cP0000206 | PotentialSSR | PentaSSR-Cp | 90278 | 90287 | GAAAA | 2 | ycf2 | YP_009380257.1 [ycf2]\| |
| cH0000092 | PotentialSSR | HexaSSR-Cp | 90783 | 90794 | TAGAAG | 2 | ycf2 | YP_009380257.1 [ycf2]\| |
| cH0000093 | PotentialSSR | HexaSSR-Cp | 91383 | 91394 | CTATAT | 2 | ycf2 | YP_009380257.1 [ycf2]\| |
| cP0000207 | PotentialSSR | PentaSSR-Cp | 91877 | 91886 | AAGTT | 2 |  |  |
| cP0000208 | PotentialSSR | PentaSSR-Cp | 91905 | 91914 | TTGTT | 2 |  |  |
| cP0000209 | PotentialSSR | PentaSSR-Cp | 92149 | 92158 | GTTAC | 2 |  |  |
| cH0000094 | PotentialSSR | HexaSSR-Cp | 92324 | 92335 | ATTCCA | 2 |  |  |
| cP0000210 | PotentialSSR | PentaSSR-Cp | 92640 | 92649 | CTTAT | 2 |  |  |
| cP0000211 | PotentialSSR | PentaSSR-Cp | 92775 | 92784 | ATGGA | 2 |  |  |
| cH0000095 | PotentialSSR | HexaSSR-Cp | 93736 | 93747 | GCTGAA | 2 | ndhB | YP_009380258.1 [ndhB]\| |
| cH0000096 | PotentialSSR | HexaSSR-Cp | 93795 | 93806 | AGAGTC | 2 | ndhB | YP_009380258.1 [ndhB]\| |
| cP0000212 | PotentialSSR | PentaSSR-Cp | 94091 | 94100 | TGATT | 2 | ndhB | (Intron)YP_009380258.1 [ndhB]\| |
| cP0000213 | PotentialSSR | PentaSSR-Cp | 94291 | 94300 | AAAGA | 2 | ndhB | (Intron)YP_009380258.1 [ndhB]\| |
| cH0000097 | PotentialSSR | HexaSSR-Cp | 95358 | 95369 | TTCTTA | 2 |  |  |
| cP0000214 | PotentialSSR | PentaSSR-Cp | 95424 | 95433 | AGAAA | 2 |  |  |
| cP0000215 | PotentialSSR | PentaSSR-Cp | 95528 | 95537 | CTGTT | 2 |  |  |
| cH0000098 | PotentialSSR | HexaSSR-Cp | 96275 | 96286 | TCCATA | 2 |  |  |
| cP0000216 | PotentialSSR | PentaSSR-Cp | 96463 | 96472 | CGAAT | 2 |  |  |
| cH0000099 | PotentialSSR | HexaSSR-Cp | 97052 | 97063 | TTGATT | 2 |  |  |
| cH0000100 | PotentialSSR | HexaSSR-Cp | 97099 | 97110 | TTCCTC | 2 |  |  |
| cH0000101 | PotentialSSR | HexaSSR-Cp | 97111 | 97122 | TATCCC | 2 |  |  |
| cP0000217 | PotentialSSR | PentaSSR-Cp | 97357 | 97366 | TGTTG | 2 |  |  |
| cP0000218 | PotentialSSR | PentaSSR-Cp | 97455 | 97464 | TATTA | 2 |  |  |
| cP0000219 | PotentialSSR | PentaSSR-Cp | 97474 | 97483 | ATTAG | 2 |  |  |
| cP0000220 | PotentialSSR | PentaSSR-Cp | 97656 | 97665 | GCAAT | 2 |  |  |
| cH0000102 | PotentialSSR | HexaSSR-Cp | 97776 | 97787 | TATTAC | 2 |  |  |
| cH0000103 | PotentialSSR | HexaSSR-Cp | 97923 | 97934 | AATGGA | 2 |  |  |
| cM0000022 | SSR | MonoSSR-Cp | 98033 | 98044 | T | 12 |  |  |
| cP0000221 | PotentialSSR | PentaSSR-Cp | 98284 | 98293 | CAAGA | 2 |  |  |
| cP0000222 | PotentialSSR | PentaSSR-Cp | 98367 | 98376 | AGGGA | 2 |  |  |
| cH0000104 | PotentialSSR | HexaSSR-Cp | 98491 | 98502 | GAATGA | 2 |  |  |
| cH0000105 | PotentialSSR | HexaSSR-Cp | 99364 | 99375 | GACACT | 2 | rrn16 | rrn16 [rrn16]\| |
| cH0000106 | PotentialSSR | HexaSSR-Cp | 100821 | 100832 | AATGGA | 2 | trnI-GAU | (Intron)trnI-GAU [trnI-GAU]\| |
| cH0000107 | PotentialSSR | HexaSSR-Cp | 101577 | 101588 | AAGAAT | 2 |  |  |
| cP0000223 | PotentialSSR | PentaSSR-Cp | 101808 | 101817 | ACAAA | 2 | trnA-UGC | (Intron)trnA-UGC [trnA-UGC]\| |
| cP0000224 | PotentialSSR | PentaSSR-Cp | 102127 | 102136 | TTCAA | 2 | trnA-UGC | (Intron)trnA-UGC [trnA-UGC]\| |
| c80000010 | ExtendedSSR | 8SSR-Cp | 102351 | 102366 | TTTTGAGA | 2 | trnA-UGC | (Intron)trnA-UGC [trnA-UGC]\| |
| cD0000008 | SSR | DiSSR-Cp | 103926 | 103935 | CG | 5 | rrn23 | rrn23 [rrn23]\| |
| cH0000108 | PotentialSSR | HexaSSR-Cp | 103949 | 103960 | GAAGCG | 2 | rrn23 | rrn23 [rrn23]\| |
| cTe0000007 | SSR | TetraSSR-Cp | 104343 | 104354 | AGGT | 3 | rrn23 | rrn23 [rrn23]\| |
| cP0000225 | PotentialSSR | PentaSSR-Cp | 105436 | 105445 | GCGGA | 2 | rrn23 | rrn23 [rrn23]\| |
| cP0000226 | PotentialSSR | PentaSSR-Cp | 105734 | 105743 | ATCCA | 2 |  |  |
| c70000024 | ExtendedSSR | 7SSR-Cp | 105806 | 105819 | AAAAACC | 2 |  |  |
| cH0000109 | PotentialSSR | HexaSSR-Cp | 105836 | 105847 | TCTATC | 2 |  |  |
| cH0000110 | PotentialSSR | HexaSSR-Cp | 106213 | 106224 | TTCTTA | 2 |  |  |
| cP0000227 | PotentialSSR | PentaSSR-Cp | 106510 | 106519 | AGTGG | 2 |  |  |
| cH0000111 | PotentialSSR | HexaSSR-Cp | 106650 | 106661 | CAAGTA | 2 |  |  |
| cP0000228 | PotentialSSR | PentaSSR-Cp | 106666 | 106675 | TAGCA | 2 |  |  |
| cP0000229 | PotentialSSR | PentaSSR-Cp | 106690 | 106699 | GTCAT | 2 |  |  |
| c70000025 | ExtendedSSR | 7SSR-Cp | 106703 | 106716 | TATGTTT | 2 |  |  |
| cP0000230 | PotentialSSR | PentaSSR-Cp | 106875 | 106884 | CAGAA | 2 |  |  |
| c70000026 | ExtendedSSR | 7SSR-Cp | 106981 | 106994 | AAGAATG | 2 |  |  |
| c90000004 | ExtendedSSR | 9SSR-Cp | 107542 | 107559 | GAAGAAGGA | 2 | ycf1 | YP_009380260.1 [ycf1]\| |
| cH0000113 | PotentialSSR | HexaSSR-Cp | 108164 | 108175 | TAGAAA | 2 | ycf1 | YP_009380260.1 [ycf1]\| |
| cH0000114 | PotentialSSR | HexaSSR-Cp | 108231 | 108242 | TCCTTC | 2 | ycf1 | YP_009380260.1 [ycf1]\| |
| cP0000231 | PotentialSSR | PentaSSR-Cp | 108254 | 108263 | AAGAA | 2 | ycf1 | YP_009380260.1 [ycf1]\| |
| cH0000115 | PotentialSSR | HexaSSR-Cp | 108273 | 108284 | CAAAAT | 2 | ycf1 | YP_009380260.1 [ycf1]\| |
| cP0000232 | PotentialSSR | PentaSSR-Cp | 108320 | 108329 | ACAAA | 2 | ycf1 | YP_009380260.1 [ycf1]\| |
| cP0000233 | PotentialSSR | PentaSSR-Cp | 108545 | 108554 | GAAAT | 2 | ycf1 | YP_009380260.1 [ycf1]\| |
| cM0000023 | SSR | MonoSSR-Cp | 108731 | 108740 | A | 10 | ycf1 | YP_009380260.1 [ycf1]\| |
| cP0000234 | PotentialSSR | PentaSSR-Cp | 108845 | 108854 | AAAAT | 2 | ycf1,ndhF | YP_009380260.1 [ycf1]\|YP_009380261.1 [ndhF]\| |
| cH0000116 | PotentialSSR | HexaSSR-Cp | 110003 | 110014 | AGATCC | 2 | ndhF | YP_009380261.1 [ndhF]\| |
| c70000027 | ExtendedSSR | 7SSR-Cp | 110470 | 110483 | CTCGAAA | 2 | ndhF | YP_009380261.1 [ndhF]\| |
| cP0000235 | PotentialSSR | PentaSSR-Cp | 111096 | 111105 | TAGAA | 2 |  |  |
| c70000028 | ExtendedSSR | 7SSR-Cp | 111269 | 111282 | TTAAAAC | 2 |  |  |
| cP0000236 | PotentialSSR | PentaSSR-Cp | 111525 | 111534 | TTACT | 2 |  |  |
| cM0000024 | SSR | MonoSSR-Cp | 112239 | 112248 | A | 10 |  |  |
| cP0000237 | PotentialSSR | PentaSSR-Cp | 112487 | 112496 | TTTTA | 2 |  |  |
| c70000029 | ExtendedSSR | 7SSR-Cp | 112507 | 112520 | TAAAAGA | 2 |  |  |
| cM0000025 | SSR | MonoSSR-Cp | 112558 | 112567 | A | 10 |  |  |
| cP0000238 | PotentialSSR | PentaSSR-Cp | 112854 | 112863 | AGAAA | 2 |  |  |
| cP0000239 | PotentialSSR | PentaSSR-Cp | 112983 | 112992 | GAAAA | 2 |  |  |
| cH0000117 | PotentialSSR | HexaSSR-Cp | 113169 | 113180 | TATGAA | 2 |  |  |
| cH0000118 | PotentialSSR | HexaSSR-Cp | 113429 | 113440 | AATGAA | 2 |  |  |
| cM0000026 | SSR | MonoSSR-Cp | 113488 | 113497 | T | 10 |  |  |
| cP0000240 | PotentialSSR | PentaSSR-Cp | 114615 | 114624 | TTTTG | 2 |  |  |
| cP0000241 | PotentialSSR | PentaSSR-Cp | 114653 | 114662 | TTTCA | 2 |  |  |
| cH0000119 | PotentialSSR | HexaSSR-Cp | 114751 | 114762 | AATACC | 2 | ndhD | YP_009380264.1 [ndhD]\| |
| cM0000027 | SSR | MonoSSR-Cp | 114817 | 114826 | A | 10 | ndhD | YP_009380264.1 [ndhD]\| |
| cH0000120 | PotentialSSR | HexaSSR-Cp | 115747 | 115758 | TAATTC | 2 | ndhD | YP_009380264.1 [ndhD]\| |
| cH0000121 | PotentialSSR | HexaSSR-Cp | 116599 | 116610 | TCTAGT | 2 |  |  |
| cP0000242 | PotentialSSR | PentaSSR-Cp | 117234 | 117243 | AAGTT | 2 |  |  |
| cP0000243 | PotentialSSR | PentaSSR-Cp | 117534 | 117543 | ATACC | 2 | ndhG | YP_009380267.1 [ndhG]\| |
| c70000030 | ExtendedSSR | 7SSR-Cp | 117746 | 117759 | TAGAATA | 2 | ndhG | YP_009380267.1 [ndhG]\| |
| cP0000244 | PotentialSSR | PentaSSR-Cp | 118021 | 118030 | TAAAG | 2 |  |  |
| cP0000245 | PotentialSSR | PentaSSR-Cp | 118186 | 118195 | TTTAA | 2 |  |  |
| cP0000246 | PotentialSSR | PentaSSR-Cp | 118307 | 118316 | TAATT | 2 | ndhI | YP_009380268.1 [ndhI]\| |
| cH0000122 | PotentialSSR | HexaSSR-Cp | 118949 | 118960 | GAACAA | 2 | ndhA | YP_009380269.1 [ndhA]\| |
| cP0000247 | PotentialSSR | PentaSSR-Cp | 118973 | 118982 | TAATG | 2 | ndhA | YP_009380269.1 [ndhA]\| |
| cP0000248 | PotentialSSR | PentaSSR-Cp | 119126 | 119135 | ATAAA | 2 | ndhA | YP_009380269.1 [ndhA]\| |
| cP0000249 | PotentialSSR | PentaSSR-Cp | 119354 | 119363 | AAGAT | 2 | ndhA | (Intron)YP_009380269.1 [ndhA]\| |
| cP0000250 | PotentialSSR | PentaSSR-Cp | 119542 | 119551 | CTATA | 2 | ndhA | (Intron)YP_009380269.1 [ndhA]\| |
| c70000031 | ExtendedSSR | 7SSR-Cp | 119664 | 119677 | TATCAAT | 2 | ndhA | (Intron)YP_009380269.1 [ndhA]\| |
| cM0000028 | SSR | MonoSSR-Cp | 119793 | 119802 | T | 10 | ndhA | (Intron)YP_009380269.1 [ndhA]\| |
| cH0000123 | PotentialSSR | HexaSSR-Cp | 119807 | 119818 | CTATTA | 2 | ndhA | (Intron)YP_009380269.1 [ndhA]\| |
| cP0000251 | PotentialSSR | PentaSSR-Cp | 121258 | 121267 | CATTC | 2 | ndhH | YP_009380270.1 [ndhH]\| |
| cH0000124 | PotentialSSR | HexaSSR-Cp | 122267 | 122278 | ATAATT | 2 |  |  |
| cP0000252 | PotentialSSR | PentaSSR-Cp | 122672 | 122681 | TTTAT | 2 |  |  |
| cH0000125 | PotentialSSR | HexaSSR-Cp | 122957 | 122968 | AATTTT | 2 | ycf1 | YP_009380272.1 [ycf1]\| |
| cP0000253 | PotentialSSR | PentaSSR-Cp | 123397 | 123406 | TTCTT | 2 | ycf1 | YP_009380272.1 [ycf1]\| |
| cM0000029 | SSR | MonoSSR-Cp | 123976 | 123987 | T | 12 | ycf1 | YP_009380272.1 [ycf1]\| |
| cH0000126 | PotentialSSR | HexaSSR-Cp | 124343 | 124354 | CTATAT | 2 | ycf1 | YP_009380272.1 [ycf1]\| |
| cH0000127 | PotentialSSR | HexaSSR-Cp | 124441 | 124452 | CAATAA | 2 | ycf1 | YP_009380272.1 [ycf1]\| |
| cTe0000008 | SSR | TetraSSR-Cp | 124475 | 124486 | TAAT | 3 | ycf1 | YP_009380272.1 [ycf1]\| |
| cM0000030 | SSR | MonoSSR-Cp | 125521 | 125531 | A | 11 | ycf1 | YP_009380272.1 [ycf1]\| |
| cP0000254 | PotentialSSR | PentaSSR-Cp | 125686 | 125695 | AAAAC | 2 | ycf1 | YP_009380272.1 [ycf1]\| |
| cP0000255 | PotentialSSR | PentaSSR-Cp | 127022 | 127031 | ATTTT | 2 | ycf1 | YP_009380272.1 [ycf1]\| |
| cM0000031 | SSR | MonoSSR-Cp | 127136 | 127145 | T | 10 | ycf1 | YP_009380272.1 [ycf1]\| |
| cP0000256 | PotentialSSR | PentaSSR-Cp | 127321 | 127330 | CATTT | 2 | ycf1 | YP_009380272.1 [ycf1]\| |
| cP0000257 | PotentialSSR | PentaSSR-Cp | 127547 | 127556 | TTTGT | 2 | ycf1 | YP_009380272.1 [ycf1]\| |
| cH0000128 | PotentialSSR | HexaSSR-Cp | 127592 | 127603 | ATTTTG | 2 | ycf1 | YP_009380272.1 [ycf1]\| |
| cP0000258 | PotentialSSR | PentaSSR-Cp | 127613 | 127622 | TTCTT | 2 | ycf1 | YP_009380272.1 [ycf1]\| |
| cH0000129 | PotentialSSR | HexaSSR-Cp | 127634 | 127645 | GAAGGA | 2 | ycf1 | YP_009380272.1 [ycf1]\| |
| cH0000130 | PotentialSSR | HexaSSR-Cp | 127701 | 127712 | TTTCTA | 2 | ycf1 | YP_009380272.1 [ycf1]\| |
| c90000005 | ExtendedSSR | 9SSR-Cp | 128317 | 128334 | TCCTTCTTC | 2 | ycf1 | YP_009380272.1 [ycf1]\| |
| c70000032 | ExtendedSSR | 7SSR-Cp | 128880 | 128893 | TTCATTC | 2 |  |  |
| cP0000259 | PotentialSSR | PentaSSR-Cp | 128992 | 129001 | TTCTG | 2 |  |  |
| c70000033 | ExtendedSSR | 7SSR-Cp | 129160 | 129173 | AAACATA | 2 |  |  |
| cP0000260 | PotentialSSR | PentaSSR-Cp | 129177 | 129186 | ATGAC | 2 |  |  |
| cP0000261 | PotentialSSR | PentaSSR-Cp | 129201 | 129210 | TGCTA | 2 |  |  |
| cH0000132 | PotentialSSR | HexaSSR-Cp | 129215 | 129226 | TACTTG | 2 |  |  |
| cP0000262 | PotentialSSR | PentaSSR-Cp | 129356 | 129365 | TCCAC | 2 |  |  |
| cH0000133 | PotentialSSR | HexaSSR-Cp | 129651 | 129662 | ATAAGA | 2 |  |  |
| cH0000134 | PotentialSSR | HexaSSR-Cp | 130029 | 130040 | GATAGA | 2 |  |  |
| c70000034 | ExtendedSSR | 7SSR-Cp | 130057 | 130070 | GGTTTTT | 2 |  |  |
| cP0000263 | PotentialSSR | PentaSSR-Cp | 130133 | 130142 | TGGAT | 2 |  |  |
| cP0000264 | PotentialSSR | PentaSSR-Cp | 130431 | 130440 | TCCGC | 2 | rrn23 | rrn23 [rrn23]\| |
| cTe0000009 | SSR | TetraSSR-Cp | 131520 | 131531 | CTAC | 3 | rrn23 | rrn23 [rrn23]\| |
| cH0000135 | PotentialSSR | HexaSSR-Cp | 131916 | 131927 | CGCTTC | 2 | rrn23 | rrn23 [rrn23]\| |
| cD0000009 | SSR | DiSSR-Cp | 131941 | 131950 | CG | 5 | rrn23 | rrn23 [rrn23]\| |
| c80000011 | ExtendedSSR | 8SSR-Cp | 133510 | 133525 | TCTCAAAA | 2 | trnA-UGC | (Intron)trnA-UGC [trnA-UGC]\| |
| cP0000265 | PotentialSSR | PentaSSR-Cp | 133739 | 133748 | ATTGA | 2 | trnA-UGC | (Intron)trnA-UGC [trnA-UGC]\| |
| cP0000266 | PotentialSSR | PentaSSR-Cp | 134059 | 134068 | TTTGT | 2 | trnA-UGC | (Intron)trnA-UGC [trnA-UGC]\| |
| cH0000136 | PotentialSSR | HexaSSR-Cp | 134288 | 134299 | ATTCTT | 2 |  |  |
| cH0000137 | PotentialSSR | HexaSSR-Cp | 135044 | 135055 | TCCATT | 2 | trnI-GAU | (Intron)trnI-GAU [trnI-GAU]\| |
| cH0000138 | PotentialSSR | HexaSSR-Cp | 136499 | 136510 | TCAGTG | 2 | rrn16 | rrn16 [rrn16]\| |
| cH0000139 | PotentialSSR | HexaSSR-Cp | 137374 | 137385 | TCATTC | 2 |  |  |
| cP0000267 | PotentialSSR | PentaSSR-Cp | 137500 | 137509 | TCCCT | 2 | trnV-GAC | trnV-GAC [trnV-GAC]\| |
| cP0000268 | PotentialSSR | PentaSSR-Cp | 137583 | 137592 | TCTTG | 2 |  |  |
| cM0000032 | SSR | MonoSSR-Cp | 137832 | 137843 | A | 12 |  |  |
| cH0000140 | PotentialSSR | HexaSSR-Cp | 137940 | 137951 | TTTCCA | 2 |  |  |
| cH0000141 | PotentialSSR | HexaSSR-Cp | 138086 | 138097 | ATAGTA | 2 |  |  |
| cP0000269 | PotentialSSR | PentaSSR-Cp | 138210 | 138219 | CATTG | 2 |  |  |
| cP0000270 | PotentialSSR | PentaSSR-Cp | 138393 | 138402 | CTAAT | 2 |  |  |
| cP0000271 | PotentialSSR | PentaSSR-Cp | 138412 | 138421 | TAATA | 2 |  |  |
| cP0000272 | PotentialSSR | PentaSSR-Cp | 138510 | 138519 | CAACA | 2 |  |  |
| cH0000142 | PotentialSSR | HexaSSR-Cp | 138754 | 138765 | GGGATA | 2 |  |  |
| cH0000143 | PotentialSSR | HexaSSR-Cp | 138766 | 138777 | GAGGAA | 2 |  |  |
| cH0000144 | PotentialSSR | HexaSSR-Cp | 138811 | 138822 | AAAATC | 2 |  |  |
| cP0000273 | PotentialSSR | PentaSSR-Cp | 139403 | 139412 | GATTC | 2 |  |  |
| cH0000145 | PotentialSSR | HexaSSR-Cp | 139590 | 139601 | TATGGA | 2 |  |  |
| cP0000274 | PotentialSSR | PentaSSR-Cp | 140338 | 140347 | GAACA | 2 |  |  |
| cP0000275 | PotentialSSR | PentaSSR-Cp | 140443 | 140452 | TTTCT | 2 |  |  |
| cH0000146 | PotentialSSR | HexaSSR-Cp | 140507 | 140518 | TAAGAA | 2 |  |  |
| cP0000276 | PotentialSSR | PentaSSR-Cp | 141576 | 141585 | TCTTT | 2 | ndhB | (Intron)YP_009380274.1 [ndhB]\| |
| cP0000277 | PotentialSSR | PentaSSR-Cp | 141776 | 141785 | AATCA | 2 | ndhB | (Intron)YP_009380274.1 [ndhB]\| |
| cH0000147 | PotentialSSR | HexaSSR-Cp | 142070 | 142081 | GACTCT | 2 | ndhB | YP_009380274.1 [ndhB]\| |
| cH0000148 | PotentialSSR | HexaSSR-Cp | 142127 | 142138 | GCTTCA | 2 | ndhB | YP_009380274.1 [ndhB]\| |
| cP0000278 | PotentialSSR | PentaSSR-Cp | 143091 | 143100 | TTCCA | 2 |  |  |
| cP0000279 | PotentialSSR | PentaSSR-Cp | 143227 | 143236 | ATAAG | 2 |  |  |
| cH0000149 | PotentialSSR | HexaSSR-Cp | 143540 | 143551 | TTGGAA | 2 |  |  |
| cP0000280 | PotentialSSR | PentaSSR-Cp | 143717 | 143726 | CGTAA | 2 |  |  |
| cP0000281 | PotentialSSR | PentaSSR-Cp | 143962 | 143971 | AACAA | 2 |  |  |
| cP0000282 | PotentialSSR | PentaSSR-Cp | 143990 | 143999 | AACTT | 2 |  |  |
| cH0000150 | PotentialSSR | HexaSSR-Cp | 144482 | 144493 | ATATAG | 2 | ycf2 | YP_009380275.1 [ycf2]\| |
| cH0000151 | PotentialSSR | HexaSSR-Cp | 145082 | 145093 | CTTCTA | 2 | ycf2 | YP_009380275.1 [ycf2]\| |
| cP0000283 | PotentialSSR | PentaSSR-Cp | 145589 | 145598 | TTTTC | 2 | ycf2 | YP_009380275.1 [ycf2]\| |
| cH0000152 | PotentialSSR | HexaSSR-Cp | 145968 | 145979 | CAGCTC | 2 | ycf2 | YP_009380275.1 [ycf2]\| |
| cP0000284 | PotentialSSR | PentaSSR-Cp | 146612 | 146621 | CAATC | 2 | ycf2 | YP_009380275.1 [ycf2]\| |
| cH0000153 | PotentialSSR | HexaSSR-Cp | 146632 | 146643 | CTTTTT | 2 | ycf2 | YP_009380275.1 [ycf2]\| |
| cH0000154 | PotentialSSR | HexaSSR-Cp | 146811 | 146822 | GGGACC | 2 | ycf2 | YP_009380275.1 [ycf2]\| |
| cP0000285 | PotentialSSR | PentaSSR-Cp | 147590 | 147599 | TTGAA | 2 | ycf2 | YP_009380275.1 [ycf2]\| |
| cP0000286 | PotentialSSR | PentaSSR-Cp | 147755 | 147764 | GATCG | 2 | ycf2 | YP_009380275.1 [ycf2]\| |
| c90000006 | ExtendedSSR | 9SSR-Cp | 148594 | 148611 | AAATGTTCC | 2 | ycf2 | YP_009380275.1 [ycf2]\| |
| cP0000287 | PotentialSSR | PentaSSR-Cp | 149881 | 149890 | CGGAT | 2 | ycf2 | YP_009380275.1 [ycf2]\| |
| cH0000156 | PotentialSSR | HexaSSR-Cp | 149932 | 149943 | TCTTTC | 2 | ycf2 | YP_009380275.1 [ycf2]\| |
| cP0000288 | PotentialSSR | PentaSSR-Cp | 150599 | 150608 | ATTTC | 2 |  |  |
| cP0000289 | PotentialSSR | PentaSSR-Cp | 150816 | 150825 | TCATA | 2 |  |  |
| cP0000290 | PotentialSSR | PentaSSR-Cp | 151174 | 151183 | TATTC | 2 |  |  |
| cP0000291 | PotentialSSR | PentaSSR-Cp | 151194 | 151203 | AAAGA | 2 |  |  |
| cP0000292 | PotentialSSR | PentaSSR-Cp | 151295 | 151304 | AATCC | 2 | rpl2 | YP_009380276.1 [rpl2]\| |
| cP0000293 | PotentialSSR | PentaSSR-Cp | 151644 | 151653 | ACATA | 2 | rpl2 | YP_009380276.1 [rpl2]\| |
| cH0000157 | PotentialSSR | HexaSSR-Cp | 152057 | 152068 | GAAAAT | 2 |  |  |

**Supplementary Table 3. List of SSRs identified in CAJEJG05 of *C. album***

| **Name** | **SSR type** | **Type** | **Cooridnation** | | **Unit sequence** | **Repeat number** | **Genes** | **Annotation** |
| --- | --- | --- | --- | --- | --- | --- | --- | --- |
| cH0000001 | PotentialSSR | HexaSSR-Cp | 113 | 124 | GTAAAG | 2 |  |  |
| cH0000002 | PotentialSSR | HexaSSR-Cp | 1240 | 1251 | GCTTTC | 2 | psbA | YP_009380194.1 [psbA]\| |
| cP0000001 | PotentialSSR | PentaSSR-Cp | 1292 | 1301 | ATTTA | 2 |  |  |
| c70000001 | ExtendedSSR | 7SSR-Cp | 1454 | 1467 | AGAAAAT | 2 |  |  |
| c70000002 | ExtendedSSR | 7SSR-Cp | 1696 | 1709 | AGTAGAA | 2 | trnK-UUU | (Intron)trnK-UUU [trnK-UUU]\| |
| cH0000003 | PotentialSSR | HexaSSR-Cp | 1748 | 1759 | AATTTC | 2 | trnK-UUU | (Intron)trnK-UUU [trnK-UUU]\| |
| cH0000004 | PotentialSSR | HexaSSR-Cp | 2949 | 2960 | TTTTTC | 2 | matK,trnK-UUU | YP_009380195.1 [matK]\|(Intron)trnK-UUU [trnK-UUU]\| |
| cP0000002 | PotentialSSR | PentaSSR-Cp | 4093 | 4102 | TATGT | 2 |  |  |
| cP0000003 | PotentialSSR | PentaSSR-Cp | 4124 | 4133 | AATGG | 2 |  |  |
| cP0000004 | PotentialSSR | PentaSSR-Cp | 4202 | 4211 | CAGAT | 2 |  |  |
| cP0000005 | PotentialSSR | PentaSSR-Cp | 4355 | 4364 | CATTT | 2 |  |  |
| cH0000005 | PotentialSSR | HexaSSR-Cp | 4442 | 4453 | AATATT | 2 |  |  |
| cTe0000001 | SSR | TetraSSR-Cp | 4464 | 4475 | TTTA | 3 |  |  |
| cP0000006 | PotentialSSR | PentaSSR-Cp | 4487 | 4496 | ATTTA | 2 |  |  |
| cP0000007 | PotentialSSR | PentaSSR-Cp | 4572 | 4581 | ATATA | 2 |  |  |
| cT0000001 | SSR | TriSSR-Cp | 4613 | 4624 | ATA | 4 |  |  |
| cP0000008 | PotentialSSR | PentaSSR-Cp | 4970 | 4979 | TAGAT | 2 | rps16 | (Intron)YP_009380196.1 [rps16]\| |
| cP0000009 | PotentialSSR | PentaSSR-Cp | 5204 | 5213 | CATTT | 2 | rps16 | (Intron)YP_009380196.1 [rps16]\| |
| cH0000007 | PotentialSSR | HexaSSR-Cp | 5274 | 5285 | ATCCAA | 2 | rps16 | (Intron)YP_009380196.1 [rps16]\| |
| c70000004 | ExtendedSSR | 7SSR-Cp | 5294 | 5307 | ACAATTA | 2 | rps16 | (Intron)YP_009380196.1 [rps16]\| |
| cM0000001 | SSR | MonoSSR-Cp | 5341 | 5351 | T | 11 | rps16 | (Intron)YP_009380196.1 [rps16]\| |
| cP0000010 | PotentialSSR | PentaSSR-Cp | 5441 | 5450 | AGAAT | 2 | rps16 | (Intron)YP_009380196.1 [rps16]\| |
| cP0000011 | PotentialSSR | PentaSSR-Cp | 5997 | 6006 | AATGC | 2 |  |  |
| cP0000012 | SSR | PentaSSR-Cp | 6080 | 6094 | TTAAT | 3 |  |  |
| cP0000013 | PotentialSSR | PentaSSR-Cp | 6190 | 6199 | TATAT | 2 |  |  |
| cH0000009 | PotentialSSR | HexaSSR-Cp | 6467 | 6478 | GCTCTG | 2 |  |  |
| cP0000014 | PotentialSSR | PentaSSR-Cp | 6555 | 6564 | TTCTA | 2 |  |  |
| cP0000015 | PotentialSSR | PentaSSR-Cp | 6629 | 6638 | GATTC | 2 |  |  |
| cM0000002 | SSR | MonoSSR-Cp | 6787 | 6797 | A | 11 |  |  |
| cM0000003 | SSR | MonoSSR-Cp | 6853 | 6862 | A | 10 |  |  |
| c80000001 | ExtendedSSR | 8SSR-Cp | 7100 | 7115 | ATTCTAAT | 2 |  |  |
| cH0000010 | PotentialSSR | HexaSSR-Cp | 7345 | 7356 | TTACAA | 2 |  |  |
| cP0000016 | PotentialSSR | PentaSSR-Cp | 7398 | 7407 | TCAAA | 2 |  |  |
| cP0000017 | PotentialSSR | PentaSSR-Cp | 7608 | 7617 | TTCTT | 2 |  |  |
| cP0000018 | PotentialSSR | PentaSSR-Cp | 7640 | 7649 | ATTCT | 2 |  |  |
| cM0000004 | SSR | MonoSSR-Cp | 7732 | 7742 | A | 11 |  |  |
| cP0000019 | PotentialSSR | PentaSSR-Cp | 7898 | 7907 | GAAAA | 2 |  |  |
| cP0000020 | PotentialSSR | PentaSSR-Cp | 7988 | 7997 | ATATA | 2 |  |  |
| cH0000012 | PotentialSSR | HexaSSR-Cp | 8068 | 8079 | AGATAA | 2 |  |  |
| cP0000021 | PotentialSSR | PentaSSR-Cp | 8136 | 8145 | TAAAA | 2 |  |  |
| cP0000022 | PotentialSSR | PentaSSR-Cp | 8212 | 8221 | AAAGA | 2 |  |  |
| cP0000023 | PotentialSSR | PentaSSR-Cp | 8267 | 8276 | GGCCT | 2 |  |  |
| cH0000013 | PotentialSSR | HexaSSR-Cp | 8345 | 8356 | TTTGAT | 2 |  |  |
| cP0000024 | PotentialSSR | PentaSSR-Cp | 8861 | 8870 | AATTG | 2 | trnG-UCC | (Intron)trnG-UCC [trnG-UCC]\| |
| cP0000025 | PotentialSSR | PentaSSR-Cp | 9226 | 9235 | TTTTG | 2 | trnG-UCC | (Intron)trnG-UCC [trnG-UCC]\| |
| cP0000026 | PotentialSSR | PentaSSR-Cp | 9251 | 9260 | CTTAA | 2 | trnG-UCC | (Intron)trnG-UCC [trnG-UCC]\| |
| cP0000027 | PotentialSSR | PentaSSR-Cp | 9284 | 9293 | TTTTC | 2 | trnG-UCC | (Intron)trnG-UCC [trnG-UCC]\| |
| cP0000028 | PotentialSSR | PentaSSR-Cp | 9482 | 9491 | TCTCA | 2 |  |  |
| cP0000029 | PotentialSSR | PentaSSR-Cp | 9504 | 9513 | CAAAA | 2 |  |  |
| cM0000005 | SSR | MonoSSR-Cp | 9530 | 9540 | A | 11 |  |  |
| cH0000016 | PotentialSSR | HexaSSR-Cp | 10446 | 10457 | GCTTGT | 2 | atpA | YP_009380199.1 [atpA]\| |
| cH0000017 | PotentialSSR | HexaSSR-Cp | 10849 | 10860 | CGGGAG | 2 | atpA | YP_009380199.1 [atpA]\| |
| c80000002 | ExtendedSSR | 8SSR-Cp | 11970 | 11985 | AAAAATAG | 2 | atpF | (Intron)YP_009380200.1 [atpF]\| |
| c70000008 | ExtendedSSR | 7SSR-Cp | 12213 | 12226 | TCGGTAT | 2 | atpF | (Intron)YP_009380200.1 [atpF]\| |
| cT0000002 | SSR | TriSSR-Cp | 12319 | 12330 | ATT | 4 | atpF | (Intron)YP_009380200.1 [atpF]\| |
| cTe0000002 | SSR | TetraSSR-Cp | 12884 | 12895 | GGAA | 3 |  |  |
| cP0000030 | PotentialSSR | PentaSSR-Cp | 13032 | 13041 | AGAAA | 2 |  |  |
| cP0000031 | PotentialSSR | PentaSSR-Cp | 13057 | 13066 | TTTCT | 2 |  |  |
| cD0000001 | SSR | DiSSR-Cp | 13328 | 13337 | CA | 5 |  |  |
| cP0000032 | PotentialSSR | PentaSSR-Cp | 13819 | 13828 | AAATA | 2 |  |  |
| cD0000002 | SSR | DiSSR-Cp | 13886 | 13895 | AT | 5 |  |  |
| cH0000018 | PotentialSSR | HexaSSR-Cp | 14259 | 14270 | TAAAGC | 2 | atpI | YP_009380202.1 [atpI]\| |
| cH0000019 | PotentialSSR | HexaSSR-Cp | 14758 | 14769 | TTTAAT | 2 |  |  |
| cP0000033 | PotentialSSR | PentaSSR-Cp | 15628 | 15637 | ATTAA | 2 |  |  |
| c80000003 | ExtendedSSR | 8SSR-Cp | 15687 | 15702 | TCTACCGC | 2 |  |  |
| cP0000034 | PotentialSSR | PentaSSR-Cp | 15766 | 15775 | TTTAT | 2 |  |  |
| cP0000035 | PotentialSSR | PentaSSR-Cp | 15779 | 15788 | TAAAT | 2 |  |  |
| cP0000036 | PotentialSSR | PentaSSR-Cp | 16843 | 16852 | CAATT | 2 | rpoC2 | YP_009380204.1 [rpoC2]\| |
| cP0000037 | PotentialSSR | PentaSSR-Cp | 17658 | 17667 | CAAAA | 2 | rpoC2 | YP_009380204.1 [rpoC2]\| |
| cP0000038 | PotentialSSR | PentaSSR-Cp | 17778 | 17787 | TATCT | 2 | rpoC2 | YP_009380204.1 [rpoC2]\| |
| cM0000006 | SSR | MonoSSR-Cp | 17834 | 17846 | T | 13 | rpoC2 | YP_009380204.1 [rpoC2]\| |
| cP0000039 | PotentialSSR | PentaSSR-Cp | 18317 | 18326 | CGATT | 2 | rpoC2 | YP_009380204.1 [rpoC2]\| |
| cP0000040 | PotentialSSR | PentaSSR-Cp | 18346 | 18355 | ATCCT | 2 | rpoC2 | YP_009380204.1 [rpoC2]\| |
| cH0000020 | PotentialSSR | HexaSSR-Cp | 18459 | 18470 | TTGATC | 2 | rpoC2 | YP_009380204.1 [rpoC2]\| |
| cH0000021 | PotentialSSR | HexaSSR-Cp | 18831 | 18842 | ACGTGT | 2 | rpoC2 | YP_009380204.1 [rpoC2]\| |
| cP0000041 | PotentialSSR | PentaSSR-Cp | 19043 | 19052 | CATAA | 2 | rpoC2 | YP_009380204.1 [rpoC2]\| |
| cD0000003 | SSR | DiSSR-Cp | 19186 | 19195 | AT | 5 | rpoC2 | YP_009380204.1 [rpoC2]\| |
| cH0000022 | PotentialSSR | HexaSSR-Cp | 21525 | 21536 | CAAATC | 2 | rpoC1 | YP_009380205.1 [rpoC1]\| |
| cP0000042 | PotentialSSR | PentaSSR-Cp | 21639 | 21648 | GGATT | 2 | rpoC1 | YP_009380205.1 [rpoC1]\| |
| cP0000043 | PotentialSSR | PentaSSR-Cp | 21728 | 21737 | ATCCT | 2 | rpoC1 | (Intron)YP_009380205.1 [rpoC1]\| |
| cP0000044 | PotentialSSR | PentaSSR-Cp | 21922 | 21931 | TTCTT | 2 | rpoC1 | (Intron)YP_009380205.1 [rpoC1]\| |
| cP0000045 | PotentialSSR | PentaSSR-Cp | 21954 | 21963 | AATTA | 2 | rpoC1 | (Intron)YP_009380205.1 [rpoC1]\| |
| cP0000046 | PotentialSSR | PentaSSR-Cp | 22138 | 22147 | TTAAA | 2 | rpoC1 | (Intron)YP_009380205.1 [rpoC1]\| |
| cH0000023 | PotentialSSR | HexaSSR-Cp | 22243 | 22254 | ACAAAA | 2 | rpoC1 | (Intron)YP_009380205.1 [rpoC1]\| |
| cM0000007 | SSR | MonoSSR-Cp | 25511 | 25520 | T | 10 | rpoB | YP_009380206.1 [rpoB]\| |
| cH0000024 | PotentialSSR | HexaSSR-Cp | 25954 | 25965 | TCTTTT | 2 | rpoB | YP_009380206.1 [rpoB]\| |
| cP0000047 | PotentialSSR | PentaSSR-Cp | 26433 | 26442 | ACTTA | 2 |  |  |
| cP0000048 | PotentialSSR | PentaSSR-Cp | 26726 | 26735 | TTGTA | 2 |  |  |
| cH0000026 | PotentialSSR | HexaSSR-Cp | 26887 | 26898 | AGAAAA | 2 |  |  |
| cP0000049 | PotentialSSR | PentaSSR-Cp | 27103 | 27112 | GAATC | 2 |  |  |
| cP0000050 | PotentialSSR | PentaSSR-Cp | 27563 | 27572 | TTCCC | 2 |  |  |
| cP0000051 | PotentialSSR | PentaSSR-Cp | 27692 | 27701 | CTGAG | 2 |  |  |
| cH0000027 | PotentialSSR | HexaSSR-Cp | 27804 | 27815 | TTCATA | 2 |  |  |
| cP0000052 | PotentialSSR | PentaSSR-Cp | 27936 | 27945 | TTTAG | 2 |  |  |
| cH0000028 | PotentialSSR | HexaSSR-Cp | 27962 | 27973 | TATCAT | 2 |  |  |
| cP0000053 | PotentialSSR | PentaSSR-Cp | 28195 | 28204 | ATTTC | 2 |  |  |
| cP0000054 | PotentialSSR | PentaSSR-Cp | 28263 | 28272 | TCAAT | 2 |  |  |
| cP0000055 | PotentialSSR | PentaSSR-Cp | 28289 | 28298 | TTGGC | 2 |  |  |
| cP0000056 | PotentialSSR | PentaSSR-Cp | 28347 | 28356 | ACTTT | 2 |  |  |
| c70000009 | ExtendedSSR | 7SSR-Cp | 28414 | 28427 | TTATAGT | 2 |  |  |
| cP0000057 | PotentialSSR | PentaSSR-Cp | 28451 | 28460 | CTAAT | 2 |  |  |
| cP0000058 | PotentialSSR | PentaSSR-Cp | 28827 | 28836 | TATCA | 2 |  |  |
| cH0000029 | PotentialSSR | HexaSSR-Cp | 29052 | 29063 | CAAAAA | 2 |  |  |
| cM0000008 | SSR | MonoSSR-Cp | 29378 | 29387 | A | 10 |  |  |
| cP0000059 | PotentialSSR | PentaSSR-Cp | 29756 | 29765 | ATAAA | 2 |  |  |
| c80000004 | ExtendedSSR | 8SSR-Cp | 29775 | 29790 | TTTTCTTT | 2 |  |  |
| cP0000060 | PotentialSSR | PentaSSR-Cp | 29832 | 29841 | ACCAT | 2 |  |  |
| cH0000030 | PotentialSSR | HexaSSR-Cp | 29872 | 29883 | AATTTC | 2 |  |  |
| cTe0000003 | SSR | TetraSSR-Cp | 29891 | 29902 | AAAT | 3 |  |  |
| cP0000061 | PotentialSSR | PentaSSR-Cp | 30110 | 30119 | TCAAT | 2 |  |  |
| cP0000062 | PotentialSSR | PentaSSR-Cp | 30161 | 30170 | TACCC | 2 | trnE-UUC | trnE-UUC [trnE-UUC]\| |
| cH0000031 | PotentialSSR | HexaSSR-Cp | 30365 | 30376 | ATTTCA | 2 |  |  |
| cH0000032 | PotentialSSR | HexaSSR-Cp | 30503 | 30514 | CTAATA | 2 |  |  |
| cP0000063 | PotentialSSR | PentaSSR-Cp | 30647 | 30656 | GTATA | 2 |  |  |
| cP0000064 | PotentialSSR | PentaSSR-Cp | 30959 | 30968 | ATTAT | 2 |  |  |
| cP0000065 | PotentialSSR | PentaSSR-Cp | 31032 | 31041 | TGGAA | 2 |  |  |
| c70000010 | ExtendedSSR | 7SSR-Cp | 31493 | 31506 | GTTTATT | 2 |  |  |
| cH0000033 | PotentialSSR | HexaSSR-Cp | 31570 | 31581 | GTGAAA | 2 |  |  |
| cP0000066 | PotentialSSR | PentaSSR-Cp | 31746 | 31755 | AAAGA | 2 |  |  |
| cP0000067 | PotentialSSR | PentaSSR-Cp | 31953 | 31962 | TAAAT | 2 |  |  |
| cP0000068 | PotentialSSR | PentaSSR-Cp | 32116 | 32125 | CGTTT | 2 | psbD | YP_009380209.1 [psbD]\| |
| cP0000069 | PotentialSSR | PentaSSR-Cp | 32746 | 32755 | AACCC | 2 | psbD | YP_009380209.1 [psbD]\| |
| cP0000070 | PotentialSSR | PentaSSR-Cp | 33839 | 33848 | GTCTG | 2 | psbC | YP_009380210.1 [psbC]\| |
| cH0000034 | PotentialSSR | HexaSSR-Cp | 33975 | 33986 | CTCAAG | 2 | psbC | YP_009380210.1 [psbC]\| |
| cP0000071 | PotentialSSR | PentaSSR-Cp | 34267 | 34276 | GGGTG | 2 | psbC | YP_009380210.1 [psbC]\| |
| cH0000035 | PotentialSSR | HexaSSR-Cp | 34393 | 34404 | TGCAGC | 2 | psbC | YP_009380210.1 [psbC]\| |
| c70000011 | ExtendedSSR | 7SSR-Cp | 34516 | 34529 | TACATAT | 2 |  |  |
| cP0000072 | PotentialSSR | PentaSSR-Cp | 34594 | 34603 | TAATT | 2 |  |  |
| cH0000036 | PotentialSSR | HexaSSR-Cp | 34825 | 34836 | TTATTC | 2 |  |  |
| cP0000073 | PotentialSSR | PentaSSR-Cp | 34927 | 34936 | TATAT | 2 |  |  |
| c70000012 | ExtendedSSR | 7SSR-Cp | 34967 | 34980 | TTAATTA | 2 |  |  |
| cP0000074 | PotentialSSR | PentaSSR-Cp | 35303 | 35312 | TGGAT | 2 | psbZ | YP_009380211.1 [psbZ]\| |
| cP0000075 | PotentialSSR | PentaSSR-Cp | 35471 | 35480 | AAACA | 2 |  |  |
| cP0000076 | PotentialSSR | PentaSSR-Cp | 35634 | 35643 | TATAT | 2 |  |  |
| cH0000037 | PotentialSSR | HexaSSR-Cp | 35800 | 35811 | GTTATA | 2 |  |  |
| cP0000077 | PotentialSSR | PentaSSR-Cp | 35823 | 35832 | TAGTG | 2 |  |  |
| cP0000078 | PotentialSSR | PentaSSR-Cp | 35926 | 35935 | TATTT | 2 |  |  |
| cP0000079 | PotentialSSR | PentaSSR-Cp | 36837 | 36846 | CCACG | 2 | psaB | YP_009380213.1 [psaB]\| |
| cP0000080 | PotentialSSR | PentaSSR-Cp | 36986 | 36995 | CCATC | 2 | psaB | YP_009380213.1 [psaB]\| |
| cP0000081 | PotentialSSR | PentaSSR-Cp | 38265 | 38274 | TGTCC | 2 | psaB | YP_009380213.1 [psaB]\| |
| cP0000082 | PotentialSSR | PentaSSR-Cp | 38771 | 38780 | ACCAA | 2 | psaB | YP_009380213.1 [psaB]\| |
| cH0000038 | PotentialSSR | HexaSSR-Cp | 39197 | 39208 | TAATAG | 2 | psaA | YP_009380214.1 [psaA]\| |
| cP0000083 | PotentialSSR | PentaSSR-Cp | 39946 | 39955 | ATGTG | 2 | psaA | YP_009380214.1 [psaA]\| |
| cP0000084 | PotentialSSR | PentaSSR-Cp | 41583 | 41592 | TATTT | 2 |  |  |
| cH0000039 | PotentialSSR | HexaSSR-Cp | 41761 | 41772 | TCTTTA | 2 |  |  |
| cP0000085 | PotentialSSR | PentaSSR-Cp | 41855 | 41864 | TTTTA | 2 |  |  |
| cM0000009 | SSR | MonoSSR-Cp | 41894 | 41903 | A | 10 |  |  |
| cP0000086 | PotentialSSR | PentaSSR-Cp | 42436 | 42445 | AAAGA | 2 | ycf3 | (Intron)YP_009380215.1 [ycf3]\| |
| cP0000087 | PotentialSSR | PentaSSR-Cp | 42573 | 42582 | TTCTT | 2 | ycf3 | (Intron)YP_009380215.1 [ycf3]\| |
| cM0000010 | SSR | MonoSSR-Cp | 42646 | 42655 | T | 10 | ycf3 | (Intron)YP_009380215.1 [ycf3]\| |
| cP0000088 | PotentialSSR | PentaSSR-Cp | 43147 | 43156 | AATAT | 2 | ycf3 | (Intron)YP_009380215.1 [ycf3]\| |
| cP0000089 | PotentialSSR | PentaSSR-Cp | 43822 | 43831 | AAATC | 2 | ycf3 | (Intron)YP_009380215.1 [ycf3]\| |
| cP0000090 | PotentialSSR | PentaSSR-Cp | 44031 | 44040 | GTACA | 2 |  |  |
| cP0000091 | PotentialSSR | PentaSSR-Cp | 44120 | 44129 | TATTT | 2 |  |  |
| cP0000092 | PotentialSSR | PentaSSR-Cp | 44207 | 44216 | CAAAT | 2 |  |  |
| cP0000093 | PotentialSSR | PentaSSR-Cp | 44221 | 44230 | TAGTA | 2 |  |  |
| cP0000094 | PotentialSSR | PentaSSR-Cp | 44274 | 44283 | GATCA | 2 |  |  |
| cP0000095 | PotentialSSR | PentaSSR-Cp | 44343 | 44352 | AATAG | 2 |  |  |
| cP0000096 | PotentialSSR | PentaSSR-Cp | 44520 | 44529 | GATTC | 2 |  |  |
| cTe0000004 | SSR | TetraSSR-Cp | 44535 | 44546 | AATT | 3 |  |  |
| cP0000097 | PotentialSSR | PentaSSR-Cp | 44555 | 44564 | TATAT | 2 |  |  |
| cD0000004 | SSR | DiSSR-Cp | 44627 | 44636 | AT | 5 |  |  |
| cP0000098 | PotentialSSR | PentaSSR-Cp | 44655 | 44664 | TTTTA | 2 |  |  |
| cP0000099 | PotentialSSR | PentaSSR-Cp | 44706 | 44715 | ATTAC | 2 |  |  |
| cP0000100 | PotentialSSR | PentaSSR-Cp | 44754 | 44763 | ATAGT | 2 |  |  |
| cP0000101 | PotentialSSR | PentaSSR-Cp | 44839 | 44848 | ATAGT | 2 |  |  |
| cP0000102 | PotentialSSR | PentaSSR-Cp | 45027 | 45036 | CAAAT | 2 |  |  |
| cP0000103 | PotentialSSR | PentaSSR-Cp | 45203 | 45212 | GGGAT | 2 |  |  |
| cH0000040 | PotentialSSR | HexaSSR-Cp | 45270 | 45281 | TTTTCC | 2 |  |  |
| cP0000104 | PotentialSSR | PentaSSR-Cp | 45696 | 45705 | ACCTG | 2 | rps4 | YP_009380216.1 [rps4]\| |
| cD0000005 | SSR | DiSSR-Cp | 45979 | 45992 | TA | 7 |  |  |
| cP0000105 | PotentialSSR | PentaSSR-Cp | 46037 | 46046 | AAATG | 2 |  |  |
| cP0000106 | PotentialSSR | PentaSSR-Cp | 46128 | 46137 | TATTT | 2 |  |  |
| cH0000041 | PotentialSSR | HexaSSR-Cp | 46157 | 46168 | AAAAAG | 2 |  |  |
| cP0000107 | PotentialSSR | PentaSSR-Cp | 46212 | 46221 | CTTTT | 2 |  |  |
| cP0000108 | PotentialSSR | PentaSSR-Cp | 46504 | 46513 | TCAAA | 2 |  |  |
| cP0000109 | PotentialSSR | PentaSSR-Cp | 46582 | 46591 | CGATT | 2 |  |  |
| cP0000110 | PotentialSSR | PentaSSR-Cp | 46818 | 46827 | AATAA | 2 |  |  |
| cP0000111 | PotentialSSR | PentaSSR-Cp | 47407 | 47416 | CTGTG | 2 | trnL-UAA | (Intron)trnL-UAA [trnL-UAA]\| |
| cP0000112 | PotentialSSR | PentaSSR-Cp | 47500 | 47509 | TCTAT | 2 | trnL-UAA | (Intron)trnL-UAA [trnL-UAA]\| |
| cP0000113 | PotentialSSR | PentaSSR-Cp | 47891 | 47900 | TTTTC | 2 |  |  |
| cD0000006 | SSR | DiSSR-Cp | 47948 | 47957 | AT | 5 |  |  |
| cP0000114 | PotentialSSR | PentaSSR-Cp | 48262 | 48271 | CACAT | 2 |  |  |
| cH0000043 | PotentialSSR | HexaSSR-Cp | 48391 | 48402 | GAAAAT | 2 |  |  |
| cP0000115 | PotentialSSR | PentaSSR-Cp | 48522 | 48531 | CTTAA | 2 |  |  |
| cP0000116 | PotentialSSR | PentaSSR-Cp | 48541 | 48550 | TCTTT | 2 |  |  |
| cH0000044 | PotentialSSR | HexaSSR-Cp | 49010 | 49021 | CATATA | 2 | ndhJ | YP_009380217.1 [ndhJ]\| |
| c80000006 | ExtendedSSR | 8SSR-Cp | 49167 | 49182 | ATTTGTTT | 2 |  |  |
| cP0000117 | PotentialSSR | PentaSSR-Cp | 49190 | 49199 | TATTT | 2 |  |  |
| cP0000118 | PotentialSSR | PentaSSR-Cp | 49203 | 49212 | TTTAC | 2 |  |  |
| cH0000045 | PotentialSSR | HexaSSR-Cp | 49970 | 49981 | TTTTTC | 2 |  |  |
| cP0000119 | PotentialSSR | PentaSSR-Cp | 50035 | 50044 | TAAAC | 2 | ndhC | YP_009380219.1 [ndhC]\| |
| cP0000120 | PotentialSSR | PentaSSR-Cp | 50597 | 50606 | TTCTT | 2 |  |  |
| cP0000121 | PotentialSSR | PentaSSR-Cp | 50673 | 50682 | AATTC | 2 |  |  |
| cP0000122 | PotentialSSR | PentaSSR-Cp | 50729 | 50738 | TTCTA | 2 |  |  |
| cP0000123 | PotentialSSR | PentaSSR-Cp | 50788 | 50797 | ACTTG | 2 |  |  |
| c70000015 | ExtendedSSR | 7SSR-Cp | 52545 | 52558 | AATTTTT | 2 |  |  |
| cP0000124 | PotentialSSR | PentaSSR-Cp | 52683 | 52692 | ATTTG | 2 |  |  |
| cP0000125 | PotentialSSR | PentaSSR-Cp | 52784 | 52793 | TTGAA | 2 |  |  |
| cH0000047 | PotentialSSR | HexaSSR-Cp | 52837 | 52848 | TGTATA | 2 |  |  |
| cP0000126 | PotentialSSR | PentaSSR-Cp | 53064 | 53073 | ATTGA | 2 |  |  |
| cP0000127 | PotentialSSR | PentaSSR-Cp | 53103 | 53112 | AATTG | 2 |  |  |
| cM0000011 | SSR | MonoSSR-Cp | 53194 | 53205 | T | 12 |  |  |
| cH0000048 | PotentialSSR | HexaSSR-Cp | 53216 | 53227 | ATTTTC | 2 |  |  |
| cM0000012 | SSR | MonoSSR-Cp | 53301 | 53310 | A | 10 | atpB | YP_009380221.1 [atpB]\| |
| cP0000128 | PotentialSSR | PentaSSR-Cp | 54477 | 54486 | TTGGA | 2 | atpB | YP_009380221.1 [atpB]\| |
| cH0000049 | PotentialSSR | HexaSSR-Cp | 55174 | 55185 | ATAATC | 2 |  |  |
| cH0000050 | PotentialSSR | HexaSSR-Cp | 55244 | 55255 | TCTTTT | 2 |  |  |
| cH0000051 | PotentialSSR | HexaSSR-Cp | 55282 | 55293 | AAAAAG | 2 |  |  |
| cP0000129 | PotentialSSR | PentaSSR-Cp | 55756 | 55765 | AAACA | 2 | trnV-UAC | (Intron)trnV-UAC [trnV-UAC]\| |
| c70000016 | ExtendedSSR | 7SSR-Cp | 56088 | 56101 | AATGAAT | 2 | trnV-UAC | (Intron)trnV-UAC [trnV-UAC]\| |
| cH0000052 | PotentialSSR | HexaSSR-Cp | 56334 | 56345 | CAATTG | 2 |  |  |
| cP0000130 | PotentialSSR | PentaSSR-Cp | 56395 | 56404 | ATTTG | 2 |  |  |
| cH0000053 | PotentialSSR | HexaSSR-Cp | 56419 | 56430 | TTTGAA | 2 |  |  |
| cP0000131 | PotentialSSR | PentaSSR-Cp | 56518 | 56527 | TTTTC | 2 |  |  |
| cP0000132 | PotentialSSR | PentaSSR-Cp | 58584 | 58593 | TATAT | 2 |  |  |
| cP0000133 | PotentialSSR | PentaSSR-Cp | 58640 | 58649 | CTATA | 2 |  |  |
| cTe0000005 | SSR | TetraSSR-Cp | 58667 | 58678 | TAAT | 3 |  |  |
| cP0000134 | PotentialSSR | PentaSSR-Cp | 58681 | 58690 | TAATA | 2 |  |  |
| cH0000054 | PotentialSSR | HexaSSR-Cp | 58722 | 58733 | TACAAT | 2 |  |  |
| cP0000135 | PotentialSSR | PentaSSR-Cp | 58852 | 58861 | AAAAT | 2 | psaI | YP_009380224.1 [psaI]\| |
| cP0000136 | PotentialSSR | PentaSSR-Cp | 58870 | 58879 | TCGTC | 2 |  |  |
| cP0000137 | PotentialSSR | PentaSSR-Cp | 59010 | 59019 | ACAAT | 2 |  |  |
| cP0000138 | PotentialSSR | PentaSSR-Cp | 59174 | 59183 | TTCAA | 2 |  |  |
| cP0000139 | PotentialSSR | PentaSSR-Cp | 59484 | 59493 | TCGAT | 2 | ycf4 | YP_009380225.1 [ycf4]\| |
| cP0000140 | PotentialSSR | PentaSSR-Cp | 59589 | 59598 | TAGAA | 2 | ycf4 | YP_009380225.1 [ycf4]\| |
| cH0000055 | PotentialSSR | HexaSSR-Cp | 59922 | 59933 | TACAAA | 2 |  |  |
| cP0000141 | PotentialSSR | PentaSSR-Cp | 59935 | 59944 | CAATT | 2 |  |  |
| cP0000142 | PotentialSSR | PentaSSR-Cp | 60075 | 60084 | AATTA | 2 |  |  |
| cP0000143 | PotentialSSR | PentaSSR-Cp | 61040 | 61049 | AAAAG | 2 |  |  |
| c70000017 | ExtendedSSR | 7SSR-Cp | 61104 | 61117 | ATAATCA | 2 |  |  |
| cP0000144 | PotentialSSR | PentaSSR-Cp | 61189 | 61198 | TTATT | 2 |  |  |
| cP0000145 | PotentialSSR | PentaSSR-Cp | 61687 | 61696 | AAAGA | 2 | petA | YP_009380227.1 [petA]\| |
| cP0000146 | PotentialSSR | PentaSSR-Cp | 61958 | 61967 | GAAAA | 2 | petA | YP_009380227.1 [petA]\| |
| cH0000056 | PotentialSSR | HexaSSR-Cp | 62278 | 62289 | ATCAAG | 2 |  |  |
| cH0000057 | PotentialSSR | HexaSSR-Cp | 62294 | 62305 | TAACAA | 2 |  |  |
| cP0000147 | PotentialSSR | PentaSSR-Cp | 62318 | 62327 | CAATT | 2 |  |  |
| cM0000013 | SSR | MonoSSR-Cp | 62352 | 62366 | A | 15 |  |  |
| c80000007 | ExtendedSSR | 8SSR-Cp | 62460 | 62475 | ACTTTTTT | 2 |  |  |
| cH0000058 | PotentialSSR | HexaSSR-Cp | 62667 | 62678 | ATTTTT | 2 |  |  |
| cH0000059 | PotentialSSR | HexaSSR-Cp | 62862 | 62873 | TCTTTC | 2 |  |  |
| cP0000148 | PotentialSSR | PentaSSR-Cp | 62876 | 62885 | TTAAC | 2 |  |  |
| c70000018 | ExtendedSSR | 7SSR-Cp | 63128 | 63141 | TTACTAC | 2 | psbJ | YP_009380228.1 [psbJ]\| |
| cP0000149 | PotentialSSR | PentaSSR-Cp | 63481 | 63490 | ATTCA | 2 | psbL | YP_009380229.1 [psbL]\| |
| cH0000060 | PotentialSSR | HexaSSR-Cp | 63532 | 63543 | ATTCGG | 2 |  |  |
| c80000008 | ExtendedSSR | 8SSR-Cp | 64012 | 64027 | ACGTAAAA | 2 |  |  |
| cH0000061 | PotentialSSR | HexaSSR-Cp | 64349 | 64360 | TATAGA | 2 |  |  |
| cH0000062 | PotentialSSR | HexaSSR-Cp | 64588 | 64599 | AGTCTT | 2 |  |  |
| cP0000150 | PotentialSSR | PentaSSR-Cp | 65144 | 65153 | CTGTA | 2 |  |  |
| cM0000014 | SSR | MonoSSR-Cp | 65259 | 65272 | T | 14 |  |  |
| cP0000151 | PotentialSSR | PentaSSR-Cp | 65464 | 65473 | GATTA | 2 |  |  |
| cP0000152 | PotentialSSR | PentaSSR-Cp | 65481 | 65490 | TTTAG | 2 |  |  |
| cH0000063 | PotentialSSR | HexaSSR-Cp | 65500 | 65511 | TTTCTA | 2 |  |  |
| cP0000153 | PotentialSSR | PentaSSR-Cp | 65598 | 65607 | GAACT | 2 | trnW-CCA | trnW-CCA [trnW-CCA]\| |
| cH0000064 | PotentialSSR | HexaSSR-Cp | 65674 | 65685 | CTATAT | 2 |  |  |
| cP0000154 | PotentialSSR | PentaSSR-Cp | 65881 | 65890 | TTCAA | 2 |  |  |
| cP0000155 | PotentialSSR | PentaSSR-Cp | 65920 | 65929 | CCTTG | 2 |  |  |
| cP0000156 | PotentialSSR | PentaSSR-Cp | 66056 | 66065 | GTAAA | 2 |  |  |
| cP0000157 | PotentialSSR | PentaSSR-Cp | 66168 | 66177 | CTTAG | 2 |  |  |
| cP0000158 | PotentialSSR | PentaSSR-Cp | 66524 | 66533 | TTAGT | 2 |  |  |
| cP0000159 | PotentialSSR | PentaSSR-Cp | 66577 | 66586 | GTTAA | 2 |  |  |
| c70000019 | ExtendedSSR | 7SSR-Cp | 66718 | 66731 | CGAATTG | 2 |  |  |
| cH0000065 | PotentialSSR | HexaSSR-Cp | 67081 | 67092 | AATTTT | 2 |  |  |
| cD0000007 | SSR | DiSSR-Cp | 67106 | 67115 | AT | 5 |  |  |
| cP0000160 | PotentialSSR | PentaSSR-Cp | 67175 | 67184 | ATATA | 2 |  |  |
| cP0000161 | PotentialSSR | PentaSSR-Cp | 67250 | 67259 | ATAGG | 2 |  |  |
| cP0000162 | PotentialSSR | PentaSSR-Cp | 67277 | 67286 | TAAAC | 2 |  |  |
| cTe0000006 | SSR | TetraSSR-Cp | 67761 | 67772 | TTTA | 3 |  |  |
| cP0000163 | PotentialSSR | PentaSSR-Cp | 68324 | 68333 | ATAAA | 2 |  |  |
| cH0000066 | PotentialSSR | HexaSSR-Cp | 68815 | 68826 | ATTTTC | 2 |  |  |
| cP0000164 | PotentialSSR | PentaSSR-Cp | 68933 | 68942 | CTATT | 2 |  |  |
| cH0000067 | PotentialSSR | HexaSSR-Cp | 68980 | 68991 | TTTTGT | 2 |  |  |
| cH0000068 | PotentialSSR | HexaSSR-Cp | 69300 | 69311 | TGGGCT | 2 | clpP | YP_009380239.1 [clpP]\| |
| cP0000165 | PotentialSSR | PentaSSR-Cp | 69530 | 69539 | ACACA | 2 | clpP | (Intron)YP_009380239.1 [clpP]\| |
| cP0000166 | PotentialSSR | PentaSSR-Cp | 69675 | 69684 | ATCGA | 2 | clpP | (Intron)YP_009380239.1 [clpP]\| |
| cH0000069 | PotentialSSR | HexaSSR-Cp | 69686 | 69697 | CAGATC | 2 | clpP | (Intron)YP_009380239.1 [clpP]\| |
| cP0000167 | PotentialSSR | PentaSSR-Cp | 69971 | 69980 | GAAAA | 2 | clpP | (Intron)YP_009380239.1 [clpP]\| |
| cH0000070 | PotentialSSR | HexaSSR-Cp | 70261 | 70272 | ACAAAT | 2 | clpP | YP_009380239.1 [clpP]\| |
| cP0000168 | PotentialSSR | PentaSSR-Cp | 70674 | 70683 | TATCA | 2 | clpP | (Intron)YP_009380239.1 [clpP]\| |
| cH0000071 | PotentialSSR | HexaSSR-Cp | 70714 | 70725 | TTCTTG | 2 | clpP | (Intron)YP_009380239.1 [clpP]\| |
| cH0000072 | PotentialSSR | HexaSSR-Cp | 70924 | 70935 | TTGAAA | 2 | clpP | (Intron)YP_009380239.1 [clpP]\| |
| cH0000073 | PotentialSSR | HexaSSR-Cp | 70991 | 71002 | ATTGGG | 2 | clpP | (Intron)YP_009380239.1 [clpP]\| |
| cP0000169 | PotentialSSR | PentaSSR-Cp | 71560 | 71569 | ATAGA | 2 |  |  |
| cH0000074 | PotentialSSR | HexaSSR-Cp | 71679 | 71690 | CATAGT | 2 |  |  |
| c70000021 | ExtendedSSR | 7SSR-Cp | 71829 | 71842 | CTGGTTG | 2 | psbB | YP_009380240.1 [psbB]\| |
| cH0000075 | PotentialSSR | HexaSSR-Cp | 72193 | 72204 | GTTTTG | 2 | psbB | YP_009380240.1 [psbB]\| |
| cP0000170 | PotentialSSR | PentaSSR-Cp | 73299 | 73308 | TTTGA | 2 |  |  |
| cP0000171 | PotentialSSR | PentaSSR-Cp | 73456 | 73465 | CTCTA | 2 | psbT | YP_009380241.1 [psbT]\| |
| cP0000172 | PotentialSSR | PentaSSR-Cp | 73520 | 73529 | AAATG | 2 | psbT | YP_009380241.1 [psbT]\| |
| cH0000076 | PotentialSSR | HexaSSR-Cp | 74051 | 74062 | ACAAAA | 2 |  |  |
| c90000002 | ExtendedSSR | 9SSR-Cp | 74100 | 74117 | CAATACAAA | 2 |  |  |
| cP0000173 | PotentialSSR | PentaSSR-Cp | 74403 | 74412 | TTTTC | 2 | petB | (Intron)YP_009380244.1 [petB]\| |
| cP0000174 | PotentialSSR | PentaSSR-Cp | 74521 | 74530 | AATAA | 2 | petB | (Intron)YP_009380244.1 [petB]\| |
| cP0000175 | PotentialSSR | PentaSSR-Cp | 74533 | 74542 | CCTAT | 2 | petB | (Intron)YP_009380244.1 [petB]\| |
| cH0000077 | PotentialSSR | HexaSSR-Cp | 74709 | 74720 | ATTATA | 2 | petB | (Intron)YP_009380244.1 [petB]\| |
| cH0000078 | PotentialSSR | HexaSSR-Cp | 74721 | 74732 | TACAAA | 2 | petB | (Intron)YP_009380244.1 [petB]\| |
| cP0000176 | PotentialSSR | PentaSSR-Cp | 75663 | 75672 | ATAGA | 2 | petB | YP_009380244.1 [petB]\| |
| cP0000177 | PotentialSSR | PentaSSR-Cp | 76032 | 76041 | TCTAT | 2 | petD | (Intron)YP_009380245.1 [petD]\| |
| cP0000178 | PotentialSSR | PentaSSR-Cp | 76108 | 76117 | ATAAT | 2 | petD | (Intron)YP_009380245.1 [petD]\| |
| cP0000179 | PotentialSSR | PentaSSR-Cp | 76122 | 76131 | ATTAT | 2 | petD | (Intron)YP_009380245.1 [petD]\| |
| cP0000180 | PotentialSSR | PentaSSR-Cp | 76145 | 76154 | TTATT | 2 | petD | (Intron)YP_009380245.1 [petD]\| |
| cP0000181 | PotentialSSR | PentaSSR-Cp | 76617 | 76626 | GAATC | 2 | petD | (Intron)YP_009380245.1 [petD]\| |
| cP0000182 | PotentialSSR | PentaSSR-Cp | 76630 | 76639 | AAGAA | 2 | petD | (Intron)YP_009380245.1 [petD]\| |
| cP0000183 | PotentialSSR | PentaSSR-Cp | 77209 | 77218 | ATTCA | 2 |  |  |
| cM0000015 | SSR | MonoSSR-Cp | 77436 | 77445 | T | 10 | rpoA | YP_009380246.1 [rpoA]\| |
| cH0000079 | PotentialSSR | HexaSSR-Cp | 77655 | 77666 | CATTTC | 2 | rpoA | YP_009380246.1 [rpoA]\| |
| cP0000184 | PotentialSSR | PentaSSR-Cp | 78085 | 78094 | TCGCA | 2 | rpoA | YP_009380246.1 [rpoA]\| |
| cP0000185 | PotentialSSR | PentaSSR-Cp | 78853 | 78862 | TAGTA | 2 |  |  |
| cM0000016 | SSR | MonoSSR-Cp | 78996 | 79006 | T | 11 |  |  |
| cH0000080 | PotentialSSR | HexaSSR-Cp | 79079 | 79090 | GTTGAA | 2 |  |  |
| cP0000186 | PotentialSSR | PentaSSR-Cp | 79911 | 79920 | TTAGT | 2 |  |  |
| cH0000081 | PotentialSSR | HexaSSR-Cp | 79982 | 79993 | TATTTT | 2 |  |  |
| cH0000082 | PotentialSSR | HexaSSR-Cp | 80989 | 81000 | AAAAAT | 2 | rpl16 | (Intron)YP_009380252.1 [rpl16]\| |
| cP0000187 | PotentialSSR | PentaSSR-Cp | 81016 | 81025 | TATTT | 2 | rpl16 | (Intron)YP_009380252.1 [rpl16]\| |
| c70000023 | ExtendedSSR | 7SSR-Cp | 81234 | 81247 | TTTTATA | 2 | rpl16 | (Intron)YP_009380252.1 [rpl16]\| |
| cP0000188 | PotentialSSR | PentaSSR-Cp | 81290 | 81299 | AAAAG | 2 | rpl16 | (Intron)YP_009380252.1 [rpl16]\| |
| cM0000017 | SSR | MonoSSR-Cp | 81576 | 81585 | T | 10 | rpl16 | (Intron)YP_009380252.1 [rpl16]\| |
| cM0000018 | SSR | MonoSSR-Cp | 81709 | 81719 | T | 11 | rpl16 | (Intron)YP_009380252.1 [rpl16]\| |
| cM0000019 | SSR | MonoSSR-Cp | 81768 | 81777 | T | 10 | rpl16 | (Intron)YP_009380252.1 [rpl16]\| |
| cH0000084 | PotentialSSR | HexaSSR-Cp | 81957 | 81968 | TTTTAA | 2 | rpl16 | (Intron)YP_009380252.1 [rpl16]\| |
| cP0000189 | PotentialSSR | PentaSSR-Cp | 82264 | 82273 | ACCCT | 2 | rps3 | YP_009380253.1 [rps3]\| |
| cP0000190 | PotentialSSR | PentaSSR-Cp | 82398 | 82407 | TACTC | 2 | rps3 | YP_009380253.1 [rps3]\| |
| cP0000191 | PotentialSSR | PentaSSR-Cp | 82501 | 82510 | CAATT | 2 | rps3 | YP_009380253.1 [rps3]\| |
| cP0000192 | PotentialSSR | PentaSSR-Cp | 82859 | 82868 | TTTAT | 2 | rpl22 | YP_009380254.1 [rpl22]\| |
| cP0000193 | PotentialSSR | PentaSSR-Cp | 83380 | 83389 | TTTCT | 2 |  |  |
| cH0000085 | PotentialSSR | HexaSSR-Cp | 83809 | 83820 | ATTTTC | 2 |  |  |
| cP0000194 | PotentialSSR | PentaSSR-Cp | 84224 | 84233 | TATGT | 2 | rpl2 | YP_009380256.1 [rpl2]\| |
| cP0000195 | PotentialSSR | PentaSSR-Cp | 84572 | 84581 | TGGAT | 2 | rpl2 | YP_009380256.1 [rpl2]\| |
| cP0000196 | PotentialSSR | PentaSSR-Cp | 84673 | 84682 | TTCTT | 2 |  |  |
| cP0000197 | PotentialSSR | PentaSSR-Cp | 84694 | 84703 | GAATA | 2 |  |  |
| cP0000198 | PotentialSSR | PentaSSR-Cp | 85052 | 85061 | TATGA | 2 |  |  |
| cP0000199 | PotentialSSR | PentaSSR-Cp | 85268 | 85277 | TGAAA | 2 |  |  |
| cH0000086 | PotentialSSR | HexaSSR-Cp | 85934 | 85945 | GAAAGA | 2 | ycf2 | YP_009380257.1 [ycf2]\| |
| cP0000200 | PotentialSSR | PentaSSR-Cp | 85986 | 85995 | GATCC | 2 | ycf2 | YP_009380257.1 [ycf2]\| |
| c90000003 | ExtendedSSR | 9SSR-Cp | 87266 | 87283 | GGAACATTT | 2 | ycf2 | YP_009380257.1 [ycf2]\| |
| cP0000201 | PotentialSSR | PentaSSR-Cp | 88113 | 88122 | CGATC | 2 | ycf2 | YP_009380257.1 [ycf2]\| |
| cP0000202 | PotentialSSR | PentaSSR-Cp | 88278 | 88287 | TTCAA | 2 | ycf2 | YP_009380257.1 [ycf2]\| |
| cH0000088 | PotentialSSR | HexaSSR-Cp | 89055 | 89066 | GGTCCC | 2 | ycf2 | YP_009380257.1 [ycf2]\| |
| cH0000089 | PotentialSSR | HexaSSR-Cp | 89231 | 89242 | AAGAAA | 2 | ycf2 | YP_009380257.1 [ycf2]\| |
| cP0000203 | PotentialSSR | PentaSSR-Cp | 89256 | 89265 | GATTG | 2 | ycf2 | YP_009380257.1 [ycf2]\| |
| cH0000090 | PotentialSSR | HexaSSR-Cp | 89897 | 89908 | GGAGCT | 2 | ycf2 | YP_009380257.1 [ycf2]\| |
| cP0000204 | PotentialSSR | PentaSSR-Cp | 90279 | 90288 | GAAAA | 2 | ycf2 | YP_009380257.1 [ycf2]\| |
| cH0000091 | PotentialSSR | HexaSSR-Cp | 90784 | 90795 | TAGAAG | 2 | ycf2 | YP_009380257.1 [ycf2]\| |
| cH0000092 | PotentialSSR | HexaSSR-Cp | 91384 | 91395 | CTATAT | 2 | ycf2 | YP_009380257.1 [ycf2]\| |
| cP0000205 | PotentialSSR | PentaSSR-Cp | 91878 | 91887 | AAGTT | 2 |  |  |
| cP0000206 | PotentialSSR | PentaSSR-Cp | 91906 | 91915 | TTGTT | 2 |  |  |
| cP0000207 | PotentialSSR | PentaSSR-Cp | 92150 | 92159 | GTTAC | 2 |  |  |
| cH0000093 | PotentialSSR | HexaSSR-Cp | 92325 | 92336 | ATTCCA | 2 |  |  |
| cP0000208 | PotentialSSR | PentaSSR-Cp | 92641 | 92650 | CTTAT | 2 |  |  |
| cP0000209 | PotentialSSR | PentaSSR-Cp | 92776 | 92785 | ATGGA | 2 |  |  |
| cH0000094 | PotentialSSR | HexaSSR-Cp | 93737 | 93748 | GCTGAA | 2 | ndhB | YP_009380258.1 [ndhB]\| |
| cH0000095 | PotentialSSR | HexaSSR-Cp | 93796 | 93807 | AGAGTC | 2 | ndhB | YP_009380258.1 [ndhB]\| |
| cP0000210 | PotentialSSR | PentaSSR-Cp | 94092 | 94101 | TGATT | 2 | ndhB | (Intron)YP_009380258.1 [ndhB]\| |
| cP0000211 | PotentialSSR | PentaSSR-Cp | 94292 | 94301 | AAAGA | 2 | ndhB | (Intron)YP_009380258.1 [ndhB]\| |
| cH0000096 | PotentialSSR | HexaSSR-Cp | 95359 | 95370 | TTCTTA | 2 |  |  |
| cP0000212 | PotentialSSR | PentaSSR-Cp | 95425 | 95434 | AGAAA | 2 |  |  |
| cP0000213 | PotentialSSR | PentaSSR-Cp | 95529 | 95538 | CTGTT | 2 |  |  |
| cH0000097 | PotentialSSR | HexaSSR-Cp | 96276 | 96287 | TCCATA | 2 |  |  |
| cP0000214 | PotentialSSR | PentaSSR-Cp | 96464 | 96473 | CGAAT | 2 |  |  |
| cH0000098 | PotentialSSR | HexaSSR-Cp | 97053 | 97064 | TTGATT | 2 |  |  |
| cH0000099 | PotentialSSR | HexaSSR-Cp | 97100 | 97111 | TTCCTC | 2 |  |  |
| cH0000100 | PotentialSSR | HexaSSR-Cp | 97112 | 97123 | TATCCC | 2 |  |  |
| cP0000215 | PotentialSSR | PentaSSR-Cp | 97358 | 97367 | TGTTG | 2 |  |  |
| cP0000216 | PotentialSSR | PentaSSR-Cp | 97456 | 97465 | TATTA | 2 |  |  |
| cP0000217 | PotentialSSR | PentaSSR-Cp | 97475 | 97484 | ATTAG | 2 |  |  |
| cP0000218 | PotentialSSR | PentaSSR-Cp | 97657 | 97666 | GCAAT | 2 |  |  |
| cH0000101 | PotentialSSR | HexaSSR-Cp | 97777 | 97788 | TATTAC | 2 |  |  |
| cH0000102 | PotentialSSR | HexaSSR-Cp | 97924 | 97935 | AATGGA | 2 |  |  |
| cM0000020 | SSR | MonoSSR-Cp | 98034 | 98045 | T | 12 |  |  |
| cP0000219 | PotentialSSR | PentaSSR-Cp | 98285 | 98294 | CAAGA | 2 |  |  |
| cP0000220 | PotentialSSR | PentaSSR-Cp | 98368 | 98377 | AGGGA | 2 |  |  |
| cH0000103 | PotentialSSR | HexaSSR-Cp | 98492 | 98503 | GAATGA | 2 |  |  |
| cH0000104 | PotentialSSR | HexaSSR-Cp | 99365 | 99376 | GACACT | 2 | rrn16 | rrn16 [rrn16]\| |
| cH0000105 | PotentialSSR | HexaSSR-Cp | 100822 | 100833 | AATGGA | 2 | trnI-GAU | (Intron)trnI-GAU [trnI-GAU]\| |
| cH0000106 | PotentialSSR | HexaSSR-Cp | 101578 | 101589 | AAGAAT | 2 |  |  |
| cP0000221 | PotentialSSR | PentaSSR-Cp | 101809 | 101818 | ACAAA | 2 | trnA-UGC | (Intron)trnA-UGC [trnA-UGC]\| |
| cP0000222 | PotentialSSR | PentaSSR-Cp | 102128 | 102137 | TTCAA | 2 | trnA-UGC | (Intron)trnA-UGC [trnA-UGC]\| |
| c80000010 | ExtendedSSR | 8SSR-Cp | 102352 | 102367 | TTTTGAGA | 2 | trnA-UGC | (Intron)trnA-UGC [trnA-UGC]\| |
| cD0000008 | SSR | DiSSR-Cp | 103927 | 103936 | CG | 5 | rrn23 | rrn23 [rrn23]\| |
| cH0000107 | PotentialSSR | HexaSSR-Cp | 103950 | 103961 | GAAGCG | 2 | rrn23 | rrn23 [rrn23]\| |
| cTe0000007 | SSR | TetraSSR-Cp | 104344 | 104355 | AGGT | 3 | rrn23 | rrn23 [rrn23]\| |
| cP0000223 | PotentialSSR | PentaSSR-Cp | 105437 | 105446 | GCGGA | 2 | rrn23 | rrn23 [rrn23]\| |
| cP0000224 | PotentialSSR | PentaSSR-Cp | 105735 | 105744 | ATCCA | 2 |  |  |
| c70000024 | ExtendedSSR | 7SSR-Cp | 105807 | 105820 | AAAAACC | 2 |  |  |
| cH0000108 | PotentialSSR | HexaSSR-Cp | 105837 | 105848 | TCTATC | 2 |  |  |
| cH0000109 | PotentialSSR | HexaSSR-Cp | 106214 | 106225 | TTCTTA | 2 |  |  |
| cP0000225 | PotentialSSR | PentaSSR-Cp | 106511 | 106520 | AGTGG | 2 |  |  |
| cM0000021 | SSR | MonoSSR-Cp | 106555 | 106564 | T | 10 |  |  |
| cH0000110 | PotentialSSR | HexaSSR-Cp | 106652 | 106663 | CAAGTA | 2 |  |  |
| cP0000226 | PotentialSSR | PentaSSR-Cp | 106668 | 106677 | TAGCA | 2 |  |  |
| cP0000227 | PotentialSSR | PentaSSR-Cp | 106692 | 106701 | GTCAT | 2 |  |  |
| c70000025 | ExtendedSSR | 7SSR-Cp | 106705 | 106718 | TATGTTT | 2 |  |  |
| cP0000228 | PotentialSSR | PentaSSR-Cp | 106877 | 106886 | CAGAA | 2 |  |  |
| c70000026 | ExtendedSSR | 7SSR-Cp | 106983 | 106996 | AAGAATG | 2 |  |  |
| c90000004 | ExtendedSSR | 9SSR-Cp | 107544 | 107561 | GAAGAAGGA | 2 | ycf1 | YP_009380260.1 [ycf1]\| |
| cH0000112 | PotentialSSR | HexaSSR-Cp | 108166 | 108177 | TAGAAA | 2 | ycf1 | YP_009380260.1 [ycf1]\| |
| cH0000113 | PotentialSSR | HexaSSR-Cp | 108233 | 108244 | TCCTTC | 2 | ycf1 | YP_009380260.1 [ycf1]\| |
| cP0000229 | PotentialSSR | PentaSSR-Cp | 108256 | 108265 | AAGAA | 2 | ycf1 | YP_009380260.1 [ycf1]\| |
| cH0000114 | PotentialSSR | HexaSSR-Cp | 108275 | 108286 | CAAAAT | 2 | ycf1 | YP_009380260.1 [ycf1]\| |
| cP0000230 | PotentialSSR | PentaSSR-Cp | 108322 | 108331 | ACAAA | 2 | ycf1 | YP_009380260.1 [ycf1]\| |
| cP0000231 | PotentialSSR | PentaSSR-Cp | 108547 | 108556 | GAAAT | 2 | ycf1 | YP_009380260.1 [ycf1]\| |
| cM0000022 | SSR | MonoSSR-Cp | 108733 | 108742 | A | 10 | ycf1 | YP_009380260.1 [ycf1]\| |
| cP0000232 | PotentialSSR | PentaSSR-Cp | 108847 | 108856 | AAAAT | 2 | ycf1,ndhF | YP_009380260.1 [ycf1]\|YP_009380261.1 [ndhF]\| |
| cH0000115 | PotentialSSR | HexaSSR-Cp | 110005 | 110016 | AGATCC | 2 | ndhF | YP_009380261.1 [ndhF]\| |
| c70000027 | ExtendedSSR | 7SSR-Cp | 110472 | 110485 | CTCGAAA | 2 | ndhF | YP_009380261.1 [ndhF]\| |
| cP0000233 | PotentialSSR | PentaSSR-Cp | 111006 | 111015 | AAAAG | 2 | ndhF | YP_009380261.1 [ndhF]\| |
| cP0000234 | PotentialSSR | PentaSSR-Cp | 111098 | 111107 | TAGAA | 2 |  |  |
| c70000028 | ExtendedSSR | 7SSR-Cp | 111271 | 111284 | TTAAAAC | 2 |  |  |
| cP0000235 | PotentialSSR | PentaSSR-Cp | 111527 | 111536 | TTACT | 2 |  |  |
| cM0000023 | SSR | MonoSSR-Cp | 111925 | 111934 | A | 10 |  |  |
| cM0000024 | SSR | MonoSSR-Cp | 112242 | 112251 | A | 10 |  |  |
| cP0000236 | PotentialSSR | PentaSSR-Cp | 112490 | 112499 | TTTTA | 2 |  |  |
| c70000029 | ExtendedSSR | 7SSR-Cp | 112510 | 112523 | TAAAAGA | 2 |  |  |
| cM0000025 | SSR | MonoSSR-Cp | 112561 | 112570 | A | 10 |  |  |
| cP0000237 | PotentialSSR | PentaSSR-Cp | 112857 | 112866 | AGAAA | 2 |  |  |
| cP0000238 | PotentialSSR | PentaSSR-Cp | 112986 | 112995 | GAAAA | 2 |  |  |
| cM0000026 | SSR | MonoSSR-Cp | 113119 | 113128 | A | 10 |  |  |
| cH0000116 | PotentialSSR | HexaSSR-Cp | 113172 | 113183 | TATGAA | 2 |  |  |
| cH0000117 | PotentialSSR | HexaSSR-Cp | 113432 | 113443 | AATGAA | 2 |  |  |
| cP0000239 | PotentialSSR | PentaSSR-Cp | 114616 | 114625 | TTTTG | 2 |  |  |
| cP0000240 | PotentialSSR | PentaSSR-Cp | 114654 | 114663 | TTTCA | 2 |  |  |
| cH0000118 | PotentialSSR | HexaSSR-Cp | 114752 | 114763 | AATACC | 2 | ndhD | YP_009380264.1 [ndhD]\| |
| cM0000027 | SSR | MonoSSR-Cp | 114818 | 114827 | A | 10 | ndhD | YP_009380264.1 [ndhD]\| |
| cH0000119 | PotentialSSR | HexaSSR-Cp | 115748 | 115759 | TAATTC | 2 | ndhD | YP_009380264.1 [ndhD]\| |
| cH0000120 | PotentialSSR | HexaSSR-Cp | 116600 | 116611 | TCTAGT | 2 |  |  |
| cP0000241 | PotentialSSR | PentaSSR-Cp | 117235 | 117244 | AAGTT | 2 |  |  |
| cP0000242 | PotentialSSR | PentaSSR-Cp | 117535 | 117544 | ATACC | 2 | ndhG | YP_009380267.1 [ndhG]\| |
| c70000030 | ExtendedSSR | 7SSR-Cp | 117747 | 117760 | TAGAATA | 2 | ndhG | YP_009380267.1 [ndhG]\| |
| cP0000243 | PotentialSSR | PentaSSR-Cp | 118022 | 118031 | TAAAG | 2 |  |  |
| cP0000244 | PotentialSSR | PentaSSR-Cp | 118187 | 118196 | TTTAA | 2 |  |  |
| cP0000245 | PotentialSSR | PentaSSR-Cp | 118308 | 118317 | TAATT | 2 | ndhI | YP_009380268.1 [ndhI]\| |
| cH0000121 | PotentialSSR | HexaSSR-Cp | 118950 | 118961 | GAACAA | 2 | ndhA | YP_009380269.1 [ndhA]\| |
| cP0000246 | PotentialSSR | PentaSSR-Cp | 118974 | 118983 | TAATG | 2 | ndhA | YP_009380269.1 [ndhA]\| |
| cP0000247 | PotentialSSR | PentaSSR-Cp | 119127 | 119136 | ATAAA | 2 | ndhA | YP_009380269.1 [ndhA]\| |
| cP0000248 | PotentialSSR | PentaSSR-Cp | 119355 | 119364 | AAGAT | 2 | ndhA | (Intron)YP_009380269.1 [ndhA]\| |
| cP0000249 | PotentialSSR | PentaSSR-Cp | 119543 | 119552 | CTATA | 2 | ndhA | (Intron)YP_009380269.1 [ndhA]\| |
| c70000031 | ExtendedSSR | 7SSR-Cp | 119665 | 119678 | TATCAAT | 2 | ndhA | (Intron)YP_009380269.1 [ndhA]\| |
| cM0000028 | SSR | MonoSSR-Cp | 119794 | 119803 | T | 10 | ndhA | (Intron)YP_009380269.1 [ndhA]\| |
| cH0000122 | PotentialSSR | HexaSSR-Cp | 119808 | 119819 | CTATTA | 2 | ndhA | (Intron)YP_009380269.1 [ndhA]\| |
| cP0000250 | PotentialSSR | PentaSSR-Cp | 121259 | 121268 | CATTC | 2 | ndhH | YP_009380270.1 [ndhH]\| |
| cH0000123 | PotentialSSR | HexaSSR-Cp | 122268 | 122279 | ATAATT | 2 |  |  |
| cP0000251 | PotentialSSR | PentaSSR-Cp | 122673 | 122682 | TTTAT | 2 |  |  |
| cH0000124 | PotentialSSR | HexaSSR-Cp | 122958 | 122969 | AATTTT | 2 | ycf1 | YP_009380272.1 [ycf1]\| |
| cP0000252 | PotentialSSR | PentaSSR-Cp | 123398 | 123407 | TTCTT | 2 | ycf1 | YP_009380272.1 [ycf1]\| |
| cM0000029 | SSR | MonoSSR-Cp | 123977 | 123988 | T | 12 | ycf1 | YP_009380272.1 [ycf1]\| |
| cH0000125 | PotentialSSR | HexaSSR-Cp | 124344 | 124355 | CTATAT | 2 | ycf1 | YP_009380272.1 [ycf1]\| |
| cH0000126 | PotentialSSR | HexaSSR-Cp | 124442 | 124453 | CAATAA | 2 | ycf1 | YP_009380272.1 [ycf1]\| |
| cTe0000008 | SSR | TetraSSR-Cp | 124476 | 124487 | TAAT | 3 | ycf1 | YP_009380272.1 [ycf1]\| |
| cM0000030 | SSR | MonoSSR-Cp | 125522 | 125532 | A | 11 | ycf1 | YP_009380272.1 [ycf1]\| |
| cP0000253 | PotentialSSR | PentaSSR-Cp | 125687 | 125696 | AAAAC | 2 | ycf1 | YP_009380272.1 [ycf1]\| |
| cP0000254 | PotentialSSR | PentaSSR-Cp | 127023 | 127032 | ATTTT | 2 | ycf1 | YP_009380272.1 [ycf1]\| |
| cM0000031 | SSR | MonoSSR-Cp | 127137 | 127146 | T | 10 | ycf1 | YP_009380272.1 [ycf1]\| |
| cP0000255 | PotentialSSR | PentaSSR-Cp | 127322 | 127331 | CATTT | 2 | ycf1 | YP_009380272.1 [ycf1]\| |
| cP0000256 | PotentialSSR | PentaSSR-Cp | 127548 | 127557 | TTTGT | 2 | ycf1 | YP_009380272.1 [ycf1]\| |
| cH0000127 | PotentialSSR | HexaSSR-Cp | 127593 | 127604 | ATTTTG | 2 | ycf1 | YP_009380272.1 [ycf1]\| |
| cP0000257 | PotentialSSR | PentaSSR-Cp | 127614 | 127623 | TTCTT | 2 | ycf1 | YP_009380272.1 [ycf1]\| |
| cH0000128 | PotentialSSR | HexaSSR-Cp | 127635 | 127646 | GAAGGA | 2 | ycf1 | YP_009380272.1 [ycf1]\| |
| cH0000129 | PotentialSSR | HexaSSR-Cp | 127702 | 127713 | TTTCTA | 2 | ycf1 | YP_009380272.1 [ycf1]\| |
| c90000005 | ExtendedSSR | 9SSR-Cp | 128318 | 128335 | TCCTTCTTC | 2 | ycf1 | YP_009380272.1 [ycf1]\| |
| c70000032 | ExtendedSSR | 7SSR-Cp | 128881 | 128894 | TTCATTC | 2 |  |  |
| cP0000258 | PotentialSSR | PentaSSR-Cp | 128993 | 129002 | TTCTG | 2 |  |  |
| c70000033 | ExtendedSSR | 7SSR-Cp | 129161 | 129174 | AAACATA | 2 |  |  |
| cP0000259 | PotentialSSR | PentaSSR-Cp | 129178 | 129187 | ATGAC | 2 |  |  |
| cP0000260 | PotentialSSR | PentaSSR-Cp | 129202 | 129211 | TGCTA | 2 |  |  |
| cH0000131 | PotentialSSR | HexaSSR-Cp | 129216 | 129227 | TACTTG | 2 |  |  |
| cM0000032 | SSR | MonoSSR-Cp | 129315 | 129324 | A | 10 |  |  |
| cP0000261 | PotentialSSR | PentaSSR-Cp | 129358 | 129367 | TCCAC | 2 |  |  |
| cH0000132 | PotentialSSR | HexaSSR-Cp | 129653 | 129664 | ATAAGA | 2 |  |  |
| cH0000133 | PotentialSSR | HexaSSR-Cp | 130031 | 130042 | GATAGA | 2 |  |  |
| c70000034 | ExtendedSSR | 7SSR-Cp | 130059 | 130072 | GGTTTTT | 2 |  |  |
| cP0000262 | PotentialSSR | PentaSSR-Cp | 130135 | 130144 | TGGAT | 2 |  |  |
| cP0000263 | PotentialSSR | PentaSSR-Cp | 130433 | 130442 | TCCGC | 2 | rrn23 | rrn23 [rrn23]\| |
| cTe0000009 | SSR | TetraSSR-Cp | 131522 | 131533 | CTAC | 3 | rrn23 | rrn23 [rrn23]\| |
| cH0000134 | PotentialSSR | HexaSSR-Cp | 131918 | 131929 | CGCTTC | 2 | rrn23 | rrn23 [rrn23]\| |
| cD0000009 | SSR | DiSSR-Cp | 131943 | 131952 | CG | 5 | rrn23 | rrn23 [rrn23]\| |
| c80000011 | ExtendedSSR | 8SSR-Cp | 133512 | 133527 | TCTCAAAA | 2 | trnA-UGC | (Intron)trnA-UGC [trnA-UGC]\| |
| cP0000264 | PotentialSSR | PentaSSR-Cp | 133741 | 133750 | ATTGA | 2 | trnA-UGC | (Intron)trnA-UGC [trnA-UGC]\| |
| cP0000265 | PotentialSSR | PentaSSR-Cp | 134061 | 134070 | TTTGT | 2 | trnA-UGC | (Intron)trnA-UGC [trnA-UGC]\| |
| cH0000135 | PotentialSSR | HexaSSR-Cp | 134290 | 134301 | ATTCTT | 2 |  |  |
| cH0000136 | PotentialSSR | HexaSSR-Cp | 135046 | 135057 | TCCATT | 2 | trnI-GAU | (Intron)trnI-GAU [trnI-GAU]\| |
| cH0000137 | PotentialSSR | HexaSSR-Cp | 136501 | 136512 | TCAGTG | 2 | rrn16 | rrn16 [rrn16]\| |
| cH0000138 | PotentialSSR | HexaSSR-Cp | 137376 | 137387 | TCATTC | 2 |  |  |
| cP0000266 | PotentialSSR | PentaSSR-Cp | 137502 | 137511 | TCCCT | 2 | trnV-GAC | trnV-GAC [trnV-GAC]\| |
| cP0000267 | PotentialSSR | PentaSSR-Cp | 137585 | 137594 | TCTTG | 2 |  |  |
| cM0000033 | SSR | MonoSSR-Cp | 137834 | 137845 | A | 12 |  |  |
| cH0000139 | PotentialSSR | HexaSSR-Cp | 137942 | 137953 | TTTCCA | 2 |  |  |
| cH0000140 | PotentialSSR | HexaSSR-Cp | 138088 | 138099 | ATAGTA | 2 |  |  |
| cP0000268 | PotentialSSR | PentaSSR-Cp | 138212 | 138221 | CATTG | 2 |  |  |
| cP0000269 | PotentialSSR | PentaSSR-Cp | 138395 | 138404 | CTAAT | 2 |  |  |
| cP0000270 | PotentialSSR | PentaSSR-Cp | 138414 | 138423 | TAATA | 2 |  |  |
| cP0000271 | PotentialSSR | PentaSSR-Cp | 138512 | 138521 | CAACA | 2 |  |  |
| cH0000141 | PotentialSSR | HexaSSR-Cp | 138756 | 138767 | GGGATA | 2 |  |  |
| cH0000142 | PotentialSSR | HexaSSR-Cp | 138768 | 138779 | GAGGAA | 2 |  |  |
| cH0000143 | PotentialSSR | HexaSSR-Cp | 138813 | 138824 | AAAATC | 2 |  |  |
| cP0000272 | PotentialSSR | PentaSSR-Cp | 139405 | 139414 | GATTC | 2 |  |  |
| cH0000144 | PotentialSSR | HexaSSR-Cp | 139592 | 139603 | TATGGA | 2 |  |  |
| cP0000273 | PotentialSSR | PentaSSR-Cp | 140340 | 140349 | GAACA | 2 |  |  |
| cP0000274 | PotentialSSR | PentaSSR-Cp | 140445 | 140454 | TTTCT | 2 |  |  |
| cH0000145 | PotentialSSR | HexaSSR-Cp | 140509 | 140520 | TAAGAA | 2 |  |  |
| cP0000275 | PotentialSSR | PentaSSR-Cp | 141578 | 141587 | TCTTT | 2 | ndhB | (Intron)YP_009380274.1 [ndhB]\| |
| cP0000276 | PotentialSSR | PentaSSR-Cp | 141778 | 141787 | AATCA | 2 | ndhB | (Intron)YP_009380274.1 [ndhB]\| |
| cH0000146 | PotentialSSR | HexaSSR-Cp | 142072 | 142083 | GACTCT | 2 | ndhB | YP_009380274.1 [ndhB]\| |
| cH0000147 | PotentialSSR | HexaSSR-Cp | 142129 | 142140 | GCTTCA | 2 | ndhB | YP_009380274.1 [ndhB]\| |
| cP0000277 | PotentialSSR | PentaSSR-Cp | 143093 | 143102 | TTCCA | 2 |  |  |
| cP0000278 | PotentialSSR | PentaSSR-Cp | 143229 | 143238 | ATAAG | 2 |  |  |
| cH0000148 | PotentialSSR | HexaSSR-Cp | 143542 | 143553 | TTGGAA | 2 |  |  |
| cP0000279 | PotentialSSR | PentaSSR-Cp | 143719 | 143728 | CGTAA | 2 |  |  |
| cP0000280 | PotentialSSR | PentaSSR-Cp | 143964 | 143973 | AACAA | 2 |  |  |
| cP0000281 | PotentialSSR | PentaSSR-Cp | 143992 | 144001 | AACTT | 2 |  |  |
| cH0000149 | PotentialSSR | HexaSSR-Cp | 144484 | 144495 | ATATAG | 2 | ycf2 | YP_009380275.1 [ycf2]\| |
| cH0000150 | PotentialSSR | HexaSSR-Cp | 145084 | 145095 | CTTCTA | 2 | ycf2 | YP_009380275.1 [ycf2]\| |
| cP0000282 | PotentialSSR | PentaSSR-Cp | 145591 | 145600 | TTTTC | 2 | ycf2 | YP_009380275.1 [ycf2]\| |
| cH0000151 | PotentialSSR | HexaSSR-Cp | 145970 | 145981 | CAGCTC | 2 | ycf2 | YP_009380275.1 [ycf2]\| |
| cP0000283 | PotentialSSR | PentaSSR-Cp | 146614 | 146623 | CAATC | 2 | ycf2 | YP_009380275.1 [ycf2]\| |
| cH0000152 | PotentialSSR | HexaSSR-Cp | 146634 | 146645 | CTTTTT | 2 | ycf2 | YP_009380275.1 [ycf2]\| |
| cH0000153 | PotentialSSR | HexaSSR-Cp | 146813 | 146824 | GGGACC | 2 | ycf2 | YP_009380275.1 [ycf2]\| |
| cP0000284 | PotentialSSR | PentaSSR-Cp | 147592 | 147601 | TTGAA | 2 | ycf2 | YP_009380275.1 [ycf2]\| |
| cP0000285 | PotentialSSR | PentaSSR-Cp | 147757 | 147766 | GATCG | 2 | ycf2 | YP_009380275.1 [ycf2]\| |
| c90000006 | ExtendedSSR | 9SSR-Cp | 148596 | 148613 | AAATGTTCC | 2 | ycf2 | YP_009380275.1 [ycf2]\| |
| cP0000286 | PotentialSSR | PentaSSR-Cp | 149883 | 149892 | CGGAT | 2 | ycf2 | YP_009380275.1 [ycf2]\| |
| cH0000155 | PotentialSSR | HexaSSR-Cp | 149934 | 149945 | TCTTTC | 2 | ycf2 | YP_009380275.1 [ycf2]\| |
| cP0000287 | PotentialSSR | PentaSSR-Cp | 150601 | 150610 | ATTTC | 2 |  |  |
| cP0000288 | PotentialSSR | PentaSSR-Cp | 150818 | 150827 | TCATA | 2 |  |  |
| cP0000289 | PotentialSSR | PentaSSR-Cp | 151176 | 151185 | TATTC | 2 |  |  |
| cP0000290 | PotentialSSR | PentaSSR-Cp | 151196 | 151205 | AAAGA | 2 |  |  |
| cP0000291 | PotentialSSR | PentaSSR-Cp | 151297 | 151306 | AATCC | 2 | rpl2 | YP_009380276.1 [rpl2]\| |
| cP0000292 | PotentialSSR | PentaSSR-Cp | 151646 | 151655 | ACATA | 2 | rpl2 | YP_009380276.1 [rpl2]\| |
| cH0000156 | PotentialSSR | HexaSSR-Cp | 152059 | 152070 | GAAAAT | 2 |  |  |

**Supplementary Table 4. List of SSRs identified in CCANG01 of *C. album***

| **Name** | **SSR type** | **Type** | **Cooridnation** | | **Unit sequence** | **Repeat number** | **Genes** | **Annotation** |
| --- | --- | --- | --- | --- | --- | --- | --- | --- |
| cH0000001 | PotentialSSR | HexaSSR-Cp | 113 | 124 | GTAAAG | 2 |  |  |
| cH0000002 | PotentialSSR | HexaSSR-Cp | 1240 | 1251 | GCTTTC | 2 | psbA | YP_009380194.1 [psbA]\| |
| cP0000001 | PotentialSSR | PentaSSR-Cp | 1292 | 1301 | ATTTA | 2 |  |  |
| c70000001 | ExtendedSSR | 7SSR-Cp | 1454 | 1467 | AGAAAAT | 2 |  |  |
| c70000002 | ExtendedSSR | 7SSR-Cp | 1696 | 1709 | AGTAGAA | 2 | trnK-UUU | (Intron)trnK-UUU [trnK-UUU]\| |
| cH0000003 | PotentialSSR | HexaSSR-Cp | 1748 | 1759 | AATTTC | 2 | trnK-UUU | (Intron)trnK-UUU [trnK-UUU]\| |
| c90000001 | ExtendedSSR | 9SSR-Cp | 2942 | 2959 | TTTCTTTTT | 2 | matK,trnK-UUU | YP_009380195.1 [matK]\|(Intron)trnK-UUU [trnK-UUU]\| |
| cH0000004 | PotentialSSR | HexaSSR-Cp | 2949 | 2960 | TTTTTC | 2 | matK,trnK-UUU | YP_009380195.1 [matK]\|(Intron)trnK-UUU [trnK-UUU]\| |
| cM0000001 | SSR | MonoSSR-Cp | 3483 | 3493 | T | 11 | trnK-UUU | (Intron)trnK-UUU [trnK-UUU]\| |
| cP0000002 | PotentialSSR | PentaSSR-Cp | 4093 | 4102 | TATGT | 2 |  |  |
| cP0000003 | PotentialSSR | PentaSSR-Cp | 4124 | 4133 | AATGG | 2 |  |  |
| cP0000004 | PotentialSSR | PentaSSR-Cp | 4202 | 4211 | CAGAT | 2 |  |  |
| cP0000005 | PotentialSSR | PentaSSR-Cp | 4355 | 4364 | CATTT | 2 |  |  |
| cH0000005 | PotentialSSR | HexaSSR-Cp | 4442 | 4453 | AATATT | 2 |  |  |
| cTe0000001 | SSR | TetraSSR-Cp | 4464 | 4475 | TTTA | 3 |  |  |
| cH0000006 | PotentialSSR | HexaSSR-Cp | 4479 | 4490 | TTATTT | 2 |  |  |
| cP0000006 | PotentialSSR | PentaSSR-Cp | 4487 | 4496 | ATTTA | 2 |  |  |
| c70000003 | ExtendedSSR | 7SSR-Cp | 4569 | 4582 | AATATAT | 2 |  |  |
| cP0000007 | PotentialSSR | PentaSSR-Cp | 4572 | 4581 | ATATA | 2 |  |  |
| cT0000001 | SSR | TriSSR-Cp | 4613 | 4624 | ATA | 4 |  |  |
| cP0000008 | PotentialSSR | PentaSSR-Cp | 4970 | 4979 | TAGAT | 2 | rps16 | (Intron)YP_009380196.1 [rps16]\| |
| cP0000009 | PotentialSSR | PentaSSR-Cp | 5204 | 5213 | CATTT | 2 | rps16 | (Intron)YP_009380196.1 [rps16]\| |
| cH0000007 | PotentialSSR | HexaSSR-Cp | 5274 | 5285 | ATCCAA | 2 | rps16 | (Intron)YP_009380196.1 [rps16]\| |
| c70000004 | ExtendedSSR | 7SSR-Cp | 5294 | 5307 | ACAATTA | 2 | rps16 | (Intron)YP_009380196.1 [rps16]\| |
| cH0000008 | PotentialSSR | HexaSSR-Cp | 5332 | 5343 | ATCTTT | 2 | rps16 | (Intron)YP_009380196.1 [rps16]\| |
| cM0000002 | SSR | MonoSSR-Cp | 5341 | 5351 | T | 11 | rps16 | (Intron)YP_009380196.1 [rps16]\| |
| cP0000010 | PotentialSSR | PentaSSR-Cp | 5441 | 5450 | AGAAT | 2 | rps16 | (Intron)YP_009380196.1 [rps16]\| |
| cP0000011 | PotentialSSR | PentaSSR-Cp | 5997 | 6006 | AATGC | 2 |  |  |
| cP0000012 | SSR | PentaSSR-Cp | 6080 | 6094 | TTAAT | 3 |  |  |
| cP0000013 | PotentialSSR | PentaSSR-Cp | 6190 | 6199 | TATAT | 2 |  |  |
| cH0000009 | PotentialSSR | HexaSSR-Cp | 6467 | 6478 | GCTCTG | 2 |  |  |
| cP0000014 | PotentialSSR | PentaSSR-Cp | 6555 | 6564 | TTCTA | 2 |  |  |
| cP0000015 | PotentialSSR | PentaSSR-Cp | 6629 | 6638 | GATTC | 2 |  |  |
| cM0000003 | SSR | MonoSSR-Cp | 6787 | 6797 | A | 11 |  |  |
| cM0000004 | SSR | MonoSSR-Cp | 6853 | 6862 | A | 10 |  |  |
| c80000001 | ExtendedSSR | 8SSR-Cp | 7100 | 7115 | ATTCTAAT | 2 |  |  |
| cH0000010 | PotentialSSR | HexaSSR-Cp | 7345 | 7356 | TTACAA | 2 |  |  |
| cP0000016 | PotentialSSR | PentaSSR-Cp | 7398 | 7407 | TCAAA | 2 |  |  |
| cH0000011 | PotentialSSR | HexaSSR-Cp | 7403 | 7414 | TCAAAT | 2 |  |  |
| cP0000017 | PotentialSSR | PentaSSR-Cp | 7608 | 7617 | TTCTT | 2 |  |  |
| cP0000018 | PotentialSSR | PentaSSR-Cp | 7640 | 7649 | ATTCT | 2 |  |  |
| cM0000005 | SSR | MonoSSR-Cp | 7729 | 7739 | A | 11 |  |  |
| cP0000019 | PotentialSSR | PentaSSR-Cp | 7895 | 7904 | GAAAA | 2 |  |  |
| c70000005 | ExtendedSSR | 7SSR-Cp | 7983 | 7996 | ATATATA | 2 |  |  |
| cP0000020 | PotentialSSR | PentaSSR-Cp | 7985 | 7994 | ATATA | 2 |  |  |
| cH0000012 | PotentialSSR | HexaSSR-Cp | 8065 | 8076 | AGATAA | 2 |  |  |
| cP0000021 | PotentialSSR | PentaSSR-Cp | 8133 | 8142 | TAAAA | 2 |  |  |
| c70000006 | ExtendedSSR | 7SSR-Cp | 8134 | 8147 | AAAATAA | 2 |  |  |
| cP0000022 | PotentialSSR | PentaSSR-Cp | 8209 | 8218 | AAAGA | 2 |  |  |
| c70000007 | ExtendedSSR | 7SSR-Cp | 8213 | 8226 | AAAAGAA | 2 |  |  |
| cP0000023 | PotentialSSR | PentaSSR-Cp | 8264 | 8273 | GGCCT | 2 |  |  |
| cH0000013 | PotentialSSR | HexaSSR-Cp | 8342 | 8353 | TTTGAT | 2 |  |  |
| cH0000014 | PotentialSSR | HexaSSR-Cp | 8853 | 8864 | ATTGAA | 2 | trnG-UCC | (Intron)trnG-UCC [trnG-UCC]\| |
| cP0000024 | PotentialSSR | PentaSSR-Cp | 8858 | 8867 | AATTG | 2 | trnG-UCC | (Intron)trnG-UCC [trnG-UCC]\| |
| cP0000025 | PotentialSSR | PentaSSR-Cp | 9223 | 9232 | TTTTG | 2 | trnG-UCC | (Intron)trnG-UCC [trnG-UCC]\| |
| cP0000026 | PotentialSSR | PentaSSR-Cp | 9248 | 9257 | CTTAA | 2 | trnG-UCC | (Intron)trnG-UCC [trnG-UCC]\| |
| cH0000015 | PotentialSSR | HexaSSR-Cp | 9251 | 9262 | AACTTA | 2 | trnG-UCC | (Intron)trnG-UCC [trnG-UCC]\| |
| cP0000027 | PotentialSSR | PentaSSR-Cp | 9281 | 9290 | TTTTC | 2 | trnG-UCC | (Intron)trnG-UCC [trnG-UCC]\| |
| cP0000028 | PotentialSSR | PentaSSR-Cp | 9479 | 9488 | TCTCA | 2 |  |  |
| cP0000029 | PotentialSSR | PentaSSR-Cp | 9501 | 9510 | CAAAA | 2 |  |  |
| cM0000006 | SSR | MonoSSR-Cp | 9527 | 9537 | A | 11 |  |  |
| cH0000016 | PotentialSSR | HexaSSR-Cp | 10443 | 10454 | GCTTGT | 2 | atpA | YP_009380199.1 [atpA]\| |
| cH0000017 | PotentialSSR | HexaSSR-Cp | 10846 | 10857 | CGGGAG | 2 | atpA | YP_009380199.1 [atpA]\| |
| c80000002 | ExtendedSSR | 8SSR-Cp | 11967 | 11982 | AAAAATAG | 2 | atpF | (Intron)YP_009380200.1 [atpF]\| |
| c70000008 | ExtendedSSR | 7SSR-Cp | 12209 | 12222 | TCGGTAT | 2 | atpF | (Intron)YP_009380200.1 [atpF]\| |
| cT0000002 | SSR | TriSSR-Cp | 12315 | 12326 | ATT | 4 | atpF | (Intron)YP_009380200.1 [atpF]\| |
| cTe0000002 | SSR | TetraSSR-Cp | 12880 | 12891 | GGAA | 3 |  |  |
| cP0000030 | PotentialSSR | PentaSSR-Cp | 13028 | 13037 | AGAAA | 2 |  |  |
| cP0000031 | PotentialSSR | PentaSSR-Cp | 13053 | 13062 | TTTCT | 2 |  |  |
| cD0000001 | SSR | DiSSR-Cp | 13324 | 13333 | CA | 5 |  |  |
| cP0000032 | PotentialSSR | PentaSSR-Cp | 13815 | 13824 | AAATA | 2 |  |  |
| cD0000002 | SSR | DiSSR-Cp | 13882 | 13891 | AT | 5 |  |  |
| cH0000018 | PotentialSSR | HexaSSR-Cp | 14255 | 14266 | TAAAGC | 2 | atpI | YP_009380202.1 [atpI]\| |
| cM0000007 | SSR | MonoSSR-Cp | 14707 | 14716 | A | 10 |  |  |
| cH0000019 | PotentialSSR | HexaSSR-Cp | 14755 | 14766 | TTTAAT | 2 |  |  |
| cP0000033 | PotentialSSR | PentaSSR-Cp | 15625 | 15634 | ATTAA | 2 |  |  |
| c80000003 | ExtendedSSR | 8SSR-Cp | 15684 | 15699 | TCTACCGC | 2 |  |  |
| cP0000034 | PotentialSSR | PentaSSR-Cp | 15763 | 15772 | TTTAT | 2 |  |  |
| cP0000035 | PotentialSSR | PentaSSR-Cp | 15776 | 15785 | TAAAT | 2 |  |  |
| cP0000036 | PotentialSSR | PentaSSR-Cp | 16840 | 16849 | CAATT | 2 | rpoC2 | YP_009380204.1 [rpoC2]\| |
| cP0000037 | PotentialSSR | PentaSSR-Cp | 17655 | 17664 | CAAAA | 2 | rpoC2 | YP_009380204.1 [rpoC2]\| |
| cP0000038 | PotentialSSR | PentaSSR-Cp | 17775 | 17784 | TATCT | 2 | rpoC2 | YP_009380204.1 [rpoC2]\| |
| cM0000008 | SSR | MonoSSR-Cp | 17831 | 17843 | T | 13 | rpoC2 | YP_009380204.1 [rpoC2]\| |
| cP0000039 | PotentialSSR | PentaSSR-Cp | 18314 | 18323 | CGATT | 2 | rpoC2 | YP_009380204.1 [rpoC2]\| |
| cP0000040 | PotentialSSR | PentaSSR-Cp | 18343 | 18352 | ATCCT | 2 | rpoC2 | YP_009380204.1 [rpoC2]\| |
| cH0000020 | PotentialSSR | HexaSSR-Cp | 18456 | 18467 | TTGATC | 2 | rpoC2 | YP_009380204.1 [rpoC2]\| |
| cH0000021 | PotentialSSR | HexaSSR-Cp | 18828 | 18839 | ACGTGT | 2 | rpoC2 | YP_009380204.1 [rpoC2]\| |
| cP0000041 | PotentialSSR | PentaSSR-Cp | 19040 | 19049 | CATAA | 2 | rpoC2 | YP_009380204.1 [rpoC2]\| |
| cD0000003 | SSR | DiSSR-Cp | 19183 | 19192 | AT | 5 | rpoC2 | YP_009380204.1 [rpoC2]\| |
| cH0000022 | PotentialSSR | HexaSSR-Cp | 21522 | 21533 | CAAATC | 2 | rpoC1 | YP_009380205.1 [rpoC1]\| |
| cP0000042 | PotentialSSR | PentaSSR-Cp | 21636 | 21645 | GGATT | 2 | rpoC1 | YP_009380205.1 [rpoC1]\| |
| cP0000043 | PotentialSSR | PentaSSR-Cp | 21725 | 21734 | ATCCT | 2 | rpoC1 | (Intron)YP_009380205.1 [rpoC1]\| |
| cP0000044 | PotentialSSR | PentaSSR-Cp | 21919 | 21928 | TTCTT | 2 | rpoC1 | (Intron)YP_009380205.1 [rpoC1]\| |
| cP0000045 | PotentialSSR | PentaSSR-Cp | 21951 | 21960 | AATTA | 2 | rpoC1 | (Intron)YP_009380205.1 [rpoC1]\| |
| cP0000046 | PotentialSSR | PentaSSR-Cp | 22135 | 22144 | TTAAA | 2 | rpoC1 | (Intron)YP_009380205.1 [rpoC1]\| |
| cH0000023 | PotentialSSR | HexaSSR-Cp | 22240 | 22251 | ACAAAA | 2 | rpoC1 | (Intron)YP_009380205.1 [rpoC1]\| |
| cM0000009 | SSR | MonoSSR-Cp | 25508 | 25517 | T | 10 | rpoB | YP_009380206.1 [rpoB]\| |
| cH0000024 | PotentialSSR | HexaSSR-Cp | 25951 | 25962 | TCTTTT | 2 | rpoB | YP_009380206.1 [rpoB]\| |
| cH0000025 | PotentialSSR | HexaSSR-Cp | 26423 | 26434 | TACTTA | 2 |  |  |
| cP0000047 | PotentialSSR | PentaSSR-Cp | 26430 | 26439 | ACTTA | 2 |  |  |
| cP0000048 | PotentialSSR | PentaSSR-Cp | 26723 | 26732 | TTGTA | 2 |  |  |
| cH0000026 | PotentialSSR | HexaSSR-Cp | 26884 | 26895 | AGAAAA | 2 |  |  |
| cP0000049 | PotentialSSR | PentaSSR-Cp | 27100 | 27109 | GAATC | 2 |  |  |
| cP0000050 | PotentialSSR | PentaSSR-Cp | 27560 | 27569 | TTCCC | 2 |  |  |
| cP0000051 | PotentialSSR | PentaSSR-Cp | 27689 | 27698 | CTGAG | 2 |  |  |
| cH0000027 | PotentialSSR | HexaSSR-Cp | 27801 | 27812 | TTCATA | 2 |  |  |
| cP0000052 | PotentialSSR | PentaSSR-Cp | 27933 | 27942 | TTTAG | 2 |  |  |
| cH0000028 | PotentialSSR | HexaSSR-Cp | 27959 | 27970 | TATCAT | 2 |  |  |
| cP0000053 | PotentialSSR | PentaSSR-Cp | 28089 | 28098 | GAATT | 2 |  |  |
| cP0000054 | PotentialSSR | PentaSSR-Cp | 28132 | 28141 | AATTC | 2 |  |  |
| cP0000055 | PotentialSSR | PentaSSR-Cp | 28192 | 28201 | ATTTC | 2 |  |  |
| cP0000056 | PotentialSSR | PentaSSR-Cp | 28260 | 28269 | TCAAT | 2 |  |  |
| cP0000057 | PotentialSSR | PentaSSR-Cp | 28286 | 28295 | TTGGC | 2 |  |  |
| cP0000058 | PotentialSSR | PentaSSR-Cp | 28344 | 28353 | ACTTT | 2 |  |  |
| c70000009 | ExtendedSSR | 7SSR-Cp | 28411 | 28424 | TTATAGT | 2 |  |  |
| cP0000059 | PotentialSSR | PentaSSR-Cp | 28448 | 28457 | CTAAT | 2 |  |  |
| cP0000060 | PotentialSSR | PentaSSR-Cp | 28824 | 28833 | TATCA | 2 |  |  |
| cH0000029 | PotentialSSR | HexaSSR-Cp | 29049 | 29060 | CAAAAA | 2 |  |  |
| cM0000010 | SSR | MonoSSR-Cp | 29375 | 29384 | A | 10 |  |  |
| cP0000061 | PotentialSSR | PentaSSR-Cp | 29753 | 29762 | ATAAA | 2 |  |  |
| c80000004 | ExtendedSSR | 8SSR-Cp | 29772 | 29787 | TTTTCTTT | 2 |  |  |
| cP0000062 | PotentialSSR | PentaSSR-Cp | 29829 | 29838 | ACCAT | 2 |  |  |
| cH0000030 | PotentialSSR | HexaSSR-Cp | 29869 | 29880 | AATTTC | 2 |  |  |
| cTe0000003 | SSR | TetraSSR-Cp | 29888 | 29899 | AAAT | 3 |  |  |
| cP0000063 | PotentialSSR | PentaSSR-Cp | 30107 | 30116 | TCAAT | 2 |  |  |
| cP0000064 | PotentialSSR | PentaSSR-Cp | 30158 | 30167 | TACCC | 2 | trnE-UUC | trnE-UUC [trnE-UUC]\| |
| cH0000031 | PotentialSSR | HexaSSR-Cp | 30362 | 30373 | ATTTCA | 2 |  |  |
| cH0000032 | PotentialSSR | HexaSSR-Cp | 30500 | 30511 | CTAATA | 2 |  |  |
| cP0000065 | PotentialSSR | PentaSSR-Cp | 30644 | 30653 | GTATA | 2 |  |  |
| cP0000066 | PotentialSSR | PentaSSR-Cp | 30956 | 30965 | ATTAT | 2 |  |  |
| cP0000067 | PotentialSSR | PentaSSR-Cp | 31029 | 31038 | TGGAA | 2 |  |  |
| c70000010 | ExtendedSSR | 7SSR-Cp | 31490 | 31503 | GTTTATT | 2 |  |  |
| cH0000033 | PotentialSSR | HexaSSR-Cp | 31567 | 31578 | GTGAAA | 2 |  |  |
| cP0000068 | PotentialSSR | PentaSSR-Cp | 31743 | 31752 | AAAGA | 2 |  |  |
| cP0000069 | PotentialSSR | PentaSSR-Cp | 31950 | 31959 | TAAAT | 2 |  |  |
| cP0000070 | PotentialSSR | PentaSSR-Cp | 32113 | 32122 | CGTTT | 2 | psbD | YP_009380209.1 [psbD]\| |
| cP0000071 | PotentialSSR | PentaSSR-Cp | 32743 | 32752 | AACCC | 2 | psbD | YP_009380209.1 [psbD]\| |
| cP0000072 | PotentialSSR | PentaSSR-Cp | 33836 | 33845 | GTCTG | 2 | psbC | YP_009380210.1 [psbC]\| |
| cH0000034 | PotentialSSR | HexaSSR-Cp | 33972 | 33983 | CTCAAG | 2 | psbC | YP_009380210.1 [psbC]\| |
| cP0000073 | PotentialSSR | PentaSSR-Cp | 34264 | 34273 | GGGTG | 2 | psbC | YP_009380210.1 [psbC]\| |
| cH0000035 | PotentialSSR | HexaSSR-Cp | 34390 | 34401 | TGCAGC | 2 | psbC | YP_009380210.1 [psbC]\| |
| c70000011 | ExtendedSSR | 7SSR-Cp | 34513 | 34526 | TACATAT | 2 |  |  |
| cP0000074 | PotentialSSR | PentaSSR-Cp | 34591 | 34600 | TAATT | 2 |  |  |
| cH0000036 | PotentialSSR | HexaSSR-Cp | 34822 | 34833 | TTATTC | 2 |  |  |
| cP0000075 | PotentialSSR | PentaSSR-Cp | 34924 | 34933 | TATAT | 2 |  |  |
| c70000012 | ExtendedSSR | 7SSR-Cp | 34964 | 34977 | TTAATTA | 2 |  |  |
| cP0000076 | PotentialSSR | PentaSSR-Cp | 35300 | 35309 | TGGAT | 2 | psbZ | YP_009380211.1 [psbZ]\| |
| cP0000077 | PotentialSSR | PentaSSR-Cp | 35468 | 35477 | AAACA | 2 |  |  |
| cP0000078 | PotentialSSR | PentaSSR-Cp | 35631 | 35640 | TATAT | 2 |  |  |
| cH0000037 | PotentialSSR | HexaSSR-Cp | 35797 | 35808 | GTTATA | 2 |  |  |
| cP0000079 | PotentialSSR | PentaSSR-Cp | 35820 | 35829 | TAGTG | 2 |  |  |
| cP0000080 | PotentialSSR | PentaSSR-Cp | 35923 | 35932 | TATTT | 2 |  |  |
| cP0000081 | PotentialSSR | PentaSSR-Cp | 36834 | 36843 | CCACG | 2 | psaB | YP_009380213.1 [psaB]\| |
| cP0000082 | PotentialSSR | PentaSSR-Cp | 36983 | 36992 | CCATC | 2 | psaB | YP_009380213.1 [psaB]\| |
| cP0000083 | PotentialSSR | PentaSSR-Cp | 38262 | 38271 | TGTCC | 2 | psaB | YP_009380213.1 [psaB]\| |
| cP0000084 | PotentialSSR | PentaSSR-Cp | 38768 | 38777 | ACCAA | 2 | psaB | YP_009380213.1 [psaB]\| |
| cH0000038 | PotentialSSR | HexaSSR-Cp | 39194 | 39205 | TAATAG | 2 | psaA | YP_009380214.1 [psaA]\| |
| cP0000085 | PotentialSSR | PentaSSR-Cp | 39943 | 39952 | ATGTG | 2 | psaA | YP_009380214.1 [psaA]\| |
| cP0000086 | PotentialSSR | PentaSSR-Cp | 41580 | 41589 | TATTT | 2 |  |  |
| cH0000039 | PotentialSSR | HexaSSR-Cp | 41758 | 41769 | TCTTTA | 2 |  |  |
| cP0000087 | PotentialSSR | PentaSSR-Cp | 41852 | 41861 | TTTTA | 2 |  |  |
| cM0000011 | SSR | MonoSSR-Cp | 41891 | 41900 | A | 10 |  |  |
| cP0000088 | PotentialSSR | PentaSSR-Cp | 42433 | 42442 | AAAGA | 2 | ycf3 | (Intron)YP_009380215.1 [ycf3]\| |
| cP0000089 | PotentialSSR | PentaSSR-Cp | 42570 | 42579 | TTCTT | 2 | ycf3 | (Intron)YP_009380215.1 [ycf3]\| |
| cM0000012 | SSR | MonoSSR-Cp | 42643 | 42652 | T | 10 | ycf3 | (Intron)YP_009380215.1 [ycf3]\| |
| cP0000090 | PotentialSSR | PentaSSR-Cp | 43144 | 43153 | AATAT | 2 | ycf3 | (Intron)YP_009380215.1 [ycf3]\| |
| cP0000091 | PotentialSSR | PentaSSR-Cp | 43819 | 43828 | AAATC | 2 | ycf3 | (Intron)YP_009380215.1 [ycf3]\| |
| cP0000092 | PotentialSSR | PentaSSR-Cp | 44028 | 44037 | GTACA | 2 |  |  |
| cP0000093 | PotentialSSR | PentaSSR-Cp | 44117 | 44126 | TATTT | 2 |  |  |
| cP0000094 | PotentialSSR | PentaSSR-Cp | 44204 | 44213 | CAAAT | 2 |  |  |
| cP0000095 | PotentialSSR | PentaSSR-Cp | 44218 | 44227 | TAGTA | 2 |  |  |
| cP0000096 | PotentialSSR | PentaSSR-Cp | 44271 | 44280 | GATCA | 2 |  |  |
| cP0000097 | PotentialSSR | PentaSSR-Cp | 44340 | 44349 | AATAG | 2 |  |  |
| cP0000098 | PotentialSSR | PentaSSR-Cp | 44517 | 44526 | GATTC | 2 |  |  |
| cTe0000004 | SSR | TetraSSR-Cp | 44532 | 44543 | AATT | 3 |  |  |
| cP0000099 | PotentialSSR | PentaSSR-Cp | 44552 | 44561 | TATAT | 2 |  |  |
| cD0000004 | SSR | DiSSR-Cp | 44624 | 44633 | AT | 5 |  |  |
| cP0000100 | PotentialSSR | PentaSSR-Cp | 44652 | 44661 | TTTTA | 2 |  |  |
| cP0000101 | PotentialSSR | PentaSSR-Cp | 44703 | 44712 | ATTAC | 2 |  |  |
| cP0000102 | PotentialSSR | PentaSSR-Cp | 44751 | 44760 | ATAGT | 2 |  |  |
| cP0000103 | PotentialSSR | PentaSSR-Cp | 44836 | 44845 | ATAGT | 2 |  |  |
| cP0000104 | PotentialSSR | PentaSSR-Cp | 45024 | 45033 | CAAAT | 2 |  |  |
| cP0000105 | PotentialSSR | PentaSSR-Cp | 45200 | 45209 | GGGAT | 2 |  |  |
| cH0000040 | PotentialSSR | HexaSSR-Cp | 45267 | 45278 | TTTTCC | 2 |  |  |
| cH0000041 | PotentialSSR | HexaSSR-Cp | 45282 | 45293 | TGAAAG | 2 |  |  |
| cP0000106 | PotentialSSR | PentaSSR-Cp | 45699 | 45708 | ACCTG | 2 | rps4 | YP_009380216.1 [rps4]\| |
| cD0000005 | SSR | DiSSR-Cp | 45982 | 45995 | TA | 7 |  |  |
| c70000013 | ExtendedSSR | 7SSR-Cp | 46032 | 46045 | TAAATGA | 2 |  |  |
| cP0000107 | PotentialSSR | PentaSSR-Cp | 46040 | 46049 | AAATG | 2 |  |  |
| cP0000108 | PotentialSSR | PentaSSR-Cp | 46131 | 46140 | TATTT | 2 |  |  |
| cH0000042 | PotentialSSR | HexaSSR-Cp | 46160 | 46171 | AAAAAG | 2 |  |  |
| cP0000109 | PotentialSSR | PentaSSR-Cp | 46215 | 46224 | CTTTT | 2 |  |  |
| cP0000110 | PotentialSSR | PentaSSR-Cp | 46507 | 46516 | TCAAA | 2 |  |  |
| c70000014 | ExtendedSSR | 7SSR-Cp | 46511 | 46524 | ATCAAAT | 2 |  |  |
| cP0000111 | PotentialSSR | PentaSSR-Cp | 46585 | 46594 | CGATT | 2 |  |  |
| cP0000112 | PotentialSSR | PentaSSR-Cp | 46821 | 46830 | AATAA | 2 |  |  |
| cP0000113 | PotentialSSR | PentaSSR-Cp | 47410 | 47419 | CTGTG | 2 | trnL-UAA | (Intron)trnL-UAA [trnL-UAA]\| |
| cP0000114 | PotentialSSR | PentaSSR-Cp | 47503 | 47512 | TCTAT | 2 | trnL-UAA | (Intron)trnL-UAA [trnL-UAA]\| |
| cH0000043 | PotentialSSR | HexaSSR-Cp | 47891 | 47902 | TCTTTT | 2 |  |  |
| cP0000115 | PotentialSSR | PentaSSR-Cp | 47894 | 47903 | TTTTC | 2 |  |  |
| cD0000006 | SSR | DiSSR-Cp | 47951 | 47960 | AT | 5 |  |  |
| cP0000116 | PotentialSSR | PentaSSR-Cp | 48265 | 48274 | CACAT | 2 |  |  |
| cH0000044 | PotentialSSR | HexaSSR-Cp | 48394 | 48405 | GAAAAT | 2 |  |  |
| cP0000117 | PotentialSSR | PentaSSR-Cp | 48525 | 48534 | CTTAA | 2 |  |  |
| cP0000118 | PotentialSSR | PentaSSR-Cp | 48544 | 48553 | TCTTT | 2 |  |  |
| c80000005 | ExtendedSSR | 8SSR-Cp | 48546 | 48561 | TTTTCTTT | 2 |  |  |
| cH0000045 | PotentialSSR | HexaSSR-Cp | 49013 | 49024 | CATATA | 2 | ndhJ | YP_009380217.1 [ndhJ]\| |
| c80000006 | ExtendedSSR | 8SSR-Cp | 49170 | 49185 | ATTTGTTT | 2 |  |  |
| cP0000119 | PotentialSSR | PentaSSR-Cp | 49193 | 49202 | TATTT | 2 |  |  |
| cP0000120 | PotentialSSR | PentaSSR-Cp | 49206 | 49215 | TTTAC | 2 |  |  |
| cH0000046 | PotentialSSR | HexaSSR-Cp | 49973 | 49984 | TTTTTC | 2 |  |  |
| cP0000121 | PotentialSSR | PentaSSR-Cp | 50038 | 50047 | TAAAC | 2 | ndhC | YP_009380219.1 [ndhC]\| |
| cP0000122 | PotentialSSR | PentaSSR-Cp | 50600 | 50609 | TTCTT | 2 |  |  |
| cH0000047 | PotentialSSR | HexaSSR-Cp | 50609 | 50620 | TATACA | 2 |  |  |
| cP0000123 | PotentialSSR | PentaSSR-Cp | 50676 | 50685 | AATTC | 2 |  |  |
| cM0000013 | SSR | MonoSSR-Cp | 50689 | 50698 | T | 10 |  |  |
| cP0000124 | PotentialSSR | PentaSSR-Cp | 50733 | 50742 | TTCTA | 2 |  |  |
| cP0000125 | PotentialSSR | PentaSSR-Cp | 50792 | 50801 | ACTTG | 2 |  |  |
| c70000015 | ExtendedSSR | 7SSR-Cp | 52549 | 52562 | AATTTTT | 2 |  |  |
| cP0000126 | PotentialSSR | PentaSSR-Cp | 52687 | 52696 | ATTTG | 2 |  |  |
| cP0000127 | PotentialSSR | PentaSSR-Cp | 52788 | 52797 | TTGAA | 2 |  |  |
| cH0000048 | PotentialSSR | HexaSSR-Cp | 52841 | 52852 | TGTATA | 2 |  |  |
| cP0000128 | PotentialSSR | PentaSSR-Cp | 53068 | 53077 | ATTGA | 2 |  |  |
| cP0000129 | PotentialSSR | PentaSSR-Cp | 53107 | 53116 | AATTG | 2 |  |  |
| cM0000014 | SSR | MonoSSR-Cp | 53198 | 53209 | T | 12 |  |  |
| cH0000049 | PotentialSSR | HexaSSR-Cp | 53220 | 53231 | ATTTTC | 2 |  |  |
| cM0000015 | SSR | MonoSSR-Cp | 53305 | 53314 | A | 10 | atpB | YP_009380221.1 [atpB]\| |
| cP0000130 | PotentialSSR | PentaSSR-Cp | 54481 | 54490 | TTGGA | 2 | atpB | YP_009380221.1 [atpB]\| |
| cH0000050 | PotentialSSR | HexaSSR-Cp | 55178 | 55189 | ATAATC | 2 |  |  |
| cH0000051 | PotentialSSR | HexaSSR-Cp | 55248 | 55259 | TCTTTT | 2 |  |  |
| cH0000052 | PotentialSSR | HexaSSR-Cp | 55286 | 55297 | AAAAAG | 2 |  |  |
| cP0000131 | PotentialSSR | PentaSSR-Cp | 55760 | 55769 | AAACA | 2 | trnV-UAC | (Intron)trnV-UAC [trnV-UAC]\| |
| c70000016 | ExtendedSSR | 7SSR-Cp | 56092 | 56105 | AATGAAT | 2 | trnV-UAC | (Intron)trnV-UAC [trnV-UAC]\| |
| cH0000053 | PotentialSSR | HexaSSR-Cp | 56338 | 56349 | CAATTG | 2 |  |  |
| cP0000132 | PotentialSSR | PentaSSR-Cp | 56399 | 56408 | ATTTG | 2 |  |  |
| cH0000054 | PotentialSSR | HexaSSR-Cp | 56423 | 56434 | TTTGAA | 2 |  |  |
| cP0000133 | PotentialSSR | PentaSSR-Cp | 56522 | 56531 | TTTTC | 2 |  |  |
| cP0000134 | PotentialSSR | PentaSSR-Cp | 58588 | 58597 | TATAT | 2 |  |  |
| cP0000135 | PotentialSSR | PentaSSR-Cp | 58644 | 58653 | CTATA | 2 |  |  |
| cTe0000005 | SSR | TetraSSR-Cp | 58671 | 58682 | TAAT | 3 |  |  |
| cP0000136 | PotentialSSR | PentaSSR-Cp | 58685 | 58694 | TAATA | 2 |  |  |
| cH0000055 | PotentialSSR | HexaSSR-Cp | 58726 | 58737 | TACAAT | 2 |  |  |
| cP0000137 | PotentialSSR | PentaSSR-Cp | 58856 | 58865 | AAAAT | 2 | psaI | YP_009380224.1 [psaI]\| |
| cP0000138 | PotentialSSR | PentaSSR-Cp | 58874 | 58883 | TCGTC | 2 |  |  |
| cP0000139 | PotentialSSR | PentaSSR-Cp | 59014 | 59023 | ACAAT | 2 |  |  |
| cP0000140 | PotentialSSR | PentaSSR-Cp | 59178 | 59187 | TTCAA | 2 |  |  |
| cP0000141 | PotentialSSR | PentaSSR-Cp | 59488 | 59497 | TCGAT | 2 | ycf4 | YP_009380225.1 [ycf4]\| |
| cP0000142 | PotentialSSR | PentaSSR-Cp | 59593 | 59602 | TAGAA | 2 | ycf4 | YP_009380225.1 [ycf4]\| |
| cH0000056 | PotentialSSR | HexaSSR-Cp | 59926 | 59937 | TACAAA | 2 |  |  |
| cP0000143 | PotentialSSR | PentaSSR-Cp | 59939 | 59948 | CAATT | 2 |  |  |
| cP0000144 | PotentialSSR | PentaSSR-Cp | 60079 | 60088 | AATTA | 2 |  |  |
| cP0000145 | PotentialSSR | PentaSSR-Cp | 61044 | 61053 | AAAAG | 2 |  |  |
| c70000017 | ExtendedSSR | 7SSR-Cp | 61108 | 61121 | ATAATCA | 2 |  |  |
| cP0000146 | PotentialSSR | PentaSSR-Cp | 61193 | 61202 | TTATT | 2 |  |  |
| cP0000147 | PotentialSSR | PentaSSR-Cp | 61691 | 61700 | AAAGA | 2 | petA | YP_009380227.1 [petA]\| |
| cP0000148 | PotentialSSR | PentaSSR-Cp | 61962 | 61971 | GAAAA | 2 | petA | YP_009380227.1 [petA]\| |
| cH0000057 | PotentialSSR | HexaSSR-Cp | 62282 | 62293 | ATCAAG | 2 |  |  |
| cH0000058 | PotentialSSR | HexaSSR-Cp | 62298 | 62309 | TAACAA | 2 |  |  |
| cP0000149 | PotentialSSR | PentaSSR-Cp | 62322 | 62331 | CAATT | 2 |  |  |
| cM0000016 | SSR | MonoSSR-Cp | 62356 | 62369 | A | 14 |  |  |
| c80000007 | ExtendedSSR | 8SSR-Cp | 62463 | 62478 | ACTTTTTT | 2 |  |  |
| cH0000059 | PotentialSSR | HexaSSR-Cp | 62670 | 62681 | ATTTTT | 2 |  |  |
| cH0000060 | PotentialSSR | HexaSSR-Cp | 62865 | 62876 | TCTTTC | 2 |  |  |
| cP0000150 | PotentialSSR | PentaSSR-Cp | 62879 | 62888 | TTAAC | 2 |  |  |
| c70000018 | ExtendedSSR | 7SSR-Cp | 63131 | 63144 | TTACTAC | 2 | psbJ | YP_009380228.1 [psbJ]\| |
| cP0000151 | PotentialSSR | PentaSSR-Cp | 63484 | 63493 | ATTCA | 2 | psbL | YP_009380229.1 [psbL]\| |
| cH0000061 | PotentialSSR | HexaSSR-Cp | 63535 | 63546 | ATTCGG | 2 |  |  |
| c80000008 | ExtendedSSR | 8SSR-Cp | 64015 | 64030 | ACGTAAAA | 2 |  |  |
| cH0000062 | PotentialSSR | HexaSSR-Cp | 64351 | 64362 | TATAGA | 2 |  |  |
| cH0000063 | PotentialSSR | HexaSSR-Cp | 64590 | 64601 | AGTCTT | 2 |  |  |
| cP0000152 | PotentialSSR | PentaSSR-Cp | 65146 | 65155 | CTGTA | 2 |  |  |
| cM0000017 | SSR | MonoSSR-Cp | 65261 | 65273 | T | 13 |  |  |
| cP0000153 | PotentialSSR | PentaSSR-Cp | 65465 | 65474 | GATTA | 2 |  |  |
| cP0000154 | PotentialSSR | PentaSSR-Cp | 65482 | 65491 | TTTAG | 2 |  |  |
| cH0000064 | PotentialSSR | HexaSSR-Cp | 65501 | 65512 | TTTCTA | 2 |  |  |
| cP0000155 | PotentialSSR | PentaSSR-Cp | 65599 | 65608 | GAACT | 2 | trnW-CCA | trnW-CCA [trnW-CCA]\| |
| cH0000065 | PotentialSSR | HexaSSR-Cp | 65675 | 65686 | CTATAT | 2 |  |  |
| cP0000156 | PotentialSSR | PentaSSR-Cp | 65882 | 65891 | TTCAA | 2 |  |  |
| cP0000157 | PotentialSSR | PentaSSR-Cp | 65921 | 65930 | CCTTG | 2 |  |  |
| cP0000158 | PotentialSSR | PentaSSR-Cp | 66057 | 66066 | GTAAA | 2 |  |  |
| cP0000159 | PotentialSSR | PentaSSR-Cp | 66169 | 66178 | CTTAG | 2 |  |  |
| cP0000160 | PotentialSSR | PentaSSR-Cp | 66525 | 66534 | TTAGT | 2 |  |  |
| cP0000161 | PotentialSSR | PentaSSR-Cp | 66578 | 66587 | GTTAA | 2 |  |  |
| c70000019 | ExtendedSSR | 7SSR-Cp | 66719 | 66732 | CGAATTG | 2 |  |  |
| cH0000066 | PotentialSSR | HexaSSR-Cp | 67082 | 67093 | AATTTT | 2 |  |  |
| cD0000007 | SSR | DiSSR-Cp | 67107 | 67116 | AT | 5 |  |  |
| c70000020 | ExtendedSSR | 7SSR-Cp | 67174 | 67187 | ATATATA | 2 |  |  |
| cP0000162 | PotentialSSR | PentaSSR-Cp | 67176 | 67185 | ATATA | 2 |  |  |
| cP0000163 | PotentialSSR | PentaSSR-Cp | 67251 | 67260 | ATAGG | 2 |  |  |
| cP0000164 | PotentialSSR | PentaSSR-Cp | 67278 | 67287 | TAAAC | 2 |  |  |
| cTe0000006 | SSR | TetraSSR-Cp | 67762 | 67773 | TTTA | 3 |  |  |
| cP0000165 | PotentialSSR | PentaSSR-Cp | 68325 | 68334 | ATAAA | 2 |  |  |
| cH0000067 | PotentialSSR | HexaSSR-Cp | 68816 | 68827 | ATTTTC | 2 |  |  |
| cP0000166 | PotentialSSR | PentaSSR-Cp | 68934 | 68943 | CTATT | 2 |  |  |
| cH0000068 | PotentialSSR | HexaSSR-Cp | 68981 | 68992 | TTTTGT | 2 |  |  |
| cH0000069 | PotentialSSR | HexaSSR-Cp | 69301 | 69312 | TGGGCT | 2 | clpP | YP_009380239.1 [clpP]\| |
| cP0000167 | PotentialSSR | PentaSSR-Cp | 69531 | 69540 | ACACA | 2 | clpP | (Intron)YP_009380239.1 [clpP]\| |
| cP0000168 | PotentialSSR | PentaSSR-Cp | 69676 | 69685 | ATCGA | 2 | clpP | (Intron)YP_009380239.1 [clpP]\| |
| cH0000070 | PotentialSSR | HexaSSR-Cp | 69687 | 69698 | CAGATC | 2 | clpP | (Intron)YP_009380239.1 [clpP]\| |
| cP0000169 | PotentialSSR | PentaSSR-Cp | 69972 | 69981 | GAAAA | 2 | clpP | (Intron)YP_009380239.1 [clpP]\| |
| cH0000071 | PotentialSSR | HexaSSR-Cp | 70262 | 70273 | ACAAAT | 2 | clpP | YP_009380239.1 [clpP]\| |
| cP0000170 | PotentialSSR | PentaSSR-Cp | 70675 | 70684 | TATCA | 2 | clpP | (Intron)YP_009380239.1 [clpP]\| |
| cH0000072 | PotentialSSR | HexaSSR-Cp | 70715 | 70726 | TTCTTG | 2 | clpP | (Intron)YP_009380239.1 [clpP]\| |
| cH0000073 | PotentialSSR | HexaSSR-Cp | 70925 | 70936 | TTGAAA | 2 | clpP | (Intron)YP_009380239.1 [clpP]\| |
| cH0000074 | PotentialSSR | HexaSSR-Cp | 70992 | 71003 | ATTGGG | 2 | clpP | (Intron)YP_009380239.1 [clpP]\| |
| cP0000171 | PotentialSSR | PentaSSR-Cp | 71561 | 71570 | ATAGA | 2 |  |  |
| cH0000075 | PotentialSSR | HexaSSR-Cp | 71680 | 71691 | CATAGT | 2 |  |  |
| c70000021 | ExtendedSSR | 7SSR-Cp | 71830 | 71843 | CTGGTTG | 2 | psbB | YP_009380240.1 [psbB]\| |
| cH0000076 | PotentialSSR | HexaSSR-Cp | 72194 | 72205 | GTTTTG | 2 | psbB | YP_009380240.1 [psbB]\| |
| cP0000172 | PotentialSSR | PentaSSR-Cp | 73300 | 73309 | TTTGA | 2 |  |  |
| cP0000173 | PotentialSSR | PentaSSR-Cp | 73457 | 73466 | CTCTA | 2 | psbT | YP_009380241.1 [psbT]\| |
| cP0000174 | PotentialSSR | PentaSSR-Cp | 73521 | 73530 | AAATG | 2 | psbT | YP_009380241.1 [psbT]\| |
| cH0000077 | PotentialSSR | HexaSSR-Cp | 74052 | 74063 | ACAAAA | 2 |  |  |
| c90000002 | ExtendedSSR | 9SSR-Cp | 74101 | 74118 | CAATACAAA | 2 |  |  |
| cP0000175 | PotentialSSR | PentaSSR-Cp | 74404 | 74413 | TTTTC | 2 | petB | (Intron)YP_009380244.1 [petB]\| |
| cP0000176 | PotentialSSR | PentaSSR-Cp | 74522 | 74531 | AATAA | 2 | petB | (Intron)YP_009380244.1 [petB]\| |
| cP0000177 | PotentialSSR | PentaSSR-Cp | 74534 | 74543 | CCTAT | 2 | petB | (Intron)YP_009380244.1 [petB]\| |
| cH0000078 | PotentialSSR | HexaSSR-Cp | 74710 | 74721 | ATTATA | 2 | petB | (Intron)YP_009380244.1 [petB]\| |
| cH0000079 | PotentialSSR | HexaSSR-Cp | 74722 | 74733 | TACAAA | 2 | petB | (Intron)YP_009380244.1 [petB]\| |
| cP0000178 | PotentialSSR | PentaSSR-Cp | 75664 | 75673 | ATAGA | 2 | petB | YP_009380244.1 [petB]\| |
| cP0000179 | PotentialSSR | PentaSSR-Cp | 76033 | 76042 | TCTAT | 2 | petD | (Intron)YP_009380245.1 [petD]\| |
| cP0000180 | PotentialSSR | PentaSSR-Cp | 76109 | 76118 | ATAAT | 2 | petD | (Intron)YP_009380245.1 [petD]\| |
| cP0000181 | PotentialSSR | PentaSSR-Cp | 76123 | 76132 | ATTAT | 2 | petD | (Intron)YP_009380245.1 [petD]\| |
| cP0000182 | PotentialSSR | PentaSSR-Cp | 76146 | 76155 | TTATT | 2 | petD | (Intron)YP_009380245.1 [petD]\| |
| cP0000183 | PotentialSSR | PentaSSR-Cp | 76618 | 76627 | GAATC | 2 | petD | (Intron)YP_009380245.1 [petD]\| |
| cP0000184 | PotentialSSR | PentaSSR-Cp | 76631 | 76640 | AAGAA | 2 | petD | (Intron)YP_009380245.1 [petD]\| |
| cP0000185 | PotentialSSR | PentaSSR-Cp | 77210 | 77219 | ATTCA | 2 |  |  |
| cM0000018 | SSR | MonoSSR-Cp | 77437 | 77446 | T | 10 | rpoA | YP_009380246.1 [rpoA]\| |
| cH0000080 | PotentialSSR | HexaSSR-Cp | 77656 | 77667 | CATTTC | 2 | rpoA | YP_009380246.1 [rpoA]\| |
| cP0000186 | PotentialSSR | PentaSSR-Cp | 78086 | 78095 | TCGCA | 2 | rpoA | YP_009380246.1 [rpoA]\| |
| c70000022 | ExtendedSSR | 7SSR-Cp | 78844 | 78857 | TTTTAGT | 2 |  |  |
| cP0000187 | PotentialSSR | PentaSSR-Cp | 78854 | 78863 | TAGTA | 2 |  |  |
| cM0000019 | SSR | MonoSSR-Cp | 78997 | 79007 | T | 11 |  |  |
| cH0000081 | PotentialSSR | HexaSSR-Cp | 79080 | 79091 | GTTGAA | 2 |  |  |
| cP0000188 | PotentialSSR | PentaSSR-Cp | 79912 | 79921 | TTAGT | 2 |  |  |
| cH0000082 | PotentialSSR | HexaSSR-Cp | 79983 | 79994 | TATTTT | 2 |  |  |
| cH0000083 | PotentialSSR | HexaSSR-Cp | 80990 | 81001 | AAAAAT | 2 | rpl16 | (Intron)YP_009380252.1 [rpl16]\| |
| cP0000189 | PotentialSSR | PentaSSR-Cp | 81017 | 81026 | TATTT | 2 | rpl16 | (Intron)YP_009380252.1 [rpl16]\| |
| cH0000084 | PotentialSSR | HexaSSR-Cp | 81019 | 81030 | TTTTAT | 2 | rpl16 | (Intron)YP_009380252.1 [rpl16]\| |
| c70000023 | ExtendedSSR | 7SSR-Cp | 81235 | 81248 | TTTTATA | 2 | rpl16 | (Intron)YP_009380252.1 [rpl16]\| |
| cP0000190 | PotentialSSR | PentaSSR-Cp | 81291 | 81300 | AAAAG | 2 | rpl16 | (Intron)YP_009380252.1 [rpl16]\| |
| cM0000020 | SSR | MonoSSR-Cp | 81577 | 81586 | T | 10 | rpl16 | (Intron)YP_009380252.1 [rpl16]\| |
| cM0000021 | SSR | MonoSSR-Cp | 81710 | 81720 | T | 11 | rpl16 | (Intron)YP_009380252.1 [rpl16]\| |
| cM0000022 | SSR | MonoSSR-Cp | 81769 | 81778 | T | 10 | rpl16 | (Intron)YP_009380252.1 [rpl16]\| |
| cH0000085 | PotentialSSR | HexaSSR-Cp | 81958 | 81969 | TTTTAA | 2 | rpl16 | (Intron)YP_009380252.1 [rpl16]\| |
| cP0000191 | PotentialSSR | PentaSSR-Cp | 82265 | 82274 | ACCCT | 2 | rps3 | YP_009380253.1 [rps3]\| |
| cP0000192 | PotentialSSR | PentaSSR-Cp | 82399 | 82408 | TACTC | 2 | rps3 | YP_009380253.1 [rps3]\| |
| cP0000193 | PotentialSSR | PentaSSR-Cp | 82502 | 82511 | CAATT | 2 | rps3 | YP_009380253.1 [rps3]\| |
| cP0000194 | PotentialSSR | PentaSSR-Cp | 82860 | 82869 | TTTAT | 2 | rpl22 | YP_009380254.1 [rpl22]\| |
| cP0000195 | PotentialSSR | PentaSSR-Cp | 83381 | 83390 | TTTCT | 2 |  |  |
| cH0000086 | PotentialSSR | HexaSSR-Cp | 83810 | 83821 | ATTTTC | 2 |  |  |
| cP0000196 | PotentialSSR | PentaSSR-Cp | 84225 | 84234 | TATGT | 2 | rpl2 | YP_009380256.1 [rpl2]\| |
| cP0000197 | PotentialSSR | PentaSSR-Cp | 84573 | 84582 | TGGAT | 2 | rpl2 | YP_009380256.1 [rpl2]\| |
| cP0000198 | PotentialSSR | PentaSSR-Cp | 84674 | 84683 | TTCTT | 2 |  |  |
| cP0000199 | PotentialSSR | PentaSSR-Cp | 84695 | 84704 | GAATA | 2 |  |  |
| cP0000200 | PotentialSSR | PentaSSR-Cp | 85053 | 85062 | TATGA | 2 |  |  |
| cP0000201 | PotentialSSR | PentaSSR-Cp | 85269 | 85278 | TGAAA | 2 |  |  |
| cH0000087 | PotentialSSR | HexaSSR-Cp | 85935 | 85946 | GAAAGA | 2 | ycf2 | YP_009380257.1 [ycf2]\| |
| cP0000202 | PotentialSSR | PentaSSR-Cp | 85987 | 85996 | GATCC | 2 | ycf2 | YP_009380257.1 [ycf2]\| |
| c90000003 | ExtendedSSR | 9SSR-Cp | 87267 | 87284 | GGAACATTT | 2 | ycf2 | YP_009380257.1 [ycf2]\| |
| cP0000203 | PotentialSSR | PentaSSR-Cp | 88114 | 88123 | CGATC | 2 | ycf2 | YP_009380257.1 [ycf2]\| |
| cH0000088 | PotentialSSR | HexaSSR-Cp | 88274 | 88285 | TCAATT | 2 | ycf2 | YP_009380257.1 [ycf2]\| |
| cP0000204 | PotentialSSR | PentaSSR-Cp | 88279 | 88288 | TTCAA | 2 | ycf2 | YP_009380257.1 [ycf2]\| |
| cH0000089 | PotentialSSR | HexaSSR-Cp | 89056 | 89067 | GGTCCC | 2 | ycf2 | YP_009380257.1 [ycf2]\| |
| cH0000090 | PotentialSSR | HexaSSR-Cp | 89232 | 89243 | AAGAAA | 2 | ycf2 | YP_009380257.1 [ycf2]\| |
| cP0000205 | PotentialSSR | PentaSSR-Cp | 89257 | 89266 | GATTG | 2 | ycf2 | YP_009380257.1 [ycf2]\| |
| cH0000091 | PotentialSSR | HexaSSR-Cp | 89898 | 89909 | GGAGCT | 2 | ycf2 | YP_009380257.1 [ycf2]\| |
| cP0000206 | PotentialSSR | PentaSSR-Cp | 90280 | 90289 | GAAAA | 2 | ycf2 | YP_009380257.1 [ycf2]\| |
| cH0000092 | PotentialSSR | HexaSSR-Cp | 90785 | 90796 | TAGAAG | 2 | ycf2 | YP_009380257.1 [ycf2]\| |
| cH0000093 | PotentialSSR | HexaSSR-Cp | 91385 | 91396 | CTATAT | 2 | ycf2 | YP_009380257.1 [ycf2]\| |
| cP0000207 | PotentialSSR | PentaSSR-Cp | 91879 | 91888 | AAGTT | 2 |  |  |
| cP0000208 | PotentialSSR | PentaSSR-Cp | 91907 | 91916 | TTGTT | 2 |  |  |
| cP0000209 | PotentialSSR | PentaSSR-Cp | 92151 | 92160 | GTTAC | 2 |  |  |
| cH0000094 | PotentialSSR | HexaSSR-Cp | 92326 | 92337 | ATTCCA | 2 |  |  |
| cP0000210 | PotentialSSR | PentaSSR-Cp | 92642 | 92651 | CTTAT | 2 |  |  |
| cP0000211 | PotentialSSR | PentaSSR-Cp | 92777 | 92786 | ATGGA | 2 |  |  |
| cH0000095 | PotentialSSR | HexaSSR-Cp | 93738 | 93749 | GCTGAA | 2 | ndhB | YP_009380258.1 [ndhB]\| |
| cH0000096 | PotentialSSR | HexaSSR-Cp | 93797 | 93808 | AGAGTC | 2 | ndhB | YP_009380258.1 [ndhB]\| |
| cP0000212 | PotentialSSR | PentaSSR-Cp | 94093 | 94102 | TGATT | 2 | ndhB | (Intron)YP_009380258.1 [ndhB]\| |
| cP0000213 | PotentialSSR | PentaSSR-Cp | 94293 | 94302 | AAAGA | 2 | ndhB | (Intron)YP_009380258.1 [ndhB]\| |
| cH0000097 | PotentialSSR | HexaSSR-Cp | 95360 | 95371 | TTCTTA | 2 |  |  |
| cP0000214 | PotentialSSR | PentaSSR-Cp | 95426 | 95435 | AGAAA | 2 |  |  |
| cP0000215 | PotentialSSR | PentaSSR-Cp | 95530 | 95539 | CTGTT | 2 |  |  |
| cH0000098 | PotentialSSR | HexaSSR-Cp | 96277 | 96288 | TCCATA | 2 |  |  |
| cP0000216 | PotentialSSR | PentaSSR-Cp | 96465 | 96474 | CGAAT | 2 |  |  |
| cH0000099 | PotentialSSR | HexaSSR-Cp | 97054 | 97065 | TTGATT | 2 |  |  |
| cH0000100 | PotentialSSR | HexaSSR-Cp | 97101 | 97112 | TTCCTC | 2 |  |  |
| cH0000101 | PotentialSSR | HexaSSR-Cp | 97113 | 97124 | TATCCC | 2 |  |  |
| cP0000217 | PotentialSSR | PentaSSR-Cp | 97359 | 97368 | TGTTG | 2 |  |  |
| cP0000218 | PotentialSSR | PentaSSR-Cp | 97457 | 97466 | TATTA | 2 |  |  |
| cP0000219 | PotentialSSR | PentaSSR-Cp | 97476 | 97485 | ATTAG | 2 |  |  |
| cP0000220 | PotentialSSR | PentaSSR-Cp | 97658 | 97667 | GCAAT | 2 |  |  |
| cH0000102 | PotentialSSR | HexaSSR-Cp | 97778 | 97789 | TATTAC | 2 |  |  |
| cH0000103 | PotentialSSR | HexaSSR-Cp | 97925 | 97936 | AATGGA | 2 |  |  |
| cP0000221 | PotentialSSR | PentaSSR-Cp | 98030 | 98039 | CTTTT | 2 |  |  |
| cM0000023 | SSR | MonoSSR-Cp | 98036 | 98047 | T | 12 |  |  |
| cP0000222 | PotentialSSR | PentaSSR-Cp | 98287 | 98296 | CAAGA | 2 |  |  |
| c80000009 | ExtendedSSR | 8SSR-Cp | 98294 | 98309 | AGAATAGT | 2 |  |  |
| cP0000223 | PotentialSSR | PentaSSR-Cp | 98370 | 98379 | AGGGA | 2 |  |  |
| cH0000104 | PotentialSSR | HexaSSR-Cp | 98494 | 98505 | GAATGA | 2 |  |  |
| cH0000105 | PotentialSSR | HexaSSR-Cp | 99367 | 99378 | GACACT | 2 | rrn16 | rrn16 [rrn16]\| |
| cH0000106 | PotentialSSR | HexaSSR-Cp | 100824 | 100835 | AATGGA | 2 | trnI-GAU | (Intron)trnI-GAU [trnI-GAU]\| |
| cH0000107 | PotentialSSR | HexaSSR-Cp | 101514 | 101525 | AAGAAT | 2 |  |  |
| cP0000224 | PotentialSSR | PentaSSR-Cp | 101745 | 101754 | ACAAA | 2 | trnA-UGC | (Intron)trnA-UGC [trnA-UGC]\| |
| cP0000225 | PotentialSSR | PentaSSR-Cp | 102064 | 102073 | TTCAA | 2 | trnA-UGC | (Intron)trnA-UGC [trnA-UGC]\| |
| c80000010 | ExtendedSSR | 8SSR-Cp | 102288 | 102303 | TTTTGAGA | 2 | trnA-UGC | (Intron)trnA-UGC [trnA-UGC]\| |
| cD0000008 | SSR | DiSSR-Cp | 103863 | 103872 | CG | 5 | rrn23 | rrn23 [rrn23]\| |
| cH0000108 | PotentialSSR | HexaSSR-Cp | 103886 | 103897 | GAAGCG | 2 | rrn23 | rrn23 [rrn23]\| |
| cTe0000007 | SSR | TetraSSR-Cp | 104280 | 104291 | AGGT | 3 | rrn23 | rrn23 [rrn23]\| |
| cP0000226 | PotentialSSR | PentaSSR-Cp | 105373 | 105382 | GCGGA | 2 | rrn23 | rrn23 [rrn23]\| |
| cP0000227 | PotentialSSR | PentaSSR-Cp | 105671 | 105680 | ATCCA | 2 |  |  |
| c70000024 | ExtendedSSR | 7SSR-Cp | 105743 | 105756 | AAAAACC | 2 |  |  |
| cH0000109 | PotentialSSR | HexaSSR-Cp | 105773 | 105784 | TCTATC | 2 |  |  |
| cH0000110 | PotentialSSR | HexaSSR-Cp | 106150 | 106161 | TTCTTA | 2 |  |  |
| cP0000228 | PotentialSSR | PentaSSR-Cp | 106447 | 106456 | AGTGG | 2 |  |  |
| cH0000111 | PotentialSSR | HexaSSR-Cp | 106587 | 106598 | CAAGTA | 2 |  |  |
| cP0000229 | PotentialSSR | PentaSSR-Cp | 106603 | 106612 | TAGCA | 2 |  |  |
| cP0000230 | PotentialSSR | PentaSSR-Cp | 106627 | 106636 | GTCAT | 2 |  |  |
| c70000025 | ExtendedSSR | 7SSR-Cp | 106640 | 106653 | TATGTTT | 2 |  |  |
| cP0000231 | PotentialSSR | PentaSSR-Cp | 106812 | 106821 | CAGAA | 2 |  |  |
| cH0000112 | PotentialSSR | HexaSSR-Cp | 106818 | 106829 | AGAATG | 2 |  |  |
| c70000026 | ExtendedSSR | 7SSR-Cp | 106918 | 106931 | AAGAATG | 2 |  |  |
| c90000004 | ExtendedSSR | 9SSR-Cp | 107479 | 107496 | GAAGAAGGA | 2 | ycf1 | YP_009380260.1 [ycf1]\| |
| cH0000113 | PotentialSSR | HexaSSR-Cp | 108101 | 108112 | TAGAAA | 2 | ycf1 | YP_009380260.1 [ycf1]\| |
| cH0000114 | PotentialSSR | HexaSSR-Cp | 108168 | 108179 | TCCTTC | 2 | ycf1 | YP_009380260.1 [ycf1]\| |
| cP0000232 | PotentialSSR | PentaSSR-Cp | 108191 | 108200 | AAGAA | 2 | ycf1 | YP_009380260.1 [ycf1]\| |
| cH0000115 | PotentialSSR | HexaSSR-Cp | 108210 | 108221 | CAAAAT | 2 | ycf1 | YP_009380260.1 [ycf1]\| |
| cP0000233 | PotentialSSR | PentaSSR-Cp | 108257 | 108266 | ACAAA | 2 | ycf1 | YP_009380260.1 [ycf1]\| |
| cP0000234 | PotentialSSR | PentaSSR-Cp | 108482 | 108491 | GAAAT | 2 | ycf1 | YP_009380260.1 [ycf1]\| |
| cM0000024 | SSR | MonoSSR-Cp | 108668 | 108677 | A | 10 | ycf1 | YP_009380260.1 [ycf1]\| |
| cP0000235 | PotentialSSR | PentaSSR-Cp | 108782 | 108791 | AAAAT | 2 | ycf1,ndhF | YP_009380260.1 [ycf1]\|YP_009380261.1 [ndhF]\| |
| cH0000116 | PotentialSSR | HexaSSR-Cp | 109940 | 109951 | AGATCC | 2 | ndhF | YP_009380261.1 [ndhF]\| |
| c70000027 | ExtendedSSR | 7SSR-Cp | 110407 | 110420 | CTCGAAA | 2 | ndhF | YP_009380261.1 [ndhF]\| |
| cP0000236 | PotentialSSR | PentaSSR-Cp | 111033 | 111042 | TAGAA | 2 |  |  |
| c70000028 | ExtendedSSR | 7SSR-Cp | 111206 | 111219 | TTAAAAC | 2 |  |  |
| cP0000237 | PotentialSSR | PentaSSR-Cp | 111462 | 111471 | TTACT | 2 |  |  |
| cM0000025 | SSR | MonoSSR-Cp | 112176 | 112185 | A | 10 |  |  |
| cP0000238 | PotentialSSR | PentaSSR-Cp | 112424 | 112433 | TTTTA | 2 |  |  |
| c70000029 | ExtendedSSR | 7SSR-Cp | 112444 | 112457 | TAAAAGA | 2 |  |  |
| cM0000026 | SSR | MonoSSR-Cp | 112495 | 112504 | A | 10 |  |  |
| cP0000239 | PotentialSSR | PentaSSR-Cp | 112791 | 112800 | AGAAA | 2 |  |  |
| cH0000117 | PotentialSSR | HexaSSR-Cp | 112944 | 112955 | TATGAA | 2 |  |  |
| cH0000118 | PotentialSSR | HexaSSR-Cp | 113204 | 113215 | AATGAA | 2 |  |  |
| cM0000027 | SSR | MonoSSR-Cp | 113263 | 113272 | T | 10 |  |  |
| cP0000240 | PotentialSSR | PentaSSR-Cp | 114390 | 114399 | TTTTG | 2 |  |  |
| cP0000241 | PotentialSSR | PentaSSR-Cp | 114428 | 114437 | TTTCA | 2 |  |  |
| cH0000119 | PotentialSSR | HexaSSR-Cp | 114526 | 114537 | AATACC | 2 | ndhD | YP_009380264.1 [ndhD]\| |
| cM0000028 | SSR | MonoSSR-Cp | 114592 | 114601 | A | 10 | ndhD | YP_009380264.1 [ndhD]\| |
| cH0000120 | PotentialSSR | HexaSSR-Cp | 115522 | 115533 | TAATTC | 2 | ndhD | YP_009380264.1 [ndhD]\| |
| cH0000121 | PotentialSSR | HexaSSR-Cp | 116374 | 116385 | TCTAGT | 2 |  |  |
| cP0000242 | PotentialSSR | PentaSSR-Cp | 117009 | 117018 | AAGTT | 2 |  |  |
| cP0000243 | PotentialSSR | PentaSSR-Cp | 117309 | 117318 | ATACC | 2 | ndhG | YP_009380267.1 [ndhG]\| |
| c70000030 | ExtendedSSR | 7SSR-Cp | 117521 | 117534 | TAGAATA | 2 | ndhG | YP_009380267.1 [ndhG]\| |
| cP0000244 | PotentialSSR | PentaSSR-Cp | 117796 | 117805 | TAAAG | 2 |  |  |
| cP0000245 | PotentialSSR | PentaSSR-Cp | 117961 | 117970 | TTTAA | 2 |  |  |
| cP0000246 | PotentialSSR | PentaSSR-Cp | 118082 | 118091 | TAATT | 2 | ndhI | YP_009380268.1 [ndhI]\| |
| cH0000122 | PotentialSSR | HexaSSR-Cp | 118724 | 118735 | GAACAA | 2 | ndhA | YP_009380269.1 [ndhA]\| |
| cP0000247 | PotentialSSR | PentaSSR-Cp | 118748 | 118757 | TAATG | 2 | ndhA | YP_009380269.1 [ndhA]\| |
| cP0000248 | PotentialSSR | PentaSSR-Cp | 118901 | 118910 | ATAAA | 2 | ndhA | YP_009380269.1 [ndhA]\| |
| cP0000249 | PotentialSSR | PentaSSR-Cp | 119129 | 119138 | AAGAT | 2 | ndhA | (Intron)YP_009380269.1 [ndhA]\| |
| cP0000250 | PotentialSSR | PentaSSR-Cp | 119317 | 119326 | CTATA | 2 | ndhA | (Intron)YP_009380269.1 [ndhA]\| |
| c70000031 | ExtendedSSR | 7SSR-Cp | 119439 | 119452 | TATCAAT | 2 | ndhA | (Intron)YP_009380269.1 [ndhA]\| |
| cM0000029 | SSR | MonoSSR-Cp | 119568 | 119577 | T | 10 | ndhA | (Intron)YP_009380269.1 [ndhA]\| |
| cH0000123 | PotentialSSR | HexaSSR-Cp | 119582 | 119593 | CTATTA | 2 | ndhA | (Intron)YP_009380269.1 [ndhA]\| |
| cP0000251 | PotentialSSR | PentaSSR-Cp | 121033 | 121042 | CATTC | 2 | ndhH | YP_009380270.1 [ndhH]\| |
| cH0000124 | PotentialSSR | HexaSSR-Cp | 122042 | 122053 | ATAATT | 2 |  |  |
| cP0000252 | PotentialSSR | PentaSSR-Cp | 122447 | 122456 | TTTAT | 2 |  |  |
| cH0000125 | PotentialSSR | HexaSSR-Cp | 122732 | 122743 | AATTTT | 2 | ycf1 | YP_009380272.1 [ycf1]\| |
| cP0000253 | PotentialSSR | PentaSSR-Cp | 123172 | 123181 | TTCTT | 2 | ycf1 | YP_009380272.1 [ycf1]\| |
| cM0000030 | SSR | MonoSSR-Cp | 123751 | 123762 | T | 12 | ycf1 | YP_009380272.1 [ycf1]\| |
| cH0000126 | PotentialSSR | HexaSSR-Cp | 124118 | 124129 | CTATAT | 2 | ycf1 | YP_009380272.1 [ycf1]\| |
| cH0000127 | PotentialSSR | HexaSSR-Cp | 124216 | 124227 | CAATAA | 2 | ycf1 | YP_009380272.1 [ycf1]\| |
| cTe0000008 | SSR | TetraSSR-Cp | 124250 | 124261 | TAAT | 3 | ycf1 | YP_009380272.1 [ycf1]\| |
| cM0000031 | SSR | MonoSSR-Cp | 125296 | 125306 | A | 11 | ycf1 | YP_009380272.1 [ycf1]\| |
| cP0000254 | PotentialSSR | PentaSSR-Cp | 125461 | 125470 | AAAAC | 2 | ycf1 | YP_009380272.1 [ycf1]\| |
| cP0000255 | PotentialSSR | PentaSSR-Cp | 126797 | 126806 | ATTTT | 2 | ycf1 | YP_009380272.1 [ycf1]\| |
| cM0000032 | SSR | MonoSSR-Cp | 126911 | 126920 | T | 10 | ycf1 | YP_009380272.1 [ycf1]\| |
| cP0000256 | PotentialSSR | PentaSSR-Cp | 127096 | 127105 | CATTT | 2 | ycf1 | YP_009380272.1 [ycf1]\| |
| cP0000257 | PotentialSSR | PentaSSR-Cp | 127322 | 127331 | TTTGT | 2 | ycf1 | YP_009380272.1 [ycf1]\| |
| cH0000128 | PotentialSSR | HexaSSR-Cp | 127367 | 127378 | ATTTTG | 2 | ycf1 | YP_009380272.1 [ycf1]\| |
| cP0000258 | PotentialSSR | PentaSSR-Cp | 127388 | 127397 | TTCTT | 2 | ycf1 | YP_009380272.1 [ycf1]\| |
| cH0000129 | PotentialSSR | HexaSSR-Cp | 127409 | 127420 | GAAGGA | 2 | ycf1 | YP_009380272.1 [ycf1]\| |
| cH0000130 | PotentialSSR | HexaSSR-Cp | 127476 | 127487 | TTTCTA | 2 | ycf1 | YP_009380272.1 [ycf1]\| |
| c90000005 | ExtendedSSR | 9SSR-Cp | 128092 | 128109 | TCCTTCTTC | 2 | ycf1 | YP_009380272.1 [ycf1]\| |
| c70000032 | ExtendedSSR | 7SSR-Cp | 128655 | 128668 | TTCATTC | 2 |  |  |
| cH0000131 | PotentialSSR | HexaSSR-Cp | 128759 | 128770 | CATTCT | 2 |  |  |
| cP0000259 | PotentialSSR | PentaSSR-Cp | 128767 | 128776 | TTCTG | 2 |  |  |
| c70000033 | ExtendedSSR | 7SSR-Cp | 128935 | 128948 | AAACATA | 2 |  |  |
| cP0000260 | PotentialSSR | PentaSSR-Cp | 128952 | 128961 | ATGAC | 2 |  |  |
| cP0000261 | PotentialSSR | PentaSSR-Cp | 128976 | 128985 | TGCTA | 2 |  |  |
| cH0000132 | PotentialSSR | HexaSSR-Cp | 128990 | 129001 | TACTTG | 2 |  |  |
| cP0000262 | PotentialSSR | PentaSSR-Cp | 129131 | 129140 | TCCAC | 2 |  |  |
| cH0000133 | PotentialSSR | HexaSSR-Cp | 129426 | 129437 | ATAAGA | 2 |  |  |
| cH0000134 | PotentialSSR | HexaSSR-Cp | 129804 | 129815 | GATAGA | 2 |  |  |
| c70000034 | ExtendedSSR | 7SSR-Cp | 129832 | 129845 | GGTTTTT | 2 |  |  |
| cP0000263 | PotentialSSR | PentaSSR-Cp | 129908 | 129917 | TGGAT | 2 |  |  |
| cP0000264 | PotentialSSR | PentaSSR-Cp | 130206 | 130215 | TCCGC | 2 | rrn23 | rrn23 [rrn23]\| |
| cTe0000009 | SSR | TetraSSR-Cp | 131295 | 131306 | CTAC | 3 | rrn23 | rrn23 [rrn23]\| |
| cH0000135 | PotentialSSR | HexaSSR-Cp | 131691 | 131702 | CGCTTC | 2 | rrn23 | rrn23 [rrn23]\| |
| cD0000009 | SSR | DiSSR-Cp | 131716 | 131725 | CG | 5 | rrn23 | rrn23 [rrn23]\| |
| c80000011 | ExtendedSSR | 8SSR-Cp | 133285 | 133300 | TCTCAAAA | 2 | trnA-UGC | (Intron)trnA-UGC [trnA-UGC]\| |
| cP0000265 | PotentialSSR | PentaSSR-Cp | 133514 | 133523 | ATTGA | 2 | trnA-UGC | (Intron)trnA-UGC [trnA-UGC]\| |
| cP0000266 | PotentialSSR | PentaSSR-Cp | 133834 | 133843 | TTTGT | 2 | trnA-UGC | (Intron)trnA-UGC [trnA-UGC]\| |
| cH0000136 | PotentialSSR | HexaSSR-Cp | 134063 | 134074 | ATTCTT | 2 |  |  |
| cH0000137 | PotentialSSR | HexaSSR-Cp | 134753 | 134764 | TCCATT | 2 | trnI-GAU | (Intron)trnI-GAU [trnI-GAU]\| |
| cH0000138 | PotentialSSR | HexaSSR-Cp | 136208 | 136219 | TCAGTG | 2 | rrn16 | rrn16 [rrn16]\| |
| cH0000139 | PotentialSSR | HexaSSR-Cp | 137083 | 137094 | TCATTC | 2 |  |  |
| cP0000267 | PotentialSSR | PentaSSR-Cp | 137209 | 137218 | TCCCT | 2 | trnV-GAC | trnV-GAC [trnV-GAC]\| |
| c80000012 | ExtendedSSR | 8SSR-Cp | 137279 | 137294 | ACTATTCT | 2 |  |  |
| cP0000268 | PotentialSSR | PentaSSR-Cp | 137292 | 137301 | TCTTG | 2 |  |  |
| cM0000033 | SSR | MonoSSR-Cp | 137541 | 137552 | A | 12 |  |  |
| cH0000140 | PotentialSSR | HexaSSR-Cp | 137650 | 137661 | TTTCCA | 2 |  |  |
| cH0000141 | PotentialSSR | HexaSSR-Cp | 137796 | 137807 | ATAGTA | 2 |  |  |
| cP0000269 | PotentialSSR | PentaSSR-Cp | 137920 | 137929 | CATTG | 2 |  |  |
| cP0000270 | PotentialSSR | PentaSSR-Cp | 138103 | 138112 | CTAAT | 2 |  |  |
| cP0000271 | PotentialSSR | PentaSSR-Cp | 138122 | 138131 | TAATA | 2 |  |  |
| cP0000272 | PotentialSSR | PentaSSR-Cp | 138220 | 138229 | CAACA | 2 |  |  |
| cH0000142 | PotentialSSR | HexaSSR-Cp | 138464 | 138475 | GGGATA | 2 |  |  |
| cH0000143 | PotentialSSR | HexaSSR-Cp | 138476 | 138487 | GAGGAA | 2 |  |  |
| cH0000144 | PotentialSSR | HexaSSR-Cp | 138521 | 138532 | AAAATC | 2 |  |  |
| cP0000273 | PotentialSSR | PentaSSR-Cp | 139113 | 139122 | GATTC | 2 |  |  |
| cH0000145 | PotentialSSR | HexaSSR-Cp | 139300 | 139311 | TATGGA | 2 |  |  |
| cP0000274 | PotentialSSR | PentaSSR-Cp | 140048 | 140057 | GAACA | 2 |  |  |
| cP0000275 | PotentialSSR | PentaSSR-Cp | 140153 | 140162 | TTTCT | 2 |  |  |
| cH0000146 | PotentialSSR | HexaSSR-Cp | 140217 | 140228 | TAAGAA | 2 |  |  |
| cP0000276 | PotentialSSR | PentaSSR-Cp | 141286 | 141295 | TCTTT | 2 | ndhB | (Intron)YP_009380274.1 [ndhB]\| |
| cP0000277 | PotentialSSR | PentaSSR-Cp | 141486 | 141495 | AATCA | 2 | ndhB | (Intron)YP_009380274.1 [ndhB]\| |
| cH0000147 | PotentialSSR | HexaSSR-Cp | 141780 | 141791 | GACTCT | 2 | ndhB | YP_009380274.1 [ndhB]\| |
| cH0000148 | PotentialSSR | HexaSSR-Cp | 141837 | 141848 | GCTTCA | 2 | ndhB | YP_009380274.1 [ndhB]\| |
| cP0000278 | PotentialSSR | PentaSSR-Cp | 142801 | 142810 | TTCCA | 2 |  |  |
| cP0000279 | PotentialSSR | PentaSSR-Cp | 142937 | 142946 | ATAAG | 2 |  |  |
| cH0000149 | PotentialSSR | HexaSSR-Cp | 143250 | 143261 | TTGGAA | 2 |  |  |
| cP0000280 | PotentialSSR | PentaSSR-Cp | 143427 | 143436 | CGTAA | 2 |  |  |
| cP0000281 | PotentialSSR | PentaSSR-Cp | 143672 | 143681 | AACAA | 2 |  |  |
| cP0000282 | PotentialSSR | PentaSSR-Cp | 143700 | 143709 | AACTT | 2 |  |  |
| cH0000150 | PotentialSSR | HexaSSR-Cp | 144192 | 144203 | ATATAG | 2 | ycf2 | YP_009380275.1 [ycf2]\| |
| cH0000151 | PotentialSSR | HexaSSR-Cp | 144792 | 144803 | CTTCTA | 2 | ycf2 | YP_009380275.1 [ycf2]\| |
| cP0000283 | PotentialSSR | PentaSSR-Cp | 145299 | 145308 | TTTTC | 2 | ycf2 | YP_009380275.1 [ycf2]\| |
| cH0000152 | PotentialSSR | HexaSSR-Cp | 145678 | 145689 | CAGCTC | 2 | ycf2 | YP_009380275.1 [ycf2]\| |
| cP0000284 | PotentialSSR | PentaSSR-Cp | 146322 | 146331 | CAATC | 2 | ycf2 | YP_009380275.1 [ycf2]\| |
| cH0000153 | PotentialSSR | HexaSSR-Cp | 146342 | 146353 | CTTTTT | 2 | ycf2 | YP_009380275.1 [ycf2]\| |
| cH0000154 | PotentialSSR | HexaSSR-Cp | 146521 | 146532 | GGGACC | 2 | ycf2 | YP_009380275.1 [ycf2]\| |
| cP0000285 | PotentialSSR | PentaSSR-Cp | 147300 | 147309 | TTGAA | 2 | ycf2 | YP_009380275.1 [ycf2]\| |
| cH0000155 | PotentialSSR | HexaSSR-Cp | 147303 | 147314 | AATTGA | 2 | ycf2 | YP_009380275.1 [ycf2]\| |
| cP0000286 | PotentialSSR | PentaSSR-Cp | 147465 | 147474 | GATCG | 2 | ycf2 | YP_009380275.1 [ycf2]\| |
| c90000006 | ExtendedSSR | 9SSR-Cp | 148304 | 148321 | AAATGTTCC | 2 | ycf2 | YP_009380275.1 [ycf2]\| |
| cP0000287 | PotentialSSR | PentaSSR-Cp | 149591 | 149600 | CGGAT | 2 | ycf2 | YP_009380275.1 [ycf2]\| |
| cH0000156 | PotentialSSR | HexaSSR-Cp | 149642 | 149653 | TCTTTC | 2 | ycf2 | YP_009380275.1 [ycf2]\| |
| cP0000288 | PotentialSSR | PentaSSR-Cp | 150309 | 150318 | ATTTC | 2 |  |  |
| cP0000289 | PotentialSSR | PentaSSR-Cp | 150526 | 150535 | TCATA | 2 |  |  |
| cP0000290 | PotentialSSR | PentaSSR-Cp | 150884 | 150893 | TATTC | 2 |  |  |
| cP0000291 | PotentialSSR | PentaSSR-Cp | 150904 | 150913 | AAAGA | 2 |  |  |
| cP0000292 | PotentialSSR | PentaSSR-Cp | 151005 | 151014 | AATCC | 2 | rpl2 | YP_009380276.1 [rpl2]\| |
| cP0000293 | PotentialSSR | PentaSSR-Cp | 151354 | 151363 | ACATA | 2 | rpl2 | YP_009380276.1 [rpl2]\| |
| cH0000157 | PotentialSSR | HexaSSR-Cp | 151767 | 151778 | GAAAAT | 2 |  |  |

**Supplementary Table 5. List of SSRs identified in CSJUK01 of *C. album***

| **Name** | **SSR type** | **Type** | **Cooridnation** | | **Unit sequence** | **Repeat number** | **Genes** | **Annotation** |
| --- | --- | --- | --- | --- | --- | --- | --- | --- |
| cH0000001 | PotentialSSR | HexaSSR-Cp | 113 | 124 | GTAAAG | 2 |  |  |
| cH0000002 | PotentialSSR | HexaSSR-Cp | 1240 | 1251 | GCTTTC | 2 | psbA | YP_009380194.1 [psbA]\| |
| cP0000001 | PotentialSSR | PentaSSR-Cp | 1292 | 1301 | ATTTA | 2 |  |  |
| c70000001 | ExtendedSSR | 7SSR-Cp | 1454 | 1467 | AGAAAAT | 2 |  |  |
| c70000002 | ExtendedSSR | 7SSR-Cp | 1696 | 1709 | AGTAGAA | 2 | trnK-UUU | (Intron)trnK-UUU [trnK-UUU]\| |
| cH0000003 | PotentialSSR | HexaSSR-Cp | 1748 | 1759 | AATTTC | 2 | trnK-UUU | (Intron)trnK-UUU [trnK-UUU]\| |
| cH0000004 | PotentialSSR | HexaSSR-Cp | 2949 | 2960 | TTTTTC | 2 | matK,trnK-UUU | YP_009380195.1 [matK]\|(Intron)trnK-UUU [trnK-UUU]\| |
| cP0000002 | PotentialSSR | PentaSSR-Cp | 4093 | 4102 | TATGT | 2 |  |  |
| cP0000003 | PotentialSSR | PentaSSR-Cp | 4124 | 4133 | AATGG | 2 |  |  |
| cP0000004 | PotentialSSR | PentaSSR-Cp | 4202 | 4211 | CAGAT | 2 |  |  |
| cP0000005 | PotentialSSR | PentaSSR-Cp | 4355 | 4364 | CATTT | 2 |  |  |
| cH0000005 | PotentialSSR | HexaSSR-Cp | 4442 | 4453 | AATATT | 2 |  |  |
| cTe0000001 | SSR | TetraSSR-Cp | 4464 | 4475 | TTTA | 3 |  |  |
| cP0000006 | PotentialSSR | PentaSSR-Cp | 4487 | 4496 | ATTTA | 2 |  |  |
| cP0000007 | PotentialSSR | PentaSSR-Cp | 4572 | 4581 | ATATA | 2 |  |  |
| cT0000001 | SSR | TriSSR-Cp | 4613 | 4624 | ATA | 4 |  |  |
| cP0000008 | PotentialSSR | PentaSSR-Cp | 4970 | 4979 | TAGAT | 2 | rps16 | (Intron)YP_009380196.1 [rps16]\| |
| cP0000009 | PotentialSSR | PentaSSR-Cp | 5204 | 5213 | CATTT | 2 | rps16 | (Intron)YP_009380196.1 [rps16]\| |
| cH0000007 | PotentialSSR | HexaSSR-Cp | 5274 | 5285 | ATCCAA | 2 | rps16 | (Intron)YP_009380196.1 [rps16]\| |
| c70000004 | ExtendedSSR | 7SSR-Cp | 5294 | 5307 | ACAATTA | 2 | rps16 | (Intron)YP_009380196.1 [rps16]\| |
| cM0000001 | SSR | MonoSSR-Cp | 5341 | 5351 | T | 11 | rps16 | (Intron)YP_009380196.1 [rps16]\| |
| cP0000010 | PotentialSSR | PentaSSR-Cp | 5441 | 5450 | AGAAT | 2 | rps16 | (Intron)YP_009380196.1 [rps16]\| |
| cP0000011 | PotentialSSR | PentaSSR-Cp | 5997 | 6006 | AATGC | 2 |  |  |
| cP0000012 | SSR | PentaSSR-Cp | 6080 | 6094 | TTAAT | 3 |  |  |
| cP0000013 | PotentialSSR | PentaSSR-Cp | 6190 | 6199 | TATAT | 2 |  |  |
| cH0000009 | PotentialSSR | HexaSSR-Cp | 6467 | 6478 | GCTCTG | 2 |  |  |
| cP0000014 | PotentialSSR | PentaSSR-Cp | 6555 | 6564 | TTCTA | 2 |  |  |
| cP0000015 | PotentialSSR | PentaSSR-Cp | 6629 | 6638 | GATTC | 2 |  |  |
| cM0000002 | SSR | MonoSSR-Cp | 6787 | 6797 | A | 11 |  |  |
| cM0000003 | SSR | MonoSSR-Cp | 6853 | 6862 | A | 10 |  |  |
| c80000001 | ExtendedSSR | 8SSR-Cp | 7100 | 7115 | ATTCTAAT | 2 |  |  |
| cH0000010 | PotentialSSR | HexaSSR-Cp | 7345 | 7356 | TTACAA | 2 |  |  |
| cP0000016 | PotentialSSR | PentaSSR-Cp | 7398 | 7407 | TCAAA | 2 |  |  |
| cP0000017 | PotentialSSR | PentaSSR-Cp | 7608 | 7617 | TTCTT | 2 |  |  |
| cP0000018 | PotentialSSR | PentaSSR-Cp | 7640 | 7649 | ATTCT | 2 |  |  |
| cM0000004 | SSR | MonoSSR-Cp | 7729 | 7739 | A | 11 |  |  |
| cP0000019 | PotentialSSR | PentaSSR-Cp | 7895 | 7904 | GAAAA | 2 |  |  |
| cP0000020 | PotentialSSR | PentaSSR-Cp | 7985 | 7994 | ATATA | 2 |  |  |
| cH0000012 | PotentialSSR | HexaSSR-Cp | 8065 | 8076 | AGATAA | 2 |  |  |
| cP0000021 | PotentialSSR | PentaSSR-Cp | 8133 | 8142 | TAAAA | 2 |  |  |
| cP0000022 | PotentialSSR | PentaSSR-Cp | 8209 | 8218 | AAAGA | 2 |  |  |
| cP0000023 | PotentialSSR | PentaSSR-Cp | 8264 | 8273 | GGCCT | 2 |  |  |
| cH0000013 | PotentialSSR | HexaSSR-Cp | 8342 | 8353 | TTTGAT | 2 |  |  |
| cP0000024 | PotentialSSR | PentaSSR-Cp | 8858 | 8867 | AATTG | 2 | trnG-UCC | (Intron)trnG-UCC [trnG-UCC]\| |
| cP0000025 | PotentialSSR | PentaSSR-Cp | 9223 | 9232 | TTTTG | 2 | trnG-UCC | (Intron)trnG-UCC [trnG-UCC]\| |
| cP0000026 | PotentialSSR | PentaSSR-Cp | 9248 | 9257 | CTTAA | 2 | trnG-UCC | (Intron)trnG-UCC [trnG-UCC]\| |
| cP0000027 | PotentialSSR | PentaSSR-Cp | 9281 | 9290 | TTTTC | 2 | trnG-UCC | (Intron)trnG-UCC [trnG-UCC]\| |
| cP0000028 | PotentialSSR | PentaSSR-Cp | 9479 | 9488 | TCTCA | 2 |  |  |
| cP0000029 | PotentialSSR | PentaSSR-Cp | 9501 | 9510 | CAAAA | 2 |  |  |
| cM0000005 | SSR | MonoSSR-Cp | 9527 | 9536 | A | 10 |  |  |
| cH0000016 | PotentialSSR | HexaSSR-Cp | 10442 | 10453 | GCTTGT | 2 | atpA | YP_009380199.1 [atpA]\| |
| cH0000017 | PotentialSSR | HexaSSR-Cp | 10845 | 10856 | CGGGAG | 2 | atpA | YP_009380199.1 [atpA]\| |
| c80000002 | ExtendedSSR | 8SSR-Cp | 11966 | 11981 | AAAAATAG | 2 | atpF | (Intron)YP_009380200.1 [atpF]\| |
| c70000008 | ExtendedSSR | 7SSR-Cp | 12208 | 12221 | TCGGTAT | 2 | atpF | (Intron)YP_009380200.1 [atpF]\| |
| cT0000002 | SSR | TriSSR-Cp | 12314 | 12325 | ATT | 4 | atpF | (Intron)YP_009380200.1 [atpF]\| |
| cTe0000002 | SSR | TetraSSR-Cp | 12879 | 12890 | GGAA | 3 |  |  |
| cP0000030 | PotentialSSR | PentaSSR-Cp | 13027 | 13036 | AGAAA | 2 |  |  |
| cP0000031 | PotentialSSR | PentaSSR-Cp | 13052 | 13061 | TTTCT | 2 |  |  |
| cD0000001 | SSR | DiSSR-Cp | 13323 | 13332 | CA | 5 |  |  |
| cP0000032 | PotentialSSR | PentaSSR-Cp | 13814 | 13823 | AAATA | 2 |  |  |
| cD0000002 | SSR | DiSSR-Cp | 13881 | 13890 | AT | 5 |  |  |
| cH0000018 | PotentialSSR | HexaSSR-Cp | 14254 | 14265 | TAAAGC | 2 | atpI | YP_009380202.1 [atpI]\| |
| cM0000006 | SSR | MonoSSR-Cp | 14706 | 14715 | A | 10 |  |  |
| cH0000019 | PotentialSSR | HexaSSR-Cp | 14754 | 14765 | TTTAAT | 2 |  |  |
| cP0000033 | PotentialSSR | PentaSSR-Cp | 15624 | 15633 | ATTAA | 2 |  |  |
| c80000003 | ExtendedSSR | 8SSR-Cp | 15683 | 15698 | TCTACCGC | 2 |  |  |
| cP0000034 | PotentialSSR | PentaSSR-Cp | 15762 | 15771 | TTTAT | 2 |  |  |
| cP0000035 | PotentialSSR | PentaSSR-Cp | 15775 | 15784 | TAAAT | 2 |  |  |
| cP0000036 | PotentialSSR | PentaSSR-Cp | 16839 | 16848 | CAATT | 2 | rpoC2 | YP_009380204.1 [rpoC2]\| |
| cP0000037 | PotentialSSR | PentaSSR-Cp | 17654 | 17663 | CAAAA | 2 | rpoC2 | YP_009380204.1 [rpoC2]\| |
| cP0000038 | PotentialSSR | PentaSSR-Cp | 17774 | 17783 | TATCT | 2 | rpoC2 | YP_009380204.1 [rpoC2]\| |
| cM0000007 | SSR | MonoSSR-Cp | 17830 | 17842 | T | 13 | rpoC2 | YP_009380204.1 [rpoC2]\| |
| cP0000039 | PotentialSSR | PentaSSR-Cp | 18313 | 18322 | CGATT | 2 | rpoC2 | YP_009380204.1 [rpoC2]\| |
| cP0000040 | PotentialSSR | PentaSSR-Cp | 18342 | 18351 | ATCCT | 2 | rpoC2 | YP_009380204.1 [rpoC2]\| |
| cH0000020 | PotentialSSR | HexaSSR-Cp | 18455 | 18466 | TTGATC | 2 | rpoC2 | YP_009380204.1 [rpoC2]\| |
| cH0000021 | PotentialSSR | HexaSSR-Cp | 18827 | 18838 | ACGTGT | 2 | rpoC2 | YP_009380204.1 [rpoC2]\| |
| cP0000041 | PotentialSSR | PentaSSR-Cp | 19039 | 19048 | CATAA | 2 | rpoC2 | YP_009380204.1 [rpoC2]\| |
| cD0000003 | SSR | DiSSR-Cp | 19182 | 19191 | AT | 5 | rpoC2 | YP_009380204.1 [rpoC2]\| |
| cH0000022 | PotentialSSR | HexaSSR-Cp | 21521 | 21532 | CAAATC | 2 | rpoC1 | YP_009380205.1 [rpoC1]\| |
| cP0000042 | PotentialSSR | PentaSSR-Cp | 21635 | 21644 | GGATT | 2 | rpoC1 | YP_009380205.1 [rpoC1]\| |
| cP0000043 | PotentialSSR | PentaSSR-Cp | 21724 | 21733 | ATCCT | 2 | rpoC1 | (Intron)YP_009380205.1 [rpoC1]\| |
| cP0000044 | PotentialSSR | PentaSSR-Cp | 21918 | 21927 | TTCTT | 2 | rpoC1 | (Intron)YP_009380205.1 [rpoC1]\| |
| cP0000045 | PotentialSSR | PentaSSR-Cp | 21950 | 21959 | AATTA | 2 | rpoC1 | (Intron)YP_009380205.1 [rpoC1]\| |
| cP0000046 | PotentialSSR | PentaSSR-Cp | 22134 | 22143 | TTAAA | 2 | rpoC1 | (Intron)YP_009380205.1 [rpoC1]\| |
| cH0000023 | PotentialSSR | HexaSSR-Cp | 22239 | 22250 | ACAAAA | 2 | rpoC1 | (Intron)YP_009380205.1 [rpoC1]\| |
| cM0000008 | SSR | MonoSSR-Cp | 25507 | 25516 | T | 10 | rpoB | YP_009380206.1 [rpoB]\| |
| cH0000024 | PotentialSSR | HexaSSR-Cp | 25950 | 25961 | TCTTTT | 2 | rpoB | YP_009380206.1 [rpoB]\| |
| cP0000047 | PotentialSSR | PentaSSR-Cp | 26429 | 26438 | ACTTA | 2 |  |  |
| cP0000048 | PotentialSSR | PentaSSR-Cp | 26722 | 26731 | TTGTA | 2 |  |  |
| cH0000026 | PotentialSSR | HexaSSR-Cp | 26883 | 26894 | AGAAAA | 2 |  |  |
| cP0000049 | PotentialSSR | PentaSSR-Cp | 27099 | 27108 | GAATC | 2 |  |  |
| cP0000050 | PotentialSSR | PentaSSR-Cp | 27559 | 27568 | TTCCC | 2 |  |  |
| cP0000051 | PotentialSSR | PentaSSR-Cp | 27688 | 27697 | CTGAG | 2 |  |  |
| cH0000027 | PotentialSSR | HexaSSR-Cp | 27800 | 27811 | TTCATA | 2 |  |  |
| cP0000052 | PotentialSSR | PentaSSR-Cp | 27932 | 27941 | TTTAG | 2 |  |  |
| cH0000028 | PotentialSSR | HexaSSR-Cp | 27958 | 27969 | TATCAT | 2 |  |  |
| cP0000053 | PotentialSSR | PentaSSR-Cp | 28191 | 28200 | ATTTC | 2 |  |  |
| cP0000054 | PotentialSSR | PentaSSR-Cp | 28259 | 28268 | TCAAT | 2 |  |  |
| cP0000055 | PotentialSSR | PentaSSR-Cp | 28285 | 28294 | TTGGC | 2 |  |  |
| cP0000056 | PotentialSSR | PentaSSR-Cp | 28343 | 28352 | ACTTT | 2 |  |  |
| c70000009 | ExtendedSSR | 7SSR-Cp | 28410 | 28423 | TTATAGT | 2 |  |  |
| cP0000057 | PotentialSSR | PentaSSR-Cp | 28447 | 28456 | CTAAT | 2 |  |  |
| cP0000058 | PotentialSSR | PentaSSR-Cp | 28823 | 28832 | TATCA | 2 |  |  |
| cH0000029 | PotentialSSR | HexaSSR-Cp | 29048 | 29059 | CAAAAA | 2 |  |  |
| cM0000009 | SSR | MonoSSR-Cp | 29374 | 29383 | A | 10 |  |  |
| cP0000059 | PotentialSSR | PentaSSR-Cp | 29752 | 29761 | ATAAA | 2 |  |  |
| c80000004 | ExtendedSSR | 8SSR-Cp | 29771 | 29786 | TTTTCTTT | 2 |  |  |
| cP0000060 | PotentialSSR | PentaSSR-Cp | 29828 | 29837 | ACCAT | 2 |  |  |
| cH0000030 | PotentialSSR | HexaSSR-Cp | 29868 | 29879 | AATTTC | 2 |  |  |
| cTe0000003 | SSR | TetraSSR-Cp | 29887 | 29898 | AAAT | 3 |  |  |
| cP0000061 | PotentialSSR | PentaSSR-Cp | 30106 | 30115 | TCAAT | 2 |  |  |
| cP0000062 | PotentialSSR | PentaSSR-Cp | 30157 | 30166 | TACCC | 2 | trnE-UUC | trnE-UUC [trnE-UUC]\| |
| cH0000031 | PotentialSSR | HexaSSR-Cp | 30361 | 30372 | ATTTCA | 2 |  |  |
| cH0000032 | PotentialSSR | HexaSSR-Cp | 30499 | 30510 | CTAATA | 2 |  |  |
| cP0000063 | PotentialSSR | PentaSSR-Cp | 30643 | 30652 | GTATA | 2 |  |  |
| cP0000064 | PotentialSSR | PentaSSR-Cp | 30955 | 30964 | ATTAT | 2 |  |  |
| cP0000065 | PotentialSSR | PentaSSR-Cp | 31028 | 31037 | TGGAA | 2 |  |  |
| c70000010 | ExtendedSSR | 7SSR-Cp | 31489 | 31502 | GTTTATT | 2 |  |  |
| cH0000033 | PotentialSSR | HexaSSR-Cp | 31566 | 31577 | GTGAAA | 2 |  |  |
| cP0000066 | PotentialSSR | PentaSSR-Cp | 31742 | 31751 | AAAGA | 2 |  |  |
| cP0000067 | PotentialSSR | PentaSSR-Cp | 31949 | 31958 | TAAAT | 2 |  |  |
| cP0000068 | PotentialSSR | PentaSSR-Cp | 32112 | 32121 | CGTTT | 2 | psbD | YP_009380209.1 [psbD]\| |
| cP0000069 | PotentialSSR | PentaSSR-Cp | 32742 | 32751 | AACCC | 2 | psbD | YP_009380209.1 [psbD]\| |
| cP0000070 | PotentialSSR | PentaSSR-Cp | 33835 | 33844 | GTCTG | 2 | psbC | YP_009380210.1 [psbC]\| |
| cH0000034 | PotentialSSR | HexaSSR-Cp | 33971 | 33982 | CTCAAG | 2 | psbC | YP_009380210.1 [psbC]\| |
| cP0000071 | PotentialSSR | PentaSSR-Cp | 34263 | 34272 | GGGTG | 2 | psbC | YP_009380210.1 [psbC]\| |
| cH0000035 | PotentialSSR | HexaSSR-Cp | 34389 | 34400 | TGCAGC | 2 | psbC | YP_009380210.1 [psbC]\| |
| c70000011 | ExtendedSSR | 7SSR-Cp | 34512 | 34525 | TACATAT | 2 |  |  |
| cP0000072 | PotentialSSR | PentaSSR-Cp | 34590 | 34599 | TAATT | 2 |  |  |
| cH0000036 | PotentialSSR | HexaSSR-Cp | 34821 | 34832 | TTATTC | 2 |  |  |
| cP0000073 | PotentialSSR | PentaSSR-Cp | 34923 | 34932 | TATAT | 2 |  |  |
| c70000012 | ExtendedSSR | 7SSR-Cp | 34963 | 34976 | TTAATTA | 2 |  |  |
| cP0000074 | PotentialSSR | PentaSSR-Cp | 35299 | 35308 | TGGAT | 2 | psbZ | YP_009380211.1 [psbZ]\| |
| cP0000075 | PotentialSSR | PentaSSR-Cp | 35467 | 35476 | AAACA | 2 |  |  |
| cP0000076 | PotentialSSR | PentaSSR-Cp | 35630 | 35639 | TATAT | 2 |  |  |
| cH0000037 | PotentialSSR | HexaSSR-Cp | 35796 | 35807 | GTTATA | 2 |  |  |
| cP0000077 | PotentialSSR | PentaSSR-Cp | 35819 | 35828 | TAGTG | 2 |  |  |
| cP0000078 | PotentialSSR | PentaSSR-Cp | 35922 | 35931 | TATTT | 2 |  |  |
| cP0000079 | PotentialSSR | PentaSSR-Cp | 36833 | 36842 | CCACG | 2 | psaB | YP_009380213.1 [psaB]\| |
| cP0000080 | PotentialSSR | PentaSSR-Cp | 36982 | 36991 | CCATC | 2 | psaB | YP_009380213.1 [psaB]\| |
| cP0000081 | PotentialSSR | PentaSSR-Cp | 38261 | 38270 | TGTCC | 2 | psaB | YP_009380213.1 [psaB]\| |
| cP0000082 | PotentialSSR | PentaSSR-Cp | 38767 | 38776 | ACCAA | 2 | psaB | YP_009380213.1 [psaB]\| |
| cH0000038 | PotentialSSR | HexaSSR-Cp | 39193 | 39204 | TAATAG | 2 | psaA | YP_009380214.1 [psaA]\| |
| cP0000083 | PotentialSSR | PentaSSR-Cp | 39942 | 39951 | ATGTG | 2 | psaA | YP_009380214.1 [psaA]\| |
| cP0000084 | PotentialSSR | PentaSSR-Cp | 41579 | 41588 | TATTT | 2 |  |  |
| cH0000039 | PotentialSSR | HexaSSR-Cp | 41757 | 41768 | TCTTTA | 2 |  |  |
| cP0000085 | PotentialSSR | PentaSSR-Cp | 41851 | 41860 | TTTTA | 2 |  |  |
| cM0000010 | SSR | MonoSSR-Cp | 41890 | 41899 | A | 10 |  |  |
| cP0000086 | PotentialSSR | PentaSSR-Cp | 42432 | 42441 | AAAGA | 2 | ycf3 | (Intron)YP_009380215.1 [ycf3]\| |
| cP0000087 | PotentialSSR | PentaSSR-Cp | 42569 | 42578 | TTCTT | 2 | ycf3 | (Intron)YP_009380215.1 [ycf3]\| |
| cM0000011 | SSR | MonoSSR-Cp | 42642 | 42651 | T | 10 | ycf3 | (Intron)YP_009380215.1 [ycf3]\| |
| cP0000088 | PotentialSSR | PentaSSR-Cp | 43143 | 43152 | AATAT | 2 | ycf3 | (Intron)YP_009380215.1 [ycf3]\| |
| cTe0000004 | SSR | TetraSSR-Cp | 43713 | 43724 | GTTT | 3 | ycf3 | (Intron)YP_009380215.1 [ycf3]\| |
| cP0000089 | PotentialSSR | PentaSSR-Cp | 43822 | 43831 | AAATC | 2 | ycf3 | (Intron)YP_009380215.1 [ycf3]\| |
| cP0000090 | PotentialSSR | PentaSSR-Cp | 44031 | 44040 | GTACA | 2 |  |  |
| cP0000091 | PotentialSSR | PentaSSR-Cp | 44120 | 44129 | TATTT | 2 |  |  |
| cP0000092 | PotentialSSR | PentaSSR-Cp | 44207 | 44216 | CAAAT | 2 |  |  |
| cP0000093 | PotentialSSR | PentaSSR-Cp | 44221 | 44230 | TAGTA | 2 |  |  |
| cP0000094 | PotentialSSR | PentaSSR-Cp | 44274 | 44283 | GATCA | 2 |  |  |
| cP0000095 | PotentialSSR | PentaSSR-Cp | 44343 | 44352 | AATAG | 2 |  |  |
| cP0000096 | PotentialSSR | PentaSSR-Cp | 44520 | 44529 | GATTC | 2 |  |  |
| cTe0000005 | SSR | TetraSSR-Cp | 44535 | 44546 | AATT | 3 |  |  |
| cP0000097 | PotentialSSR | PentaSSR-Cp | 44555 | 44564 | TATAT | 2 |  |  |
| cD0000004 | SSR | DiSSR-Cp | 44627 | 44636 | AT | 5 |  |  |
| cP0000098 | PotentialSSR | PentaSSR-Cp | 44655 | 44664 | TTTTA | 2 |  |  |
| cP0000099 | PotentialSSR | PentaSSR-Cp | 44706 | 44715 | ATTAC | 2 |  |  |
| cP0000100 | PotentialSSR | PentaSSR-Cp | 44754 | 44763 | ATAGT | 2 |  |  |
| cP0000101 | PotentialSSR | PentaSSR-Cp | 44839 | 44848 | ATAGT | 2 |  |  |
| cP0000102 | PotentialSSR | PentaSSR-Cp | 45027 | 45036 | CAAAT | 2 |  |  |
| cP0000103 | PotentialSSR | PentaSSR-Cp | 45203 | 45212 | GGGAT | 2 |  |  |
| cH0000040 | PotentialSSR | HexaSSR-Cp | 45270 | 45281 | TTTTCC | 2 |  |  |
| cP0000104 | PotentialSSR | PentaSSR-Cp | 45696 | 45705 | ACCTG | 2 | rps4 | YP_009380216.1 [rps4]\| |
| cD0000005 | SSR | DiSSR-Cp | 45979 | 45992 | TA | 7 |  |  |
| cP0000105 | PotentialSSR | PentaSSR-Cp | 46037 | 46046 | AAATG | 2 |  |  |
| cP0000106 | PotentialSSR | PentaSSR-Cp | 46128 | 46137 | TATTT | 2 |  |  |
| cH0000041 | PotentialSSR | HexaSSR-Cp | 46157 | 46168 | AAAAAG | 2 |  |  |
| cP0000107 | PotentialSSR | PentaSSR-Cp | 46212 | 46221 | CTTTT | 2 |  |  |
| cP0000108 | PotentialSSR | PentaSSR-Cp | 46504 | 46513 | TCAAA | 2 |  |  |
| cP0000109 | PotentialSSR | PentaSSR-Cp | 46582 | 46591 | CGATT | 2 |  |  |
| cP0000110 | PotentialSSR | PentaSSR-Cp | 46818 | 46827 | AATAA | 2 |  |  |
| cP0000111 | PotentialSSR | PentaSSR-Cp | 47407 | 47416 | CTGTG | 2 | trnL-UAA | (Intron)trnL-UAA [trnL-UAA]\| |
| cP0000112 | PotentialSSR | PentaSSR-Cp | 47500 | 47509 | TCTAT | 2 | trnL-UAA | (Intron)trnL-UAA [trnL-UAA]\| |
| cP0000113 | PotentialSSR | PentaSSR-Cp | 47891 | 47900 | TTTTC | 2 |  |  |
| cD0000006 | SSR | DiSSR-Cp | 47948 | 47957 | AT | 5 |  |  |
| cP0000114 | PotentialSSR | PentaSSR-Cp | 48262 | 48271 | CACAT | 2 |  |  |
| cH0000043 | PotentialSSR | HexaSSR-Cp | 48391 | 48402 | GAAAAT | 2 |  |  |
| cP0000115 | PotentialSSR | PentaSSR-Cp | 48522 | 48531 | CTTAA | 2 |  |  |
| cP0000116 | PotentialSSR | PentaSSR-Cp | 48541 | 48550 | TCTTT | 2 |  |  |
| cH0000044 | PotentialSSR | HexaSSR-Cp | 49010 | 49021 | CATATA | 2 | ndhJ | YP_009380217.1 [ndhJ]\| |
| c80000006 | ExtendedSSR | 8SSR-Cp | 49167 | 49182 | ATTTGTTT | 2 |  |  |
| cP0000117 | PotentialSSR | PentaSSR-Cp | 49190 | 49199 | TATTT | 2 |  |  |
| cP0000118 | PotentialSSR | PentaSSR-Cp | 49203 | 49212 | TTTAC | 2 |  |  |
| cH0000045 | PotentialSSR | HexaSSR-Cp | 49970 | 49981 | TTTTTC | 2 |  |  |
| cP0000119 | PotentialSSR | PentaSSR-Cp | 50035 | 50044 | TAAAC | 2 | ndhC | YP_009380219.1 [ndhC]\| |
| cP0000120 | PotentialSSR | PentaSSR-Cp | 50597 | 50606 | TTCTT | 2 |  |  |
| cP0000121 | PotentialSSR | PentaSSR-Cp | 50673 | 50682 | AATTC | 2 |  |  |
| cM0000012 | SSR | MonoSSR-Cp | 50686 | 50695 | T | 10 |  |  |
| cP0000122 | PotentialSSR | PentaSSR-Cp | 50730 | 50739 | TTCTA | 2 |  |  |
| cP0000123 | PotentialSSR | PentaSSR-Cp | 50789 | 50798 | ACTTG | 2 |  |  |
| c70000015 | ExtendedSSR | 7SSR-Cp | 52546 | 52559 | AATTTTT | 2 |  |  |
| cP0000124 | PotentialSSR | PentaSSR-Cp | 52684 | 52693 | ATTTG | 2 |  |  |
| cP0000125 | PotentialSSR | PentaSSR-Cp | 52785 | 52794 | TTGAA | 2 |  |  |
| cH0000047 | PotentialSSR | HexaSSR-Cp | 52838 | 52849 | TGTATA | 2 |  |  |
| cP0000126 | PotentialSSR | PentaSSR-Cp | 53065 | 53074 | ATTGA | 2 |  |  |
| cP0000127 | PotentialSSR | PentaSSR-Cp | 53104 | 53113 | AATTG | 2 |  |  |
| cM0000013 | SSR | MonoSSR-Cp | 53195 | 53206 | T | 12 |  |  |
| cH0000048 | PotentialSSR | HexaSSR-Cp | 53217 | 53228 | ATTTTC | 2 |  |  |
| cM0000014 | SSR | MonoSSR-Cp | 53302 | 53311 | A | 10 | atpB | YP_009380221.1 [atpB]\| |
| cP0000128 | PotentialSSR | PentaSSR-Cp | 54478 | 54487 | TTGGA | 2 | atpB | YP_009380221.1 [atpB]\| |
| cH0000049 | PotentialSSR | HexaSSR-Cp | 55175 | 55186 | ATAATC | 2 |  |  |
| cH0000050 | PotentialSSR | HexaSSR-Cp | 55245 | 55256 | TCTTTT | 2 |  |  |
| cH0000051 | PotentialSSR | HexaSSR-Cp | 55283 | 55294 | AAAAAG | 2 |  |  |
| cP0000129 | PotentialSSR | PentaSSR-Cp | 55757 | 55766 | AAACA | 2 | trnV-UAC | (Intron)trnV-UAC [trnV-UAC]\| |
| c70000016 | ExtendedSSR | 7SSR-Cp | 56089 | 56102 | AATGAAT | 2 | trnV-UAC | (Intron)trnV-UAC [trnV-UAC]\| |
| cH0000052 | PotentialSSR | HexaSSR-Cp | 56335 | 56346 | CAATTG | 2 |  |  |
| cP0000130 | PotentialSSR | PentaSSR-Cp | 56396 | 56405 | ATTTG | 2 |  |  |
| cH0000053 | PotentialSSR | HexaSSR-Cp | 56420 | 56431 | TTTGAA | 2 |  |  |
| cP0000131 | PotentialSSR | PentaSSR-Cp | 56519 | 56528 | TTTTC | 2 |  |  |
| cP0000132 | PotentialSSR | PentaSSR-Cp | 58585 | 58594 | TATAT | 2 |  |  |
| cP0000133 | PotentialSSR | PentaSSR-Cp | 58641 | 58650 | CTATA | 2 |  |  |
| cTe0000006 | SSR | TetraSSR-Cp | 58668 | 58679 | TAAT | 3 |  |  |
| cP0000134 | PotentialSSR | PentaSSR-Cp | 58682 | 58691 | TAATA | 2 |  |  |
| cH0000054 | PotentialSSR | HexaSSR-Cp | 58723 | 58734 | TACAAT | 2 |  |  |
| cP0000135 | PotentialSSR | PentaSSR-Cp | 58853 | 58862 | AAAAT | 2 | psaI | YP_009380224.1 [psaI]\| |
| cP0000136 | PotentialSSR | PentaSSR-Cp | 58871 | 58880 | TCGTC | 2 |  |  |
| cP0000137 | PotentialSSR | PentaSSR-Cp | 59011 | 59020 | ACAAT | 2 |  |  |
| cP0000138 | PotentialSSR | PentaSSR-Cp | 59175 | 59184 | TTCAA | 2 |  |  |
| cP0000139 | PotentialSSR | PentaSSR-Cp | 59485 | 59494 | TCGAT | 2 | ycf4 | YP_009380225.1 [ycf4]\| |
| cP0000140 | PotentialSSR | PentaSSR-Cp | 59590 | 59599 | TAGAA | 2 | ycf4 | YP_009380225.1 [ycf4]\| |
| cH0000055 | PotentialSSR | HexaSSR-Cp | 59923 | 59934 | TACAAA | 2 |  |  |
| cP0000141 | PotentialSSR | PentaSSR-Cp | 59936 | 59945 | CAATT | 2 |  |  |
| cP0000142 | PotentialSSR | PentaSSR-Cp | 60076 | 60085 | AATTA | 2 |  |  |
| cP0000143 | PotentialSSR | PentaSSR-Cp | 61041 | 61050 | AAAAG | 2 |  |  |
| c70000017 | ExtendedSSR | 7SSR-Cp | 61105 | 61118 | ATAATCA | 2 |  |  |
| cP0000144 | PotentialSSR | PentaSSR-Cp | 61190 | 61199 | TTATT | 2 |  |  |
| cP0000145 | PotentialSSR | PentaSSR-Cp | 61688 | 61697 | AAAGA | 2 | petA | YP_009380227.1 [petA]\| |
| cP0000146 | PotentialSSR | PentaSSR-Cp | 61959 | 61968 | GAAAA | 2 | petA | YP_009380227.1 [petA]\| |
| cH0000056 | PotentialSSR | HexaSSR-Cp | 62279 | 62290 | ATCAAG | 2 |  |  |
| cH0000057 | PotentialSSR | HexaSSR-Cp | 62295 | 62306 | TAACAA | 2 |  |  |
| cP0000147 | PotentialSSR | PentaSSR-Cp | 62319 | 62328 | CAATT | 2 |  |  |
| cM0000015 | SSR | MonoSSR-Cp | 62353 | 62366 | A | 14 |  |  |
| c80000007 | ExtendedSSR | 8SSR-Cp | 62460 | 62475 | ACTTTTTT | 2 |  |  |
| cH0000058 | PotentialSSR | HexaSSR-Cp | 62667 | 62678 | ATTTTT | 2 |  |  |
| cH0000059 | PotentialSSR | HexaSSR-Cp | 62862 | 62873 | TCTTTC | 2 |  |  |
| cP0000148 | PotentialSSR | PentaSSR-Cp | 62876 | 62885 | TTAAC | 2 |  |  |
| c70000018 | ExtendedSSR | 7SSR-Cp | 63128 | 63141 | TTACTAC | 2 | psbJ | YP_009380228.1 [psbJ]\| |
| cP0000149 | PotentialSSR | PentaSSR-Cp | 63481 | 63490 | ATTCA | 2 | psbL | YP_009380229.1 [psbL]\| |
| cH0000060 | PotentialSSR | HexaSSR-Cp | 63532 | 63543 | ATTCGG | 2 |  |  |
| c80000008 | ExtendedSSR | 8SSR-Cp | 64012 | 64027 | ACGTAAAA | 2 |  |  |
| cH0000061 | PotentialSSR | HexaSSR-Cp | 64349 | 64360 | TATAGA | 2 |  |  |
| cH0000062 | PotentialSSR | HexaSSR-Cp | 64588 | 64599 | AGTCTT | 2 |  |  |
| cP0000150 | PotentialSSR | PentaSSR-Cp | 65144 | 65153 | CTGTA | 2 |  |  |
| cM0000016 | SSR | MonoSSR-Cp | 65259 | 65271 | T | 13 |  |  |
| cP0000151 | PotentialSSR | PentaSSR-Cp | 65463 | 65472 | GATTA | 2 |  |  |
| cP0000152 | PotentialSSR | PentaSSR-Cp | 65480 | 65489 | TTTAG | 2 |  |  |
| cH0000063 | PotentialSSR | HexaSSR-Cp | 65499 | 65510 | TTTCTA | 2 |  |  |
| cP0000153 | PotentialSSR | PentaSSR-Cp | 65597 | 65606 | GAACT | 2 | trnW-CCA | trnW-CCA [trnW-CCA]\| |
| cH0000064 | PotentialSSR | HexaSSR-Cp | 65673 | 65684 | CTATAT | 2 |  |  |
| cP0000154 | PotentialSSR | PentaSSR-Cp | 65880 | 65889 | TTCAA | 2 |  |  |
| cP0000155 | PotentialSSR | PentaSSR-Cp | 65919 | 65928 | CCTTG | 2 |  |  |
| cP0000156 | PotentialSSR | PentaSSR-Cp | 66055 | 66064 | GTAAA | 2 |  |  |
| cP0000157 | PotentialSSR | PentaSSR-Cp | 66167 | 66176 | CTTAG | 2 |  |  |
| cP0000158 | PotentialSSR | PentaSSR-Cp | 66523 | 66532 | TTAGT | 2 |  |  |
| cP0000159 | PotentialSSR | PentaSSR-Cp | 66576 | 66585 | GTTAA | 2 |  |  |
| c70000019 | ExtendedSSR | 7SSR-Cp | 66717 | 66730 | CGAATTG | 2 |  |  |
| cH0000065 | PotentialSSR | HexaSSR-Cp | 67080 | 67091 | AATTTT | 2 |  |  |
| cD0000007 | SSR | DiSSR-Cp | 67105 | 67114 | AT | 5 |  |  |
| cP0000160 | PotentialSSR | PentaSSR-Cp | 67174 | 67183 | ATATA | 2 |  |  |
| cP0000161 | PotentialSSR | PentaSSR-Cp | 67249 | 67258 | ATAGG | 2 |  |  |
| cP0000162 | PotentialSSR | PentaSSR-Cp | 67276 | 67285 | TAAAC | 2 |  |  |
| cTe0000007 | SSR | TetraSSR-Cp | 67760 | 67771 | TTTA | 3 |  |  |
| cP0000163 | PotentialSSR | PentaSSR-Cp | 68323 | 68332 | ATAAA | 2 |  |  |
| cH0000066 | PotentialSSR | HexaSSR-Cp | 68814 | 68825 | ATTTTC | 2 |  |  |
| cP0000164 | PotentialSSR | PentaSSR-Cp | 68932 | 68941 | CTATT | 2 |  |  |
| cH0000067 | PotentialSSR | HexaSSR-Cp | 68979 | 68990 | TTTTGT | 2 |  |  |
| cH0000068 | PotentialSSR | HexaSSR-Cp | 69299 | 69310 | TGGGCT | 2 | clpP | YP_009380239.1 [clpP]\| |
| cP0000165 | PotentialSSR | PentaSSR-Cp | 69529 | 69538 | ACACA | 2 | clpP | (Intron)YP_009380239.1 [clpP]\| |
| cP0000166 | PotentialSSR | PentaSSR-Cp | 69674 | 69683 | ATCGA | 2 | clpP | (Intron)YP_009380239.1 [clpP]\| |
| cH0000069 | PotentialSSR | HexaSSR-Cp | 69685 | 69696 | CAGATC | 2 | clpP | (Intron)YP_009380239.1 [clpP]\| |
| cP0000167 | PotentialSSR | PentaSSR-Cp | 69970 | 69979 | GAAAA | 2 | clpP | (Intron)YP_009380239.1 [clpP]\| |
| cH0000070 | PotentialSSR | HexaSSR-Cp | 70260 | 70271 | ACAAAT | 2 | clpP | YP_009380239.1 [clpP]\| |
| cP0000168 | PotentialSSR | PentaSSR-Cp | 70673 | 70682 | TATCA | 2 | clpP | (Intron)YP_009380239.1 [clpP]\| |
| cH0000071 | PotentialSSR | HexaSSR-Cp | 70713 | 70724 | TTCTTG | 2 | clpP | (Intron)YP_009380239.1 [clpP]\| |
| cH0000072 | PotentialSSR | HexaSSR-Cp | 70923 | 70934 | TTGAAA | 2 | clpP | (Intron)YP_009380239.1 [clpP]\| |
| cH0000073 | PotentialSSR | HexaSSR-Cp | 70990 | 71001 | ATTGGG | 2 | clpP | (Intron)YP_009380239.1 [clpP]\| |
| cP0000169 | PotentialSSR | PentaSSR-Cp | 71559 | 71568 | ATAGA | 2 |  |  |
| cH0000074 | PotentialSSR | HexaSSR-Cp | 71678 | 71689 | CATAGT | 2 |  |  |
| c70000021 | ExtendedSSR | 7SSR-Cp | 71828 | 71841 | CTGGTTG | 2 | psbB | YP_009380240.1 [psbB]\| |
| cH0000075 | PotentialSSR | HexaSSR-Cp | 72192 | 72203 | GTTTTG | 2 | psbB | YP_009380240.1 [psbB]\| |
| cP0000170 | PotentialSSR | PentaSSR-Cp | 73298 | 73307 | TTTGA | 2 |  |  |
| cP0000171 | PotentialSSR | PentaSSR-Cp | 73455 | 73464 | CTCTA | 2 | psbT | YP_009380241.1 [psbT]\| |
| cP0000172 | PotentialSSR | PentaSSR-Cp | 73519 | 73528 | AAATG | 2 | psbT | YP_009380241.1 [psbT]\| |
| cH0000076 | PotentialSSR | HexaSSR-Cp | 74050 | 74061 | ACAAAA | 2 |  |  |
| c90000002 | ExtendedSSR | 9SSR-Cp | 74099 | 74116 | CAATACAAA | 2 |  |  |
| cP0000173 | PotentialSSR | PentaSSR-Cp | 74402 | 74411 | TTTTC | 2 | petB | (Intron)YP_009380244.1 [petB]\| |
| cP0000174 | PotentialSSR | PentaSSR-Cp | 74520 | 74529 | AATAA | 2 | petB | (Intron)YP_009380244.1 [petB]\| |
| cP0000175 | PotentialSSR | PentaSSR-Cp | 74532 | 74541 | CCTAT | 2 | petB | (Intron)YP_009380244.1 [petB]\| |
| cH0000077 | PotentialSSR | HexaSSR-Cp | 74708 | 74719 | ATTATA | 2 | petB | (Intron)YP_009380244.1 [petB]\| |
| cH0000078 | PotentialSSR | HexaSSR-Cp | 74720 | 74731 | TACAAA | 2 | petB | (Intron)YP_009380244.1 [petB]\| |
| cP0000176 | PotentialSSR | PentaSSR-Cp | 75662 | 75671 | ATAGA | 2 | petB | YP_009380244.1 [petB]\| |
| cP0000177 | PotentialSSR | PentaSSR-Cp | 76031 | 76040 | TCTAT | 2 | petD | (Intron)YP_009380245.1 [petD]\| |
| cP0000178 | PotentialSSR | PentaSSR-Cp | 76107 | 76116 | ATAAT | 2 | petD | (Intron)YP_009380245.1 [petD]\| |
| cP0000179 | PotentialSSR | PentaSSR-Cp | 76121 | 76130 | ATTAT | 2 | petD | (Intron)YP_009380245.1 [petD]\| |
| cP0000180 | PotentialSSR | PentaSSR-Cp | 76144 | 76153 | TTATT | 2 | petD | (Intron)YP_009380245.1 [petD]\| |
| cP0000181 | PotentialSSR | PentaSSR-Cp | 76616 | 76625 | GAATC | 2 | petD | (Intron)YP_009380245.1 [petD]\| |
| cP0000182 | PotentialSSR | PentaSSR-Cp | 76629 | 76638 | AAGAA | 2 | petD | (Intron)YP_009380245.1 [petD]\| |
| cP0000183 | PotentialSSR | PentaSSR-Cp | 77208 | 77217 | ATTCA | 2 |  |  |
| cM0000017 | SSR | MonoSSR-Cp | 77435 | 77444 | T | 10 | rpoA | YP_009380246.1 [rpoA]\| |
| cH0000079 | PotentialSSR | HexaSSR-Cp | 77654 | 77665 | CATTTC | 2 | rpoA | YP_009380246.1 [rpoA]\| |
| cP0000184 | PotentialSSR | PentaSSR-Cp | 78084 | 78093 | TCGCA | 2 | rpoA | YP_009380246.1 [rpoA]\| |
| cP0000185 | PotentialSSR | PentaSSR-Cp | 78852 | 78861 | TAGTA | 2 |  |  |
| cM0000018 | SSR | MonoSSR-Cp | 78995 | 79005 | T | 11 |  |  |
| cH0000080 | PotentialSSR | HexaSSR-Cp | 79078 | 79089 | GTTGAA | 2 |  |  |
| cP0000186 | PotentialSSR | PentaSSR-Cp | 79910 | 79919 | TTAGT | 2 |  |  |
| cH0000081 | PotentialSSR | HexaSSR-Cp | 79981 | 79992 | TATTTT | 2 |  |  |
| cH0000082 | PotentialSSR | HexaSSR-Cp | 80988 | 80999 | AAAAAT | 2 | rpl16 | (Intron)YP_009380252.1 [rpl16]\| |
| cP0000187 | PotentialSSR | PentaSSR-Cp | 81015 | 81024 | TATTT | 2 | rpl16 | (Intron)YP_009380252.1 [rpl16]\| |
| c70000023 | ExtendedSSR | 7SSR-Cp | 81233 | 81246 | TTTTATA | 2 | rpl16 | (Intron)YP_009380252.1 [rpl16]\| |
| cP0000188 | PotentialSSR | PentaSSR-Cp | 81289 | 81298 | AAAAG | 2 | rpl16 | (Intron)YP_009380252.1 [rpl16]\| |
| cM0000019 | SSR | MonoSSR-Cp | 81575 | 81584 | T | 10 | rpl16 | (Intron)YP_009380252.1 [rpl16]\| |
| cM0000020 | SSR | MonoSSR-Cp | 81708 | 81718 | T | 11 | rpl16 | (Intron)YP_009380252.1 [rpl16]\| |
| cM0000021 | SSR | MonoSSR-Cp | 81767 | 81776 | T | 10 | rpl16 | (Intron)YP_009380252.1 [rpl16]\| |
| cH0000084 | PotentialSSR | HexaSSR-Cp | 81956 | 81967 | TTTTAA | 2 | rpl16 | (Intron)YP_009380252.1 [rpl16]\| |
| cP0000189 | PotentialSSR | PentaSSR-Cp | 82263 | 82272 | ACCCT | 2 | rps3 | YP_009380253.1 [rps3]\| |
| cP0000190 | PotentialSSR | PentaSSR-Cp | 82397 | 82406 | TACTC | 2 | rps3 | YP_009380253.1 [rps3]\| |
| cP0000191 | PotentialSSR | PentaSSR-Cp | 82500 | 82509 | CAATT | 2 | rps3 | YP_009380253.1 [rps3]\| |
| cP0000192 | PotentialSSR | PentaSSR-Cp | 82858 | 82867 | TTTAT | 2 | rpl22 | YP_009380254.1 [rpl22]\| |
| cP0000193 | PotentialSSR | PentaSSR-Cp | 83379 | 83388 | TTTCT | 2 |  |  |
| cH0000085 | PotentialSSR | HexaSSR-Cp | 83808 | 83819 | ATTTTC | 2 |  |  |
| cP0000194 | PotentialSSR | PentaSSR-Cp | 84223 | 84232 | TATGT | 2 | rpl2 | YP_009380256.1 [rpl2]\| |
| cP0000195 | PotentialSSR | PentaSSR-Cp | 84571 | 84580 | TGGAT | 2 | rpl2 | YP_009380256.1 [rpl2]\| |
| cP0000196 | PotentialSSR | PentaSSR-Cp | 84672 | 84681 | TTCTT | 2 |  |  |
| cP0000197 | PotentialSSR | PentaSSR-Cp | 84693 | 84702 | GAATA | 2 |  |  |
| cP0000198 | PotentialSSR | PentaSSR-Cp | 85051 | 85060 | TATGA | 2 |  |  |
| cP0000199 | PotentialSSR | PentaSSR-Cp | 85267 | 85276 | TGAAA | 2 |  |  |
| cH0000086 | PotentialSSR | HexaSSR-Cp | 85933 | 85944 | GAAAGA | 2 | ycf2 | YP_009380257.1 [ycf2]\| |
| cP0000200 | PotentialSSR | PentaSSR-Cp | 85985 | 85994 | GATCC | 2 | ycf2 | YP_009380257.1 [ycf2]\| |
| c90000003 | ExtendedSSR | 9SSR-Cp | 87265 | 87282 | GGAACATTT | 2 | ycf2 | YP_009380257.1 [ycf2]\| |
| cP0000201 | PotentialSSR | PentaSSR-Cp | 88112 | 88121 | CGATC | 2 | ycf2 | YP_009380257.1 [ycf2]\| |
| cP0000202 | PotentialSSR | PentaSSR-Cp | 88277 | 88286 | TTCAA | 2 | ycf2 | YP_009380257.1 [ycf2]\| |
| cH0000088 | PotentialSSR | HexaSSR-Cp | 89054 | 89065 | GGTCCC | 2 | ycf2 | YP_009380257.1 [ycf2]\| |
| cH0000089 | PotentialSSR | HexaSSR-Cp | 89230 | 89241 | AAGAAA | 2 | ycf2 | YP_009380257.1 [ycf2]\| |
| cP0000203 | PotentialSSR | PentaSSR-Cp | 89255 | 89264 | GATTG | 2 | ycf2 | YP_009380257.1 [ycf2]\| |
| cH0000090 | PotentialSSR | HexaSSR-Cp | 89896 | 89907 | GGAGCT | 2 | ycf2 | YP_009380257.1 [ycf2]\| |
| cP0000204 | PotentialSSR | PentaSSR-Cp | 90278 | 90287 | GAAAA | 2 | ycf2 | YP_009380257.1 [ycf2]\| |
| cH0000091 | PotentialSSR | HexaSSR-Cp | 90783 | 90794 | TAGAAG | 2 | ycf2 | YP_009380257.1 [ycf2]\| |
| cH0000092 | PotentialSSR | HexaSSR-Cp | 91383 | 91394 | CTATAT | 2 | ycf2 | YP_009380257.1 [ycf2]\| |
| cP0000205 | PotentialSSR | PentaSSR-Cp | 91877 | 91886 | AAGTT | 2 |  |  |
| cP0000206 | PotentialSSR | PentaSSR-Cp | 91905 | 91914 | TTGTT | 2 |  |  |
| cP0000207 | PotentialSSR | PentaSSR-Cp | 92149 | 92158 | GTTAC | 2 |  |  |
| cH0000093 | PotentialSSR | HexaSSR-Cp | 92324 | 92335 | ATTCCA | 2 |  |  |
| cP0000208 | PotentialSSR | PentaSSR-Cp | 92640 | 92649 | CTTAT | 2 |  |  |
| cP0000209 | PotentialSSR | PentaSSR-Cp | 92775 | 92784 | ATGGA | 2 |  |  |
| cH0000094 | PotentialSSR | HexaSSR-Cp | 93736 | 93747 | GCTGAA | 2 | ndhB | YP_009380258.1 [ndhB]\| |
| cH0000095 | PotentialSSR | HexaSSR-Cp | 93795 | 93806 | AGAGTC | 2 | ndhB | YP_009380258.1 [ndhB]\| |
| cP0000210 | PotentialSSR | PentaSSR-Cp | 94091 | 94100 | TGATT | 2 | ndhB | (Intron)YP_009380258.1 [ndhB]\| |
| cP0000211 | PotentialSSR | PentaSSR-Cp | 94291 | 94300 | AAAGA | 2 | ndhB | (Intron)YP_009380258.1 [ndhB]\| |
| cH0000096 | PotentialSSR | HexaSSR-Cp | 95358 | 95369 | TTCTTA | 2 |  |  |
| cP0000212 | PotentialSSR | PentaSSR-Cp | 95424 | 95433 | AGAAA | 2 |  |  |
| cP0000213 | PotentialSSR | PentaSSR-Cp | 95528 | 95537 | CTGTT | 2 |  |  |
| cH0000097 | PotentialSSR | HexaSSR-Cp | 96275 | 96286 | TCCATA | 2 |  |  |
| cP0000214 | PotentialSSR | PentaSSR-Cp | 96463 | 96472 | CGAAT | 2 |  |  |
| cH0000098 | PotentialSSR | HexaSSR-Cp | 97052 | 97063 | TTGATT | 2 |  |  |
| cH0000099 | PotentialSSR | HexaSSR-Cp | 97099 | 97110 | TTCCTC | 2 |  |  |
| cH0000100 | PotentialSSR | HexaSSR-Cp | 97111 | 97122 | TATCCC | 2 |  |  |
| cP0000215 | PotentialSSR | PentaSSR-Cp | 97357 | 97366 | TGTTG | 2 |  |  |
| cP0000216 | PotentialSSR | PentaSSR-Cp | 97455 | 97464 | TATTA | 2 |  |  |
| cP0000217 | PotentialSSR | PentaSSR-Cp | 97474 | 97483 | ATTAG | 2 |  |  |
| cP0000218 | PotentialSSR | PentaSSR-Cp | 97656 | 97665 | GCAAT | 2 |  |  |
| cH0000101 | PotentialSSR | HexaSSR-Cp | 97776 | 97787 | TATTAC | 2 |  |  |
| cH0000102 | PotentialSSR | HexaSSR-Cp | 97923 | 97934 | AATGGA | 2 |  |  |
| cM0000022 | SSR | MonoSSR-Cp | 98033 | 98044 | T | 12 |  |  |
| cP0000219 | PotentialSSR | PentaSSR-Cp | 98284 | 98293 | CAAGA | 2 |  |  |
| cP0000220 | PotentialSSR | PentaSSR-Cp | 98367 | 98376 | AGGGA | 2 |  |  |
| cH0000103 | PotentialSSR | HexaSSR-Cp | 98491 | 98502 | GAATGA | 2 |  |  |
| cH0000104 | PotentialSSR | HexaSSR-Cp | 99364 | 99375 | GACACT | 2 | rrn16 | rrn16 [rrn16]\| |
| cH0000105 | PotentialSSR | HexaSSR-Cp | 100821 | 100832 | AATGGA | 2 | trnI-GAU | (Intron)trnI-GAU [trnI-GAU]\| |
| cH0000106 | PotentialSSR | HexaSSR-Cp | 101577 | 101588 | AAGAAT | 2 |  |  |
| cP0000221 | PotentialSSR | PentaSSR-Cp | 101808 | 101817 | ACAAA | 2 | trnA-UGC | (Intron)trnA-UGC [trnA-UGC]\| |
| cP0000222 | PotentialSSR | PentaSSR-Cp | 102127 | 102136 | TTCAA | 2 | trnA-UGC | (Intron)trnA-UGC [trnA-UGC]\| |
| c80000010 | ExtendedSSR | 8SSR-Cp | 102351 | 102366 | TTTTGAGA | 2 | trnA-UGC | (Intron)trnA-UGC [trnA-UGC]\| |
| cD0000008 | SSR | DiSSR-Cp | 103926 | 103935 | CG | 5 | rrn23 | rrn23 [rrn23]\| |
| cH0000107 | PotentialSSR | HexaSSR-Cp | 103949 | 103960 | GAAGCG | 2 | rrn23 | rrn23 [rrn23]\| |
| cTe0000008 | SSR | TetraSSR-Cp | 104343 | 104354 | AGGT | 3 | rrn23 | rrn23 [rrn23]\| |
| cP0000223 | PotentialSSR | PentaSSR-Cp | 105436 | 105445 | GCGGA | 2 | rrn23 | rrn23 [rrn23]\| |
| cP0000224 | PotentialSSR | PentaSSR-Cp | 105734 | 105743 | ATCCA | 2 |  |  |
| c70000024 | ExtendedSSR | 7SSR-Cp | 105806 | 105819 | AAAAACC | 2 |  |  |
| cH0000108 | PotentialSSR | HexaSSR-Cp | 105836 | 105847 | TCTATC | 2 |  |  |
| cH0000109 | PotentialSSR | HexaSSR-Cp | 106213 | 106224 | TTCTTA | 2 |  |  |
| cP0000225 | PotentialSSR | PentaSSR-Cp | 106510 | 106519 | AGTGG | 2 |  |  |
| cH0000110 | PotentialSSR | HexaSSR-Cp | 106650 | 106661 | CAAGTA | 2 |  |  |
| cP0000226 | PotentialSSR | PentaSSR-Cp | 106666 | 106675 | TAGCA | 2 |  |  |
| cP0000227 | PotentialSSR | PentaSSR-Cp | 106690 | 106699 | GTCAT | 2 |  |  |
| c70000025 | ExtendedSSR | 7SSR-Cp | 106703 | 106716 | TATGTTT | 2 |  |  |
| cP0000228 | PotentialSSR | PentaSSR-Cp | 106875 | 106884 | CAGAA | 2 |  |  |
| c70000026 | ExtendedSSR | 7SSR-Cp | 106981 | 106994 | AAGAATG | 2 |  |  |
| c90000004 | ExtendedSSR | 9SSR-Cp | 107542 | 107559 | GAAGAAGGA | 2 | ycf1 | YP_009380260.1 [ycf1]\| |
| cH0000112 | PotentialSSR | HexaSSR-Cp | 108164 | 108175 | TAGAAA | 2 | ycf1 | YP_009380260.1 [ycf1]\| |
| cH0000113 | PotentialSSR | HexaSSR-Cp | 108231 | 108242 | TCCTTC | 2 | ycf1 | YP_009380260.1 [ycf1]\| |
| cP0000229 | PotentialSSR | PentaSSR-Cp | 108254 | 108263 | AAGAA | 2 | ycf1 | YP_009380260.1 [ycf1]\| |
| cH0000114 | PotentialSSR | HexaSSR-Cp | 108273 | 108284 | CAAAAT | 2 | ycf1 | YP_009380260.1 [ycf1]\| |
| cP0000230 | PotentialSSR | PentaSSR-Cp | 108320 | 108329 | ACAAA | 2 | ycf1 | YP_009380260.1 [ycf1]\| |
| cP0000231 | PotentialSSR | PentaSSR-Cp | 108545 | 108554 | GAAAT | 2 | ycf1 | YP_009380260.1 [ycf1]\| |
| cM0000023 | SSR | MonoSSR-Cp | 108731 | 108740 | A | 10 | ycf1 | YP_009380260.1 [ycf1]\| |
| cP0000232 | PotentialSSR | PentaSSR-Cp | 108845 | 108854 | AAAAT | 2 | ycf1,ndhF | YP_009380260.1 [ycf1]\|YP_009380261.1 [ndhF]\| |
| cH0000115 | PotentialSSR | HexaSSR-Cp | 110003 | 110014 | AGATCC | 2 | ndhF | YP_009380261.1 [ndhF]\| |
| c70000027 | ExtendedSSR | 7SSR-Cp | 110470 | 110483 | CTCGAAA | 2 | ndhF | YP_009380261.1 [ndhF]\| |
| cP0000233 | PotentialSSR | PentaSSR-Cp | 111096 | 111105 | TAGAA | 2 |  |  |
| c70000028 | ExtendedSSR | 7SSR-Cp | 111269 | 111282 | TTAAAAC | 2 |  |  |
| cP0000234 | PotentialSSR | PentaSSR-Cp | 111525 | 111534 | TTACT | 2 |  |  |
| cM0000024 | SSR | MonoSSR-Cp | 112239 | 112248 | A | 10 |  |  |
| cP0000235 | PotentialSSR | PentaSSR-Cp | 112487 | 112496 | TTTTA | 2 |  |  |
| c70000029 | ExtendedSSR | 7SSR-Cp | 112507 | 112520 | TAAAAGA | 2 |  |  |
| cM0000025 | SSR | MonoSSR-Cp | 112558 | 112567 | A | 10 |  |  |
| cP0000236 | PotentialSSR | PentaSSR-Cp | 112854 | 112863 | AGAAA | 2 |  |  |
| cP0000237 | PotentialSSR | PentaSSR-Cp | 112983 | 112992 | GAAAA | 2 |  |  |
| cH0000116 | PotentialSSR | HexaSSR-Cp | 113169 | 113180 | TATGAA | 2 |  |  |
| cH0000117 | PotentialSSR | HexaSSR-Cp | 113429 | 113440 | AATGAA | 2 |  |  |
| cM0000026 | SSR | MonoSSR-Cp | 113488 | 113498 | T | 11 |  |  |
| cP0000238 | PotentialSSR | PentaSSR-Cp | 114616 | 114625 | TTTTG | 2 |  |  |
| cP0000239 | PotentialSSR | PentaSSR-Cp | 114654 | 114663 | TTTCA | 2 |  |  |
| cH0000118 | PotentialSSR | HexaSSR-Cp | 114752 | 114763 | AATACC | 2 | ndhD | YP_009380264.1 [ndhD]\| |
| cM0000027 | SSR | MonoSSR-Cp | 114818 | 114827 | A | 10 | ndhD | YP_009380264.1 [ndhD]\| |
| cH0000119 | PotentialSSR | HexaSSR-Cp | 115748 | 115759 | TAATTC | 2 | ndhD | YP_009380264.1 [ndhD]\| |
| cH0000120 | PotentialSSR | HexaSSR-Cp | 116600 | 116611 | TCTAGT | 2 |  |  |
| cP0000240 | PotentialSSR | PentaSSR-Cp | 117235 | 117244 | AAGTT | 2 |  |  |
| cP0000241 | PotentialSSR | PentaSSR-Cp | 117535 | 117544 | ATACC | 2 | ndhG | YP_009380267.1 [ndhG]\| |
| c70000030 | ExtendedSSR | 7SSR-Cp | 117747 | 117760 | TAGAATA | 2 | ndhG | YP_009380267.1 [ndhG]\| |
| cP0000242 | PotentialSSR | PentaSSR-Cp | 118022 | 118031 | TAAAG | 2 |  |  |
| cP0000243 | PotentialSSR | PentaSSR-Cp | 118187 | 118196 | TTTAA | 2 |  |  |
| cP0000244 | PotentialSSR | PentaSSR-Cp | 118308 | 118317 | TAATT | 2 | ndhI | YP_009380268.1 [ndhI]\| |
| cH0000121 | PotentialSSR | HexaSSR-Cp | 118950 | 118961 | GAACAA | 2 | ndhA | YP_009380269.1 [ndhA]\| |
| cP0000245 | PotentialSSR | PentaSSR-Cp | 118974 | 118983 | TAATG | 2 | ndhA | YP_009380269.1 [ndhA]\| |
| cP0000246 | PotentialSSR | PentaSSR-Cp | 119127 | 119136 | ATAAA | 2 | ndhA | YP_009380269.1 [ndhA]\| |
| cP0000247 | PotentialSSR | PentaSSR-Cp | 119355 | 119364 | AAGAT | 2 | ndhA | (Intron)YP_009380269.1 [ndhA]\| |
| cP0000248 | PotentialSSR | PentaSSR-Cp | 119543 | 119552 | CTATA | 2 | ndhA | (Intron)YP_009380269.1 [ndhA]\| |
| c70000031 | ExtendedSSR | 7SSR-Cp | 119665 | 119678 | TATCAAT | 2 | ndhA | (Intron)YP_009380269.1 [ndhA]\| |
| cM0000028 | SSR | MonoSSR-Cp | 119794 | 119803 | T | 10 | ndhA | (Intron)YP_009380269.1 [ndhA]\| |
| cH0000122 | PotentialSSR | HexaSSR-Cp | 119808 | 119819 | CTATTA | 2 | ndhA | (Intron)YP_009380269.1 [ndhA]\| |
| cP0000249 | PotentialSSR | PentaSSR-Cp | 121259 | 121268 | CATTC | 2 | ndhH | YP_009380270.1 [ndhH]\| |
| cH0000123 | PotentialSSR | HexaSSR-Cp | 122268 | 122279 | ATAATT | 2 |  |  |
| cP0000250 | PotentialSSR | PentaSSR-Cp | 122673 | 122682 | TTTAT | 2 |  |  |
| cH0000124 | PotentialSSR | HexaSSR-Cp | 122958 | 122969 | AATTTT | 2 | ycf1 | YP_009380272.1 [ycf1]\| |
| cP0000251 | PotentialSSR | PentaSSR-Cp | 123398 | 123407 | TTCTT | 2 | ycf1 | YP_009380272.1 [ycf1]\| |
| cM0000029 | SSR | MonoSSR-Cp | 123976 | 123988 | T | 13 | ycf1 | YP_009380272.1 [ycf1]\| |
| cH0000125 | PotentialSSR | HexaSSR-Cp | 124344 | 124355 | CTATAT | 2 | ycf1 | YP_009380272.1 [ycf1]\| |
| cH0000126 | PotentialSSR | HexaSSR-Cp | 124442 | 124453 | CAATAA | 2 | ycf1 | YP_009380272.1 [ycf1]\| |
| cTe0000009 | SSR | TetraSSR-Cp | 124476 | 124487 | TAAT | 3 | ycf1 | YP_009380272.1 [ycf1]\| |
| cM0000030 | SSR | MonoSSR-Cp | 125522 | 125532 | A | 11 | ycf1 | YP_009380272.1 [ycf1]\| |
| cP0000252 | PotentialSSR | PentaSSR-Cp | 125687 | 125696 | AAAAC | 2 | ycf1 | YP_009380272.1 [ycf1]\| |
| cP0000253 | PotentialSSR | PentaSSR-Cp | 127023 | 127032 | ATTTT | 2 | ycf1 | YP_009380272.1 [ycf1]\| |
| cM0000031 | SSR | MonoSSR-Cp | 127137 | 127146 | T | 10 | ycf1 | YP_009380272.1 [ycf1]\| |
| cP0000254 | PotentialSSR | PentaSSR-Cp | 127322 | 127331 | CATTT | 2 | ycf1 | YP_009380272.1 [ycf1]\| |
| cP0000255 | PotentialSSR | PentaSSR-Cp | 127548 | 127557 | TTTGT | 2 | ycf1 | YP_009380272.1 [ycf1]\| |
| cH0000127 | PotentialSSR | HexaSSR-Cp | 127593 | 127604 | ATTTTG | 2 | ycf1 | YP_009380272.1 [ycf1]\| |
| cP0000256 | PotentialSSR | PentaSSR-Cp | 127614 | 127623 | TTCTT | 2 | ycf1 | YP_009380272.1 [ycf1]\| |
| cH0000128 | PotentialSSR | HexaSSR-Cp | 127635 | 127646 | GAAGGA | 2 | ycf1 | YP_009380272.1 [ycf1]\| |
| cH0000129 | PotentialSSR | HexaSSR-Cp | 127702 | 127713 | TTTCTA | 2 | ycf1 | YP_009380272.1 [ycf1]\| |
| c90000005 | ExtendedSSR | 9SSR-Cp | 128318 | 128335 | TCCTTCTTC | 2 | ycf1 | YP_009380272.1 [ycf1]\| |
| c70000032 | ExtendedSSR | 7SSR-Cp | 128881 | 128894 | TTCATTC | 2 |  |  |
| cP0000257 | PotentialSSR | PentaSSR-Cp | 128993 | 129002 | TTCTG | 2 |  |  |
| c70000033 | ExtendedSSR | 7SSR-Cp | 129161 | 129174 | AAACATA | 2 |  |  |
| cP0000258 | PotentialSSR | PentaSSR-Cp | 129178 | 129187 | ATGAC | 2 |  |  |
| cP0000259 | PotentialSSR | PentaSSR-Cp | 129202 | 129211 | TGCTA | 2 |  |  |
| cH0000131 | PotentialSSR | HexaSSR-Cp | 129216 | 129227 | TACTTG | 2 |  |  |
| cP0000260 | PotentialSSR | PentaSSR-Cp | 129357 | 129366 | TCCAC | 2 |  |  |
| cH0000132 | PotentialSSR | HexaSSR-Cp | 129652 | 129663 | ATAAGA | 2 |  |  |
| cH0000133 | PotentialSSR | HexaSSR-Cp | 130030 | 130041 | GATAGA | 2 |  |  |
| c70000034 | ExtendedSSR | 7SSR-Cp | 130058 | 130071 | GGTTTTT | 2 |  |  |
| cP0000261 | PotentialSSR | PentaSSR-Cp | 130134 | 130143 | TGGAT | 2 |  |  |
| cP0000262 | PotentialSSR | PentaSSR-Cp | 130432 | 130441 | TCCGC | 2 | rrn23 | rrn23 [rrn23]\| |
| cTe0000010 | SSR | TetraSSR-Cp | 131521 | 131532 | CTAC | 3 | rrn23 | rrn23 [rrn23]\| |
| cH0000134 | PotentialSSR | HexaSSR-Cp | 131917 | 131928 | CGCTTC | 2 | rrn23 | rrn23 [rrn23]\| |
| cD0000009 | SSR | DiSSR-Cp | 131942 | 131951 | CG | 5 | rrn23 | rrn23 [rrn23]\| |
| c80000011 | ExtendedSSR | 8SSR-Cp | 133511 | 133526 | TCTCAAAA | 2 | trnA-UGC | (Intron)trnA-UGC [trnA-UGC]\| |
| cP0000263 | PotentialSSR | PentaSSR-Cp | 133740 | 133749 | ATTGA | 2 | trnA-UGC | (Intron)trnA-UGC [trnA-UGC]\| |
| cP0000264 | PotentialSSR | PentaSSR-Cp | 134060 | 134069 | TTTGT | 2 | trnA-UGC | (Intron)trnA-UGC [trnA-UGC]\| |
| cH0000135 | PotentialSSR | HexaSSR-Cp | 134289 | 134300 | ATTCTT | 2 |  |  |
| cH0000136 | PotentialSSR | HexaSSR-Cp | 135045 | 135056 | TCCATT | 2 | trnI-GAU | (Intron)trnI-GAU [trnI-GAU]\| |
| cH0000137 | PotentialSSR | HexaSSR-Cp | 136500 | 136511 | TCAGTG | 2 | rrn16 | rrn16 [rrn16]\| |
| cH0000138 | PotentialSSR | HexaSSR-Cp | 137375 | 137386 | TCATTC | 2 |  |  |
| cP0000265 | PotentialSSR | PentaSSR-Cp | 137501 | 137510 | TCCCT | 2 | trnV-GAC | trnV-GAC [trnV-GAC]\| |
| cP0000266 | PotentialSSR | PentaSSR-Cp | 137584 | 137593 | TCTTG | 2 |  |  |
| cM0000032 | SSR | MonoSSR-Cp | 137833 | 137844 | A | 12 |  |  |
| cH0000139 | PotentialSSR | HexaSSR-Cp | 137941 | 137952 | TTTCCA | 2 |  |  |
| cH0000140 | PotentialSSR | HexaSSR-Cp | 138087 | 138098 | ATAGTA | 2 |  |  |
| cP0000267 | PotentialSSR | PentaSSR-Cp | 138211 | 138220 | CATTG | 2 |  |  |
| cP0000268 | PotentialSSR | PentaSSR-Cp | 138394 | 138403 | CTAAT | 2 |  |  |
| cP0000269 | PotentialSSR | PentaSSR-Cp | 138413 | 138422 | TAATA | 2 |  |  |
| cP0000270 | PotentialSSR | PentaSSR-Cp | 138511 | 138520 | CAACA | 2 |  |  |
| cH0000141 | PotentialSSR | HexaSSR-Cp | 138755 | 138766 | GGGATA | 2 |  |  |
| cH0000142 | PotentialSSR | HexaSSR-Cp | 138767 | 138778 | GAGGAA | 2 |  |  |
| cH0000143 | PotentialSSR | HexaSSR-Cp | 138812 | 138823 | AAAATC | 2 |  |  |
| cP0000271 | PotentialSSR | PentaSSR-Cp | 139404 | 139413 | GATTC | 2 |  |  |
| cH0000144 | PotentialSSR | HexaSSR-Cp | 139591 | 139602 | TATGGA | 2 |  |  |
| cP0000272 | PotentialSSR | PentaSSR-Cp | 140339 | 140348 | GAACA | 2 |  |  |
| cP0000273 | PotentialSSR | PentaSSR-Cp | 140444 | 140453 | TTTCT | 2 |  |  |
| cH0000145 | PotentialSSR | HexaSSR-Cp | 140508 | 140519 | TAAGAA | 2 |  |  |
| cP0000274 | PotentialSSR | PentaSSR-Cp | 141577 | 141586 | TCTTT | 2 | ndhB | (Intron)YP_009380274.1 [ndhB]\| |
| cP0000275 | PotentialSSR | PentaSSR-Cp | 141777 | 141786 | AATCA | 2 | ndhB | (Intron)YP_009380274.1 [ndhB]\| |
| cH0000146 | PotentialSSR | HexaSSR-Cp | 142071 | 142082 | GACTCT | 2 | ndhB | YP_009380274.1 [ndhB]\| |
| cH0000147 | PotentialSSR | HexaSSR-Cp | 142128 | 142139 | GCTTCA | 2 | ndhB | YP_009380274.1 [ndhB]\| |
| cP0000276 | PotentialSSR | PentaSSR-Cp | 143092 | 143101 | TTCCA | 2 |  |  |
| cP0000277 | PotentialSSR | PentaSSR-Cp | 143228 | 143237 | ATAAG | 2 |  |  |
| cH0000148 | PotentialSSR | HexaSSR-Cp | 143541 | 143552 | TTGGAA | 2 |  |  |
| cP0000278 | PotentialSSR | PentaSSR-Cp | 143718 | 143727 | CGTAA | 2 |  |  |
| cP0000279 | PotentialSSR | PentaSSR-Cp | 143963 | 143972 | AACAA | 2 |  |  |
| cP0000280 | PotentialSSR | PentaSSR-Cp | 143991 | 144000 | AACTT | 2 |  |  |
| cH0000149 | PotentialSSR | HexaSSR-Cp | 144483 | 144494 | ATATAG | 2 | ycf2 | YP_009380275.1 [ycf2]\| |
| cH0000150 | PotentialSSR | HexaSSR-Cp | 145083 | 145094 | CTTCTA | 2 | ycf2 | YP_009380275.1 [ycf2]\| |
| cP0000281 | PotentialSSR | PentaSSR-Cp | 145590 | 145599 | TTTTC | 2 | ycf2 | YP_009380275.1 [ycf2]\| |
| cH0000151 | PotentialSSR | HexaSSR-Cp | 145969 | 145980 | CAGCTC | 2 | ycf2 | YP_009380275.1 [ycf2]\| |
| cP0000282 | PotentialSSR | PentaSSR-Cp | 146613 | 146622 | CAATC | 2 | ycf2 | YP_009380275.1 [ycf2]\| |
| cH0000152 | PotentialSSR | HexaSSR-Cp | 146633 | 146644 | CTTTTT | 2 | ycf2 | YP_009380275.1 [ycf2]\| |
| cH0000153 | PotentialSSR | HexaSSR-Cp | 146812 | 146823 | GGGACC | 2 | ycf2 | YP_009380275.1 [ycf2]\| |
| cP0000283 | PotentialSSR | PentaSSR-Cp | 147591 | 147600 | TTGAA | 2 | ycf2 | YP_009380275.1 [ycf2]\| |
| cP0000284 | PotentialSSR | PentaSSR-Cp | 147756 | 147765 | GATCG | 2 | ycf2 | YP_009380275.1 [ycf2]\| |
| c90000006 | ExtendedSSR | 9SSR-Cp | 148595 | 148612 | AAATGTTCC | 2 | ycf2 | YP_009380275.1 [ycf2]\| |
| cP0000285 | PotentialSSR | PentaSSR-Cp | 149882 | 149891 | CGGAT | 2 | ycf2 | YP_009380275.1 [ycf2]\| |
| cH0000155 | PotentialSSR | HexaSSR-Cp | 149933 | 149944 | TCTTTC | 2 | ycf2 | YP_009380275.1 [ycf2]\| |
| cP0000286 | PotentialSSR | PentaSSR-Cp | 150600 | 150609 | ATTTC | 2 |  |  |
| cP0000287 | PotentialSSR | PentaSSR-Cp | 150817 | 150826 | TCATA | 2 |  |  |
| cP0000288 | PotentialSSR | PentaSSR-Cp | 151175 | 151184 | TATTC | 2 |  |  |
| cP0000289 | PotentialSSR | PentaSSR-Cp | 151195 | 151204 | AAAGA | 2 |  |  |
| cP0000290 | PotentialSSR | PentaSSR-Cp | 151296 | 151305 | AATCC | 2 | rpl2 | YP_009380276.1 [rpl2]\| |
| cP0000291 | PotentialSSR | PentaSSR-Cp | 151645 | 151654 | ACATA | 2 | rpl2 | YP_009380276.1 [rpl2]\| |
| cH0000156 | PotentialSSR | HexaSSR-Cp | 152058 | 152069 | GAAAAT | 2 |  |  |

**Supplementary Table 6. List of SSRs identified in CVHUP01 of *C. album***

| **Name** | **SSR type** | **Type** | **Cooridnation** | | **Unit sequence** | **Repeat number** | **Genes** | **Annotation** |
| --- | --- | --- | --- | --- | --- | --- | --- | --- |
| cH0000001 | PotentialSSR | HexaSSR-Cp | 113 | 124 | GTAAAG | 2 |  |  |
| cH0000002 | PotentialSSR | HexaSSR-Cp | 1234 | 1245 | GCTTTC | 2 | psbA | YP_009380194.1 [psbA]\| |
| cP0000001 | PotentialSSR | PentaSSR-Cp | 1286 | 1295 | ATTTA | 2 |  |  |
| c70000001 | ExtendedSSR | 7SSR-Cp | 1448 | 1461 | AGAAAAT | 2 |  |  |
| c70000002 | ExtendedSSR | 7SSR-Cp | 1690 | 1703 | AGTAGAA | 2 | trnK-UUU | (Intron)trnK-UUU [trnK-UUU]\| |
| cH0000003 | PotentialSSR | HexaSSR-Cp | 1742 | 1753 | AATTTC | 2 | trnK-UUU | (Intron)trnK-UUU [trnK-UUU]\| |
| cH0000004 | PotentialSSR | HexaSSR-Cp | 2949 | 2960 | TTTTTC | 2 | matK,trnK-UUU | YP_009380195.1 [matK]\|(Intron)trnK-UUU [trnK-UUU]\| |
| cP0000002 | PotentialSSR | PentaSSR-Cp | 4093 | 4102 | TATGT | 2 |  |  |
| cP0000003 | PotentialSSR | PentaSSR-Cp | 4124 | 4133 | AATGG | 2 |  |  |
| cP0000004 | PotentialSSR | PentaSSR-Cp | 4202 | 4211 | CAGAT | 2 |  |  |
| cP0000005 | PotentialSSR | PentaSSR-Cp | 4355 | 4364 | CATTT | 2 |  |  |
| cH0000005 | PotentialSSR | HexaSSR-Cp | 4442 | 4453 | AATATT | 2 |  |  |
| cTe0000001 | SSR | TetraSSR-Cp | 4464 | 4475 | TTTA | 3 |  |  |
| cP0000006 | PotentialSSR | PentaSSR-Cp | 4487 | 4496 | ATTTA | 2 |  |  |
| cP0000007 | PotentialSSR | PentaSSR-Cp | 4572 | 4581 | ATATA | 2 |  |  |
| cT0000001 | SSR | TriSSR-Cp | 4613 | 4624 | ATA | 4 |  |  |
| cP0000008 | PotentialSSR | PentaSSR-Cp | 4970 | 4979 | TAGAT | 2 | rps16 | (Intron)YP_009380196.1 [rps16]\| |
| cP0000009 | PotentialSSR | PentaSSR-Cp | 5204 | 5213 | CATTT | 2 | rps16 | (Intron)YP_009380196.1 [rps16]\| |
| cH0000007 | PotentialSSR | HexaSSR-Cp | 5274 | 5285 | ATCCAA | 2 | rps16 | (Intron)YP_009380196.1 [rps16]\| |
| c70000004 | ExtendedSSR | 7SSR-Cp | 5294 | 5307 | ACAATTA | 2 | rps16 | (Intron)YP_009380196.1 [rps16]\| |
| cM0000001 | SSR | MonoSSR-Cp | 5341 | 5352 | T | 12 | rps16 | (Intron)YP_009380196.1 [rps16]\| |
| cP0000010 | PotentialSSR | PentaSSR-Cp | 5442 | 5451 | AGAAT | 2 | rps16 | (Intron)YP_009380196.1 [rps16]\| |
| cP0000011 | PotentialSSR | PentaSSR-Cp | 5998 | 6007 | AATGC | 2 |  |  |
| cP0000012 | SSR | PentaSSR-Cp | 6081 | 6095 | TTAAT | 3 |  |  |
| cP0000013 | PotentialSSR | PentaSSR-Cp | 6191 | 6200 | TATAT | 2 |  |  |
| cH0000009 | PotentialSSR | HexaSSR-Cp | 6468 | 6479 | GCTCTG | 2 |  |  |
| cP0000014 | PotentialSSR | PentaSSR-Cp | 6556 | 6565 | TTCTA | 2 |  |  |
| cP0000015 | PotentialSSR | PentaSSR-Cp | 6630 | 6639 | GATTC | 2 |  |  |
| cM0000002 | SSR | MonoSSR-Cp | 6788 | 6798 | A | 11 |  |  |
| cM0000003 | SSR | MonoSSR-Cp | 6854 | 6863 | A | 10 |  |  |
| c80000001 | ExtendedSSR | 8SSR-Cp | 7101 | 7116 | ATTCTAAT | 2 |  |  |
| cH0000010 | PotentialSSR | HexaSSR-Cp | 7346 | 7357 | TTACAA | 2 |  |  |
| cP0000016 | PotentialSSR | PentaSSR-Cp | 7399 | 7408 | TCAAA | 2 |  |  |
| cP0000017 | PotentialSSR | PentaSSR-Cp | 7609 | 7618 | TTCTT | 2 |  |  |
| cP0000018 | PotentialSSR | PentaSSR-Cp | 7641 | 7650 | ATTCT | 2 |  |  |
| cP0000019 | PotentialSSR | PentaSSR-Cp | 7720 | 7729 | TTATA | 2 |  |  |
| cM0000004 | SSR | MonoSSR-Cp | 7735 | 7745 | A | 11 |  |  |
| cP0000020 | PotentialSSR | PentaSSR-Cp | 7901 | 7910 | GAAAA | 2 |  |  |
| cP0000021 | PotentialSSR | PentaSSR-Cp | 7991 | 8000 | ATATA | 2 |  |  |
| cH0000012 | PotentialSSR | HexaSSR-Cp | 8071 | 8082 | AGATAA | 2 |  |  |
| cP0000022 | PotentialSSR | PentaSSR-Cp | 8139 | 8148 | TAAAA | 2 |  |  |
| cP0000023 | PotentialSSR | PentaSSR-Cp | 8215 | 8224 | AAAGA | 2 |  |  |
| cP0000024 | PotentialSSR | PentaSSR-Cp | 8270 | 8279 | GGCCT | 2 |  |  |
| cH0000013 | PotentialSSR | HexaSSR-Cp | 8348 | 8359 | TTTGAT | 2 |  |  |
| cP0000025 | PotentialSSR | PentaSSR-Cp | 8864 | 8873 | AATTG | 2 | trnG-UCC | (Intron)trnG-UCC [trnG-UCC]\| |
| cP0000026 | PotentialSSR | PentaSSR-Cp | 9229 | 9238 | TTTTG | 2 | trnG-UCC | (Intron)trnG-UCC [trnG-UCC]\| |
| cP0000027 | PotentialSSR | PentaSSR-Cp | 9254 | 9263 | CTTAA | 2 | trnG-UCC | (Intron)trnG-UCC [trnG-UCC]\| |
| cP0000028 | PotentialSSR | PentaSSR-Cp | 9287 | 9296 | TTTTC | 2 | trnG-UCC | (Intron)trnG-UCC [trnG-UCC]\| |
| cP0000029 | PotentialSSR | PentaSSR-Cp | 9485 | 9494 | TCTCA | 2 |  |  |
| cP0000030 | PotentialSSR | PentaSSR-Cp | 9507 | 9516 | CAAAA | 2 |  |  |
| cM0000005 | SSR | MonoSSR-Cp | 9533 | 9543 | A | 11 |  |  |
| cH0000016 | PotentialSSR | HexaSSR-Cp | 10449 | 10460 | GCTTGT | 2 | atpA | YP_009380199.1 [atpA]\| |
| cH0000017 | PotentialSSR | HexaSSR-Cp | 10852 | 10863 | CGGGAG | 2 | atpA | YP_009380199.1 [atpA]\| |
| c80000002 | ExtendedSSR | 8SSR-Cp | 11973 | 11988 | AAAAATAG | 2 | atpF | (Intron)YP_009380200.1 [atpF]\| |
| c70000008 | ExtendedSSR | 7SSR-Cp | 12216 | 12229 | TCGGTAT | 2 | atpF | (Intron)YP_009380200.1 [atpF]\| |
| cT0000002 | SSR | TriSSR-Cp | 12322 | 12333 | ATT | 4 | atpF | (Intron)YP_009380200.1 [atpF]\| |
| cTe0000002 | SSR | TetraSSR-Cp | 12887 | 12898 | GGAA | 3 |  |  |
| cP0000031 | PotentialSSR | PentaSSR-Cp | 13035 | 13044 | AGAAA | 2 |  |  |
| cP0000032 | PotentialSSR | PentaSSR-Cp | 13060 | 13069 | TTTCT | 2 |  |  |
| cD0000001 | SSR | DiSSR-Cp | 13331 | 13340 | CA | 5 |  |  |
| cP0000033 | PotentialSSR | PentaSSR-Cp | 13822 | 13831 | AAATA | 2 |  |  |
| cD0000002 | SSR | DiSSR-Cp | 13889 | 13898 | AT | 5 |  |  |
| cH0000018 | PotentialSSR | HexaSSR-Cp | 14262 | 14273 | TAAAGC | 2 | atpI | YP_009380202.1 [atpI]\| |
| cM0000006 | SSR | MonoSSR-Cp | 14714 | 14723 | A | 10 |  |  |
| cH0000019 | PotentialSSR | HexaSSR-Cp | 14762 | 14773 | TTTAAT | 2 |  |  |
| cP0000034 | PotentialSSR | PentaSSR-Cp | 15632 | 15641 | ATTAA | 2 |  |  |
| c80000003 | ExtendedSSR | 8SSR-Cp | 15691 | 15706 | TCTACCGC | 2 |  |  |
| cP0000035 | PotentialSSR | PentaSSR-Cp | 15770 | 15779 | TTTAT | 2 |  |  |
| cP0000036 | PotentialSSR | PentaSSR-Cp | 15783 | 15792 | TAAAT | 2 |  |  |
| cP0000037 | PotentialSSR | PentaSSR-Cp | 16847 | 16856 | CAATT | 2 | rpoC2 | YP_009380204.1 [rpoC2]\| |
| cP0000038 | PotentialSSR | PentaSSR-Cp | 17662 | 17671 | CAAAA | 2 | rpoC2 | YP_009380204.1 [rpoC2]\| |
| cP0000039 | PotentialSSR | PentaSSR-Cp | 17782 | 17791 | TATCT | 2 | rpoC2 | YP_009380204.1 [rpoC2]\| |
| cM0000007 | SSR | MonoSSR-Cp | 17838 | 17850 | T | 13 | rpoC2 | YP_009380204.1 [rpoC2]\| |
| cP0000040 | PotentialSSR | PentaSSR-Cp | 18321 | 18330 | CGATT | 2 | rpoC2 | YP_009380204.1 [rpoC2]\| |
| cP0000041 | PotentialSSR | PentaSSR-Cp | 18350 | 18359 | ATCCT | 2 | rpoC2 | YP_009380204.1 [rpoC2]\| |
| cH0000020 | PotentialSSR | HexaSSR-Cp | 18463 | 18474 | TTGATC | 2 | rpoC2 | YP_009380204.1 [rpoC2]\| |
| cH0000021 | PotentialSSR | HexaSSR-Cp | 18835 | 18846 | ACGTGT | 2 | rpoC2 | YP_009380204.1 [rpoC2]\| |
| cP0000042 | PotentialSSR | PentaSSR-Cp | 19047 | 19056 | CATAA | 2 | rpoC2 | YP_009380204.1 [rpoC2]\| |
| cD0000003 | SSR | DiSSR-Cp | 19190 | 19199 | AT | 5 | rpoC2 | YP_009380204.1 [rpoC2]\| |
| cH0000022 | PotentialSSR | HexaSSR-Cp | 21529 | 21540 | CAAATC | 2 | rpoC1 | YP_009380205.1 [rpoC1]\| |
| cP0000043 | PotentialSSR | PentaSSR-Cp | 21643 | 21652 | GGATT | 2 | rpoC1 | YP_009380205.1 [rpoC1]\| |
| cP0000044 | PotentialSSR | PentaSSR-Cp | 21732 | 21741 | ATCCT | 2 | rpoC1 | (Intron)YP_009380205.1 [rpoC1]\| |
| cP0000045 | PotentialSSR | PentaSSR-Cp | 21926 | 21935 | TTCTT | 2 | rpoC1 | (Intron)YP_009380205.1 [rpoC1]\| |
| cP0000046 | PotentialSSR | PentaSSR-Cp | 21958 | 21967 | AATTA | 2 | rpoC1 | (Intron)YP_009380205.1 [rpoC1]\| |
| cP0000047 | PotentialSSR | PentaSSR-Cp | 22142 | 22151 | TTAAA | 2 | rpoC1 | (Intron)YP_009380205.1 [rpoC1]\| |
| cH0000023 | PotentialSSR | HexaSSR-Cp | 22247 | 22258 | ACAAAA | 2 | rpoC1 | (Intron)YP_009380205.1 [rpoC1]\| |
| cM0000008 | SSR | MonoSSR-Cp | 25515 | 25524 | T | 10 | rpoB | YP_009380206.1 [rpoB]\| |
| cH0000024 | PotentialSSR | HexaSSR-Cp | 25958 | 25969 | TCTTTT | 2 | rpoB | YP_009380206.1 [rpoB]\| |
| cP0000048 | PotentialSSR | PentaSSR-Cp | 26437 | 26446 | ACTTA | 2 |  |  |
| cP0000049 | PotentialSSR | PentaSSR-Cp | 26730 | 26739 | TTGTA | 2 |  |  |
| cH0000026 | PotentialSSR | HexaSSR-Cp | 26891 | 26902 | AGAAAA | 2 |  |  |
| cP0000050 | PotentialSSR | PentaSSR-Cp | 27107 | 27116 | GAATC | 2 |  |  |
| cP0000051 | PotentialSSR | PentaSSR-Cp | 27567 | 27576 | TTCCC | 2 |  |  |
| cP0000052 | PotentialSSR | PentaSSR-Cp | 27696 | 27705 | CTGAG | 2 |  |  |
| cH0000027 | PotentialSSR | HexaSSR-Cp | 27808 | 27819 | TTCATA | 2 |  |  |
| cP0000053 | PotentialSSR | PentaSSR-Cp | 27940 | 27949 | TTTAG | 2 |  |  |
| cH0000028 | PotentialSSR | HexaSSR-Cp | 27966 | 27977 | TATCAT | 2 |  |  |
| cP0000054 | PotentialSSR | PentaSSR-Cp | 28199 | 28208 | ATTTC | 2 |  |  |
| cP0000055 | PotentialSSR | PentaSSR-Cp | 28267 | 28276 | TCAAT | 2 |  |  |
| cP0000056 | PotentialSSR | PentaSSR-Cp | 28293 | 28302 | TTGGC | 2 |  |  |
| cP0000057 | PotentialSSR | PentaSSR-Cp | 28351 | 28360 | ACTTT | 2 |  |  |
| c70000009 | ExtendedSSR | 7SSR-Cp | 28418 | 28431 | TTATAGT | 2 |  |  |
| cP0000058 | PotentialSSR | PentaSSR-Cp | 28455 | 28464 | CTAAT | 2 |  |  |
| cP0000059 | PotentialSSR | PentaSSR-Cp | 28831 | 28840 | TATCA | 2 |  |  |
| cH0000029 | PotentialSSR | HexaSSR-Cp | 29056 | 29067 | CAAAAA | 2 |  |  |
| cM0000009 | SSR | MonoSSR-Cp | 29376 | 29385 | A | 10 |  |  |
| cP0000060 | PotentialSSR | PentaSSR-Cp | 29754 | 29763 | ATAAA | 2 |  |  |
| c80000004 | ExtendedSSR | 8SSR-Cp | 29773 | 29788 | TTTTCTTT | 2 |  |  |
| cP0000061 | PotentialSSR | PentaSSR-Cp | 29830 | 29839 | ACCAT | 2 |  |  |
| cH0000030 | PotentialSSR | HexaSSR-Cp | 29870 | 29881 | AATTTC | 2 |  |  |
| cTe0000003 | SSR | TetraSSR-Cp | 29889 | 29900 | AAAT | 3 |  |  |
| cP0000062 | PotentialSSR | PentaSSR-Cp | 30108 | 30117 | TCAAT | 2 |  |  |
| cP0000063 | PotentialSSR | PentaSSR-Cp | 30159 | 30168 | TACCC | 2 | trnE-UUC | trnE-UUC [trnE-UUC]\| |
| cH0000031 | PotentialSSR | HexaSSR-Cp | 30363 | 30374 | ATTTCA | 2 |  |  |
| cH0000032 | PotentialSSR | HexaSSR-Cp | 30501 | 30512 | CTAATA | 2 |  |  |
| cP0000064 | PotentialSSR | PentaSSR-Cp | 30645 | 30654 | GTATA | 2 |  |  |
| cP0000065 | PotentialSSR | PentaSSR-Cp | 30957 | 30966 | ATTAT | 2 |  |  |
| cP0000066 | PotentialSSR | PentaSSR-Cp | 31030 | 31039 | TGGAA | 2 |  |  |
| c70000010 | ExtendedSSR | 7SSR-Cp | 31491 | 31504 | GTTTATT | 2 |  |  |
| cH0000033 | PotentialSSR | HexaSSR-Cp | 31568 | 31579 | GTGAAA | 2 |  |  |
| cP0000067 | PotentialSSR | PentaSSR-Cp | 31744 | 31753 | AAAGA | 2 |  |  |
| cP0000068 | PotentialSSR | PentaSSR-Cp | 31951 | 31960 | TAAAT | 2 |  |  |
| cP0000069 | PotentialSSR | PentaSSR-Cp | 32114 | 32123 | CGTTT | 2 | psbD | YP_009380209.1 [psbD]\| |
| cP0000070 | PotentialSSR | PentaSSR-Cp | 32744 | 32753 | AACCC | 2 | psbD | YP_009380209.1 [psbD]\| |
| cP0000071 | PotentialSSR | PentaSSR-Cp | 33837 | 33846 | GTCTG | 2 | psbC | YP_009380210.1 [psbC]\| |
| cH0000034 | PotentialSSR | HexaSSR-Cp | 33973 | 33984 | CTCAAG | 2 | psbC | YP_009380210.1 [psbC]\| |
| cP0000072 | PotentialSSR | PentaSSR-Cp | 34265 | 34274 | GGGTG | 2 | psbC | YP_009380210.1 [psbC]\| |
| cH0000035 | PotentialSSR | HexaSSR-Cp | 34391 | 34402 | TGCAGC | 2 | psbC | YP_009380210.1 [psbC]\| |
| c70000011 | ExtendedSSR | 7SSR-Cp | 34514 | 34527 | TACATAT | 2 |  |  |
| cP0000073 | PotentialSSR | PentaSSR-Cp | 34592 | 34601 | TAATT | 2 |  |  |
| cH0000036 | PotentialSSR | HexaSSR-Cp | 34823 | 34834 | TTATTC | 2 |  |  |
| cP0000074 | PotentialSSR | PentaSSR-Cp | 34925 | 34934 | TATAT | 2 |  |  |
| c70000012 | ExtendedSSR | 7SSR-Cp | 34965 | 34978 | TTAATTA | 2 |  |  |
| cP0000075 | PotentialSSR | PentaSSR-Cp | 35301 | 35310 | TGGAT | 2 | psbZ | YP_009380211.1 [psbZ]\| |
| cP0000076 | PotentialSSR | PentaSSR-Cp | 35469 | 35478 | AAACA | 2 |  |  |
| cP0000077 | PotentialSSR | PentaSSR-Cp | 35632 | 35641 | TATAT | 2 |  |  |
| cH0000037 | PotentialSSR | HexaSSR-Cp | 35798 | 35809 | GTTATA | 2 |  |  |
| cP0000078 | PotentialSSR | PentaSSR-Cp | 35821 | 35830 | TAGTG | 2 |  |  |
| cP0000079 | PotentialSSR | PentaSSR-Cp | 35924 | 35933 | TATTT | 2 |  |  |
| cP0000080 | PotentialSSR | PentaSSR-Cp | 36835 | 36844 | CCACG | 2 | psaB | YP_009380213.1 [psaB]\| |
| cP0000081 | PotentialSSR | PentaSSR-Cp | 36984 | 36993 | CCATC | 2 | psaB | YP_009380213.1 [psaB]\| |
| cP0000082 | PotentialSSR | PentaSSR-Cp | 38263 | 38272 | TGTCC | 2 | psaB | YP_009380213.1 [psaB]\| |
| cP0000083 | PotentialSSR | PentaSSR-Cp | 38769 | 38778 | ACCAA | 2 | psaB | YP_009380213.1 [psaB]\| |
| cH0000038 | PotentialSSR | HexaSSR-Cp | 39195 | 39206 | TAATAG | 2 | psaA | YP_009380214.1 [psaA]\| |
| cP0000084 | PotentialSSR | PentaSSR-Cp | 39944 | 39953 | ATGTG | 2 | psaA | YP_009380214.1 [psaA]\| |
| cP0000085 | PotentialSSR | PentaSSR-Cp | 41581 | 41590 | TATTT | 2 |  |  |
| cH0000039 | PotentialSSR | HexaSSR-Cp | 41759 | 41770 | TCTTTA | 2 |  |  |
| cP0000086 | PotentialSSR | PentaSSR-Cp | 41853 | 41862 | TTTTA | 2 |  |  |
| cM0000010 | SSR | MonoSSR-Cp | 41892 | 41901 | A | 10 |  |  |
| cP0000087 | PotentialSSR | PentaSSR-Cp | 42434 | 42443 | AAAGA | 2 | ycf3 | (Intron)YP_009380215.1 [ycf3]\| |
| cP0000088 | PotentialSSR | PentaSSR-Cp | 42571 | 42580 | TTCTT | 2 | ycf3 | (Intron)YP_009380215.1 [ycf3]\| |
| cM0000011 | SSR | MonoSSR-Cp | 42644 | 42653 | T | 10 | ycf3 | (Intron)YP_009380215.1 [ycf3]\| |
| cP0000089 | PotentialSSR | PentaSSR-Cp | 43145 | 43154 | AATAT | 2 | ycf3 | (Intron)YP_009380215.1 [ycf3]\| |
| cP0000090 | PotentialSSR | PentaSSR-Cp | 43820 | 43829 | AAATC | 2 | ycf3 | (Intron)YP_009380215.1 [ycf3]\| |
| cP0000091 | PotentialSSR | PentaSSR-Cp | 44029 | 44038 | GTACA | 2 |  |  |
| cP0000092 | PotentialSSR | PentaSSR-Cp | 44118 | 44127 | TATTT | 2 |  |  |
| cP0000093 | PotentialSSR | PentaSSR-Cp | 44205 | 44214 | CAAAT | 2 |  |  |
| cP0000094 | PotentialSSR | PentaSSR-Cp | 44219 | 44228 | TAGTA | 2 |  |  |
| cP0000095 | PotentialSSR | PentaSSR-Cp | 44272 | 44281 | GATCA | 2 |  |  |
| cP0000096 | PotentialSSR | PentaSSR-Cp | 44341 | 44350 | AATAG | 2 |  |  |
| cP0000097 | PotentialSSR | PentaSSR-Cp | 44518 | 44527 | GATTC | 2 |  |  |
| cTe0000004 | SSR | TetraSSR-Cp | 44533 | 44544 | AATT | 3 |  |  |
| cP0000098 | PotentialSSR | PentaSSR-Cp | 44553 | 44562 | TATAT | 2 |  |  |
| cD0000004 | SSR | DiSSR-Cp | 44625 | 44634 | AT | 5 |  |  |
| cP0000099 | PotentialSSR | PentaSSR-Cp | 44653 | 44662 | TTTTA | 2 |  |  |
| cP0000100 | PotentialSSR | PentaSSR-Cp | 44704 | 44713 | ATTAC | 2 |  |  |
| cP0000101 | PotentialSSR | PentaSSR-Cp | 44752 | 44761 | ATAGT | 2 |  |  |
| cP0000102 | PotentialSSR | PentaSSR-Cp | 44837 | 44846 | ATAGT | 2 |  |  |
| cP0000103 | PotentialSSR | PentaSSR-Cp | 45025 | 45034 | CAAAT | 2 |  |  |
| cP0000104 | PotentialSSR | PentaSSR-Cp | 45201 | 45210 | GGGAT | 2 |  |  |
| cH0000040 | PotentialSSR | HexaSSR-Cp | 45268 | 45279 | TTTTCC | 2 |  |  |
| cP0000105 | PotentialSSR | PentaSSR-Cp | 45694 | 45703 | ACCTG | 2 | rps4 | YP_009380216.1 [rps4]\| |
| cD0000005 | SSR | DiSSR-Cp | 45977 | 45990 | TA | 7 |  |  |
| cP0000106 | PotentialSSR | PentaSSR-Cp | 46035 | 46044 | AAATG | 2 |  |  |
| cP0000107 | PotentialSSR | PentaSSR-Cp | 46126 | 46135 | TATTT | 2 |  |  |
| cH0000041 | PotentialSSR | HexaSSR-Cp | 46155 | 46166 | AAAAAG | 2 |  |  |
| cP0000108 | PotentialSSR | PentaSSR-Cp | 46210 | 46219 | CTTTT | 2 |  |  |
| cP0000109 | PotentialSSR | PentaSSR-Cp | 46502 | 46511 | TCAAA | 2 |  |  |
| cP0000110 | PotentialSSR | PentaSSR-Cp | 46580 | 46589 | CGATT | 2 |  |  |
| cP0000111 | PotentialSSR | PentaSSR-Cp | 46816 | 46825 | AATAA | 2 |  |  |
| cP0000112 | PotentialSSR | PentaSSR-Cp | 47405 | 47414 | CTGTG | 2 | trnL-UAA | (Intron)trnL-UAA [trnL-UAA]\| |
| cP0000113 | PotentialSSR | PentaSSR-Cp | 47498 | 47507 | TCTAT | 2 | trnL-UAA | (Intron)trnL-UAA [trnL-UAA]\| |
| cP0000114 | PotentialSSR | PentaSSR-Cp | 47889 | 47898 | TTTTC | 2 |  |  |
| cD0000006 | SSR | DiSSR-Cp | 47946 | 47955 | AT | 5 |  |  |
| cP0000115 | PotentialSSR | PentaSSR-Cp | 48260 | 48269 | CACAT | 2 |  |  |
| cH0000043 | PotentialSSR | HexaSSR-Cp | 48389 | 48400 | GAAAAT | 2 |  |  |
| cP0000116 | PotentialSSR | PentaSSR-Cp | 48520 | 48529 | CTTAA | 2 |  |  |
| cP0000117 | PotentialSSR | PentaSSR-Cp | 48539 | 48548 | TCTTT | 2 |  |  |
| cH0000044 | PotentialSSR | HexaSSR-Cp | 49008 | 49019 | CATATA | 2 | ndhJ | YP_009380217.1 [ndhJ]\| |
| c80000006 | ExtendedSSR | 8SSR-Cp | 49165 | 49180 | ATTTGTTT | 2 |  |  |
| cP0000118 | PotentialSSR | PentaSSR-Cp | 49188 | 49197 | TATTT | 2 |  |  |
| cP0000119 | PotentialSSR | PentaSSR-Cp | 49201 | 49210 | TTTAC | 2 |  |  |
| cH0000045 | PotentialSSR | HexaSSR-Cp | 49968 | 49979 | TTTTTC | 2 |  |  |
| cP0000120 | PotentialSSR | PentaSSR-Cp | 50033 | 50042 | TAAAC | 2 | ndhC | YP_009380219.1 [ndhC]\| |
| cP0000121 | PotentialSSR | PentaSSR-Cp | 50595 | 50604 | TTCTT | 2 |  |  |
| cP0000122 | PotentialSSR | PentaSSR-Cp | 50671 | 50680 | AATTC | 2 |  |  |
| cP0000123 | PotentialSSR | PentaSSR-Cp | 50727 | 50736 | TTCTA | 2 |  |  |
| cP0000124 | PotentialSSR | PentaSSR-Cp | 50786 | 50795 | ACTTG | 2 |  |  |
| c70000015 | ExtendedSSR | 7SSR-Cp | 52543 | 52556 | AATTTTT | 2 |  |  |
| cP0000125 | PotentialSSR | PentaSSR-Cp | 52681 | 52690 | ATTTG | 2 |  |  |
| cP0000126 | PotentialSSR | PentaSSR-Cp | 52782 | 52791 | TTGAA | 2 |  |  |
| cH0000047 | PotentialSSR | HexaSSR-Cp | 52835 | 52846 | TGTATA | 2 |  |  |
| cP0000127 | PotentialSSR | PentaSSR-Cp | 53062 | 53071 | ATTGA | 2 |  |  |
| cP0000128 | PotentialSSR | PentaSSR-Cp | 53101 | 53110 | AATTG | 2 |  |  |
| cM0000012 | SSR | MonoSSR-Cp | 53192 | 53203 | T | 12 |  |  |
| cH0000048 | PotentialSSR | HexaSSR-Cp | 53214 | 53225 | ATTTTC | 2 |  |  |
| cM0000013 | SSR | MonoSSR-Cp | 53299 | 53308 | A | 10 | atpB | YP_009380221.1 [atpB]\| |
| cP0000129 | PotentialSSR | PentaSSR-Cp | 54475 | 54484 | TTGGA | 2 | atpB | YP_009380221.1 [atpB]\| |
| cH0000049 | PotentialSSR | HexaSSR-Cp | 55172 | 55183 | ATAATC | 2 |  |  |
| cH0000050 | PotentialSSR | HexaSSR-Cp | 55242 | 55253 | TCTTTT | 2 |  |  |
| cH0000051 | PotentialSSR | HexaSSR-Cp | 55280 | 55291 | AAAAAG | 2 |  |  |
| cP0000130 | PotentialSSR | PentaSSR-Cp | 55754 | 55763 | AAACA | 2 | trnV-UAC | (Intron)trnV-UAC [trnV-UAC]\| |
| c70000016 | ExtendedSSR | 7SSR-Cp | 56086 | 56099 | AATGAAT | 2 | trnV-UAC | (Intron)trnV-UAC [trnV-UAC]\| |
| cH0000052 | PotentialSSR | HexaSSR-Cp | 56332 | 56343 | CAATTG | 2 |  |  |
| cP0000131 | PotentialSSR | PentaSSR-Cp | 56393 | 56402 | ATTTG | 2 |  |  |
| cH0000053 | PotentialSSR | HexaSSR-Cp | 56417 | 56428 | TTTGAA | 2 |  |  |
| cP0000132 | PotentialSSR | PentaSSR-Cp | 56516 | 56525 | TTTTC | 2 |  |  |
| cP0000133 | PotentialSSR | PentaSSR-Cp | 58582 | 58591 | TATAT | 2 |  |  |
| cP0000134 | PotentialSSR | PentaSSR-Cp | 58638 | 58647 | CTATA | 2 |  |  |
| cTe0000005 | SSR | TetraSSR-Cp | 58665 | 58676 | TAAT | 3 |  |  |
| cP0000135 | PotentialSSR | PentaSSR-Cp | 58679 | 58688 | TAATA | 2 |  |  |
| cH0000054 | PotentialSSR | HexaSSR-Cp | 58720 | 58731 | TACAAT | 2 |  |  |
| cP0000136 | PotentialSSR | PentaSSR-Cp | 58850 | 58859 | AAAAT | 2 | psaI | YP_009380224.1 [psaI]\| |
| cP0000137 | PotentialSSR | PentaSSR-Cp | 58868 | 58877 | TCGTC | 2 |  |  |
| cP0000138 | PotentialSSR | PentaSSR-Cp | 59008 | 59017 | ACAAT | 2 |  |  |
| cP0000139 | PotentialSSR | PentaSSR-Cp | 59172 | 59181 | TTCAA | 2 |  |  |
| cP0000140 | PotentialSSR | PentaSSR-Cp | 59482 | 59491 | TCGAT | 2 | ycf4 | YP_009380225.1 [ycf4]\| |
| cP0000141 | PotentialSSR | PentaSSR-Cp | 59587 | 59596 | TAGAA | 2 | ycf4 | YP_009380225.1 [ycf4]\| |
| cH0000055 | PotentialSSR | HexaSSR-Cp | 59920 | 59931 | TACAAA | 2 |  |  |
| cP0000142 | PotentialSSR | PentaSSR-Cp | 59933 | 59942 | CAATT | 2 |  |  |
| cP0000143 | PotentialSSR | PentaSSR-Cp | 60073 | 60082 | AATTA | 2 |  |  |
| cP0000144 | PotentialSSR | PentaSSR-Cp | 61038 | 61047 | AAAAG | 2 |  |  |
| c70000017 | ExtendedSSR | 7SSR-Cp | 61102 | 61115 | ATAATCA | 2 |  |  |
| cP0000145 | PotentialSSR | PentaSSR-Cp | 61187 | 61196 | TTATT | 2 |  |  |
| cP0000146 | PotentialSSR | PentaSSR-Cp | 61685 | 61694 | AAAGA | 2 | petA | YP_009380227.1 [petA]\| |
| cP0000147 | PotentialSSR | PentaSSR-Cp | 61956 | 61965 | GAAAA | 2 | petA | YP_009380227.1 [petA]\| |
| cH0000056 | PotentialSSR | HexaSSR-Cp | 62276 | 62287 | ATCAAG | 2 |  |  |
| cH0000057 | PotentialSSR | HexaSSR-Cp | 62292 | 62303 | TAACAA | 2 |  |  |
| cP0000148 | PotentialSSR | PentaSSR-Cp | 62316 | 62325 | CAATT | 2 |  |  |
| cM0000014 | SSR | MonoSSR-Cp | 62350 | 62363 | A | 14 |  |  |
| c80000007 | ExtendedSSR | 8SSR-Cp | 62457 | 62472 | ACTTTTTT | 2 |  |  |
| cH0000058 | PotentialSSR | HexaSSR-Cp | 62664 | 62675 | ATTTTT | 2 |  |  |
| cH0000059 | PotentialSSR | HexaSSR-Cp | 62859 | 62870 | TCTTTC | 2 |  |  |
| cP0000149 | PotentialSSR | PentaSSR-Cp | 62873 | 62882 | TTAAC | 2 |  |  |
| c70000018 | ExtendedSSR | 7SSR-Cp | 63125 | 63138 | TTACTAC | 2 | psbJ | YP_009380228.1 [psbJ]\| |
| cP0000150 | PotentialSSR | PentaSSR-Cp | 63478 | 63487 | ATTCA | 2 | psbL | YP_009380229.1 [psbL]\| |
| cH0000060 | PotentialSSR | HexaSSR-Cp | 63529 | 63540 | ATTCGG | 2 |  |  |
| c80000008 | ExtendedSSR | 8SSR-Cp | 64009 | 64024 | ACGTAAAA | 2 |  |  |
| cH0000061 | PotentialSSR | HexaSSR-Cp | 64346 | 64357 | TATAGA | 2 |  |  |
| cH0000062 | PotentialSSR | HexaSSR-Cp | 64585 | 64596 | AGTCTT | 2 |  |  |
| cP0000151 | PotentialSSR | PentaSSR-Cp | 65141 | 65150 | CTGTA | 2 |  |  |
| cM0000015 | SSR | MonoSSR-Cp | 65256 | 65268 | T | 13 |  |  |
| cP0000152 | PotentialSSR | PentaSSR-Cp | 65460 | 65469 | GATTA | 2 |  |  |
| cP0000153 | PotentialSSR | PentaSSR-Cp | 65477 | 65486 | TTTAG | 2 |  |  |
| cH0000063 | PotentialSSR | HexaSSR-Cp | 65496 | 65507 | TTTCTA | 2 |  |  |
| cP0000154 | PotentialSSR | PentaSSR-Cp | 65594 | 65603 | GAACT | 2 | trnW-CCA | trnW-CCA [trnW-CCA]\| |
| cH0000064 | PotentialSSR | HexaSSR-Cp | 65670 | 65681 | CTATAT | 2 |  |  |
| cP0000155 | PotentialSSR | PentaSSR-Cp | 65877 | 65886 | TTCAA | 2 |  |  |
| cP0000156 | PotentialSSR | PentaSSR-Cp | 65916 | 65925 | CCTTG | 2 |  |  |
| cP0000157 | PotentialSSR | PentaSSR-Cp | 66052 | 66061 | GTAAA | 2 |  |  |
| cP0000158 | PotentialSSR | PentaSSR-Cp | 66164 | 66173 | CTTAG | 2 |  |  |
| cP0000159 | PotentialSSR | PentaSSR-Cp | 66520 | 66529 | TTAGT | 2 |  |  |
| cP0000160 | PotentialSSR | PentaSSR-Cp | 66573 | 66582 | GTTAA | 2 |  |  |
| c70000019 | ExtendedSSR | 7SSR-Cp | 66714 | 66727 | CGAATTG | 2 |  |  |
| cH0000065 | PotentialSSR | HexaSSR-Cp | 67077 | 67088 | AATTTT | 2 |  |  |
| cD0000007 | SSR | DiSSR-Cp | 67102 | 67111 | AT | 5 |  |  |
| cP0000161 | PotentialSSR | PentaSSR-Cp | 67171 | 67180 | ATATA | 2 |  |  |
| cP0000162 | PotentialSSR | PentaSSR-Cp | 67246 | 67255 | ATAGG | 2 |  |  |
| cP0000163 | PotentialSSR | PentaSSR-Cp | 67273 | 67282 | TAAAC | 2 |  |  |
| cTe0000006 | SSR | TetraSSR-Cp | 67757 | 67768 | TTTA | 3 |  |  |
| cP0000164 | PotentialSSR | PentaSSR-Cp | 68320 | 68329 | ATAAA | 2 |  |  |
| cH0000066 | PotentialSSR | HexaSSR-Cp | 68811 | 68822 | ATTTTC | 2 |  |  |
| cP0000165 | PotentialSSR | PentaSSR-Cp | 68929 | 68938 | CTATT | 2 |  |  |
| cH0000067 | PotentialSSR | HexaSSR-Cp | 68976 | 68987 | TTTTGT | 2 |  |  |
| cH0000068 | PotentialSSR | HexaSSR-Cp | 69296 | 69307 | TGGGCT | 2 | clpP | YP_009380239.1 [clpP]\| |
| cP0000166 | PotentialSSR | PentaSSR-Cp | 69526 | 69535 | ACACA | 2 | clpP | (Intron)YP_009380239.1 [clpP]\| |
| cP0000167 | PotentialSSR | PentaSSR-Cp | 69666 | 69675 | ATCGA | 2 | clpP | (Intron)YP_009380239.1 [clpP]\| |
| cH0000069 | PotentialSSR | HexaSSR-Cp | 69677 | 69688 | CAGATC | 2 | clpP | (Intron)YP_009380239.1 [clpP]\| |
| cP0000168 | PotentialSSR | PentaSSR-Cp | 69962 | 69971 | GAAAA | 2 | clpP | (Intron)YP_009380239.1 [clpP]\| |
| cH0000070 | PotentialSSR | HexaSSR-Cp | 70252 | 70263 | ACAAAT | 2 | clpP | YP_009380239.1 [clpP]\| |
| cP0000169 | PotentialSSR | PentaSSR-Cp | 70665 | 70674 | TATCA | 2 | clpP | (Intron)YP_009380239.1 [clpP]\| |
| cH0000071 | PotentialSSR | HexaSSR-Cp | 70705 | 70716 | TTCTTG | 2 | clpP | (Intron)YP_009380239.1 [clpP]\| |
| cH0000072 | PotentialSSR | HexaSSR-Cp | 70915 | 70926 | TTGAAA | 2 | clpP | (Intron)YP_009380239.1 [clpP]\| |
| cH0000073 | PotentialSSR | HexaSSR-Cp | 70982 | 70993 | ATTGGG | 2 | clpP | (Intron)YP_009380239.1 [clpP]\| |
| cP0000170 | PotentialSSR | PentaSSR-Cp | 71551 | 71560 | ATAGA | 2 |  |  |
| cH0000074 | PotentialSSR | HexaSSR-Cp | 71670 | 71681 | CATAGT | 2 |  |  |
| c70000021 | ExtendedSSR | 7SSR-Cp | 71820 | 71833 | CTGGTTG | 2 | psbB | YP_009380240.1 [psbB]\| |
| cH0000075 | PotentialSSR | HexaSSR-Cp | 72184 | 72195 | GTTTTG | 2 | psbB | YP_009380240.1 [psbB]\| |
| cP0000171 | PotentialSSR | PentaSSR-Cp | 73290 | 73299 | TTTGA | 2 |  |  |
| cP0000172 | PotentialSSR | PentaSSR-Cp | 73447 | 73456 | CTCTA | 2 | psbT | YP_009380241.1 [psbT]\| |
| cP0000173 | PotentialSSR | PentaSSR-Cp | 73511 | 73520 | AAATG | 2 | psbT | YP_009380241.1 [psbT]\| |
| cH0000076 | PotentialSSR | HexaSSR-Cp | 74042 | 74053 | ACAAAA | 2 |  |  |
| c90000002 | ExtendedSSR | 9SSR-Cp | 74091 | 74108 | CAATACAAA | 2 |  |  |
| cP0000174 | PotentialSSR | PentaSSR-Cp | 74394 | 74403 | TTTTC | 2 | petB | (Intron)YP_009380244.1 [petB]\| |
| cP0000175 | PotentialSSR | PentaSSR-Cp | 74512 | 74521 | AATAA | 2 | petB | (Intron)YP_009380244.1 [petB]\| |
| cP0000176 | PotentialSSR | PentaSSR-Cp | 74524 | 74533 | CCTAT | 2 | petB | (Intron)YP_009380244.1 [petB]\| |
| cH0000077 | PotentialSSR | HexaSSR-Cp | 74700 | 74711 | ATTATA | 2 | petB | (Intron)YP_009380244.1 [petB]\| |
| cH0000078 | PotentialSSR | HexaSSR-Cp | 74712 | 74723 | TACAAA | 2 | petB | (Intron)YP_009380244.1 [petB]\| |
| cP0000177 | PotentialSSR | PentaSSR-Cp | 75654 | 75663 | ATAGA | 2 | petB | YP_009380244.1 [petB]\| |
| cP0000178 | PotentialSSR | PentaSSR-Cp | 76023 | 76032 | TCTAT | 2 | petD | (Intron)YP_009380245.1 [petD]\| |
| cP0000179 | PotentialSSR | PentaSSR-Cp | 76099 | 76108 | ATAAT | 2 | petD | (Intron)YP_009380245.1 [petD]\| |
| cP0000180 | PotentialSSR | PentaSSR-Cp | 76113 | 76122 | ATTAT | 2 | petD | (Intron)YP_009380245.1 [petD]\| |
| cP0000181 | PotentialSSR | PentaSSR-Cp | 76136 | 76145 | TTATT | 2 | petD | (Intron)YP_009380245.1 [petD]\| |
| cP0000182 | PotentialSSR | PentaSSR-Cp | 76608 | 76617 | GAATC | 2 | petD | (Intron)YP_009380245.1 [petD]\| |
| cP0000183 | PotentialSSR | PentaSSR-Cp | 76621 | 76630 | AAGAA | 2 | petD | (Intron)YP_009380245.1 [petD]\| |
| cP0000184 | PotentialSSR | PentaSSR-Cp | 77200 | 77209 | ATTCA | 2 |  |  |
| cM0000016 | SSR | MonoSSR-Cp | 77427 | 77436 | T | 10 | rpoA | YP_009380246.1 [rpoA]\| |
| cH0000079 | PotentialSSR | HexaSSR-Cp | 77646 | 77657 | CATTTC | 2 | rpoA | YP_009380246.1 [rpoA]\| |
| cP0000185 | PotentialSSR | PentaSSR-Cp | 78076 | 78085 | TCGCA | 2 | rpoA | YP_009380246.1 [rpoA]\| |
| cP0000186 | PotentialSSR | PentaSSR-Cp | 78844 | 78853 | TAGTA | 2 |  |  |
| cM0000017 | SSR | MonoSSR-Cp | 78987 | 78997 | T | 11 |  |  |
| cH0000080 | PotentialSSR | HexaSSR-Cp | 79070 | 79081 | GTTGAA | 2 |  |  |
| cP0000187 | PotentialSSR | PentaSSR-Cp | 79902 | 79911 | TTAGT | 2 |  |  |
| cH0000081 | PotentialSSR | HexaSSR-Cp | 79973 | 79984 | TATTTT | 2 |  |  |
| cH0000082 | PotentialSSR | HexaSSR-Cp | 80980 | 80991 | AAAAAT | 2 | rpl16 | (Intron)YP_009380252.1 [rpl16]\| |
| cP0000188 | PotentialSSR | PentaSSR-Cp | 81007 | 81016 | TATTT | 2 | rpl16 | (Intron)YP_009380252.1 [rpl16]\| |
| c70000023 | ExtendedSSR | 7SSR-Cp | 81225 | 81238 | TTTTATA | 2 | rpl16 | (Intron)YP_009380252.1 [rpl16]\| |
| cP0000189 | PotentialSSR | PentaSSR-Cp | 81281 | 81290 | AAAAG | 2 | rpl16 | (Intron)YP_009380252.1 [rpl16]\| |
| cM0000018 | SSR | MonoSSR-Cp | 81567 | 81576 | T | 10 | rpl16 | (Intron)YP_009380252.1 [rpl16]\| |
| cM0000019 | SSR | MonoSSR-Cp | 81700 | 81711 | T | 12 | rpl16 | (Intron)YP_009380252.1 [rpl16]\| |
| cM0000020 | SSR | MonoSSR-Cp | 81760 | 81769 | T | 10 | rpl16 | (Intron)YP_009380252.1 [rpl16]\| |
| cH0000084 | PotentialSSR | HexaSSR-Cp | 81949 | 81960 | TTTTAA | 2 | rpl16 | (Intron)YP_009380252.1 [rpl16]\| |
| cP0000190 | PotentialSSR | PentaSSR-Cp | 82256 | 82265 | ACCCT | 2 | rps3 | YP_009380253.1 [rps3]\| |
| cP0000191 | PotentialSSR | PentaSSR-Cp | 82390 | 82399 | TACTC | 2 | rps3 | YP_009380253.1 [rps3]\| |
| cP0000192 | PotentialSSR | PentaSSR-Cp | 82493 | 82502 | CAATT | 2 | rps3 | YP_009380253.1 [rps3]\| |
| cP0000193 | PotentialSSR | PentaSSR-Cp | 82851 | 82860 | TTTAT | 2 | rpl22 | YP_009380254.1 [rpl22]\| |
| cP0000194 | PotentialSSR | PentaSSR-Cp | 83372 | 83381 | TTTCT | 2 |  |  |
| cH0000085 | PotentialSSR | HexaSSR-Cp | 83801 | 83812 | ATTTTC | 2 |  |  |
| cP0000195 | PotentialSSR | PentaSSR-Cp | 84216 | 84225 | TATGT | 2 | rpl2 | YP_009380256.1 [rpl2]\| |
| cP0000196 | PotentialSSR | PentaSSR-Cp | 84564 | 84573 | TGGAT | 2 | rpl2 | YP_009380256.1 [rpl2]\| |
| cP0000197 | PotentialSSR | PentaSSR-Cp | 84665 | 84674 | TTCTT | 2 |  |  |
| cP0000198 | PotentialSSR | PentaSSR-Cp | 84686 | 84695 | GAATA | 2 |  |  |
| cP0000199 | PotentialSSR | PentaSSR-Cp | 85044 | 85053 | TATGA | 2 |  |  |
| cP0000200 | PotentialSSR | PentaSSR-Cp | 85260 | 85269 | TGAAA | 2 |  |  |
| cH0000086 | PotentialSSR | HexaSSR-Cp | 85926 | 85937 | GAAAGA | 2 | ycf2 | YP_009380257.1 [ycf2]\| |
| cP0000201 | PotentialSSR | PentaSSR-Cp | 85978 | 85987 | GATCC | 2 | ycf2 | YP_009380257.1 [ycf2]\| |
| c90000003 | ExtendedSSR | 9SSR-Cp | 87258 | 87275 | GGAACATTT | 2 | ycf2 | YP_009380257.1 [ycf2]\| |
| cP0000202 | PotentialSSR | PentaSSR-Cp | 88105 | 88114 | CGATC | 2 | ycf2 | YP_009380257.1 [ycf2]\| |
| cP0000203 | PotentialSSR | PentaSSR-Cp | 88270 | 88279 | TTCAA | 2 | ycf2 | YP_009380257.1 [ycf2]\| |
| cH0000088 | PotentialSSR | HexaSSR-Cp | 89047 | 89058 | GGTCCC | 2 | ycf2 | YP_009380257.1 [ycf2]\| |
| cH0000089 | PotentialSSR | HexaSSR-Cp | 89223 | 89234 | AAGAAA | 2 | ycf2 | YP_009380257.1 [ycf2]\| |
| cP0000204 | PotentialSSR | PentaSSR-Cp | 89248 | 89257 | GATTG | 2 | ycf2 | YP_009380257.1 [ycf2]\| |
| cH0000090 | PotentialSSR | HexaSSR-Cp | 89889 | 89900 | GGAGCT | 2 | ycf2 | YP_009380257.1 [ycf2]\| |
| cP0000205 | PotentialSSR | PentaSSR-Cp | 90271 | 90280 | GAAAA | 2 | ycf2 | YP_009380257.1 [ycf2]\| |
| cH0000091 | PotentialSSR | HexaSSR-Cp | 90776 | 90787 | TAGAAG | 2 | ycf2 | YP_009380257.1 [ycf2]\| |
| cH0000092 | PotentialSSR | HexaSSR-Cp | 91376 | 91387 | CTATAT | 2 | ycf2 | YP_009380257.1 [ycf2]\| |
| cP0000206 | PotentialSSR | PentaSSR-Cp | 91870 | 91879 | AAGTT | 2 |  |  |
| cP0000207 | PotentialSSR | PentaSSR-Cp | 91898 | 91907 | TTGTT | 2 |  |  |
| cP0000208 | PotentialSSR | PentaSSR-Cp | 92142 | 92151 | GTTAC | 2 |  |  |
| cH0000093 | PotentialSSR | HexaSSR-Cp | 92317 | 92328 | ATTCCA | 2 |  |  |
| cP0000209 | PotentialSSR | PentaSSR-Cp | 92633 | 92642 | CTTAT | 2 |  |  |
| cP0000210 | PotentialSSR | PentaSSR-Cp | 92768 | 92777 | ATGGA | 2 |  |  |
| cH0000094 | PotentialSSR | HexaSSR-Cp | 93729 | 93740 | GCTGAA | 2 | ndhB | YP_009380258.1 [ndhB]\| |
| cH0000095 | PotentialSSR | HexaSSR-Cp | 93788 | 93799 | AGAGTC | 2 | ndhB | YP_009380258.1 [ndhB]\| |
| cP0000211 | PotentialSSR | PentaSSR-Cp | 94084 | 94093 | TGATT | 2 | ndhB | (Intron)YP_009380258.1 [ndhB]\| |
| cP0000212 | PotentialSSR | PentaSSR-Cp | 94284 | 94293 | AAAGA | 2 | ndhB | (Intron)YP_009380258.1 [ndhB]\| |
| cH0000096 | PotentialSSR | HexaSSR-Cp | 95351 | 95362 | TTCTTA | 2 |  |  |
| cP0000213 | PotentialSSR | PentaSSR-Cp | 95417 | 95426 | AGAAA | 2 |  |  |
| cP0000214 | PotentialSSR | PentaSSR-Cp | 95521 | 95530 | CTGTT | 2 |  |  |
| cH0000097 | PotentialSSR | HexaSSR-Cp | 96268 | 96279 | TCCATA | 2 |  |  |
| cP0000215 | PotentialSSR | PentaSSR-Cp | 96456 | 96465 | CGAAT | 2 |  |  |
| cH0000098 | PotentialSSR | HexaSSR-Cp | 97045 | 97056 | TTGATT | 2 |  |  |
| cH0000099 | PotentialSSR | HexaSSR-Cp | 97092 | 97103 | TTCCTC | 2 |  |  |
| cH0000100 | PotentialSSR | HexaSSR-Cp | 97104 | 97115 | TATCCC | 2 |  |  |
| cP0000216 | PotentialSSR | PentaSSR-Cp | 97350 | 97359 | TGTTG | 2 |  |  |
| cP0000217 | PotentialSSR | PentaSSR-Cp | 97448 | 97457 | TATTA | 2 |  |  |
| cP0000218 | PotentialSSR | PentaSSR-Cp | 97467 | 97476 | ATTAG | 2 |  |  |
| cP0000219 | PotentialSSR | PentaSSR-Cp | 97649 | 97658 | GCAAT | 2 |  |  |
| cH0000101 | PotentialSSR | HexaSSR-Cp | 97769 | 97780 | TATTAC | 2 |  |  |
| cH0000102 | PotentialSSR | HexaSSR-Cp | 97916 | 97927 | AATGGA | 2 |  |  |
| cM0000021 | SSR | MonoSSR-Cp | 98026 | 98037 | T | 12 |  |  |
| cP0000220 | PotentialSSR | PentaSSR-Cp | 98277 | 98286 | CAAGA | 2 |  |  |
| cP0000221 | PotentialSSR | PentaSSR-Cp | 98360 | 98369 | AGGGA | 2 |  |  |
| cH0000103 | PotentialSSR | HexaSSR-Cp | 98484 | 98495 | GAATGA | 2 |  |  |
| cH0000104 | PotentialSSR | HexaSSR-Cp | 99357 | 99368 | GACACT | 2 | rrn16 | rrn16 [rrn16]\| |
| cH0000105 | PotentialSSR | HexaSSR-Cp | 100814 | 100825 | AATGGA | 2 | trnI-GAU | (Intron)trnI-GAU [trnI-GAU]\| |
| cH0000106 | PotentialSSR | HexaSSR-Cp | 101570 | 101581 | AAGAAT | 2 |  |  |
| cP0000222 | PotentialSSR | PentaSSR-Cp | 101801 | 101810 | ACAAA | 2 | trnA-UGC | (Intron)trnA-UGC [trnA-UGC]\| |
| cP0000223 | PotentialSSR | PentaSSR-Cp | 102120 | 102129 | TTCAA | 2 | trnA-UGC | (Intron)trnA-UGC [trnA-UGC]\| |
| c80000010 | ExtendedSSR | 8SSR-Cp | 102344 | 102359 | TTTTGAGA | 2 | trnA-UGC | (Intron)trnA-UGC [trnA-UGC]\| |
| cD0000008 | SSR | DiSSR-Cp | 103919 | 103928 | CG | 5 | rrn23 | rrn23 [rrn23]\| |
| cH0000107 | PotentialSSR | HexaSSR-Cp | 103942 | 103953 | GAAGCG | 2 | rrn23 | rrn23 [rrn23]\| |
| cTe0000007 | SSR | TetraSSR-Cp | 104336 | 104347 | AGGT | 3 | rrn23 | rrn23 [rrn23]\| |
| cP0000224 | PotentialSSR | PentaSSR-Cp | 105429 | 105438 | GCGGA | 2 | rrn23 | rrn23 [rrn23]\| |
| cP0000225 | PotentialSSR | PentaSSR-Cp | 105727 | 105736 | ATCCA | 2 |  |  |
| c70000024 | ExtendedSSR | 7SSR-Cp | 105799 | 105812 | AAAAACC | 2 |  |  |
| cH0000108 | PotentialSSR | HexaSSR-Cp | 105829 | 105840 | TCTATC | 2 |  |  |
| cH0000109 | PotentialSSR | HexaSSR-Cp | 106206 | 106217 | TTCTTA | 2 |  |  |
| cP0000226 | PotentialSSR | PentaSSR-Cp | 106503 | 106512 | AGTGG | 2 |  |  |
| cH0000110 | PotentialSSR | HexaSSR-Cp | 106643 | 106654 | CAAGTA | 2 |  |  |
| cP0000227 | PotentialSSR | PentaSSR-Cp | 106659 | 106668 | TAGCA | 2 |  |  |
| cP0000228 | PotentialSSR | PentaSSR-Cp | 106683 | 106692 | GTCAT | 2 |  |  |
| c70000025 | ExtendedSSR | 7SSR-Cp | 106696 | 106709 | TATGTTT | 2 |  |  |
| cP0000229 | PotentialSSR | PentaSSR-Cp | 106868 | 106877 | CAGAA | 2 |  |  |
| c70000026 | ExtendedSSR | 7SSR-Cp | 106974 | 106987 | AAGAATG | 2 |  |  |
| c90000004 | ExtendedSSR | 9SSR-Cp | 107535 | 107552 | GAAGAAGGA | 2 | ycf1 | YP_009380260.1 [ycf1]\| |
| cH0000112 | PotentialSSR | HexaSSR-Cp | 108157 | 108168 | TAGAAA | 2 | ycf1 | YP_009380260.1 [ycf1]\| |
| cH0000113 | PotentialSSR | HexaSSR-Cp | 108224 | 108235 | TCCTTC | 2 | ycf1 | YP_009380260.1 [ycf1]\| |
| cP0000230 | PotentialSSR | PentaSSR-Cp | 108247 | 108256 | AAGAA | 2 | ycf1 | YP_009380260.1 [ycf1]\| |
| cH0000114 | PotentialSSR | HexaSSR-Cp | 108266 | 108277 | CAAAAT | 2 | ycf1 | YP_009380260.1 [ycf1]\| |
| cP0000231 | PotentialSSR | PentaSSR-Cp | 108313 | 108322 | ACAAA | 2 | ycf1 | YP_009380260.1 [ycf1]\| |
| cP0000232 | PotentialSSR | PentaSSR-Cp | 108538 | 108547 | GAAAT | 2 | ycf1 | YP_009380260.1 [ycf1]\| |
| cM0000022 | SSR | MonoSSR-Cp | 108724 | 108733 | A | 10 | ycf1 | YP_009380260.1 [ycf1]\| |
| cP0000233 | PotentialSSR | PentaSSR-Cp | 108838 | 108847 | AAAAT | 2 | ycf1,ndhF | YP_009380260.1 [ycf1]\|YP_009380261.1 [ndhF]\| |
| cH0000115 | PotentialSSR | HexaSSR-Cp | 109996 | 110007 | AGATCC | 2 | ndhF | YP_009380261.1 [ndhF]\| |
| c70000027 | ExtendedSSR | 7SSR-Cp | 110463 | 110476 | CTCGAAA | 2 | ndhF | YP_009380261.1 [ndhF]\| |
| cP0000234 | PotentialSSR | PentaSSR-Cp | 110997 | 111006 | AAAAG | 2 | ndhF | YP_009380261.1 [ndhF]\| |
| cP0000235 | PotentialSSR | PentaSSR-Cp | 111089 | 111098 | TAGAA | 2 |  |  |
| c70000028 | ExtendedSSR | 7SSR-Cp | 111262 | 111275 | TTAAAAC | 2 |  |  |
| cP0000236 | PotentialSSR | PentaSSR-Cp | 111518 | 111527 | TTACT | 2 |  |  |
| cM0000023 | SSR | MonoSSR-Cp | 111916 | 111925 | A | 10 |  |  |
| cM0000024 | SSR | MonoSSR-Cp | 112233 | 112242 | A | 10 |  |  |
| cP0000237 | PotentialSSR | PentaSSR-Cp | 112481 | 112490 | TTTTA | 2 |  |  |
| c70000029 | ExtendedSSR | 7SSR-Cp | 112501 | 112514 | TAAAAGA | 2 |  |  |
| cM0000025 | SSR | MonoSSR-Cp | 112552 | 112561 | A | 10 |  |  |
| cP0000238 | PotentialSSR | PentaSSR-Cp | 112848 | 112857 | AGAAA | 2 |  |  |
| cP0000239 | PotentialSSR | PentaSSR-Cp | 112977 | 112986 | GAAAA | 2 |  |  |
| cH0000116 | PotentialSSR | HexaSSR-Cp | 113163 | 113174 | TATGAA | 2 |  |  |
| cH0000117 | PotentialSSR | HexaSSR-Cp | 113423 | 113434 | AATGAA | 2 |  |  |
| cM0000026 | SSR | MonoSSR-Cp | 113482 | 113491 | T | 10 |  |  |
| cP0000240 | PotentialSSR | PentaSSR-Cp | 114609 | 114618 | TTTTG | 2 |  |  |
| cP0000241 | PotentialSSR | PentaSSR-Cp | 114647 | 114656 | TTTCA | 2 |  |  |
| cH0000118 | PotentialSSR | HexaSSR-Cp | 114745 | 114756 | AATACC | 2 | ndhD | YP_009380264.1 [ndhD]\| |
| cM0000027 | SSR | MonoSSR-Cp | 114811 | 114820 | A | 10 | ndhD | YP_009380264.1 [ndhD]\| |
| cH0000119 | PotentialSSR | HexaSSR-Cp | 115741 | 115752 | TAATTC | 2 | ndhD | YP_009380264.1 [ndhD]\| |
| cH0000120 | PotentialSSR | HexaSSR-Cp | 116593 | 116604 | TCTAGT | 2 |  |  |
| cP0000242 | PotentialSSR | PentaSSR-Cp | 117228 | 117237 | AAGTT | 2 |  |  |
| cP0000243 | PotentialSSR | PentaSSR-Cp | 117528 | 117537 | ATACC | 2 | ndhG | YP_009380267.1 [ndhG]\| |
| c70000030 | ExtendedSSR | 7SSR-Cp | 117740 | 117753 | TAGAATA | 2 | ndhG | YP_009380267.1 [ndhG]\| |
| cP0000244 | PotentialSSR | PentaSSR-Cp | 118015 | 118024 | TAAAG | 2 |  |  |
| cP0000245 | PotentialSSR | PentaSSR-Cp | 118180 | 118189 | TTTAA | 2 |  |  |
| cP0000246 | PotentialSSR | PentaSSR-Cp | 118301 | 118310 | TAATT | 2 | ndhI | YP_009380268.1 [ndhI]\| |
| cH0000121 | PotentialSSR | HexaSSR-Cp | 118943 | 118954 | GAACAA | 2 | ndhA | YP_009380269.1 [ndhA]\| |
| cP0000247 | PotentialSSR | PentaSSR-Cp | 118967 | 118976 | TAATG | 2 | ndhA | YP_009380269.1 [ndhA]\| |
| cP0000248 | PotentialSSR | PentaSSR-Cp | 119120 | 119129 | ATAAA | 2 | ndhA | YP_009380269.1 [ndhA]\| |
| cP0000249 | PotentialSSR | PentaSSR-Cp | 119348 | 119357 | AAGAT | 2 | ndhA | (Intron)YP_009380269.1 [ndhA]\| |
| cP0000250 | PotentialSSR | PentaSSR-Cp | 119536 | 119545 | CTATA | 2 | ndhA | (Intron)YP_009380269.1 [ndhA]\| |
| c70000031 | ExtendedSSR | 7SSR-Cp | 119658 | 119671 | TATCAAT | 2 | ndhA | (Intron)YP_009380269.1 [ndhA]\| |
| cM0000028 | SSR | MonoSSR-Cp | 119787 | 119796 | T | 10 | ndhA | (Intron)YP_009380269.1 [ndhA]\| |
| cH0000122 | PotentialSSR | HexaSSR-Cp | 119801 | 119812 | CTATTA | 2 | ndhA | (Intron)YP_009380269.1 [ndhA]\| |
| cP0000251 | PotentialSSR | PentaSSR-Cp | 121252 | 121261 | CATTC | 2 | ndhH | YP_009380270.1 [ndhH]\| |
| cH0000123 | PotentialSSR | HexaSSR-Cp | 122261 | 122272 | ATAATT | 2 |  |  |
| cP0000252 | PotentialSSR | PentaSSR-Cp | 122666 | 122675 | TTTAT | 2 |  |  |
| cH0000124 | PotentialSSR | HexaSSR-Cp | 122951 | 122962 | AATTTT | 2 | ycf1 | YP_009380272.1 [ycf1]\| |
| cP0000253 | PotentialSSR | PentaSSR-Cp | 123391 | 123400 | TTCTT | 2 | ycf1 | YP_009380272.1 [ycf1]\| |
| cM0000029 | SSR | MonoSSR-Cp | 123970 | 123981 | T | 12 | ycf1 | YP_009380272.1 [ycf1]\| |
| cH0000125 | PotentialSSR | HexaSSR-Cp | 124337 | 124348 | CTATAT | 2 | ycf1 | YP_009380272.1 [ycf1]\| |
| cH0000126 | PotentialSSR | HexaSSR-Cp | 124435 | 124446 | CAATAA | 2 | ycf1 | YP_009380272.1 [ycf1]\| |
| cTe0000008 | SSR | TetraSSR-Cp | 124469 | 124480 | TAAT | 3 | ycf1 | YP_009380272.1 [ycf1]\| |
| cM0000030 | SSR | MonoSSR-Cp | 125515 | 125525 | A | 11 | ycf1 | YP_009380272.1 [ycf1]\| |
| cP0000254 | PotentialSSR | PentaSSR-Cp | 125680 | 125689 | AAAAC | 2 | ycf1 | YP_009380272.1 [ycf1]\| |
| cP0000255 | PotentialSSR | PentaSSR-Cp | 127016 | 127025 | ATTTT | 2 | ycf1 | YP_009380272.1 [ycf1]\| |
| cM0000031 | SSR | MonoSSR-Cp | 127130 | 127139 | T | 10 | ycf1 | YP_009380272.1 [ycf1]\| |
| cP0000256 | PotentialSSR | PentaSSR-Cp | 127315 | 127324 | CATTT | 2 | ycf1 | YP_009380272.1 [ycf1]\| |
| cP0000257 | PotentialSSR | PentaSSR-Cp | 127541 | 127550 | TTTGT | 2 | ycf1 | YP_009380272.1 [ycf1]\| |
| cH0000127 | PotentialSSR | HexaSSR-Cp | 127586 | 127597 | ATTTTG | 2 | ycf1 | YP_009380272.1 [ycf1]\| |
| cP0000258 | PotentialSSR | PentaSSR-Cp | 127607 | 127616 | TTCTT | 2 | ycf1 | YP_009380272.1 [ycf1]\| |
| cH0000128 | PotentialSSR | HexaSSR-Cp | 127628 | 127639 | GAAGGA | 2 | ycf1 | YP_009380272.1 [ycf1]\| |
| cH0000129 | PotentialSSR | HexaSSR-Cp | 127695 | 127706 | TTTCTA | 2 | ycf1 | YP_009380272.1 [ycf1]\| |
| c90000005 | ExtendedSSR | 9SSR-Cp | 128311 | 128328 | TCCTTCTTC | 2 | ycf1 | YP_009380272.1 [ycf1]\| |
| c70000032 | ExtendedSSR | 7SSR-Cp | 128874 | 128887 | TTCATTC | 2 |  |  |
| cP0000259 | PotentialSSR | PentaSSR-Cp | 128986 | 128995 | TTCTG | 2 |  |  |
| c70000033 | ExtendedSSR | 7SSR-Cp | 129154 | 129167 | AAACATA | 2 |  |  |
| cP0000260 | PotentialSSR | PentaSSR-Cp | 129171 | 129180 | ATGAC | 2 |  |  |
| cP0000261 | PotentialSSR | PentaSSR-Cp | 129195 | 129204 | TGCTA | 2 |  |  |
| cH0000131 | PotentialSSR | HexaSSR-Cp | 129209 | 129220 | TACTTG | 2 |  |  |
| cP0000262 | PotentialSSR | PentaSSR-Cp | 129350 | 129359 | TCCAC | 2 |  |  |
| cH0000132 | PotentialSSR | HexaSSR-Cp | 129645 | 129656 | ATAAGA | 2 |  |  |
| cH0000133 | PotentialSSR | HexaSSR-Cp | 130023 | 130034 | GATAGA | 2 |  |  |
| c70000034 | ExtendedSSR | 7SSR-Cp | 130051 | 130064 | GGTTTTT | 2 |  |  |
| cP0000263 | PotentialSSR | PentaSSR-Cp | 130127 | 130136 | TGGAT | 2 |  |  |
| cP0000264 | PotentialSSR | PentaSSR-Cp | 130425 | 130434 | TCCGC | 2 | rrn23 | rrn23 [rrn23]\| |
| cTe0000009 | SSR | TetraSSR-Cp | 131514 | 131525 | CTAC | 3 | rrn23 | rrn23 [rrn23]\| |
| cH0000134 | PotentialSSR | HexaSSR-Cp | 131910 | 131921 | CGCTTC | 2 | rrn23 | rrn23 [rrn23]\| |
| cD0000009 | SSR | DiSSR-Cp | 131935 | 131944 | CG | 5 | rrn23 | rrn23 [rrn23]\| |
| c80000011 | ExtendedSSR | 8SSR-Cp | 133504 | 133519 | TCTCAAAA | 2 | trnA-UGC | (Intron)trnA-UGC [trnA-UGC]\| |
| cP0000265 | PotentialSSR | PentaSSR-Cp | 133733 | 133742 | ATTGA | 2 | trnA-UGC | (Intron)trnA-UGC [trnA-UGC]\| |
| cP0000266 | PotentialSSR | PentaSSR-Cp | 134053 | 134062 | TTTGT | 2 | trnA-UGC | (Intron)trnA-UGC [trnA-UGC]\| |
| cH0000135 | PotentialSSR | HexaSSR-Cp | 134282 | 134293 | ATTCTT | 2 |  |  |
| cH0000136 | PotentialSSR | HexaSSR-Cp | 135038 | 135049 | TCCATT | 2 | trnI-GAU | (Intron)trnI-GAU [trnI-GAU]\| |
| cH0000137 | PotentialSSR | HexaSSR-Cp | 136493 | 136504 | TCAGTG | 2 | rrn16 | rrn16 [rrn16]\| |
| cH0000138 | PotentialSSR | HexaSSR-Cp | 137368 | 137379 | TCATTC | 2 |  |  |
| cP0000267 | PotentialSSR | PentaSSR-Cp | 137494 | 137503 | TCCCT | 2 | trnV-GAC | trnV-GAC [trnV-GAC]\| |
| cP0000268 | PotentialSSR | PentaSSR-Cp | 137577 | 137586 | TCTTG | 2 |  |  |
| cM0000032 | SSR | MonoSSR-Cp | 137826 | 137837 | A | 12 |  |  |
| cH0000139 | PotentialSSR | HexaSSR-Cp | 137934 | 137945 | TTTCCA | 2 |  |  |
| cH0000140 | PotentialSSR | HexaSSR-Cp | 138080 | 138091 | ATAGTA | 2 |  |  |
| cP0000269 | PotentialSSR | PentaSSR-Cp | 138204 | 138213 | CATTG | 2 |  |  |
| cP0000270 | PotentialSSR | PentaSSR-Cp | 138387 | 138396 | CTAAT | 2 |  |  |
| cP0000271 | PotentialSSR | PentaSSR-Cp | 138406 | 138415 | TAATA | 2 |  |  |
| cP0000272 | PotentialSSR | PentaSSR-Cp | 138504 | 138513 | CAACA | 2 |  |  |
| cH0000141 | PotentialSSR | HexaSSR-Cp | 138748 | 138759 | GGGATA | 2 |  |  |
| cH0000142 | PotentialSSR | HexaSSR-Cp | 138760 | 138771 | GAGGAA | 2 |  |  |
| cH0000143 | PotentialSSR | HexaSSR-Cp | 138805 | 138816 | AAAATC | 2 |  |  |
| cP0000273 | PotentialSSR | PentaSSR-Cp | 139397 | 139406 | GATTC | 2 |  |  |
| cH0000144 | PotentialSSR | HexaSSR-Cp | 139584 | 139595 | TATGGA | 2 |  |  |
| cP0000274 | PotentialSSR | PentaSSR-Cp | 140332 | 140341 | GAACA | 2 |  |  |
| cP0000275 | PotentialSSR | PentaSSR-Cp | 140437 | 140446 | TTTCT | 2 |  |  |
| cH0000145 | PotentialSSR | HexaSSR-Cp | 140501 | 140512 | TAAGAA | 2 |  |  |
| cP0000276 | PotentialSSR | PentaSSR-Cp | 141570 | 141579 | TCTTT | 2 | ndhB | (Intron)YP_009380274.1 [ndhB]\| |
| cP0000277 | PotentialSSR | PentaSSR-Cp | 141770 | 141779 | AATCA | 2 | ndhB | (Intron)YP_009380274.1 [ndhB]\| |
| cH0000146 | PotentialSSR | HexaSSR-Cp | 142064 | 142075 | GACTCT | 2 | ndhB | YP_009380274.1 [ndhB]\| |
| cH0000147 | PotentialSSR | HexaSSR-Cp | 142121 | 142132 | GCTTCA | 2 | ndhB | YP_009380274.1 [ndhB]\| |
| cP0000278 | PotentialSSR | PentaSSR-Cp | 143085 | 143094 | TTCCA | 2 |  |  |
| cP0000279 | PotentialSSR | PentaSSR-Cp | 143221 | 143230 | ATAAG | 2 |  |  |
| cH0000148 | PotentialSSR | HexaSSR-Cp | 143534 | 143545 | TTGGAA | 2 |  |  |
| cP0000280 | PotentialSSR | PentaSSR-Cp | 143711 | 143720 | CGTAA | 2 |  |  |
| cP0000281 | PotentialSSR | PentaSSR-Cp | 143956 | 143965 | AACAA | 2 |  |  |
| cP0000282 | PotentialSSR | PentaSSR-Cp | 143984 | 143993 | AACTT | 2 |  |  |
| cH0000149 | PotentialSSR | HexaSSR-Cp | 144476 | 144487 | ATATAG | 2 | ycf2 | YP_009380275.1 [ycf2]\| |
| cH0000150 | PotentialSSR | HexaSSR-Cp | 145076 | 145087 | CTTCTA | 2 | ycf2 | YP_009380275.1 [ycf2]\| |
| cP0000283 | PotentialSSR | PentaSSR-Cp | 145583 | 145592 | TTTTC | 2 | ycf2 | YP_009380275.1 [ycf2]\| |
| cH0000151 | PotentialSSR | HexaSSR-Cp | 145962 | 145973 | CAGCTC | 2 | ycf2 | YP_009380275.1 [ycf2]\| |
| cP0000284 | PotentialSSR | PentaSSR-Cp | 146606 | 146615 | CAATC | 2 | ycf2 | YP_009380275.1 [ycf2]\| |
| cH0000152 | PotentialSSR | HexaSSR-Cp | 146626 | 146637 | CTTTTT | 2 | ycf2 | YP_009380275.1 [ycf2]\| |
| cH0000153 | PotentialSSR | HexaSSR-Cp | 146805 | 146816 | GGGACC | 2 | ycf2 | YP_009380275.1 [ycf2]\| |
| cP0000285 | PotentialSSR | PentaSSR-Cp | 147584 | 147593 | TTGAA | 2 | ycf2 | YP_009380275.1 [ycf2]\| |
| cP0000286 | PotentialSSR | PentaSSR-Cp | 147749 | 147758 | GATCG | 2 | ycf2 | YP_009380275.1 [ycf2]\| |
| c90000006 | ExtendedSSR | 9SSR-Cp | 148588 | 148605 | AAATGTTCC | 2 | ycf2 | YP_009380275.1 [ycf2]\| |
| cP0000287 | PotentialSSR | PentaSSR-Cp | 149875 | 149884 | CGGAT | 2 | ycf2 | YP_009380275.1 [ycf2]\| |
| cH0000155 | PotentialSSR | HexaSSR-Cp | 149926 | 149937 | TCTTTC | 2 | ycf2 | YP_009380275.1 [ycf2]\| |
| cP0000288 | PotentialSSR | PentaSSR-Cp | 150593 | 150602 | ATTTC | 2 |  |  |
| cP0000289 | PotentialSSR | PentaSSR-Cp | 150810 | 150819 | TCATA | 2 |  |  |
| cP0000290 | PotentialSSR | PentaSSR-Cp | 151168 | 151177 | TATTC | 2 |  |  |
| cP0000291 | PotentialSSR | PentaSSR-Cp | 151188 | 151197 | AAAGA | 2 |  |  |
| cP0000292 | PotentialSSR | PentaSSR-Cp | 151289 | 151298 | AATCC | 2 | rpl2 | YP_009380276.1 [rpl2]\| |
| cP0000293 | PotentialSSR | PentaSSR-Cp | 151638 | 151647 | ACATA | 2 | rpl2 | YP_009380276.1 [rpl2]\| |
| cH0000156 | PotentialSSR | HexaSSR-Cp | 152051 | 152062 | GAAAAT | 2 |  |  |

**Supplementary Table 7. List of SSRs identified in NC_034950 of *C. album***

| **Name** | **SSR type** | **Type** | **Cooridnation** | | **Unit sequence** | **Repeat number** | **Genes** | **Annotation** |
| --- | --- | --- | --- | --- | --- | --- | --- | --- |
| cH0000001 | PotentialSSR | HexaSSR-Cp | 113 | 124 | GTAAAG | 2 |  |  |
| cH0000002 | PotentialSSR | HexaSSR-Cp | 1240 | 1251 | GCTTTC | 2 | psbA | YP_009380194.1 [psbA]\| |
| cP0000001 | PotentialSSR | PentaSSR-Cp | 1292 | 1301 | ATTTA | 2 |  |  |
| c70000001 | ExtendedSSR | HeptaSSR-Cp | 1454 | 1467 | AGAAAAT | 2 |  |  |
| c70000002 | ExtendedSSR | HeptaSSR-Cp | 1696 | 1709 | AGTAGAA | 2 | trnK-UUU | (Intron)trnK-UUU [trnK-UUU]\| |
| cH0000003 | PotentialSSR | HexaSSR-Cp | 1748 | 1759 | AATTTC | 2 | trnK-UUU | (Intron)trnK-UUU [trnK-UUU]\| |
| cH0000004 | PotentialSSR | HexaSSR-Cp | 2949 | 2960 | TTTTTC | 2 | matK,trnK-UUU | YP_009380195.1 [matK]\|(Intron)trnK-UUU [trnK-UUU]\| |
| cP0000002 | PotentialSSR | PentaSSR-Cp | 4093 | 4102 | TATGT | 2 |  |  |
| cP0000003 | PotentialSSR | PentaSSR-Cp | 4124 | 4133 | AATGG | 2 |  |  |
| cP0000004 | PotentialSSR | PentaSSR-Cp | 4202 | 4211 | CAGAT | 2 |  |  |
| cP0000005 | PotentialSSR | PentaSSR-Cp | 4355 | 4364 | CATTT | 2 |  |  |
| cH0000005 | PotentialSSR | HexaSSR-Cp | 4442 | 4453 | AATATT | 2 |  |  |
| cTe0000001 | SSR | TetraSSR-Cp | 4464 | 4475 | TTTA | 3 |  |  |
| cP0000006 | PotentialSSR | PentaSSR-Cp | 4487 | 4496 | ATTTA | 2 |  |  |
| cP0000007 | PotentialSSR | PentaSSR-Cp | 4572 | 4581 | ATATA | 2 |  |  |
| cT0000003 | SSR | TriSSR-Cp | 4613 | 4624 | ATA | 4 |  |  |
| cP0000008 | PotentialSSR | PentaSSR-Cp | 4970 | 4979 | TAGAT | 2 | rps16 | (Intron)YP_009380196.1 [rps16]\| |
| cP0000009 | PotentialSSR | PentaSSR-Cp | 5204 | 5213 | CATTT | 2 | rps16 | (Intron)YP_009380196.1 [rps16]\| |
| cH0000007 | PotentialSSR | HexaSSR-Cp | 5274 | 5285 | ATCCAA | 2 | rps16 | (Intron)YP_009380196.1 [rps16]\| |
| c70000004 | ExtendedSSR | HeptaSSR-Cp | 5294 | 5307 | ACAATTA | 2 | rps16 | (Intron)YP_009380196.1 [rps16]\| |
| cM0000001 | SSR | MonoSSR-Cp | 5341 | 5351 | T | 11 | rps16 | (Intron)YP_009380196.1 [rps16]\| |
| cP0000010 | PotentialSSR | PentaSSR-Cp | 5441 | 5450 | AGAAT | 2 | rps16 | (Intron)YP_009380196.1 [rps16]\| |
| cP0000011 | PotentialSSR | PentaSSR-Cp | 5997 | 6006 | AATGC | 2 |  |  |
| cP0000012 | SSR | PentaSSR-Cp | 6080 | 6094 | TTAAT | 3 |  |  |
| cP0000013 | PotentialSSR | PentaSSR-Cp | 6190 | 6199 | TATAT | 2 |  |  |
| cH0000009 | PotentialSSR | HexaSSR-Cp | 6467 | 6478 | GCTCTG | 2 |  |  |
| cP0000014 | PotentialSSR | PentaSSR-Cp | 6555 | 6564 | TTCTA | 2 |  |  |
| cP0000015 | PotentialSSR | PentaSSR-Cp | 6629 | 6638 | GATTC | 2 |  |  |
| cM0000002 | SSR | MonoSSR-Cp | 6787 | 6797 | A | 11 |  |  |
| cM0000003 | SSR | MonoSSR-Cp | 6853 | 6862 | A | 10 |  |  |
| c80000001 | ExtendedSSR | OctaSSR-Cp | 7100 | 7115 | ATTCTAAT | 2 |  |  |
| cH0000010 | PotentialSSR | HexaSSR-Cp | 7345 | 7356 | TTACAA | 2 |  |  |
| cP0000016 | PotentialSSR | PentaSSR-Cp | 7398 | 7407 | TCAAA | 2 |  |  |
| cP0000017 | PotentialSSR | PentaSSR-Cp | 7608 | 7617 | TTCTT | 2 |  |  |
| cP0000018 | PotentialSSR | PentaSSR-Cp | 7640 | 7649 | ATTCT | 2 |  |  |
| cM0000004 | SSR | MonoSSR-Cp | 7729 | 7739 | A | 11 |  |  |
| cP0000019 | PotentialSSR | PentaSSR-Cp | 7895 | 7904 | GAAAA | 2 |  |  |
| cP0000020 | PotentialSSR | PentaSSR-Cp | 7985 | 7994 | ATATA | 2 |  |  |
| cH0000012 | PotentialSSR | HexaSSR-Cp | 8065 | 8076 | AGATAA | 2 |  |  |
| cP0000021 | PotentialSSR | PentaSSR-Cp | 8133 | 8142 | TAAAA | 2 |  |  |
| cP0000022 | PotentialSSR | PentaSSR-Cp | 8209 | 8218 | AAAGA | 2 |  |  |
| cP0000023 | PotentialSSR | PentaSSR-Cp | 8264 | 8273 | GGCCT | 2 |  |  |
| cH0000013 | PotentialSSR | HexaSSR-Cp | 8342 | 8353 | TTTGAT | 2 |  |  |
| cP0000024 | PotentialSSR | PentaSSR-Cp | 8858 | 8867 | AATTG | 2 | trnG-UCC | (Intron)trnG-UCC [trnG-UCC]\| |
| cP0000025 | PotentialSSR | PentaSSR-Cp | 9223 | 9232 | TTTTG | 2 | trnG-UCC | (Intron)trnG-UCC [trnG-UCC]\| |
| cP0000026 | PotentialSSR | PentaSSR-Cp | 9248 | 9257 | CTTAA | 2 | trnG-UCC | (Intron)trnG-UCC [trnG-UCC]\| |
| cP0000027 | PotentialSSR | PentaSSR-Cp | 9281 | 9290 | TTTTC | 2 | trnG-UCC | (Intron)trnG-UCC [trnG-UCC]\| |
| cP0000028 | PotentialSSR | PentaSSR-Cp | 9479 | 9488 | TCTCA | 2 |  |  |
| cP0000029 | PotentialSSR | PentaSSR-Cp | 9501 | 9510 | CAAAA | 2 |  |  |
| cM0000005 | SSR | MonoSSR-Cp | 9527 | 9537 | A | 11 |  |  |
| cH0000016 | PotentialSSR | HexaSSR-Cp | 10443 | 10454 | GCTTGT | 2 | atpA | YP_009380199.1 [atpA]\| |
| cH0000017 | PotentialSSR | HexaSSR-Cp | 10846 | 10857 | CGGGAG | 2 | atpA | YP_009380199.1 [atpA]\| |
| c80000002 | ExtendedSSR | OctaSSR-Cp | 11967 | 11982 | AAAAATAG | 2 | atpF | (Intron)YP_009380200.1 [atpF]\| |
| c70000008 | ExtendedSSR | HeptaSSR-Cp | 12210 | 12223 | TCGGTAT | 2 | atpF | (Intron)YP_009380200.1 [atpF]\| |
| cT0000010 | SSR | TriSSR-Cp | 12316 | 12327 | ATT | 4 | atpF | (Intron)YP_009380200.1 [atpF]\| |
| cTe0000002 | SSR | TetraSSR-Cp | 12881 | 12892 | GGAA | 3 |  |  |
| cP0000030 | PotentialSSR | PentaSSR-Cp | 13029 | 13038 | AGAAA | 2 |  |  |
| cP0000031 | PotentialSSR | PentaSSR-Cp | 13054 | 13063 | TTTCT | 2 |  |  |
| cD0000001 | SSR | DiSSR-Cp | 13325 | 13334 | CA | 5 |  |  |
| cP0000032 | PotentialSSR | PentaSSR-Cp | 13816 | 13825 | AAATA | 2 |  |  |
| cD0000002 | SSR | DiSSR-Cp | 13883 | 13892 | AT | 5 |  |  |
| cH0000018 | PotentialSSR | HexaSSR-Cp | 14256 | 14267 | TAAAGC | 2 | atpI | YP_009380202.1 [atpI]\| |
| cM0000006 | SSR | MonoSSR-Cp | 14708 | 14717 | A | 10 |  |  |
| cH0000019 | PotentialSSR | HexaSSR-Cp | 14756 | 14767 | TTTAAT | 2 |  |  |
| cP0000033 | PotentialSSR | PentaSSR-Cp | 15626 | 15635 | ATTAA | 2 |  |  |
| c80000003 | ExtendedSSR | OctaSSR-Cp | 15685 | 15700 | TCTACCGC | 2 |  |  |
| cP0000034 | PotentialSSR | PentaSSR-Cp | 15764 | 15773 | TTTAT | 2 |  |  |
| cP0000035 | PotentialSSR | PentaSSR-Cp | 15777 | 15786 | TAAAT | 2 |  |  |
| cP0000036 | PotentialSSR | PentaSSR-Cp | 16841 | 16850 | CAATT | 2 | rpoC2 | YP_009380204.1 [rpoC2]\| |
| cP0000037 | PotentialSSR | PentaSSR-Cp | 17656 | 17665 | CAAAA | 2 | rpoC2 | YP_009380204.1 [rpoC2]\| |
| cP0000038 | PotentialSSR | PentaSSR-Cp | 17776 | 17785 | TATCT | 2 | rpoC2 | YP_009380204.1 [rpoC2]\| |
| cM0000007 | SSR | MonoSSR-Cp | 17832 | 17844 | T | 13 | rpoC2 | YP_009380204.1 [rpoC2]\| |
| cP0000039 | PotentialSSR | PentaSSR-Cp | 18315 | 18324 | CGATT | 2 | rpoC2 | YP_009380204.1 [rpoC2]\| |
| cP0000040 | PotentialSSR | PentaSSR-Cp | 18344 | 18353 | ATCCT | 2 | rpoC2 | YP_009380204.1 [rpoC2]\| |
| cH0000020 | PotentialSSR | HexaSSR-Cp | 18457 | 18468 | TTGATC | 2 | rpoC2 | YP_009380204.1 [rpoC2]\| |
| cH0000021 | PotentialSSR | HexaSSR-Cp | 18829 | 18840 | ACGTGT | 2 | rpoC2 | YP_009380204.1 [rpoC2]\| |
| cP0000041 | PotentialSSR | PentaSSR-Cp | 19041 | 19050 | CATAA | 2 | rpoC2 | YP_009380204.1 [rpoC2]\| |
| cD0000003 | SSR | DiSSR-Cp | 19184 | 19193 | AT | 5 | rpoC2 | YP_009380204.1 [rpoC2]\| |
| cH0000022 | PotentialSSR | HexaSSR-Cp | 21523 | 21534 | CAAATC | 2 | rpoC1 | YP_009380205.1 [rpoC1]\| |
| cP0000042 | PotentialSSR | PentaSSR-Cp | 21637 | 21646 | GGATT | 2 | rpoC1 | YP_009380205.1 [rpoC1]\| |
| cP0000043 | PotentialSSR | PentaSSR-Cp | 21726 | 21735 | ATCCT | 2 | rpoC1 | (Intron)YP_009380205.1 [rpoC1]\| |
| cP0000044 | PotentialSSR | PentaSSR-Cp | 21920 | 21929 | TTCTT | 2 | rpoC1 | (Intron)YP_009380205.1 [rpoC1]\| |
| cP0000045 | PotentialSSR | PentaSSR-Cp | 21952 | 21961 | AATTA | 2 | rpoC1 | (Intron)YP_009380205.1 [rpoC1]\| |
| cP0000046 | PotentialSSR | PentaSSR-Cp | 22136 | 22145 | TTAAA | 2 | rpoC1 | (Intron)YP_009380205.1 [rpoC1]\| |
| cH0000023 | PotentialSSR | HexaSSR-Cp | 22241 | 22252 | ACAAAA | 2 | rpoC1 | (Intron)YP_009380205.1 [rpoC1]\| |
| cM0000008 | SSR | MonoSSR-Cp | 25509 | 25518 | T | 10 | rpoB | YP_009380206.1 [rpoB]\| |
| cH0000024 | PotentialSSR | HexaSSR-Cp | 25952 | 25963 | TCTTTT | 2 | rpoB | YP_009380206.1 [rpoB]\| |
| cP0000047 | PotentialSSR | PentaSSR-Cp | 26431 | 26440 | ACTTA | 2 |  |  |
| cP0000048 | PotentialSSR | PentaSSR-Cp | 26724 | 26733 | TTGTA | 2 |  |  |
| cH0000026 | PotentialSSR | HexaSSR-Cp | 26885 | 26896 | AGAAAA | 2 |  |  |
| cP0000049 | PotentialSSR | PentaSSR-Cp | 27101 | 27110 | GAATC | 2 |  |  |
| cP0000050 | PotentialSSR | PentaSSR-Cp | 27561 | 27570 | TTCCC | 2 |  |  |
| cP0000051 | PotentialSSR | PentaSSR-Cp | 27690 | 27699 | CTGAG | 2 |  |  |
| cH0000027 | PotentialSSR | HexaSSR-Cp | 27802 | 27813 | TTCATA | 2 |  |  |
| cP0000052 | PotentialSSR | PentaSSR-Cp | 27934 | 27943 | TTTAG | 2 |  |  |
| cH0000028 | PotentialSSR | HexaSSR-Cp | 27960 | 27971 | TATCAT | 2 |  |  |
| cP0000053 | PotentialSSR | PentaSSR-Cp | 28193 | 28202 | ATTTC | 2 |  |  |
| cP0000054 | PotentialSSR | PentaSSR-Cp | 28261 | 28270 | TCAAT | 2 |  |  |
| cP0000055 | PotentialSSR | PentaSSR-Cp | 28287 | 28296 | TTGGC | 2 |  |  |
| c70000009 | ExtendedSSR | HeptaSSR-Cp | 28412 | 28425 | TTATAGT | 2 |  |  |
| cP0000057 | PotentialSSR | PentaSSR-Cp | 28449 | 28458 | CTAAT | 2 |  |  |
| cP0000058 | PotentialSSR | PentaSSR-Cp | 28825 | 28834 | TATCA | 2 |  |  |
| cH0000029 | PotentialSSR | HexaSSR-Cp | 29050 | 29061 | CAAAAA | 2 |  |  |
| cM0000009 | SSR | MonoSSR-Cp | 29376 | 29385 | A | 10 |  |  |
| cP0000059 | PotentialSSR | PentaSSR-Cp | 29754 | 29763 | ATAAA | 2 |  |  |
| c80000004 | ExtendedSSR | OctaSSR-Cp | 29773 | 29788 | TTTTCTTT | 2 |  |  |
| cP0000060 | PotentialSSR | PentaSSR-Cp | 29830 | 29839 | ACCAT | 2 |  |  |
| cH0000030 | PotentialSSR | HexaSSR-Cp | 29870 | 29881 | AATTTC | 2 |  |  |
| cTe0000003 | SSR | TetraSSR-Cp | 29889 | 29900 | AAAT | 3 |  |  |
| cP0000061 | PotentialSSR | PentaSSR-Cp | 30108 | 30117 | TCAAT | 2 |  |  |
| cP0000062 | PotentialSSR | PentaSSR-Cp | 30159 | 30168 | TACCC | 2 | trnE-UUC | trnE-UUC [trnE-UUC]\| |
| cH0000031 | PotentialSSR | HexaSSR-Cp | 30363 | 30374 | ATTTCA | 2 |  |  |
| cH0000032 | PotentialSSR | HexaSSR-Cp | 30501 | 30512 | CTAATA | 2 |  |  |
| cP0000063 | PotentialSSR | PentaSSR-Cp | 30645 | 30654 | GTATA | 2 |  |  |
| cP0000064 | PotentialSSR | PentaSSR-Cp | 30957 | 30966 | ATTAT | 2 |  |  |
| cP0000065 | PotentialSSR | PentaSSR-Cp | 31030 | 31039 | TGGAA | 2 |  |  |
| c70000010 | ExtendedSSR | HeptaSSR-Cp | 31491 | 31504 | GTTTATT | 2 |  |  |
| cH0000033 | PotentialSSR | HexaSSR-Cp | 31568 | 31579 | GTGAAA | 2 |  |  |
| cP0000066 | PotentialSSR | PentaSSR-Cp | 31744 | 31753 | AAAGA | 2 |  |  |
| cP0000067 | PotentialSSR | PentaSSR-Cp | 31951 | 31960 | TAAAT | 2 |  |  |
| cP0000068 | PotentialSSR | PentaSSR-Cp | 32114 | 32123 | CGTTT | 2 | psbD | YP_009380209.1 [psbD]\| |
| cP0000069 | PotentialSSR | PentaSSR-Cp | 32744 | 32753 | AACCC | 2 | psbD | YP_009380209.1 [psbD]\| |
| cP0000070 | PotentialSSR | PentaSSR-Cp | 33837 | 33846 | GTCTG | 2 | psbC | YP_009380210.1 [psbC]\| |
| cH0000034 | PotentialSSR | HexaSSR-Cp | 33973 | 33984 | CTCAAG | 2 | psbC | YP_009380210.1 [psbC]\| |
| cP0000071 | PotentialSSR | PentaSSR-Cp | 34265 | 34274 | GGGTG | 2 | psbC | YP_009380210.1 [psbC]\| |
| cH0000035 | PotentialSSR | HexaSSR-Cp | 34391 | 34402 | TGCAGC | 2 | psbC | YP_009380210.1 [psbC]\| |
| c70000011 | ExtendedSSR | HeptaSSR-Cp | 34514 | 34527 | TACATAT | 2 |  |  |
| cP0000072 | PotentialSSR | PentaSSR-Cp | 34592 | 34601 | TAATT | 2 |  |  |
| cH0000036 | PotentialSSR | HexaSSR-Cp | 34823 | 34834 | TTATTC | 2 |  |  |
| cP0000073 | PotentialSSR | PentaSSR-Cp | 34925 | 34934 | TATAT | 2 |  |  |
| c70000012 | ExtendedSSR | HeptaSSR-Cp | 34965 | 34978 | TTAATTA | 2 |  |  |
| cP0000074 | PotentialSSR | PentaSSR-Cp | 35301 | 35310 | TGGAT | 2 | psbZ | YP_009380211.1 [psbZ]\| |
| cP0000075 | PotentialSSR | PentaSSR-Cp | 35469 | 35478 | AAACA | 2 |  |  |
| cP0000076 | PotentialSSR | PentaSSR-Cp | 35632 | 35641 | TATAT | 2 |  |  |
| cH0000037 | PotentialSSR | HexaSSR-Cp | 35798 | 35809 | GTTATA | 2 |  |  |
| cP0000077 | PotentialSSR | PentaSSR-Cp | 35821 | 35830 | TAGTG | 2 |  |  |
| cP0000078 | PotentialSSR | PentaSSR-Cp | 35924 | 35933 | TATTT | 2 |  |  |
| cP0000079 | PotentialSSR | PentaSSR-Cp | 36835 | 36844 | CCACG | 2 | psaB | YP_009380213.1 [psaB]\| |
| cP0000080 | PotentialSSR | PentaSSR-Cp | 36984 | 36993 | CCATC | 2 | psaB | YP_009380213.1 [psaB]\| |
| cP0000081 | PotentialSSR | PentaSSR-Cp | 38263 | 38272 | TGTCC | 2 | psaB | YP_009380213.1 [psaB]\| |
| cP0000082 | PotentialSSR | PentaSSR-Cp | 38769 | 38778 | ACCAA | 2 | psaB | YP_009380213.1 [psaB]\| |
| cH0000038 | PotentialSSR | HexaSSR-Cp | 39195 | 39206 | TAATAG | 2 | psaA | YP_009380214.1 [psaA]\| |
| cP0000083 | PotentialSSR | PentaSSR-Cp | 39944 | 39953 | ATGTG | 2 | psaA | YP_009380214.1 [psaA]\| |
| cP0000084 | PotentialSSR | PentaSSR-Cp | 41581 | 41590 | TATTT | 2 |  |  |
| cH0000039 | PotentialSSR | HexaSSR-Cp | 41759 | 41770 | TCTTTA | 2 |  |  |
| cP0000085 | PotentialSSR | PentaSSR-Cp | 41853 | 41862 | TTTTA | 2 |  |  |
| cM0000010 | SSR | MonoSSR-Cp | 41892 | 41901 | A | 10 |  |  |
| cP0000086 | PotentialSSR | PentaSSR-Cp | 42434 | 42443 | AAAGA | 2 | ycf3 | (Intron)YP_009380215.1 [ycf3]\| |
| cP0000087 | PotentialSSR | PentaSSR-Cp | 42571 | 42580 | TTCTT | 2 | ycf3 | (Intron)YP_009380215.1 [ycf3]\| |
| cM0000011 | SSR | MonoSSR-Cp | 42644 | 42653 | T | 10 | ycf3 | (Intron)YP_009380215.1 [ycf3]\| |
| cP0000088 | PotentialSSR | PentaSSR-Cp | 43145 | 43154 | AATAT | 2 | ycf3 | (Intron)YP_009380215.1 [ycf3]\| |
| cP0000089 | PotentialSSR | PentaSSR-Cp | 43820 | 43829 | AAATC | 2 | ycf3 | (Intron)YP_009380215.1 [ycf3]\| |
| cP0000090 | PotentialSSR | PentaSSR-Cp | 44029 | 44038 | GTACA | 2 |  |  |
| cP0000091 | PotentialSSR | PentaSSR-Cp | 44118 | 44127 | TATTT | 2 |  |  |
| cP0000092 | PotentialSSR | PentaSSR-Cp | 44205 | 44214 | CAAAT | 2 |  |  |
| cP0000093 | PotentialSSR | PentaSSR-Cp | 44219 | 44228 | TAGTA | 2 |  |  |
| cP0000094 | PotentialSSR | PentaSSR-Cp | 44272 | 44281 | GATCA | 2 |  |  |
| cP0000095 | PotentialSSR | PentaSSR-Cp | 44341 | 44350 | AATAG | 2 |  |  |
| cP0000096 | PotentialSSR | PentaSSR-Cp | 44518 | 44527 | GATTC | 2 |  |  |
| cTe0000004 | SSR | TetraSSR-Cp | 44533 | 44544 | AATT | 3 |  |  |
| cP0000097 | PotentialSSR | PentaSSR-Cp | 44553 | 44562 | TATAT | 2 |  |  |
| cD0000004 | SSR | DiSSR-Cp | 44625 | 44634 | AT | 5 |  |  |
| cP0000098 | PotentialSSR | PentaSSR-Cp | 44653 | 44662 | TTTTA | 2 |  |  |
| cP0000099 | PotentialSSR | PentaSSR-Cp | 44704 | 44713 | ATTAC | 2 |  |  |
| cP0000100 | PotentialSSR | PentaSSR-Cp | 44752 | 44761 | ATAGT | 2 |  |  |
| cP0000101 | PotentialSSR | PentaSSR-Cp | 44837 | 44846 | ATAGT | 2 |  |  |
| cP0000102 | PotentialSSR | PentaSSR-Cp | 45025 | 45034 | CAAAT | 2 |  |  |
| cP0000103 | PotentialSSR | PentaSSR-Cp | 45201 | 45210 | GGGAT | 2 |  |  |
| cH0000040 | PotentialSSR | HexaSSR-Cp | 45268 | 45279 | TTTTCC | 2 |  |  |
| cP0000104 | PotentialSSR | PentaSSR-Cp | 45694 | 45703 | ACCTG | 2 | rps4 | YP_009380216.1 [rps4]\| |
| cD0000005 | SSR | DiSSR-Cp | 45977 | 45990 | TA | 7 |  |  |
| cP0000105 | PotentialSSR | PentaSSR-Cp | 46035 | 46044 | AAATG | 2 |  |  |
| cP0000106 | PotentialSSR | PentaSSR-Cp | 46126 | 46135 | TATTT | 2 |  |  |
| cH0000041 | PotentialSSR | HexaSSR-Cp | 46155 | 46166 | AAAAAG | 2 |  |  |
| cP0000107 | PotentialSSR | PentaSSR-Cp | 46210 | 46219 | CTTTT | 2 |  |  |
| cP0000108 | PotentialSSR | PentaSSR-Cp | 46502 | 46511 | TCAAA | 2 |  |  |
| cP0000109 | PotentialSSR | PentaSSR-Cp | 46580 | 46589 | CGATT | 2 |  |  |
| cP0000110 | PotentialSSR | PentaSSR-Cp | 46816 | 46825 | AATAA | 2 |  |  |
| cP0000111 | PotentialSSR | PentaSSR-Cp | 47405 | 47414 | CTGTG | 2 | trnL-UAA | (Intron)trnL-UAA [trnL-UAA]\| |
| cP0000112 | PotentialSSR | PentaSSR-Cp | 47498 | 47507 | TCTAT | 2 | trnL-UAA | (Intron)trnL-UAA [trnL-UAA]\| |
| cP0000113 | PotentialSSR | PentaSSR-Cp | 47889 | 47898 | TTTTC | 2 |  |  |
| cD0000006 | SSR | DiSSR-Cp | 47946 | 47955 | AT | 5 |  |  |
| cP0000114 | PotentialSSR | PentaSSR-Cp | 48260 | 48269 | CACAT | 2 |  |  |
| cH0000043 | PotentialSSR | HexaSSR-Cp | 48389 | 48400 | GAAAAT | 2 |  |  |
| cP0000115 | PotentialSSR | PentaSSR-Cp | 48520 | 48529 | CTTAA | 2 |  |  |
| cP0000116 | PotentialSSR | PentaSSR-Cp | 48539 | 48548 | TCTTT | 2 |  |  |
| cH0000044 | PotentialSSR | HexaSSR-Cp | 49008 | 49019 | CATATA | 2 | ndhJ | YP_009380217.1 [ndhJ]\| |
| c80000006 | ExtendedSSR | OctaSSR-Cp | 49165 | 49180 | ATTTGTTT | 2 |  |  |
| cP0000117 | PotentialSSR | PentaSSR-Cp | 49188 | 49197 | TATTT | 2 |  |  |
| cP0000118 | PotentialSSR | PentaSSR-Cp | 49201 | 49210 | TTTAC | 2 |  |  |
| cH0000045 | PotentialSSR | HexaSSR-Cp | 49968 | 49979 | TTTTTC | 2 |  |  |
| cP0000119 | PotentialSSR | PentaSSR-Cp | 50033 | 50042 | TAAAC | 2 | ndhC | YP_009380219.1 [ndhC]\| |
| cP0000120 | PotentialSSR | PentaSSR-Cp | 50595 | 50604 | TTCTT | 2 |  |  |
| cP0000121 | PotentialSSR | PentaSSR-Cp | 50671 | 50680 | AATTC | 2 |  |  |
| cP0000122 | PotentialSSR | PentaSSR-Cp | 50727 | 50736 | TTCTA | 2 |  |  |
| cP0000123 | PotentialSSR | PentaSSR-Cp | 50786 | 50795 | ACTTG | 2 |  |  |
| c70000015 | ExtendedSSR | HeptaSSR-Cp | 52543 | 52556 | AATTTTT | 2 |  |  |
| cP0000124 | PotentialSSR | PentaSSR-Cp | 52681 | 52690 | ATTTG | 2 |  |  |
| cP0000125 | PotentialSSR | PentaSSR-Cp | 52782 | 52791 | TTGAA | 2 |  |  |
| cH0000047 | PotentialSSR | HexaSSR-Cp | 52835 | 52846 | TGTATA | 2 |  |  |
| cP0000126 | PotentialSSR | PentaSSR-Cp | 53062 | 53071 | ATTGA | 2 |  |  |
| cP0000127 | PotentialSSR | PentaSSR-Cp | 53101 | 53110 | AATTG | 2 |  |  |
| cM0000012 | SSR | MonoSSR-Cp | 53192 | 53203 | T | 12 |  |  |
| cH0000048 | PotentialSSR | HexaSSR-Cp | 53214 | 53225 | ATTTTC | 2 |  |  |
| cM0000013 | SSR | MonoSSR-Cp | 53299 | 53308 | A | 10 | atpB | YP_009380221.1 [atpB]\| |
| cP0000128 | PotentialSSR | PentaSSR-Cp | 54475 | 54484 | TTGGA | 2 | atpB | YP_009380221.1 [atpB]\| |
| cH0000049 | PotentialSSR | HexaSSR-Cp | 55172 | 55183 | ATAATC | 2 |  |  |
| cH0000050 | PotentialSSR | HexaSSR-Cp | 55242 | 55253 | TCTTTT | 2 |  |  |
| cH0000051 | PotentialSSR | HexaSSR-Cp | 55280 | 55291 | AAAAAG | 2 |  |  |
| cP0000129 | PotentialSSR | PentaSSR-Cp | 55754 | 55763 | AAACA | 2 | trnV-UAC | (Intron)trnV-UAC [trnV-UAC]\| |
| c70000016 | ExtendedSSR | HeptaSSR-Cp | 56086 | 56099 | AATGAAT | 2 | trnV-UAC | (Intron)trnV-UAC [trnV-UAC]\| |
| cH0000052 | PotentialSSR | HexaSSR-Cp | 56332 | 56343 | CAATTG | 2 |  |  |
| cP0000130 | PotentialSSR | PentaSSR-Cp | 56393 | 56402 | ATTTG | 2 |  |  |
| cH0000053 | PotentialSSR | HexaSSR-Cp | 56417 | 56428 | TTTGAA | 2 |  |  |
| cP0000131 | PotentialSSR | PentaSSR-Cp | 56516 | 56525 | TTTTC | 2 |  |  |
| cP0000132 | PotentialSSR | PentaSSR-Cp | 58582 | 58591 | TATAT | 2 |  |  |
| cP0000133 | PotentialSSR | PentaSSR-Cp | 58638 | 58647 | CTATA | 2 |  |  |
| cTe0000005 | SSR | TetraSSR-Cp | 58665 | 58676 | TAAT | 3 |  |  |
| cP0000134 | PotentialSSR | PentaSSR-Cp | 58679 | 58688 | TAATA | 2 |  |  |
| cH0000054 | PotentialSSR | HexaSSR-Cp | 58720 | 58731 | TACAAT | 2 |  |  |
| cP0000135 | PotentialSSR | PentaSSR-Cp | 58850 | 58859 | AAAAT | 2 | psaI | YP_009380224.1 [psaI]\| |
| cP0000136 | PotentialSSR | PentaSSR-Cp | 58868 | 58877 | TCGTC | 2 |  |  |
| cP0000137 | PotentialSSR | PentaSSR-Cp | 59008 | 59017 | ACAAT | 2 |  |  |
| cP0000138 | PotentialSSR | PentaSSR-Cp | 59172 | 59181 | TTCAA | 2 |  |  |
| cP0000139 | PotentialSSR | PentaSSR-Cp | 59482 | 59491 | TCGAT | 2 | ycf4 | YP_009380225.1 [ycf4]\| |
| cP0000140 | PotentialSSR | PentaSSR-Cp | 59587 | 59596 | TAGAA | 2 | ycf4 | YP_009380225.1 [ycf4]\| |
| cH0000055 | PotentialSSR | HexaSSR-Cp | 59920 | 59931 | TACAAA | 2 |  |  |
| cP0000141 | PotentialSSR | PentaSSR-Cp | 59933 | 59942 | CAATT | 2 |  |  |
| cP0000142 | PotentialSSR | PentaSSR-Cp | 60073 | 60082 | AATTA | 2 |  |  |
| cP0000143 | PotentialSSR | PentaSSR-Cp | 61038 | 61047 | AAAAG | 2 |  |  |
| c70000017 | ExtendedSSR | HeptaSSR-Cp | 61102 | 61115 | ATAATCA | 2 |  |  |
| cP0000144 | PotentialSSR | PentaSSR-Cp | 61187 | 61196 | TTATT | 2 |  |  |
| cP0000145 | PotentialSSR | PentaSSR-Cp | 61685 | 61694 | AAAGA | 2 | petA | YP_009380227.1 [petA]\| |
| cP0000146 | PotentialSSR | PentaSSR-Cp | 61956 | 61965 | GAAAA | 2 | petA | YP_009380227.1 [petA]\| |
| cH0000056 | PotentialSSR | HexaSSR-Cp | 62276 | 62287 | ATCAAG | 2 |  |  |
| cH0000057 | PotentialSSR | HexaSSR-Cp | 62292 | 62303 | TAACAA | 2 |  |  |
| cP0000147 | PotentialSSR | PentaSSR-Cp | 62316 | 62325 | CAATT | 2 |  |  |
| cM0000014 | SSR | MonoSSR-Cp | 62350 | 62363 | A | 14 |  |  |
| c80000007 | ExtendedSSR | OctaSSR-Cp | 62457 | 62472 | ACTTTTTT | 2 |  |  |
| cH0000058 | PotentialSSR | HexaSSR-Cp | 62664 | 62675 | ATTTTT | 2 |  |  |
| cH0000059 | PotentialSSR | HexaSSR-Cp | 62859 | 62870 | TCTTTC | 2 |  |  |
| cP0000148 | PotentialSSR | PentaSSR-Cp | 62873 | 62882 | TTAAC | 2 |  |  |
| c70000018 | ExtendedSSR | HeptaSSR-Cp | 63125 | 63138 | TTACTAC | 2 | psbJ | YP_009380228.1 [psbJ]\| |
| cP0000149 | PotentialSSR | PentaSSR-Cp | 63478 | 63487 | ATTCA | 2 | psbL | YP_009380229.1 [psbL]\| |
| cH0000060 | PotentialSSR | HexaSSR-Cp | 63529 | 63540 | ATTCGG | 2 |  |  |
| c80000008 | ExtendedSSR | OctaSSR-Cp | 64009 | 64024 | ACGTAAAA | 2 |  |  |
| cH0000061 | PotentialSSR | HexaSSR-Cp | 64346 | 64357 | TATAGA | 2 |  |  |
| cH0000062 | PotentialSSR | HexaSSR-Cp | 64585 | 64596 | AGTCTT | 2 |  |  |
| cP0000150 | PotentialSSR | PentaSSR-Cp | 65141 | 65150 | CTGTA | 2 |  |  |
| cM0000015 | SSR | MonoSSR-Cp | 65256 | 65268 | T | 13 |  |  |
| cP0000151 | PotentialSSR | PentaSSR-Cp | 65460 | 65469 | GATTA | 2 |  |  |
| cP0000152 | PotentialSSR | PentaSSR-Cp | 65477 | 65486 | TTTAG | 2 |  |  |
| cH0000063 | PotentialSSR | HexaSSR-Cp | 65496 | 65507 | TTTCTA | 2 |  |  |
| cP0000153 | PotentialSSR | PentaSSR-Cp | 65594 | 65603 | GAACT | 2 | trnW-CCA | trnW-CCA [trnW-CCA]\| |
| cH0000064 | PotentialSSR | HexaSSR-Cp | 65670 | 65681 | CTATAT | 2 |  |  |
| cP0000154 | PotentialSSR | PentaSSR-Cp | 65877 | 65886 | TTCAA | 2 |  |  |
| cP0000155 | PotentialSSR | PentaSSR-Cp | 65916 | 65925 | CCTTG | 2 |  |  |
| cP0000156 | PotentialSSR | PentaSSR-Cp | 66052 | 66061 | GTAAA | 2 |  |  |
| cP0000157 | PotentialSSR | PentaSSR-Cp | 66164 | 66173 | CTTAG | 2 |  |  |
| cP0000158 | PotentialSSR | PentaSSR-Cp | 66520 | 66529 | TTAGT | 2 |  |  |
| cP0000159 | PotentialSSR | PentaSSR-Cp | 66573 | 66582 | GTTAA | 2 |  |  |
| c70000019 | ExtendedSSR | HeptaSSR-Cp | 66714 | 66727 | CGAATTG | 2 |  |  |
| cH0000065 | PotentialSSR | HexaSSR-Cp | 67077 | 67088 | AATTTT | 2 |  |  |
| cD0000007 | SSR | DiSSR-Cp | 67102 | 67111 | AT | 5 |  |  |
| cP0000160 | PotentialSSR | PentaSSR-Cp | 67171 | 67180 | ATATA | 2 |  |  |
| cP0000161 | PotentialSSR | PentaSSR-Cp | 67246 | 67255 | ATAGG | 2 |  |  |
| cP0000162 | PotentialSSR | PentaSSR-Cp | 67273 | 67282 | TAAAC | 2 |  |  |
| cTe0000006 | SSR | TetraSSR-Cp | 67757 | 67768 | TTTA | 3 |  |  |
| cP0000163 | PotentialSSR | PentaSSR-Cp | 68320 | 68329 | ATAAA | 2 |  |  |
| cH0000066 | PotentialSSR | HexaSSR-Cp | 68811 | 68822 | ATTTTC | 2 |  |  |
| cP0000164 | PotentialSSR | PentaSSR-Cp | 68929 | 68938 | CTATT | 2 |  |  |
| cH0000067 | PotentialSSR | HexaSSR-Cp | 68976 | 68987 | TTTTGT | 2 |  |  |
| cH0000068 | PotentialSSR | HexaSSR-Cp | 69296 | 69307 | TGGGCT | 2 | clpP | YP_009380239.1 [clpP]\| |
| cP0000165 | PotentialSSR | PentaSSR-Cp | 69526 | 69535 | ACACA | 2 | clpP | (Intron)YP_009380239.1 [clpP]\| |
| cP0000166 | PotentialSSR | PentaSSR-Cp | 69671 | 69680 | ATCGA | 2 | clpP | (Intron)YP_009380239.1 [clpP]\| |
| cH0000069 | PotentialSSR | HexaSSR-Cp | 69682 | 69693 | CAGATC | 2 | clpP | (Intron)YP_009380239.1 [clpP]\| |
| cP0000167 | PotentialSSR | PentaSSR-Cp | 69967 | 69976 | GAAAA | 2 | clpP | (Intron)YP_009380239.1 [clpP]\| |
| cH0000070 | PotentialSSR | HexaSSR-Cp | 70257 | 70268 | ACAAAT | 2 | clpP | YP_009380239.1 [clpP]\| |
| cP0000168 | PotentialSSR | PentaSSR-Cp | 70670 | 70679 | TATCA | 2 | clpP | (Intron)YP_009380239.1 [clpP]\| |
| cH0000071 | PotentialSSR | HexaSSR-Cp | 70710 | 70721 | TTCTTG | 2 | clpP | (Intron)YP_009380239.1 [clpP]\| |
| cH0000072 | PotentialSSR | HexaSSR-Cp | 70920 | 70931 | TTGAAA | 2 | clpP | (Intron)YP_009380239.1 [clpP]\| |
| cH0000073 | PotentialSSR | HexaSSR-Cp | 70987 | 70998 | ATTGGG | 2 | clpP | (Intron)YP_009380239.1 [clpP]\| |
| cP0000169 | PotentialSSR | PentaSSR-Cp | 71556 | 71565 | ATAGA | 2 |  |  |
| cH0000074 | PotentialSSR | HexaSSR-Cp | 71675 | 71686 | CATAGT | 2 |  |  |
| c70000021 | ExtendedSSR | HeptaSSR-Cp | 71825 | 71838 | CTGGTTG | 2 | psbB | YP_009380240.1 [psbB]\| |
| cH0000075 | PotentialSSR | HexaSSR-Cp | 72189 | 72200 | GTTTTG | 2 | psbB | YP_009380240.1 [psbB]\| |
| cP0000170 | PotentialSSR | PentaSSR-Cp | 73295 | 73304 | TTTGA | 2 |  |  |
| cP0000171 | PotentialSSR | PentaSSR-Cp | 73452 | 73461 | CTCTA | 2 | psbT | YP_009380241.1 [psbT]\| |
| cP0000172 | PotentialSSR | PentaSSR-Cp | 73516 | 73525 | AAATG | 2 | psbT | YP_009380241.1 [psbT]\| |
| cH0000076 | PotentialSSR | HexaSSR-Cp | 74047 | 74058 | ACAAAA | 2 |  |  |
| c90000002 | ExtendedSSR | NonaSSR-Cp | 74096 | 74113 | CAATACAAA | 2 |  |  |
| cP0000173 | PotentialSSR | PentaSSR-Cp | 74399 | 74408 | TTTTC | 2 | petB | (Intron)YP_009380244.1 [petB]\| |
| cP0000175 | PotentialSSR | PentaSSR-Cp | 74529 | 74538 | CCTAT | 2 | petB | (Intron)YP_009380244.1 [petB]\| |
| cH0000077 | PotentialSSR | HexaSSR-Cp | 74705 | 74716 | ATTATA | 2 | petB | (Intron)YP_009380244.1 [petB]\| |
| cH0000078 | PotentialSSR | HexaSSR-Cp | 74717 | 74728 | TACAAA | 2 | petB | (Intron)YP_009380244.1 [petB]\| |
| cP0000176 | PotentialSSR | PentaSSR-Cp | 75659 | 75668 | ATAGA | 2 | petB | YP_009380244.1 [petB]\| |
| cP0000177 | PotentialSSR | PentaSSR-Cp | 76028 | 76037 | TCTAT | 2 | petD | (Intron)YP_009380245.1 [petD]\| |
| cP0000178 | PotentialSSR | PentaSSR-Cp | 76104 | 76113 | ATAAT | 2 | petD | (Intron)YP_009380245.1 [petD]\| |
| cP0000181 | PotentialSSR | PentaSSR-Cp | 76613 | 76622 | GAATC | 2 | petD | (Intron)YP_009380245.1 [petD]\| |
| cP0000182 | PotentialSSR | PentaSSR-Cp | 76626 | 76635 | AAGAA | 2 | petD | (Intron)YP_009380245.1 [petD]\| |
| cP0000183 | PotentialSSR | PentaSSR-Cp | 77205 | 77214 | ATTCA | 2 |  |  |
| cM0000016 | SSR | MonoSSR-Cp | 77432 | 77441 | T | 10 | rpoA | YP_009380246.1 [rpoA]\| |
| cH0000079 | PotentialSSR | HexaSSR-Cp | 77651 | 77662 | CATTTC | 2 | rpoA | YP_009380246.1 [rpoA]\| |
| cP0000184 | PotentialSSR | PentaSSR-Cp | 78081 | 78090 | TCGCA | 2 | rpoA | YP_009380246.1 [rpoA]\| |
| cP0000185 | PotentialSSR | PentaSSR-Cp | 78849 | 78858 | TAGTA | 2 |  |  |
| cM0000017 | SSR | MonoSSR-Cp | 78992 | 79002 | T | 11 |  |  |
| cH0000080 | PotentialSSR | HexaSSR-Cp | 79075 | 79086 | GTTGAA | 2 |  |  |
| cP0000186 | PotentialSSR | PentaSSR-Cp | 79907 | 79916 | TTAGT | 2 |  |  |
| cH0000081 | PotentialSSR | HexaSSR-Cp | 79978 | 79989 | TATTTT | 2 |  |  |
| cH0000082 | PotentialSSR | HexaSSR-Cp | 80985 | 80996 | AAAAAT | 2 | rpl16 | (Intron)YP_009380252.1 [rpl16]\| |
| cP0000187 | PotentialSSR | PentaSSR-Cp | 81012 | 81021 | TATTT | 2 | rpl16 | (Intron)YP_009380252.1 [rpl16]\| |
| c70000023 | ExtendedSSR | HeptaSSR-Cp | 81230 | 81243 | TTTTATA | 2 | rpl16 | (Intron)YP_009380252.1 [rpl16]\| |
| cP0000188 | PotentialSSR | PentaSSR-Cp | 81286 | 81295 | AAAAG | 2 | rpl16 | (Intron)YP_009380252.1 [rpl16]\| |
| cM0000018 | SSR | MonoSSR-Cp | 81572 | 81581 | T | 10 | rpl16 | (Intron)YP_009380252.1 [rpl16]\| |
| cM0000019 | SSR | MonoSSR-Cp | 81705 | 81715 | T | 11 | rpl16 | (Intron)YP_009380252.1 [rpl16]\| |
| cM0000020 | SSR | MonoSSR-Cp | 81764 | 81773 | T | 10 | rpl16 | (Intron)YP_009380252.1 [rpl16]\| |
| cH0000084 | PotentialSSR | HexaSSR-Cp | 81953 | 81964 | TTTTAA | 2 | rpl16 | (Intron)YP_009380252.1 [rpl16]\| |
| cP0000189 | PotentialSSR | PentaSSR-Cp | 82260 | 82269 | ACCCT | 2 | rps3 | YP_009380253.1 [rps3]\| |
| cP0000190 | PotentialSSR | PentaSSR-Cp | 82394 | 82403 | TACTC | 2 | rps3 | YP_009380253.1 [rps3]\| |
| cP0000191 | PotentialSSR | PentaSSR-Cp | 82497 | 82506 | CAATT | 2 | rps3 | YP_009380253.1 [rps3]\| |
| cP0000192 | PotentialSSR | PentaSSR-Cp | 82855 | 82864 | TTTAT | 2 | rpl22 | YP_009380254.1 [rpl22]\| |
| cH0000085 | PotentialSSR | HexaSSR-Cp | 83805 | 83816 | ATTTTC | 2 |  |  |
| cP0000194 | PotentialSSR | PentaSSR-Cp | 84220 | 84229 | TATGT | 2 | rpl2 | YP_009380256.1 [rpl2]\| |
| cP0000195 | PotentialSSR | PentaSSR-Cp | 84568 | 84577 | TGGAT | 2 | rpl2 | YP_009380256.1 [rpl2]\| |
| cP0000196 | PotentialSSR | PentaSSR-Cp | 84669 | 84678 | TTCTT | 2 |  |  |
| cP0000197 | PotentialSSR | PentaSSR-Cp | 84690 | 84699 | GAATA | 2 |  |  |
| cP0000198 | PotentialSSR | PentaSSR-Cp | 85048 | 85057 | TATGA | 2 |  |  |
| cP0000199 | PotentialSSR | PentaSSR-Cp | 85264 | 85273 | TGAAA | 2 |  |  |
| cH0000086 | PotentialSSR | HexaSSR-Cp | 85930 | 85941 | GAAAGA | 2 | ycf2 | YP_009380257.1 [ycf2]\| |
| cP0000200 | PotentialSSR | PentaSSR-Cp | 85982 | 85991 | GATCC | 2 | ycf2 | YP_009380257.1 [ycf2]\| |
| c90000003 | ExtendedSSR | NonaSSR-Cp | 87262 | 87279 | GGAACATTT | 2 | ycf2 | YP_009380257.1 [ycf2]\| |
| cP0000201 | PotentialSSR | PentaSSR-Cp | 88109 | 88118 | CGATC | 2 | ycf2 | YP_009380257.1 [ycf2]\| |
| cP0000202 | PotentialSSR | PentaSSR-Cp | 88274 | 88283 | TTCAA | 2 | ycf2 | YP_009380257.1 [ycf2]\| |
| cH0000089 | PotentialSSR | HexaSSR-Cp | 89227 | 89238 | AAGAAA | 2 | ycf2 | YP_009380257.1 [ycf2]\| |
| cP0000203 | PotentialSSR | PentaSSR-Cp | 89252 | 89261 | GATTG | 2 | ycf2 | YP_009380257.1 [ycf2]\| |
| cH0000090 | PotentialSSR | HexaSSR-Cp | 89893 | 89904 | GGAGCT | 2 | ycf2 | YP_009380257.1 [ycf2]\| |
| cP0000204 | PotentialSSR | PentaSSR-Cp | 90275 | 90284 | GAAAA | 2 | ycf2 | YP_009380257.1 [ycf2]\| |
| cH0000091 | PotentialSSR | HexaSSR-Cp | 90780 | 90791 | TAGAAG | 2 | ycf2 | YP_009380257.1 [ycf2]\| |
| cH0000092 | PotentialSSR | HexaSSR-Cp | 91380 | 91391 | CTATAT | 2 | ycf2 | YP_009380257.1 [ycf2]\| |
| cP0000205 | PotentialSSR | PentaSSR-Cp | 91874 | 91883 | AAGTT | 2 |  |  |
| cP0000206 | PotentialSSR | PentaSSR-Cp | 91902 | 91911 | TTGTT | 2 |  |  |
| cP0000207 | PotentialSSR | PentaSSR-Cp | 92146 | 92155 | GTTAC | 2 |  |  |
| cH0000093 | PotentialSSR | HexaSSR-Cp | 92321 | 92332 | ATTCCA | 2 |  |  |
| cP0000208 | PotentialSSR | PentaSSR-Cp | 92637 | 92646 | CTTAT | 2 |  |  |
| cP0000209 | PotentialSSR | PentaSSR-Cp | 92772 | 92781 | ATGGA | 2 |  |  |
| cH0000094 | PotentialSSR | HexaSSR-Cp | 93733 | 93744 | GCTGAA | 2 | ndhB | YP_009380258.1 [ndhB]\| |
| cH0000095 | PotentialSSR | HexaSSR-Cp | 93792 | 93803 | AGAGTC | 2 | ndhB | YP_009380258.1 [ndhB]\| |
| cP0000210 | PotentialSSR | PentaSSR-Cp | 94088 | 94097 | TGATT | 2 | ndhB | (Intron)YP_009380258.1 [ndhB]\| |
| cP0000211 | PotentialSSR | PentaSSR-Cp | 94288 | 94297 | AAAGA | 2 | ndhB | (Intron)YP_009380258.1 [ndhB]\| |
| cH0000096 | PotentialSSR | HexaSSR-Cp | 95355 | 95366 | TTCTTA | 2 |  |  |
| cP0000212 | PotentialSSR | PentaSSR-Cp | 95421 | 95430 | AGAAA | 2 |  |  |
| cP0000213 | PotentialSSR | PentaSSR-Cp | 95525 | 95534 | CTGTT | 2 |  |  |
| cH0000097 | PotentialSSR | HexaSSR-Cp | 96272 | 96283 | TCCATA | 2 |  |  |
| cP0000214 | PotentialSSR | PentaSSR-Cp | 96460 | 96469 | CGAAT | 2 |  |  |
| cH0000098 | PotentialSSR | HexaSSR-Cp | 97049 | 97060 | TTGATT | 2 |  |  |
| cH0000099 | PotentialSSR | HexaSSR-Cp | 97096 | 97107 | TTCCTC | 2 |  |  |
| cH0000100 | PotentialSSR | HexaSSR-Cp | 97108 | 97119 | TATCCC | 2 |  |  |
| cP0000215 | PotentialSSR | PentaSSR-Cp | 97354 | 97363 | TGTTG | 2 |  |  |
| cP0000216 | PotentialSSR | PentaSSR-Cp | 97452 | 97461 | TATTA | 2 |  |  |
| cP0000217 | PotentialSSR | PentaSSR-Cp | 97471 | 97480 | ATTAG | 2 |  |  |
| cP0000218 | PotentialSSR | PentaSSR-Cp | 97653 | 97662 | GCAAT | 2 |  |  |
| cH0000101 | PotentialSSR | HexaSSR-Cp | 97773 | 97784 | TATTAC | 2 |  |  |
| cH0000102 | PotentialSSR | HexaSSR-Cp | 97920 | 97931 | AATGGA | 2 |  |  |
| cM0000021 | SSR | MonoSSR-Cp | 98030 | 98041 | T | 12 |  |  |
| cP0000219 | PotentialSSR | PentaSSR-Cp | 98281 | 98290 | CAAGA | 2 |  |  |
| cP0000220 | PotentialSSR | PentaSSR-Cp | 98364 | 98373 | AGGGA | 2 |  |  |
| cH0000103 | PotentialSSR | HexaSSR-Cp | 98488 | 98499 | GAATGA | 2 |  |  |
| cH0000104 | PotentialSSR | HexaSSR-Cp | 99361 | 99372 | GACACT | 2 | rrn16 | rrn16 [rrn16]\| |
| cH0000105 | PotentialSSR | HexaSSR-Cp | 100818 | 100829 | AATGGA | 2 | trnI-GAU | (Intron)trnI-GAU [trnI-GAU]\| |
| cH0000106 | PotentialSSR | HexaSSR-Cp | 101574 | 101585 | AAGAAT | 2 |  |  |
| cP0000221 | PotentialSSR | PentaSSR-Cp | 101805 | 101814 | ACAAA | 2 | trnA-UGC | (Intron)trnA-UGC [trnA-UGC]\| |
| cP0000222 | PotentialSSR | PentaSSR-Cp | 102124 | 102133 | TTCAA | 2 | trnA-UGC | (Intron)trnA-UGC [trnA-UGC]\| |
| c80000010 | ExtendedSSR | OctaSSR-Cp | 102348 | 102363 | TTTTGAGA | 2 | trnA-UGC | (Intron)trnA-UGC [trnA-UGC]\| |
| cD0000008 | SSR | DiSSR-Cp | 103923 | 103932 | CG | 5 | rrn23 | rrn23 [rrn23]\| |
| cH0000107 | PotentialSSR | HexaSSR-Cp | 103946 | 103957 | GAAGCG | 2 | rrn23 | rrn23 [rrn23]\| |
| cTe0000007 | SSR | TetraSSR-Cp | 104340 | 104351 | AGGT | 3 | rrn23 | rrn23 [rrn23]\| |
| cP0000223 | PotentialSSR | PentaSSR-Cp | 105433 | 105442 | GCGGA | 2 | rrn23 | rrn23 [rrn23]\| |
| cP0000224 | PotentialSSR | PentaSSR-Cp | 105731 | 105740 | ATCCA | 2 |  |  |
| c70000024 | ExtendedSSR | HeptaSSR-Cp | 105803 | 105816 | AAAAACC | 2 |  |  |
| cH0000108 | PotentialSSR | HexaSSR-Cp | 105833 | 105844 | TCTATC | 2 |  |  |
| cH0000109 | PotentialSSR | HexaSSR-Cp | 106210 | 106221 | TTCTTA | 2 |  |  |
| cP0000225 | PotentialSSR | PentaSSR-Cp | 106507 | 106516 | AGTGG | 2 |  |  |
| cH0000110 | PotentialSSR | HexaSSR-Cp | 106647 | 106658 | CAAGTA | 2 |  |  |
| cP0000226 | PotentialSSR | PentaSSR-Cp | 106663 | 106672 | TAGCA | 2 |  |  |
| cP0000227 | PotentialSSR | PentaSSR-Cp | 106687 | 106696 | GTCAT | 2 |  |  |
| c70000025 | ExtendedSSR | HeptaSSR-Cp | 106700 | 106713 | TATGTTT | 2 |  |  |
| cP0000228 | PotentialSSR | PentaSSR-Cp | 106872 | 106881 | CAGAA | 2 |  |  |
| c70000026 | ExtendedSSR | HeptaSSR-Cp | 106978 | 106991 | AAGAATG | 2 |  |  |
| c90000004 | ExtendedSSR | NonaSSR-Cp | 107539 | 107556 | GAAGAAGGA | 2 | ycf1 | YP_009380260.1 [ycf1]\| |
| cH0000112 | PotentialSSR | HexaSSR-Cp | 108161 | 108172 | TAGAAA | 2 | ycf1 | YP_009380260.1 [ycf1]\| |
| cH0000113 | PotentialSSR | HexaSSR-Cp | 108228 | 108239 | TCCTTC | 2 | ycf1 | YP_009380260.1 [ycf1]\| |
| cP0000229 | PotentialSSR | PentaSSR-Cp | 108251 | 108260 | AAGAA | 2 | ycf1 | YP_009380260.1 [ycf1]\| |
| cH0000114 | PotentialSSR | HexaSSR-Cp | 108270 | 108281 | CAAAAT | 2 | ycf1 | YP_009380260.1 [ycf1]\| |
| cP0000230 | PotentialSSR | PentaSSR-Cp | 108317 | 108326 | ACAAA | 2 | ycf1 | YP_009380260.1 [ycf1]\| |
| cP0000231 | PotentialSSR | PentaSSR-Cp | 108542 | 108551 | GAAAT | 2 | ycf1 | YP_009380260.1 [ycf1]\| |
| cM0000022 | SSR | MonoSSR-Cp | 108728 | 108737 | A | 10 | ycf1 | YP_009380260.1 [ycf1]\| |
| cH0000115 | PotentialSSR | HexaSSR-Cp | 110000 | 110011 | AGATCC | 2 | ndhF | YP_009380261.1 [ndhF]\| |
| c70000027 | ExtendedSSR | HeptaSSR-Cp | 110467 | 110480 | CTCGAAA | 2 | ndhF | YP_009380261.1 [ndhF]\| |
| cP0000232 | PotentialSSR | PentaSSR-Cp | 111001 | 111010 | AAAAG | 2 | ndhF | YP_009380261.1 [ndhF]\| |
| cP0000233 | PotentialSSR | PentaSSR-Cp | 111093 | 111102 | TAGAA | 2 |  |  |
| c70000028 | ExtendedSSR | HeptaSSR-Cp | 111266 | 111279 | TTAAAAC | 2 |  |  |
| cP0000234 | PotentialSSR | PentaSSR-Cp | 111522 | 111531 | TTACT | 2 |  |  |
| cM0000023 | SSR | MonoSSR-Cp | 111920 | 111929 | A | 10 |  |  |
| cM0000024 | SSR | MonoSSR-Cp | 112237 | 112246 | A | 10 |  |  |
| cP0000235 | PotentialSSR | PentaSSR-Cp | 112485 | 112494 | TTTTA | 2 |  |  |
| c70000029 | ExtendedSSR | HeptaSSR-Cp | 112505 | 112518 | TAAAAGA | 2 |  |  |
| cM0000025 | SSR | MonoSSR-Cp | 112556 | 112565 | A | 10 |  |  |
| cP0000236 | PotentialSSR | PentaSSR-Cp | 112852 | 112861 | AGAAA | 2 |  |  |
| cP0000237 | PotentialSSR | PentaSSR-Cp | 112981 | 112990 | GAAAA | 2 |  |  |
| cH0000116 | PotentialSSR | HexaSSR-Cp | 113167 | 113178 | TATGAA | 2 |  |  |
| cH0000117 | PotentialSSR | HexaSSR-Cp | 113427 | 113438 | AATGAA | 2 |  |  |
| cM0000026 | SSR | MonoSSR-Cp | 113486 | 113495 | T | 10 |  |  |
| cP0000238 | PotentialSSR | PentaSSR-Cp | 114613 | 114622 | TTTTG | 2 |  |  |
| cP0000239 | PotentialSSR | PentaSSR-Cp | 114651 | 114660 | TTTCA | 2 |  |  |
| cH0000118 | PotentialSSR | HexaSSR-Cp | 114749 | 114760 | AATACC | 2 | ndhD | YP_009380264.1 [ndhD]\| |
| cM0000027 | SSR | MonoSSR-Cp | 114815 | 114824 | A | 10 | ndhD | YP_009380264.1 [ndhD]\| |
| cH0000119 | PotentialSSR | HexaSSR-Cp | 115745 | 115756 | TAATTC | 2 | ndhD | YP_009380264.1 [ndhD]\| |
| cH0000120 | PotentialSSR | HexaSSR-Cp | 116597 | 116608 | TCTAGT | 2 |  |  |
| cP0000240 | PotentialSSR | PentaSSR-Cp | 117232 | 117241 | AAGTT | 2 |  |  |
| cP0000241 | PotentialSSR | PentaSSR-Cp | 117532 | 117541 | ATACC | 2 | ndhG | YP_009380267.1 [ndhG]\| |
| c70000030 | ExtendedSSR | HeptaSSR-Cp | 117744 | 117757 | TAGAATA | 2 | ndhG | YP_009380267.1 [ndhG]\| |
| cP0000242 | PotentialSSR | PentaSSR-Cp | 118019 | 118028 | TAAAG | 2 |  |  |
| cP0000243 | PotentialSSR | PentaSSR-Cp | 118184 | 118193 | TTTAA | 2 |  |  |
| cP0000244 | PotentialSSR | PentaSSR-Cp | 118305 | 118314 | TAATT | 2 | ndhI | YP_009380268.1 [ndhI]\| |
| cH0000121 | PotentialSSR | HexaSSR-Cp | 118947 | 118958 | GAACAA | 2 | ndhA | YP_009380269.1 [ndhA]\| |
| cP0000245 | PotentialSSR | PentaSSR-Cp | 118971 | 118980 | TAATG | 2 | ndhA | YP_009380269.1 [ndhA]\| |
| cP0000246 | PotentialSSR | PentaSSR-Cp | 119124 | 119133 | ATAAA | 2 | ndhA | YP_009380269.1 [ndhA]\| |
| cP0000247 | PotentialSSR | PentaSSR-Cp | 119352 | 119361 | AAGAT | 2 | ndhA | (Intron)YP_009380269.1 [ndhA]\| |
| cP0000248 | PotentialSSR | PentaSSR-Cp | 119540 | 119549 | CTATA | 2 | ndhA | (Intron)YP_009380269.1 [ndhA]\| |
| c70000031 | ExtendedSSR | HeptaSSR-Cp | 119662 | 119675 | TATCAAT | 2 | ndhA | (Intron)YP_009380269.1 [ndhA]\| |
| cM0000028 | SSR | MonoSSR-Cp | 119791 | 119800 | T | 10 | ndhA | (Intron)YP_009380269.1 [ndhA]\| |
| cH0000122 | PotentialSSR | HexaSSR-Cp | 119805 | 119816 | CTATTA | 2 | ndhA | (Intron)YP_009380269.1 [ndhA]\| |
| cP0000249 | PotentialSSR | PentaSSR-Cp | 121256 | 121265 | CATTC | 2 | ndhH | YP_009380270.1 [ndhH]\| |
| cH0000123 | PotentialSSR | HexaSSR-Cp | 122265 | 122276 | ATAATT | 2 |  |  |
| cP0000250 | PotentialSSR | PentaSSR-Cp | 122670 | 122679 | TTTAT | 2 |  |  |
| cH0000124 | PotentialSSR | HexaSSR-Cp | 122955 | 122966 | AATTTT | 2 | ycf1 | YP_009380272.1 [ycf1]\| |
| cP0000251 | PotentialSSR | PentaSSR-Cp | 123395 | 123404 | TTCTT | 2 | ycf1 | YP_009380272.1 [ycf1]\| |
| cM0000029 | SSR | MonoSSR-Cp | 123974 | 123985 | T | 12 | ycf1 | YP_009380272.1 [ycf1]\| |
| cH0000125 | PotentialSSR | HexaSSR-Cp | 124341 | 124352 | CTATAT | 2 | ycf1 | YP_009380272.1 [ycf1]\| |
| cH0000126 | PotentialSSR | HexaSSR-Cp | 124439 | 124450 | CAATAA | 2 | ycf1 | YP_009380272.1 [ycf1]\| |
| cTe0000008 | SSR | TetraSSR-Cp | 124473 | 124484 | TAAT | 3 | ycf1 | YP_009380272.1 [ycf1]\| |
| cM0000030 | SSR | MonoSSR-Cp | 125519 | 125529 | A | 11 | ycf1 | YP_009380272.1 [ycf1]\| |
| cP0000252 | PotentialSSR | PentaSSR-Cp | 125684 | 125693 | AAAAC | 2 | ycf1 | YP_009380272.1 [ycf1]\| |
| cM0000031 | SSR | MonoSSR-Cp | 127107 | 127116 | T | 10 | ycf1 | YP_009380272.1 [ycf1]\| |
| cP0000253 | PotentialSSR | PentaSSR-Cp | 127292 | 127301 | CATTT | 2 | ycf1 | YP_009380272.1 [ycf1]\| |
| cP0000254 | PotentialSSR | PentaSSR-Cp | 127518 | 127527 | TTTGT | 2 | ycf1 | YP_009380272.1 [ycf1]\| |
| cH0000127 | PotentialSSR | HexaSSR-Cp | 127563 | 127574 | ATTTTG | 2 | ycf1 | YP_009380272.1 [ycf1]\| |
| cP0000255 | PotentialSSR | PentaSSR-Cp | 127584 | 127593 | TTCTT | 2 | ycf1 | YP_009380272.1 [ycf1]\| |
| cH0000128 | PotentialSSR | HexaSSR-Cp | 127605 | 127616 | GAAGGA | 2 | ycf1 | YP_009380272.1 [ycf1]\| |
| cH0000129 | PotentialSSR | HexaSSR-Cp | 127672 | 127683 | TTTCTA | 2 | ycf1 | YP_009380272.1 [ycf1]\| |
| c90000005 | ExtendedSSR | NonaSSR-Cp | 128288 | 128305 | TCCTTCTTC | 2 | ycf1 | YP_009380272.1 [ycf1]\| |
| c70000032 | ExtendedSSR | HeptaSSR-Cp | 128851 | 128864 | TTCATTC | 2 |  |  |
| cP0000256 | PotentialSSR | PentaSSR-Cp | 128963 | 128972 | TTCTG | 2 |  |  |
| c70000033 | ExtendedSSR | HeptaSSR-Cp | 129131 | 129144 | AAACATA | 2 |  |  |
| cP0000257 | PotentialSSR | PentaSSR-Cp | 129148 | 129157 | ATGAC | 2 |  |  |
| cP0000258 | PotentialSSR | PentaSSR-Cp | 129172 | 129181 | TGCTA | 2 |  |  |
| cH0000131 | PotentialSSR | HexaSSR-Cp | 129186 | 129197 | TACTTG | 2 |  |  |
| cP0000259 | PotentialSSR | PentaSSR-Cp | 129327 | 129336 | TCCAC | 2 |  |  |
| cH0000132 | PotentialSSR | HexaSSR-Cp | 129622 | 129633 | ATAAGA | 2 |  |  |
| cH0000133 | PotentialSSR | HexaSSR-Cp | 130000 | 130011 | GATAGA | 2 |  |  |
| c70000034 | ExtendedSSR | HeptaSSR-Cp | 130028 | 130041 | GGTTTTT | 2 |  |  |
| cP0000260 | PotentialSSR | PentaSSR-Cp | 130104 | 130113 | TGGAT | 2 |  |  |
| cP0000261 | PotentialSSR | PentaSSR-Cp | 130402 | 130411 | TCCGC | 2 | rrn23 | rrn23 [rrn23]\| |
| cTe0000009 | SSR | TetraSSR-Cp | 131491 | 131502 | CTAC | 3 | rrn23 | rrn23 [rrn23]\| |
| cH0000134 | PotentialSSR | HexaSSR-Cp | 131887 | 131898 | CGCTTC | 2 | rrn23 | rrn23 [rrn23]\| |
| cD0000009 | SSR | DiSSR-Cp | 131912 | 131921 | CG | 5 | rrn23 | rrn23 [rrn23]\| |
| c80000011 | ExtendedSSR | OctaSSR-Cp | 133481 | 133496 | TCTCAAAA | 2 | trnA-UGC | (Intron)trnA-UGC [trnA-UGC]\| |
| cP0000262 | PotentialSSR | PentaSSR-Cp | 133710 | 133719 | ATTGA | 2 | trnA-UGC | (Intron)trnA-UGC [trnA-UGC]\| |
| cP0000263 | PotentialSSR | PentaSSR-Cp | 134030 | 134039 | TTTGT | 2 | trnA-UGC | (Intron)trnA-UGC [trnA-UGC]\| |
| cH0000135 | PotentialSSR | HexaSSR-Cp | 134259 | 134270 | ATTCTT | 2 |  |  |
| cH0000136 | PotentialSSR | HexaSSR-Cp | 135015 | 135026 | TCCATT | 2 | trnI-GAU | (Intron)trnI-GAU [trnI-GAU]\| |
| cH0000137 | PotentialSSR | HexaSSR-Cp | 136470 | 136481 | TCAGTG | 2 | rrn16 | rrn16 [rrn16]\| |
| cH0000138 | PotentialSSR | HexaSSR-Cp | 137345 | 137356 | TCATTC | 2 |  |  |
| cP0000264 | PotentialSSR | PentaSSR-Cp | 137471 | 137480 | TCCCT | 2 | trnV-GAC | trnV-GAC [trnV-GAC]\| |
| cP0000265 | PotentialSSR | PentaSSR-Cp | 137554 | 137563 | TCTTG | 2 |  |  |
| cM0000032 | SSR | MonoSSR-Cp | 137803 | 137814 | A | 12 |  |  |
| cH0000139 | PotentialSSR | HexaSSR-Cp | 137911 | 137922 | TTTCCA | 2 |  |  |
| cH0000140 | PotentialSSR | HexaSSR-Cp | 138057 | 138068 | ATAGTA | 2 |  |  |
| cP0000266 | PotentialSSR | PentaSSR-Cp | 138181 | 138190 | CATTG | 2 |  |  |
| cP0000267 | PotentialSSR | PentaSSR-Cp | 138364 | 138373 | CTAAT | 2 |  |  |
| cP0000268 | PotentialSSR | PentaSSR-Cp | 138383 | 138392 | TAATA | 2 |  |  |
| cP0000269 | PotentialSSR | PentaSSR-Cp | 138481 | 138490 | CAACA | 2 |  |  |
| cH0000141 | PotentialSSR | HexaSSR-Cp | 138725 | 138736 | GGGATA | 2 |  |  |
| cH0000142 | PotentialSSR | HexaSSR-Cp | 138737 | 138748 | GAGGAA | 2 |  |  |
| cH0000143 | PotentialSSR | HexaSSR-Cp | 138782 | 138793 | AAAATC | 2 |  |  |
| cP0000270 | PotentialSSR | PentaSSR-Cp | 139374 | 139383 | GATTC | 2 |  |  |
| cH0000144 | PotentialSSR | HexaSSR-Cp | 139561 | 139572 | TATGGA | 2 |  |  |
| cP0000271 | PotentialSSR | PentaSSR-Cp | 140309 | 140318 | GAACA | 2 |  |  |
| cP0000272 | PotentialSSR | PentaSSR-Cp | 140414 | 140423 | TTTCT | 2 |  |  |
| cH0000145 | PotentialSSR | HexaSSR-Cp | 140478 | 140489 | TAAGAA | 2 |  |  |
| cP0000273 | PotentialSSR | PentaSSR-Cp | 141547 | 141556 | TCTTT | 2 | ndhB | (Intron)YP_009380274.1 [ndhB]\| |
| cP0000274 | PotentialSSR | PentaSSR-Cp | 141747 | 141756 | AATCA | 2 | ndhB | (Intron)YP_009380274.1 [ndhB]\| |
| cH0000146 | PotentialSSR | HexaSSR-Cp | 142041 | 142052 | GACTCT | 2 | ndhB | YP_009380274.1 [ndhB]\| |
| cH0000147 | PotentialSSR | HexaSSR-Cp | 142098 | 142109 | GCTTCA | 2 | ndhB | YP_009380274.1 [ndhB]\| |
| cP0000275 | PotentialSSR | PentaSSR-Cp | 143062 | 143071 | TTCCA | 2 |  |  |
| cP0000276 | PotentialSSR | PentaSSR-Cp | 143198 | 143207 | ATAAG | 2 |  |  |
| cH0000148 | PotentialSSR | HexaSSR-Cp | 143511 | 143522 | TTGGAA | 2 |  |  |
| cP0000277 | PotentialSSR | PentaSSR-Cp | 143688 | 143697 | CGTAA | 2 |  |  |
| cP0000278 | PotentialSSR | PentaSSR-Cp | 143933 | 143942 | AACAA | 2 |  |  |
| cP0000279 | PotentialSSR | PentaSSR-Cp | 143961 | 143970 | AACTT | 2 |  |  |
| cH0000149 | PotentialSSR | HexaSSR-Cp | 144453 | 144464 | ATATAG | 2 | ycf2 | YP_009380275.1 [ycf2]\| |
| cH0000150 | PotentialSSR | HexaSSR-Cp | 145053 | 145064 | CTTCTA | 2 | ycf2 | YP_009380275.1 [ycf2]\| |
| cP0000280 | PotentialSSR | PentaSSR-Cp | 145560 | 145569 | TTTTC | 2 | ycf2 | YP_009380275.1 [ycf2]\| |
| cH0000151 | PotentialSSR | HexaSSR-Cp | 145939 | 145950 | CAGCTC | 2 | ycf2 | YP_009380275.1 [ycf2]\| |
| cP0000281 | PotentialSSR | PentaSSR-Cp | 146583 | 146592 | CAATC | 2 | ycf2 | YP_009380275.1 [ycf2]\| |
| cH0000152 | PotentialSSR | HexaSSR-Cp | 146603 | 146614 | CTTTTT | 2 | ycf2 | YP_009380275.1 [ycf2]\| |
| cP0000282 | PotentialSSR | PentaSSR-Cp | 147561 | 147570 | TTGAA | 2 | ycf2 | YP_009380275.1 [ycf2]\| |
| cP0000283 | PotentialSSR | PentaSSR-Cp | 147726 | 147735 | GATCG | 2 | ycf2 | YP_009380275.1 [ycf2]\| |
| c90000006 | ExtendedSSR | NonaSSR-Cp | 148565 | 148582 | AAATGTTCC | 2 | ycf2 | YP_009380275.1 [ycf2]\| |
| cP0000284 | PotentialSSR | PentaSSR-Cp | 149852 | 149861 | CGGAT | 2 | ycf2 | YP_009380275.1 [ycf2]\| |
| cH0000155 | PotentialSSR | HexaSSR-Cp | 149903 | 149914 | TCTTTC | 2 | ycf2 | YP_009380275.1 [ycf2]\| |
| cP0000285 | PotentialSSR | PentaSSR-Cp | 150570 | 150579 | ATTTC | 2 |  |  |
| cP0000286 | PotentialSSR | PentaSSR-Cp | 150787 | 150796 | TCATA | 2 |  |  |
| cP0000287 | PotentialSSR | PentaSSR-Cp | 151145 | 151154 | TATTC | 2 |  |  |
| cP0000288 | PotentialSSR | PentaSSR-Cp | 151165 | 151174 | AAAGA | 2 |  |  |
| cP0000289 | PotentialSSR | PentaSSR-Cp | 151266 | 151275 | AATCC | 2 | rpl2 | YP_009380276.1 [rpl2]\| |
| cP0000290 | PotentialSSR | PentaSSR-Cp | 151615 | 151624 | ACATA | 2 | rpl2 | YP_009380276.1 [rpl2]\| |
| cH0000156 | PotentialSSR | HexaSSR-Cp | 152028 | 152039 | GAAAAT | 2 |  |  |
